# Supplementary figures and images for: Structure of the siphophage neck–Tail complex suggests that conserved tail tip proteins facilitate receptor binding and tail assembly
Source: PLoS Biol. 2023 Dec 14;21(12):e3002441. doi: 10.1371/journal.pbio.3002441 (PMC10721106; doi:10.1371/journal.pbio.3002441)

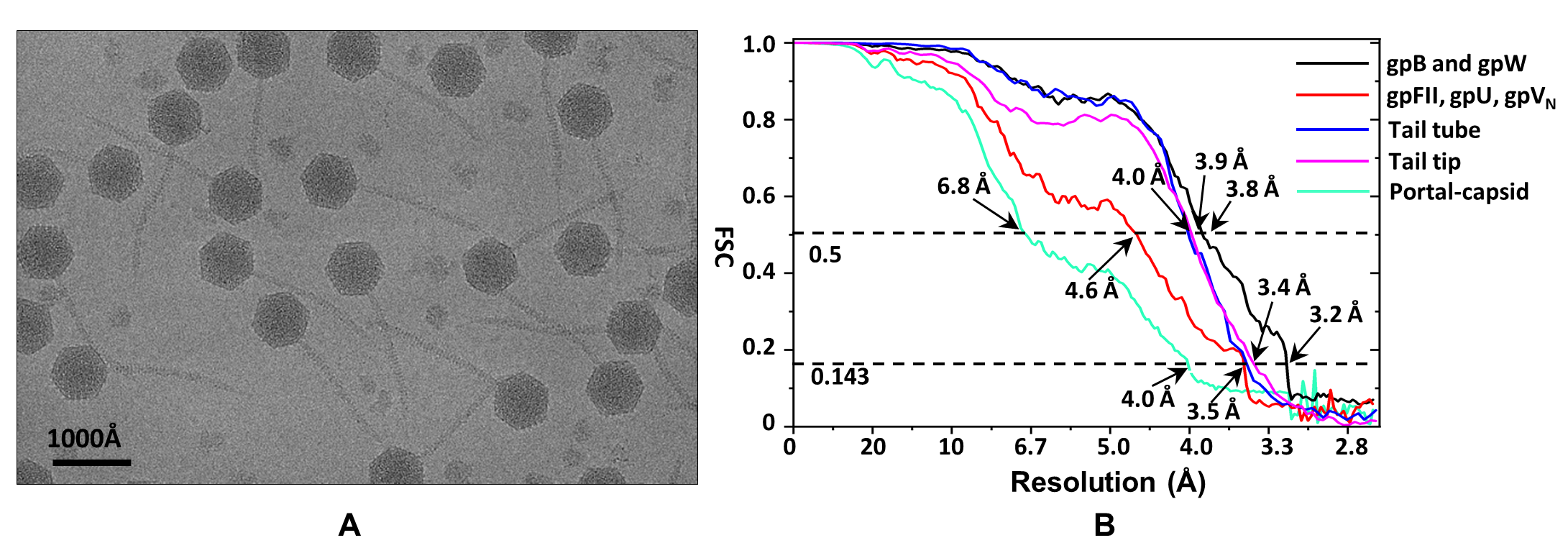

Supplement: S1 Fig — (A) Cryo-EM image of mature lambda phage showing its long flexible tail. (B) Estimated structural resolutions of the local reconstructions of the gpB and gpW with an imposed symmetry of 12 folds (black line), the gpFII, gpU, and gpVN with an imposed symmetry of 6 folds (red line), the tail tube (gpVN) with an imposed symmetry of 6 folds (blue line), the tail tip complex with an imposed symmetry of 3 folds (magenta line), and the portal–capsid without symmetry imposed (cyan line). (TIF) [file pbio.3002441.s001.tif]

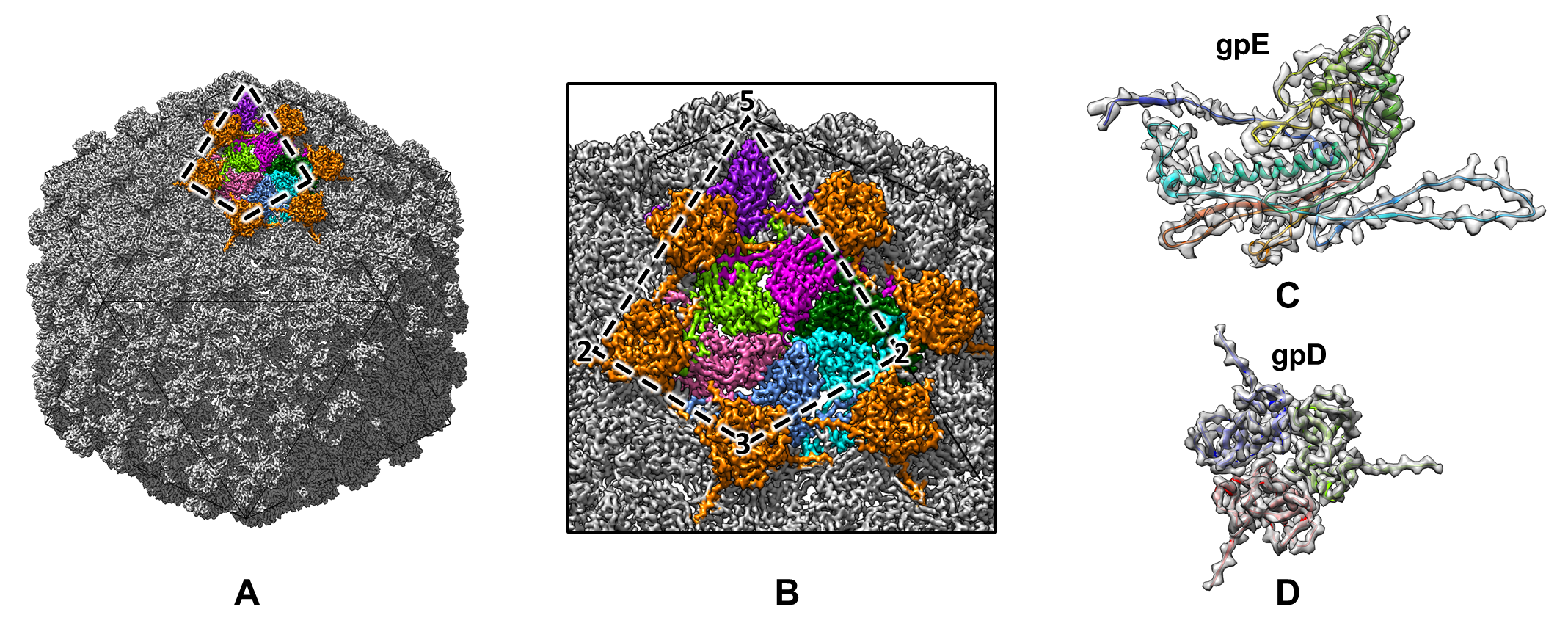

Supplement: S2 Fig — (A) Overall view of the icosahedral head structure. Seven copies of the major capsid protein gpE arranged in an asymmetric unit are shown in purple, green, hot pink, cornflower blue, cyan, dark green, and magenta, and 6 trimers of the cementing protein gpD surrounding the asymmetric unit are shown in orange. (B) Zoomed-in view of the asymmetric unit in panel (A). The 5-, 3-, and 2-fold axes are labeled. (C, D) Density maps (grey) of the major capsid protein gpE and the trimer of the cementing protein gpD superimposed on their atomic models (ribbons). (TIF) [file pbio.3002441.s002.tif]

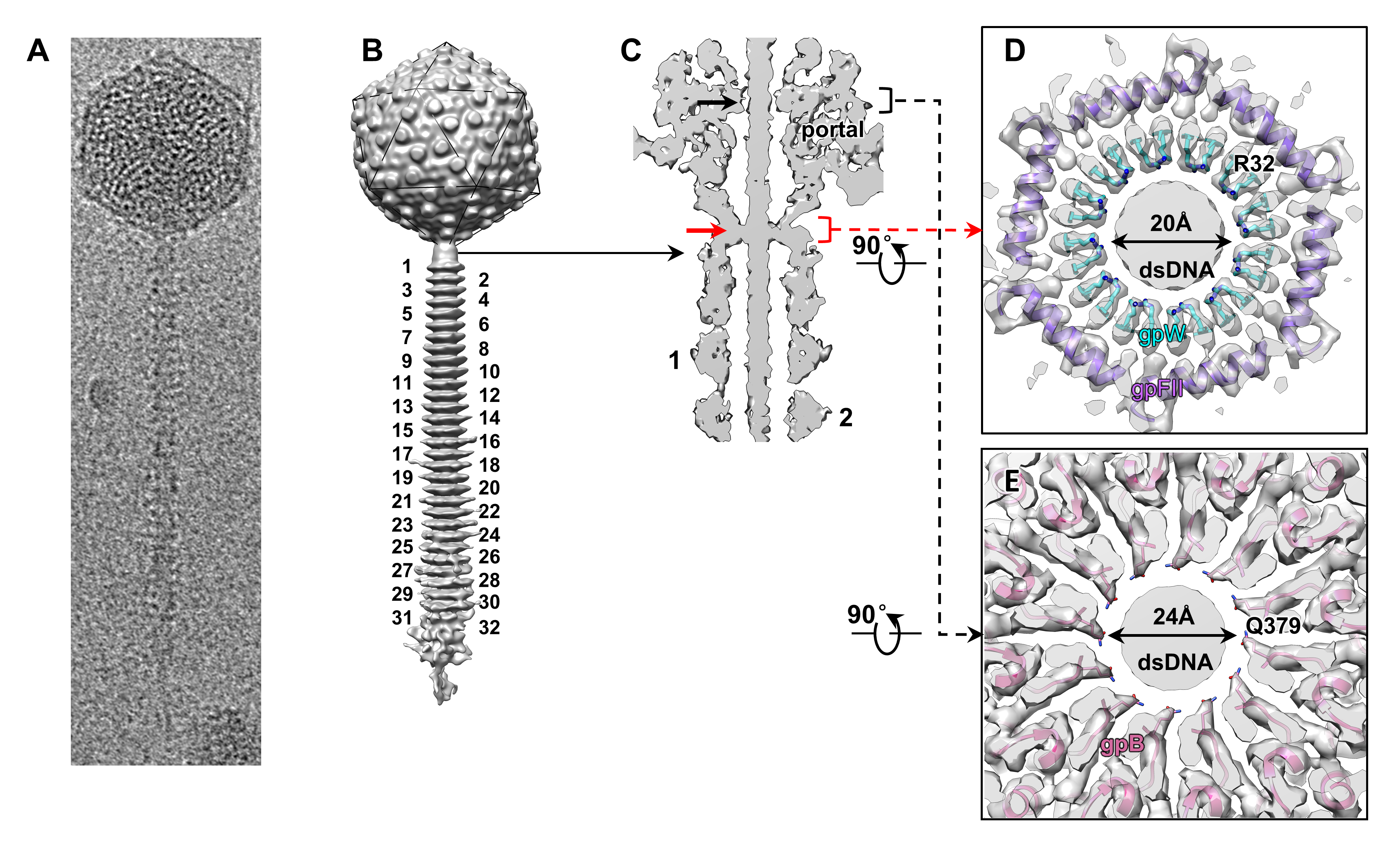

Supplement: S3 Fig — (A) Image of a lambda particle with a straight tail. (B) Overall view of an intact lambda phage at a resolution of approximately 20 Å. The numbers of the tail rings are labeled. (C) Slab view of the neck showing the DNA within the neck (red arrow). (D) Density map (transparent) at a resolution of 3.5 Å superimposed on the atomic models of gpW (cyan ribbon) and gpFII (purple ribbon), showing the interactions between the Arg32 residue of gpW and dsDNA. (E) Density map (transparent) at a resolution of 3.5 Å superimposed on the atomic model of gpB (hot pink ribbon), showing the interactions between the Gln379 residue of gpB and dsDNA. (TIF) [file pbio.3002441.s003.tif]

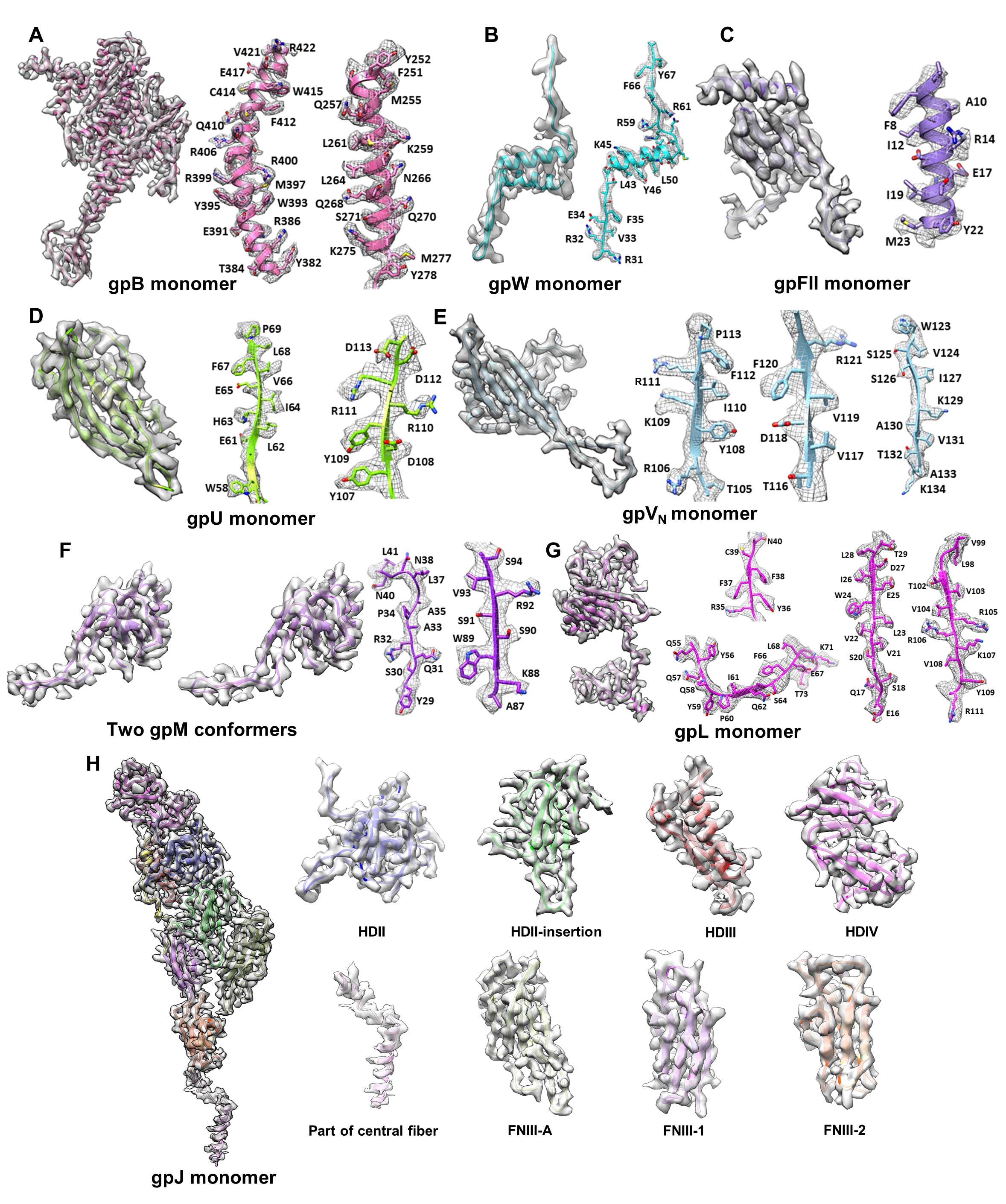

Supplement: S4 Fig — Density maps (transparent or mesh) of proteins gpB (A), gpW (B), gpFII (C), gpU (D), gpVN (E), gpM (F), gpL (G), and gpJ (H) superimposed on their atomic models. (TIF) [file pbio.3002441.s004.tif]

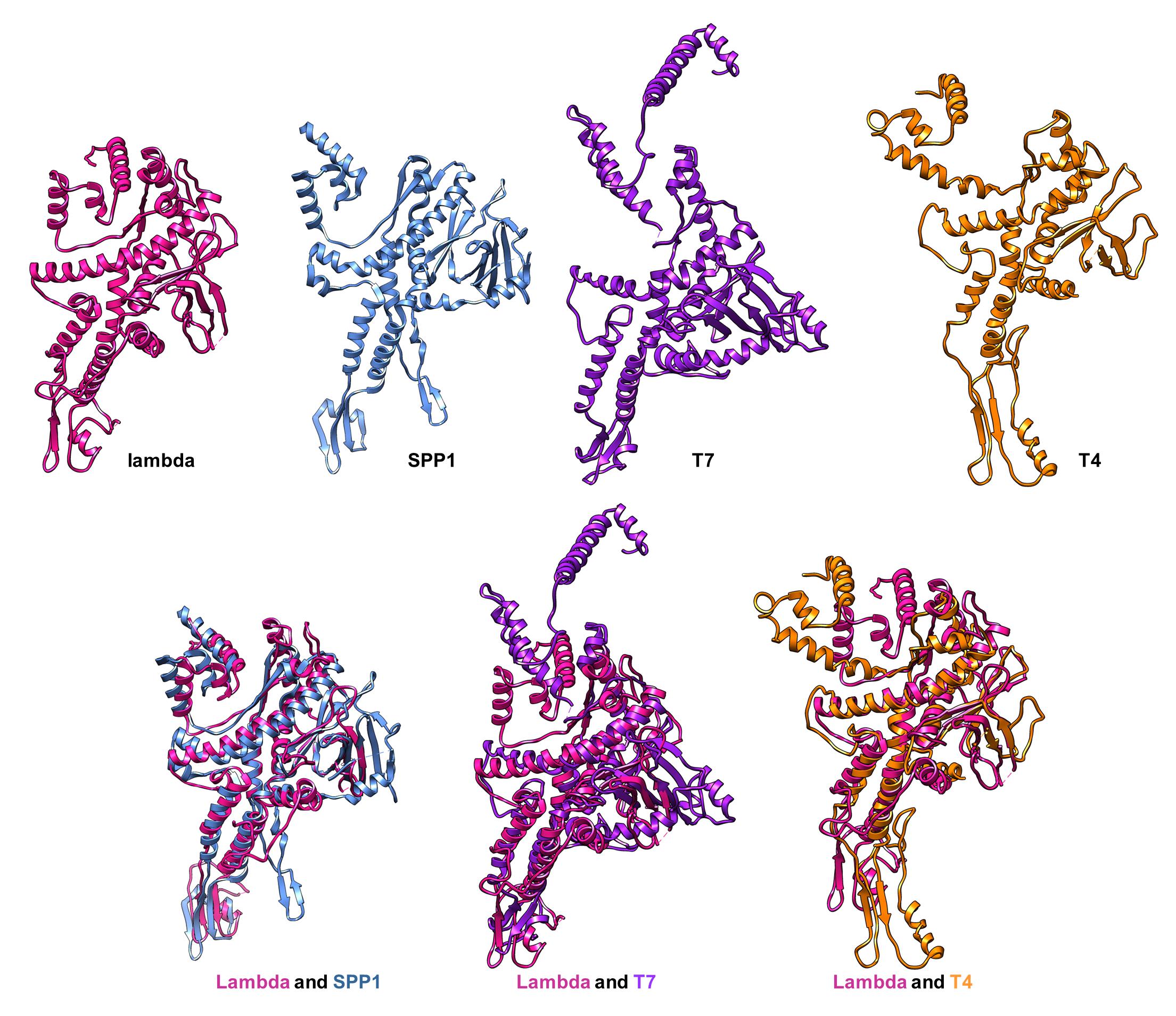

Supplement: S5 Fig — The RMSDs between the portal proteins of lambda and SPP1, T7, and T4 are 2.52, 2.48, and 2.03 Å, respectively. (TIF) [file pbio.3002441.s005.tif]

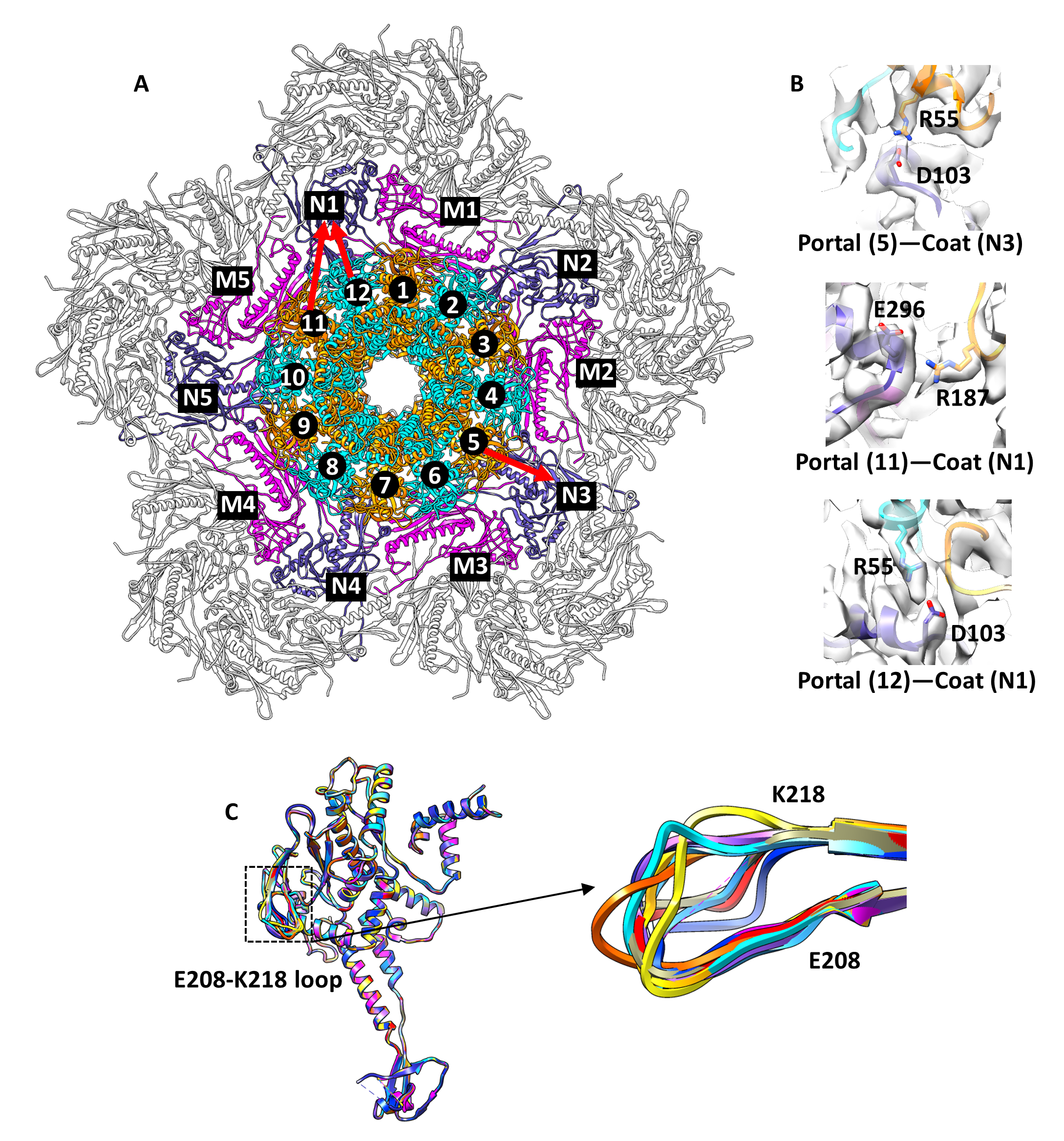

Supplement: S6 Fig — (A) A schematic representation of the portal and the surrounding capsomers. The 12 portal gpB subunits (identified as 1 to 12) are in orange and cyan. The 5 M subunits (M1 to M5) and 5 N subunits (N1 to N5) of the major capsid protein gpE are in magenta and dark blue. The red arrowheads indicate salt bridges between the portal and coat subunits. (B) Salt bridges between the portal and coat subunits. The atomic models (ribbon) are superimposed on their density maps (transparent). (C) Superimposition of the 12 portal subunits revealed the structural morphing loop (residues 208–218) in gpB. (TIF) [file pbio.3002441.s006.tif]

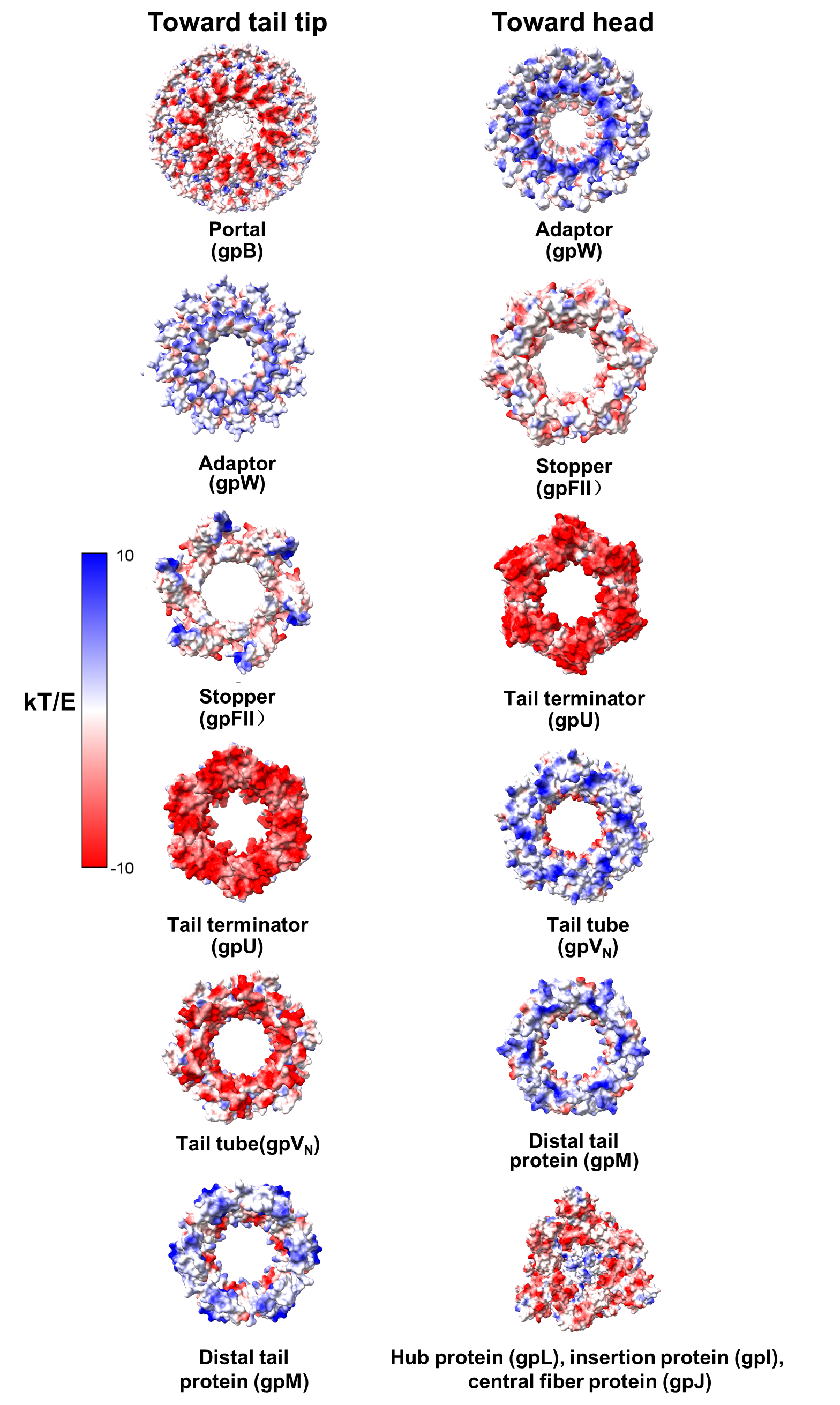

Supplement: S7 Fig — The color scale of the electrostatic potential range is the same for all protein surfaces. The rings in the left column are oriented towards the tail tip, and the rings in the right column are oriented towards the head. (TIF) [file pbio.3002441.s007.tif]

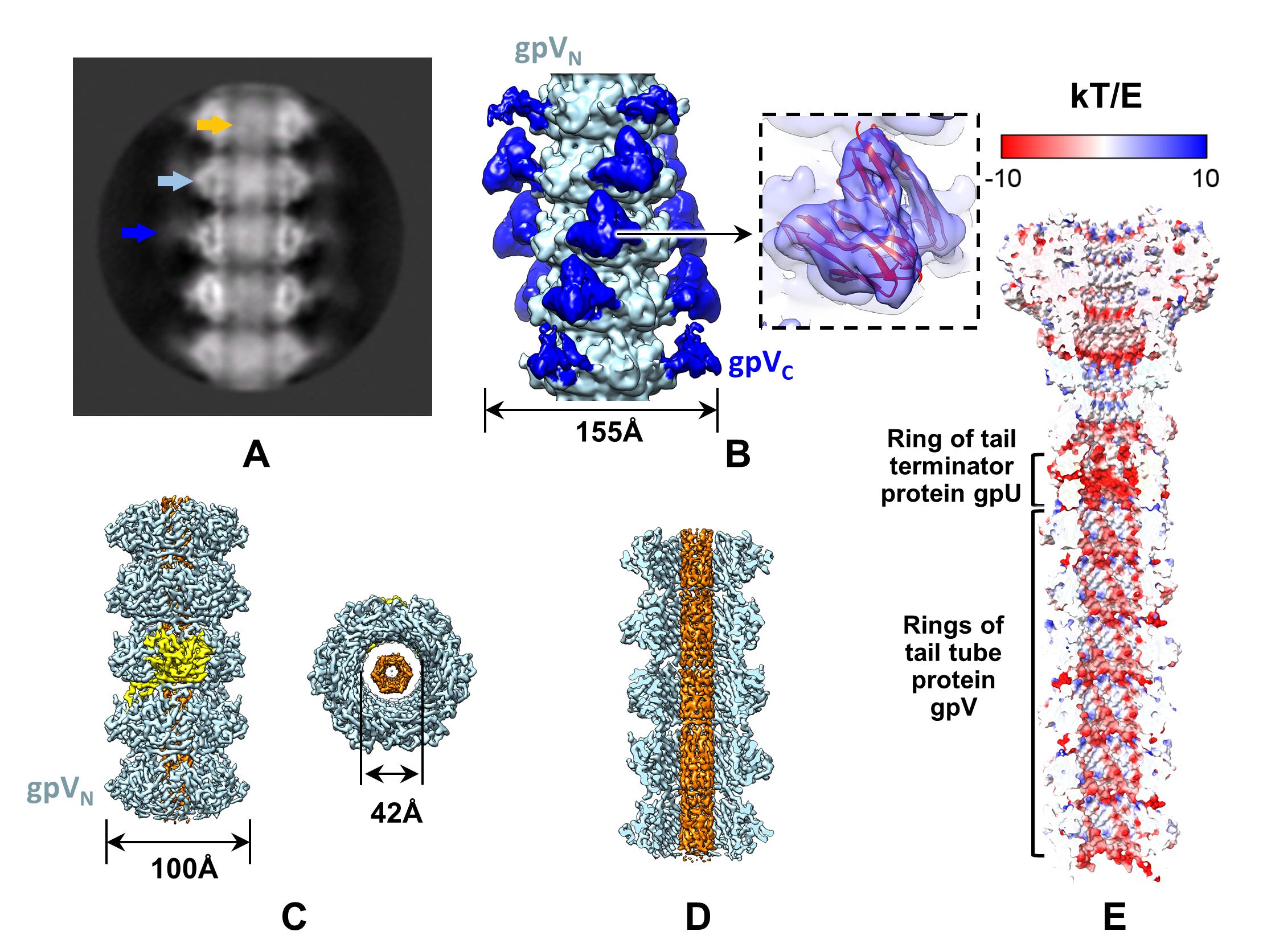

Supplement: S8 Fig — (A) Two-dimensional class average of the tail tube. White, cyan, and orange arrows indicate the rings of gpVN and gpVC and the rod of gpH or dsDNA, respectively. (B) Density map of the tail tube filtered to a resolution of 6 Å. Superposition of 2 copies of gpVC atomic model (PDB DI: 2L04) on the protrusion (right) revealed that each protrusion contains 2 gpVC domains contributed by 2 neighboring gpV subunits. (C) Side and top views of the density map of the tail tube at 3.5 Å resolution. The protrusions were poorly resolved at this resolution. One monomer of gpVN is shown in yellow. (D) Cut-open view of the tail tube. (E) Electron potential on the inner channel of the neck–tail complex. (TIF) [file pbio.3002441.s008.tif]

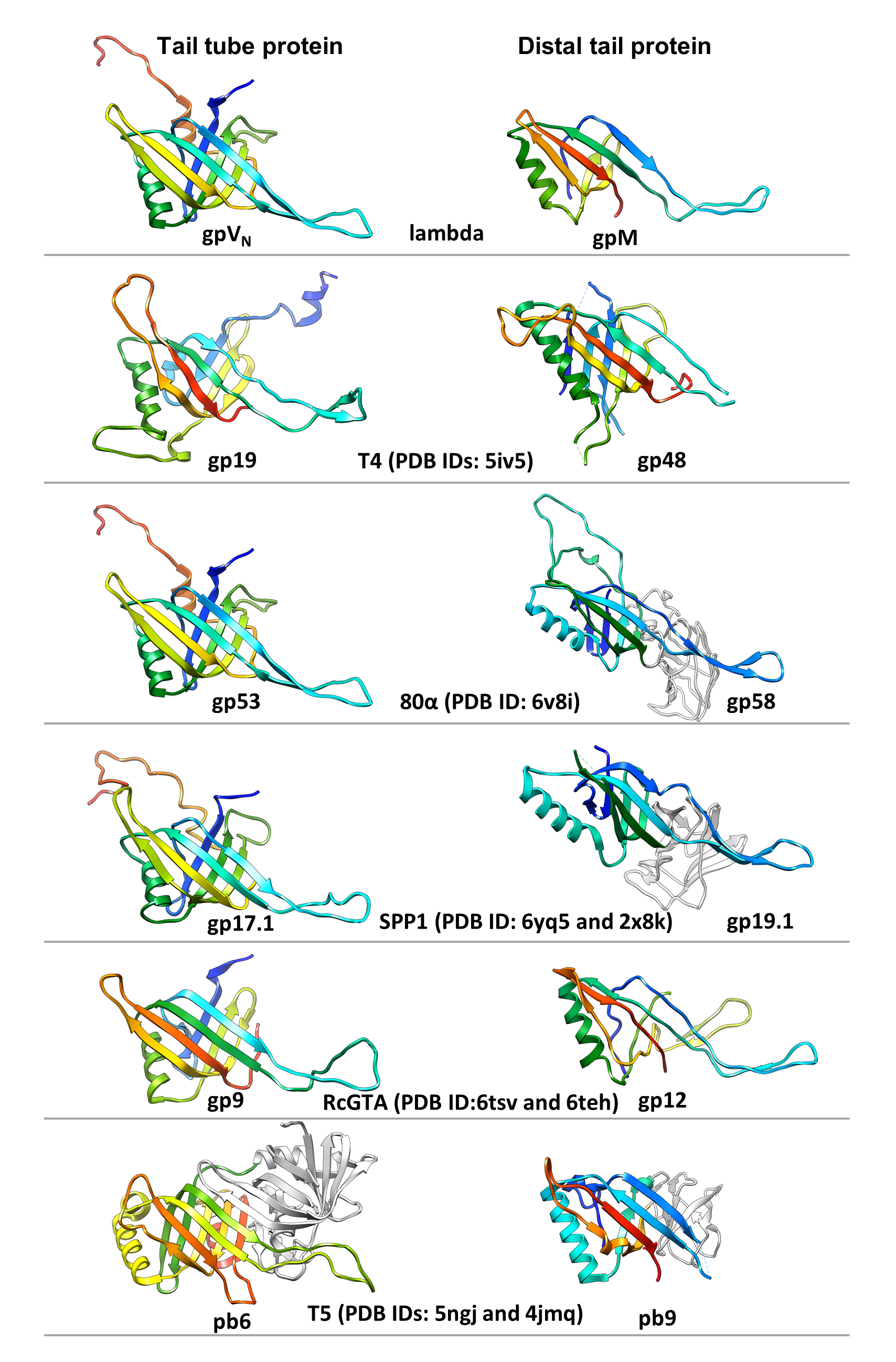

Supplement: S9 Fig — All models are shown in rainbow colors, ranging from blue at the N-termini to red at the C-termini, and the redundant parts are shown in grey. (TIF) [file pbio.3002441.s009.tif]

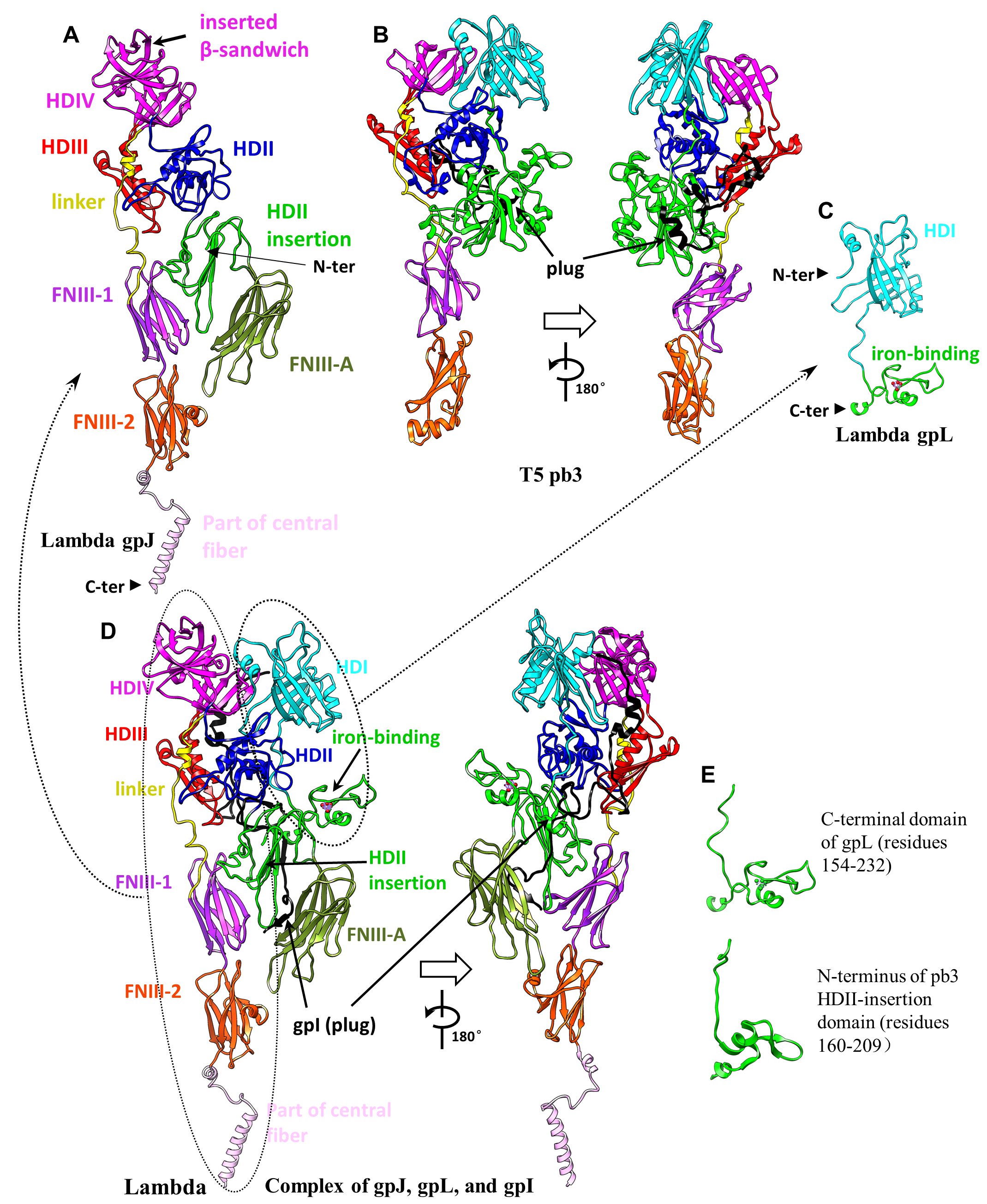

Supplement: S10 Fig — The color code for domains is identical to that in Fig 4G. (A) Structure of gpJ. (B) Structure of T5 BHP pb3. (C) Structure of gpL. (D) Complex of a copy of gpJ, gpL, and gpI in the lambda tail tip. (E) Structural comparison between the C-terminal domain of the lambda gpL (residues154-232) and the N-terminus of the T5 pb3 HDII-insertion domain shows that they are topologically similar. (TIF) [file pbio.3002441.s010.tif]

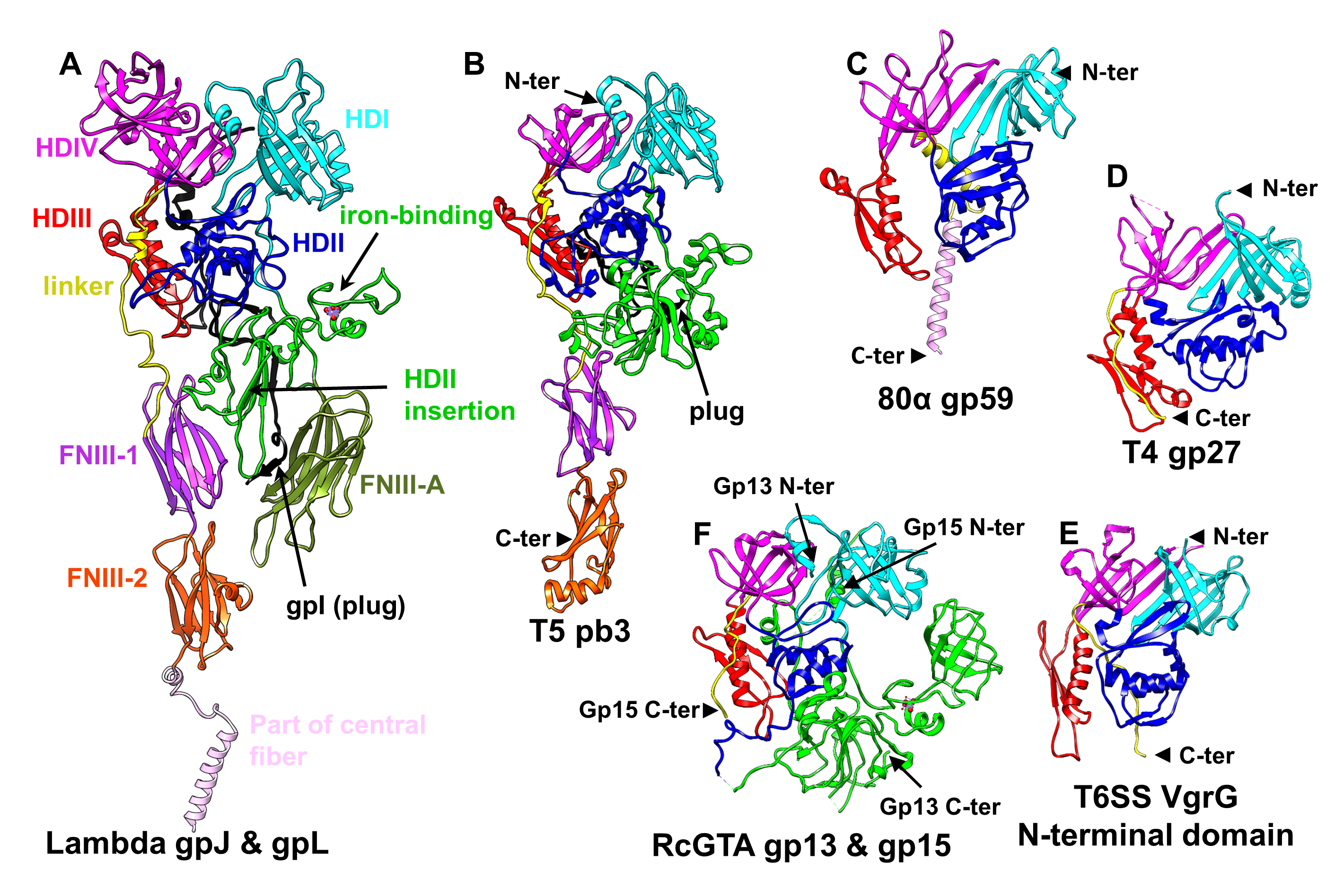

Supplement: S11 Fig — (A-F) Structure of the hub and central fiber proteins of lambda (gpL and gpJ), the baseplate hub or homologous proteins of phage T5 (7ZQB), phage 80α (PDB ID: 6V8I), T4 (PDB ID: 5IV5), T6SS (PDB ID: 6H3L), and RcGTA (PDB ID: 6TEH). Protein VgrG of T6SS belongs to the species Pseudomonas aeruginosa. The homologous domains in these proteins are in the same color. The N- and C- termini of these proteins were labeled. For the N- and C- termini of gpL and gpJ, see S10A and S10C Fig for separate views of gpJ and gpL. (TIF) [file pbio.3002441.s011.tif]

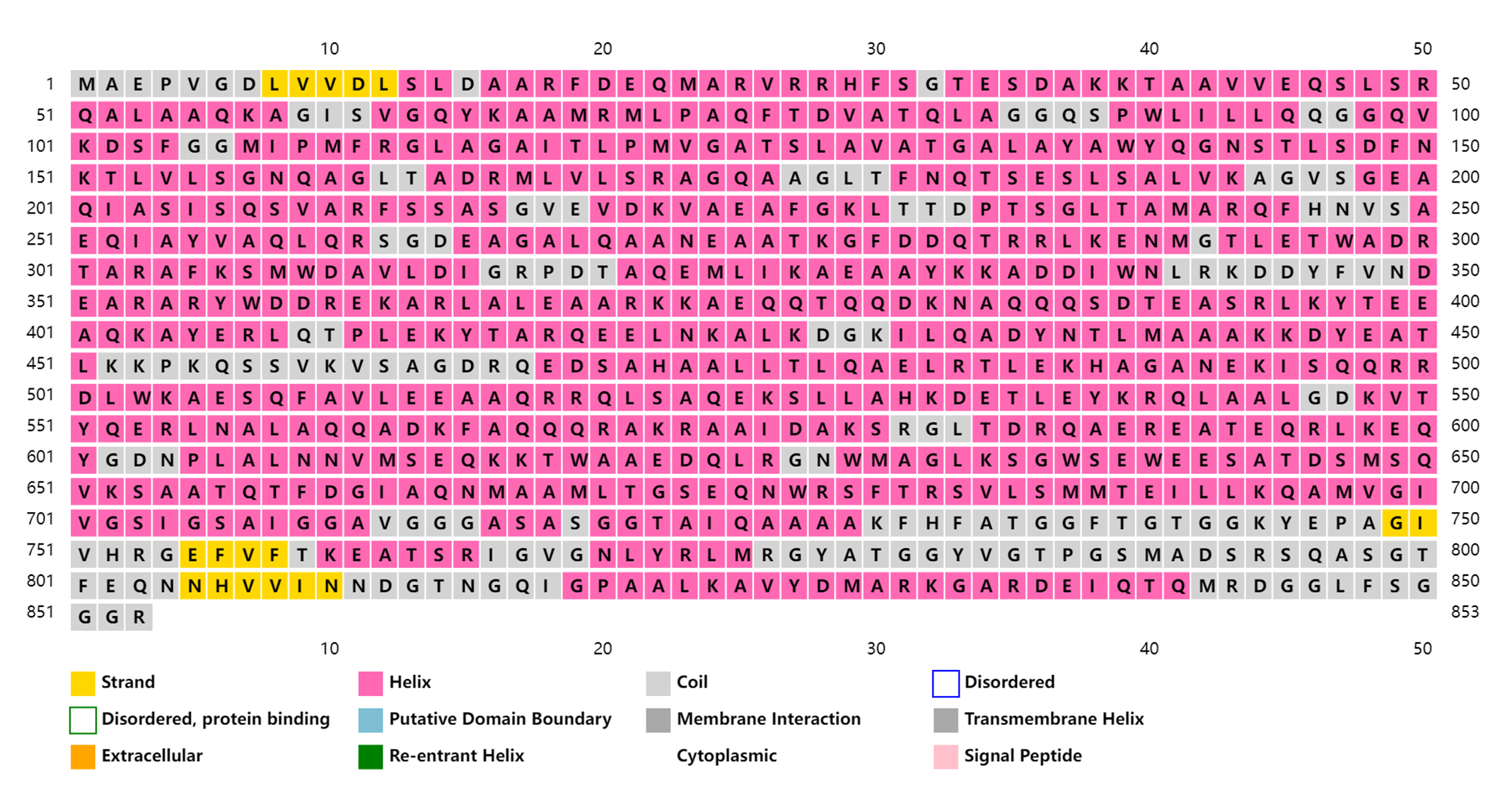

Supplement: S12 Fig — (TIF) [file pbio.3002441.s012.tif]

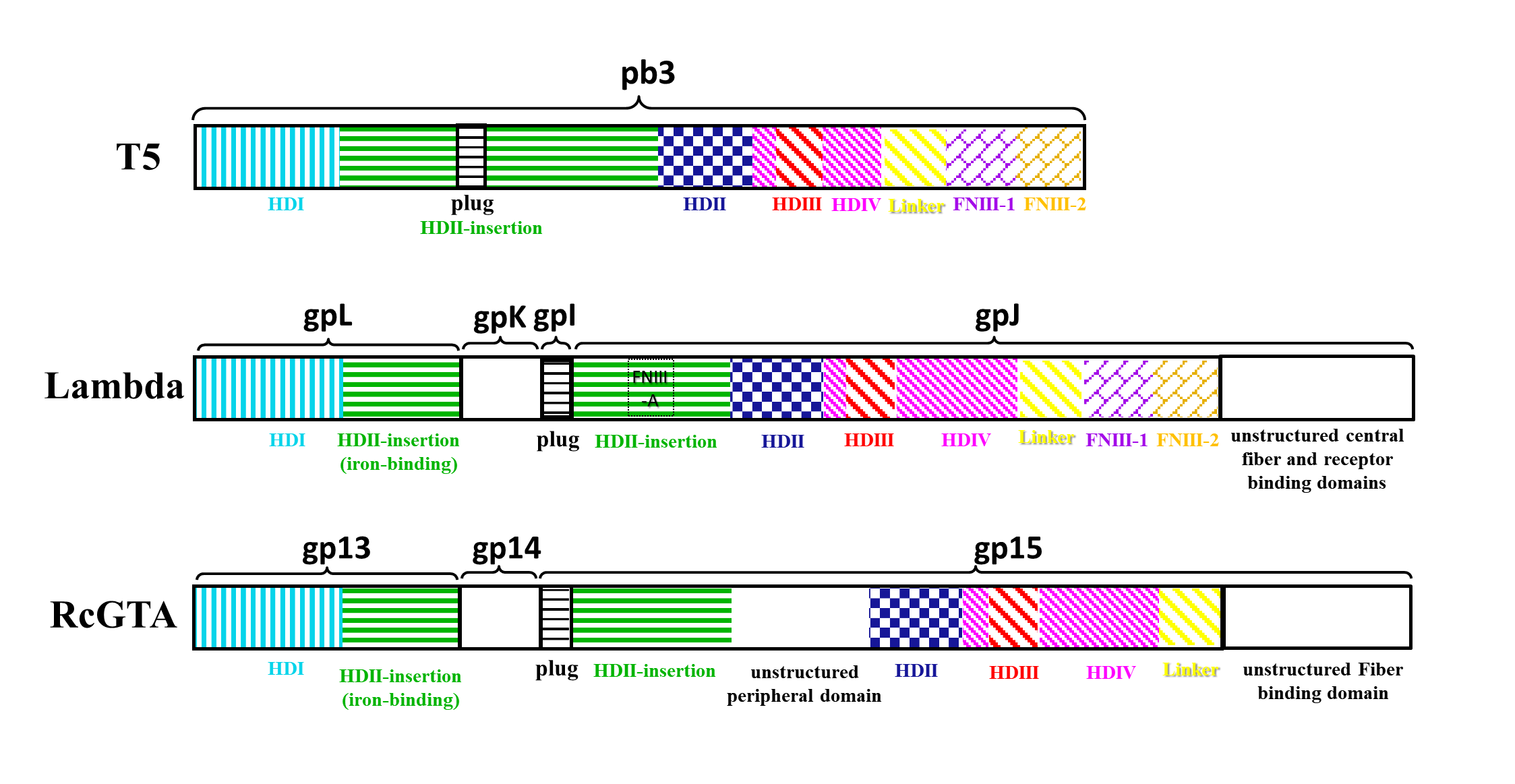

Supplement: S13 Fig — Genes encoding proteins of homologous domains are shown in the same pattern and color. The regions in white are unstructured. (TIF) [file pbio.3002441.s013.tif]

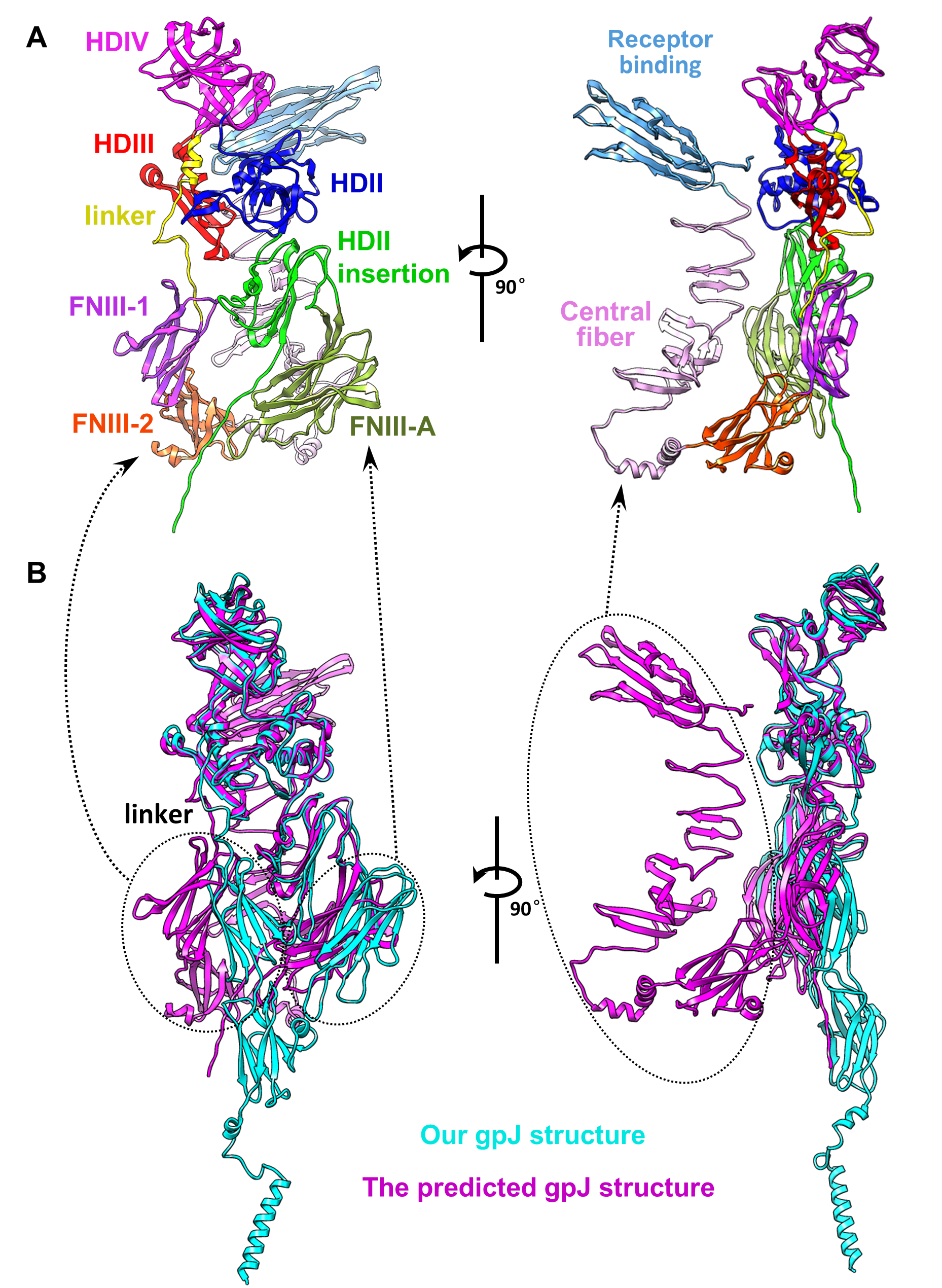

Supplement: S14 Fig — (A) Structure of the gpJ structure predicted by AlphaFold. The color code for domains is identical to that in Fig 4G except that the central fiber and receptor binding domains are in pink and cornflower blue, respectively. (B) Comparison of our gpJ structure (cyan) and predicted gpJ structure (magenta) shows the conformational change between them. (TIF) [file pbio.3002441.s014.tif]

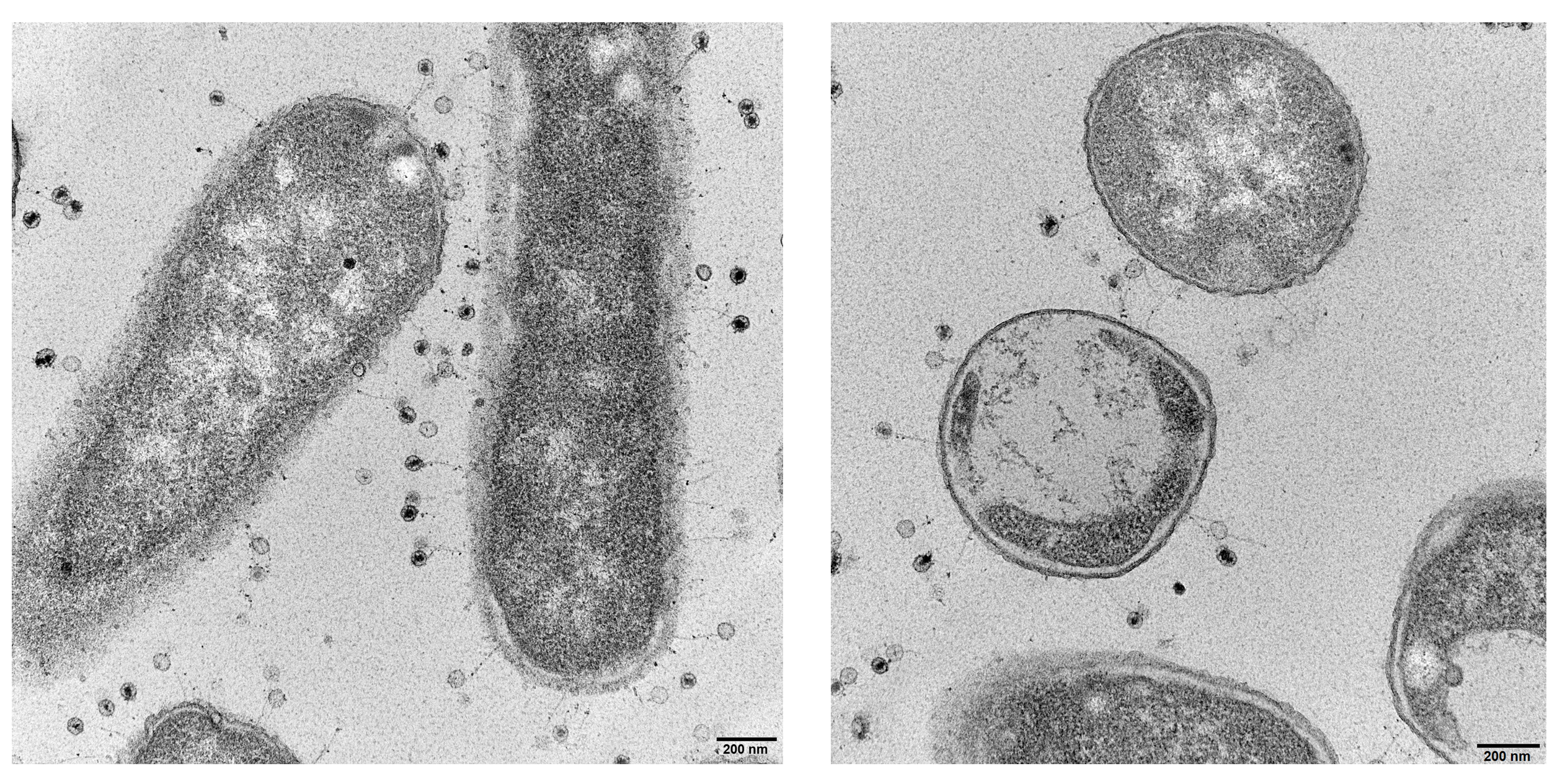

Supplement: S15 Fig — The scale bars represent 200 nm. (TIF) [file pbio.3002441.s015.tif]

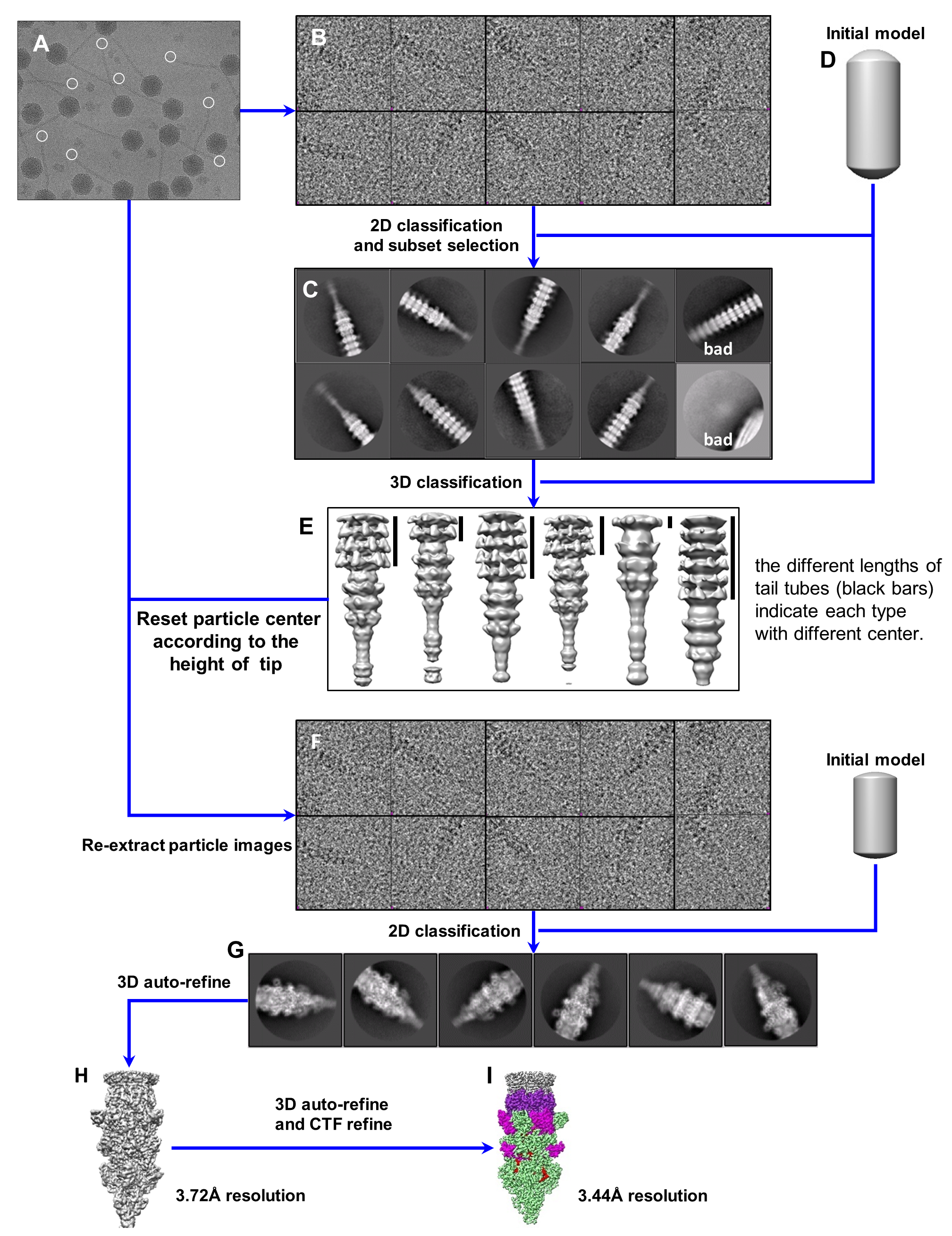

Supplement: S16 Fig — First, we manually selected a total of 70,745 tail tip particles (A, B) and performed 2D classification to exclude irrelevant images (C). Then, using a randomly generated cylinder density as the initial model (D), we selected a total of 58,215 particles from the 2D classifications to perform 3D classification with a C3 symmetry and obtained a total of 6 types of low-resolution structures (E). Third, we reextracted tail tip images (F) according to the centers of each type. Fourth, we performed 2D classification (G) and 3D auto-refinement with a C3 symmetry to obtain a density map of the tail tip complex at a resolution of 3.72 Å (H). Finally, we refined the contrast transfer function to improve the resolution of the tail tip structure to 3.44 Å (I). (TIF) [file pbio.3002441.s016.tif]
