# Supplementary material for: Structure of the siphophage neck–Tail complex suggests that conserved tail tip proteins facilitate receptor binding and tail assembly
Source: PLoS Biol. 2023 Dec 14;21(12):e3002441. doi: 10.1371/journal.pbio.3002441 (PMC10721106; doi:10.1371/journal.pbio.3002441)
Supplement: S1 Table — (PDF) [file pbio.3002441.s017.pdf]

S1 Table. Modeled proteins of the siphophage lambda.

Protein ID	Protein function	Amino acid length	MW(kDa）	Number of copies	Modeled residues
gpB	Portal	**533**	**59.5**	**12**	**24-302<br>320-513**
gpW	Adaptor	**68**	**7.6**	**12**	**4-68**
gpFII	Stopper	**117**	**12.8**	**6**	**4-117**
gpU	Tail terminator	**131**	**14.6**	**6**	**1-131**
gpV	Tail tube	**246**	**25.8**	**192**	**3-156**
gpH	Tape measure protein	**853**	**92.3**	**3**	**818-849**
gpM	Distal tail protein	**117**	**12.5**	**6**	**1-109**
gpL	Hub protein	**232**	**25.7**	**3**	**1-232**
gpI	Insertion protein	**223**	**23.1**	**3**	**135-223**
gpJ	Central fiber protein	**1132**	**124.4**	**3**	**1-851**
gpD	Cementing protein	**110**	**11.6**	**420**	**2-109**
gpE	Coat protein	**341**	**38.2**	**415**	**5-341**
