# Supplementary material for: Structure of the siphophage neck–Tail complex suggests that conserved tail tip proteins facilitate receptor binding and tail assembly
Source: PLoS Biol. 2023 Dec 14;21(12):e3002441. doi: 10.1371/journal.pbio.3002441 (PMC10721106; doi:10.1371/journal.pbio.3002441)
Supplement: S2 Table — (PDF) [file pbio.3002441.s018.pdf]

S2 Table. Detected proteins of the siphophage lambda by mass spectrometry.

Proteins	Description	No. unique peptides	Peptides	Coverage/%	Score
gpE	Coat protein	**27**	**36**	**65.69**	**473.44**
gpH	Tape measure protein	**53**	**53**	**50.06**	**156.00**
gpV	Tail tube protein	**17**	**17**	**67.48**	**120.37**
gpB	Portal protein	**14**	**24**	**54.41**	**108.62**
gpD	Cementing protein	**8**	**8**	**68.18**	**106.62**
gpJ	Central fiber protein	**48**	**48**	**43.82**	**93.73**
gpZ	Tail completion protein	**10**	**10**	**48.44**	**21.47**
gpC	Protease protein	**8**	**8**	**28.25**	**21.18**
gpM	Distal tail protein	**7**	**7**	**66.06**	**19.01**
gpW	Adaptor protein	**6**	**6**	**69.12**	**18.94**
gpFII	Stopper protein	**6**	**6**	**74.36**	**11.92**
gpL	Hub protein	**8**	**8**	**43.53**	**7.58**
gpI	Insertion protein	**1**	**1**	**9.87**	**3.81**
gpU	Tail terminator protein	**2**	**2**	**15.27**	**3.76**
