# Supplementary material for: Structure of the siphophage neck–Tail complex suggests that conserved tail tip proteins facilitate receptor binding and tail assembly
Source: PLoS Biol. 2023 Dec 14;21(12):e3002441. doi: 10.1371/journal.pbio.3002441 (PMC10721106; doi:10.1371/journal.pbio.3002441)
Supplement: S3 Table — For each protein, the most relevant hits are shown with the matched residues, Protein Data Bank ID, chain identifier, HHpred probability (%), E-value, and percent sequence identity in the matched regions. (PDF) [file pbio.3002441.s019.pdf]

S3 Table. Results of the HHpred analysis of the tail protein sequences of lambda phage. For each protein, the most relevant hits are shown with the matched residues, Protein Data Bank ID, chain identifier, HHpred probability (%), E-value, and percent sequence identity in the matched regions.

	**Residues**	**Match**	**PDB ID**	**Probability (%)**	**E-value**	**Sequence Identity (%)**
**gpV$_N$**	1-136	Rhodobacter capsulatus RcGTA g9	6TE9_G	99.81	4.3e-18	14
	1-140	Bacillus siphophage SPP1 gp17.1	6YQ5_L	99.63	4.6e-14	13
	1-141	Staphylococcus virus 80α gp53	6Y8A_FA	99.47	3.9e-12	11
	1-91	Salmonella Typhi YSD1	6XGR_M	85.47	6.4	15
	1-147	Escherichia phage T5 pb9	5NGJ_A	73.62	54	10
	36-160	Enterobacteria phage T4 gp19	5IV5_IB	48.82	90	12
**gpM**	2-84	Rhodobacter capsulatus RcGTA g12	6TEH_B	99.59	7.6e-15	17
	11-85	Escherichia phage T5 pb6	6F2M_B	95.86	0.05	11
**gpL**	3-140	Rhodobacter capsulatus RcGTA g13	6TEH_C	99.31	6.1e-11	11
**gpJ**	51-979	Rhodobacter capsulatus RcGTA g15	6TEH_D	99.86	1.2e-19	13
	92-329	Staphylococcus virus 80 α gp59	6V8I_CE	91.43	13	9
	208-373	Escherichia phage T4 gp27	1WTH_D	54.34	93	11
