# Supplementary material for: Structure of the siphophage neck–Tail complex suggests that conserved tail tip proteins facilitate receptor binding and tail assembly
Source: PLoS Biol. 2023 Dec 14;21(12):e3002441. doi: 10.1371/journal.pbio.3002441 (PMC10721106; doi:10.1371/journal.pbio.3002441)
Supplement: S4 Table — (PDF) [file pbio.3002441.s020.pdf]

S4 Table. Refinement and model statistics.

**Data collection**					
Electron microscopy	FEI 300 kV Titan Krios G3i, Gantan K3 camera				
Pixel size (Å)	1.36				
Defocus range	-1.6 to -2.2µm				
Electron exposure	32 e-/Å2				
Total micrographs	5,533				
**Local reconstruction**					
	Portal-capsid	Portal-adaptor	Neck	Tail tube	Tail tip complex
Symmrtry imposed	C1	C12	C6	C3	C3
Total particles	97,030	97,030	97,030	181,657	70,745
Final particles	72,032	72,868	75,098	121,571	54,385
Resolution(Å)	4	3.2	3.5	3.48	3.44
B-factors	90	80	80	145	160
EMDB ID	EMD-36848	EMD-36847	EMD-36846	EMD-36845	EMD-36844
**Atomic models refinement/statistics (phenix.real_space_refine）**					
	Portal-capsid	Portal-adaptor	Neck	Tail tube	Tail tip complex
PDB ID	8K39	8K38	8K37	8K36	8K35
Initial model used (PDB code)	ab-initio	ab-initio	ab-initio	ab-initio	ab-initio
Correlation coefficient (model to map fit)	0.8328	0.8762	0.8487	0.8688	0.8486
**Model composition**					
Number of chains	42	24	18	12	27
Nonhydrogen atoms	124618	50172	18528	14172	40347
Residues	158749	6396	2394	1848	5184
**R.m.s. deviations**					
Bond lengths	0.004	0.002	0.004	0.007	0.004
Bond angles	0.645	0.466	0.591	0.663	0.591
**Validation**					
MolProbity score	1.81	1.21	1.4	1.04	1.4
Clash score	7.57	4.34	5.13	2.52	5.16
Rotamer outliers (%)	0.02	0	0	0.27	0
**Ramachandran plot (%)**					
Favored	94.13	98.35	97.31	98.9	97.33
Allowed	5.67	1.65	2.69	1.1	2.67
Outliers	0.2	0	0	0	0
