# Supplementary material for: Structure of the siphophage neck–Tail complex suggests that conserved tail tip proteins facilitate receptor binding and tail assembly
Source: PLoS Biol. 2023 Dec 14;21(12):e3002441. doi: 10.1371/journal.pbio.3002441 (PMC10721106; doi:10.1371/journal.pbio.3002441)
Supplement: Attachment — Submitted filename: PBIOLOGY-D-23-00002_R2.pdf [file pbio.3002441.s023.pdf]

PLOS Biology

Symmetry-mismatch structure of the bacteriophage lambda

--Manuscript Draft--

**Manuscript Number:**	PBIOLOGY-D-23-00002R2
**Full Title:**	Symmetry-mismatch structure of the bacteriophage lambda
**Short Title:**	Cryo-EM of the siphophage lambda
**Article Type:**	Short Reports
**Keywords:**	
**Corresponding Author:**	Hongrong Liu<br>Hunan Normal University<br>Changsha, CHINA
**First Author:**	Hao Xiao
**Order of Authors:**	Hao Xiao
	Le Tan
	Zhixue Tan
	Yewei Zhang
	Wenyuan Chen
	Xiaowu Li
	Jingdong Song
	Lingpeng Cheng
	Hongrong Liu
**Abstract:**	Siphophages have a long, flexible, and noncontractile tail that connects to the capsid through a neck. The phage tail is essential for host cell recognition and virus-host cell interactions; moreover, it serves as a channel for genome delivery during infection. However, the in situ high-resolution structure of the neck-tail complex of siphophages remains unknown. Here we present the in situ structure of the neck-tail complex of the siphophage lambda wild type, which lacks side tail fiber. The neck-tail complex comprises a channel formed by stacked 12-fold and 6-fold symmetrical rings and a 3-fold symmetrical tip. The interactions among DNA and a total of 246 tail protein molecules responsible for neck-tail assembly and stabilization have been characterized. The DNA in the neck is clamped by the adaptor protein rather than the stopper protein. Structural comparisons of the tail tips, the most diversified region across the lambda and other long-tailed phages or tail-like machines suggest that their tail tip contains conserved domains, which facilitate tail assembly, receptor binding, cell adsorption, and DNA retaining/releasing. These domains are distributed in different tail tip proteins in different phages or tail-like machines. The side tail fi[illegible]s are not required for the perpendicular adsorption of lambda.
**Suggested Reviewers:**	Timothy Baker<br>University of California San Diego<br>tsb@ucsd.edu<br>Prof. Timothy Baker's expertise: Structural biology of dsRNA virus structure and high-resolution cryo-EM.<br><br>Website: http://bakerlab.ucsd.edu/index.htm
	Jun Liu<br>Yale School of Medicine: Yale University School of Medicine<br>jliu@yale.edu<br>Prof. Jun Liu's expertise: Structural virology; Bacteriophage.<br><br>Website: https://medicine.yale.edu/bbs/molecularcell/profile/jliu/

Powered by Editorial Manager® and ProduXion Manager® from Aries Systems Corporation

	Wen Jiang<br>Purdue University<br>jiang12@purdue.edu<br>Prof. Wen Jiang's expertise: Structural biology of viruses and large macromolecular complexes.<br><br>Website: https://www.purdue.edu/gradschool/pulse/groups/profiles/faculty/jiang.html
	Juha T Huiskonen<br>University of Oxford<br>juha@strubi.ox.ac.uk<br>Prof. Juha T. Huiskonen's expertise: Structures and host cell interactions of emerging viruses.<br><br>Website: https://www.ndm.ox.ac.uk/team/juha-huiskonen
	James F Conway<br>University of Pittsburgh<br>jxc100@pitt.edu<br>Prof. James F. Conway's expertise: Virology and cryo-electron microscopy.<br><br>Website: https://sites.pitt.edu/~jxc100/
	Pavel Plevka<br>Masaryk University: Masarykova Univerzita<br>pavel.plevka@ceitec.muni.cz<br>Prof. Pavel Plevka's expertise: Structural biology of viruses and cryo-electron microscopy.<br><br>Website: https://plevkalab.ceitec.cz/
**Opposed Reviewers:**	Z. Hong Zhou<br>University of California Los Angeles<br>Hong.Zhou@UCLA.edu<br>Conflicts of interest.
**Additional Information:**	
**Question**	**Response**
**Competing Interests**<br><br>You are responsible for recognizing and disclosing on behalf of all authors any competing interest that could be perceived to bias their work, acknowledging all financial support and any other relevant financial or non-financial competing interests.<br><br>Do any authors of this manuscript have competing interests (as described in the PLOS Policy on Declaration and Evaluation of Competing Interests)?<br><br>**If yes**, please provide details about any	The authors have declared that no competing interests exist.

and all competing interests in the box below. Your response should begin with this statement: *I have read the journal's policy and the authors of this manuscript have the following competing interests:*<br><br>**If no** authors have any competing interests to declare, please enter this statement in the box: "*The authors have declared that no competing interests exist.*"<br><br>* typeset<br><br>This statement is required for submission and **will appear in the published article** if the submission is accepted. Please make sure it is accurate and that any funding sources listed in your Funding Information later in the submission form are also declared in your Financial Disclosure statement.	
**Ethics Statement**<br><br>You must include an Ethics Statement in the Methods section of your manuscript if your study involved:<br>• Human participants<br>• Human specimens or tissue<br>• Vertebrate animals or cephalopods<br>• Vertebrate embryos or tissues<br>• Field research<br><br>**Not including a statement when required will delay the review process.**<br><br>General guidance is provided below. Please consult the guidelines on human subjects research and animal research for detailed instructions.	I confirm that I have read and understood PLOS' requirements for an Ethics Statement.

Powered by Editorial Manager® and ProduXion Manager® from Aries Systems Corporation

**Format for specific study types**<br><br>**Human Subject Research (involving human participants and/or tissue)**<br>• Give the name of the institutional review board or ethics committee that approved the study<br>• Include the approval number and/or a statement indicating approval of this research<br>• Indicate the form of consent obtained (written/oral) or the reason that consent was not obtained (e.g. the data were analyzed anonymously)<br><br>**Animal Research (involving vertebrate animals, embryos or tissues)**<br>• Provide the name of the Institutional Animal Care and Use Committee (IACUC) or other relevant ethics board that reviewed the study protocol, and indicate whether they approved this research or granted a formal waiver of ethical approval<br>• Include an approval number if one was obtained<br>• If the study involved *non-human primates*, add *additional details* about animal welfare and steps taken to ameliorate suffering<br><br>**Field Research**<br><br>Include the following details if this study involves the collection of plant, animal, or other materials from a natural setting:<br>• Field permit number<br>• Name of the institution or relevant body that granted permission<br><br>Please check the box to confirm your understanding of this policy, then read and agree to one of the statements.	
Please respond:<br>as follow-up to "**Ethics Statement**<br><br>You must include an Ethics Statement in the Methods section of your manuscript if	My study does not require an ethics statement.

your study involved:<br>• Human participants<br>• Human specimens or tissue<br>• Vertebrate animals or cephalopods<br>• Vertebrate embryos or tissues<br>• Field research<br><br>**Not including a statement when required will delay the review process.**<br><br>General guidance is provided below. Please consult the guidelines on human subjects research and animal research for detailed instructions.	

Powered by Editorial Manager® and ProduXion Manager® from Aries Systems Corporation

**Format for specific study types**<br><br>**Human Subject Research (involving human participants and/or tissue)**<br>• Give the name of the institutional review board or ethics committee that approved the study<br>• Include the approval number and/or a statement indicating approval of this research<br>• Indicate the form of consent obtained (written/oral) or the reason that consent was not obtained (e.g. the data were analyzed anonymously)<br><br>**Animal Research (involving vertebrate animals, embryos or tissues)**<br>• Provide the name of the Institutional Animal Care and Use Committee (IACUC) or other relevant ethics board that reviewed the study protocol, and indicate whether they approved this research or granted a formal waiver of ethical approval<br>• Include an approval number if one was obtained<br>• If the study involved *non-human primates*, add *additional details* about animal welfare and steps taken to ameliorate suffering<br><br>**Field Research**<br><br>Include the following details if this study involves the collection of plant, animal, or other materials from a natural setting:<br>• Field permit number<br>• Name of the institution or relevant body that granted permission<br><br>Please check the box to confirm your understanding of this policy, then read and agree to one of the statements. "	
**Data Availability**<br><br>PLOS journals require authors to make all data underlying the findings described in their manuscript fully available, without restriction and from the time of	Yes - all data are fully available without restriction

publication, with only rare exceptions to address legal and ethical concerns (see the PLOS Data Policy and FAQ for further details). When submitting a manuscript, authors must provide a Data Availability Statement that describes where the data underlying their manuscript can be found.<br><br>Your answers to the following constitute your statement about data availability and will be included with the article in the event of publication. **Please note that simply stating 'data available on request from the author' is not acceptable.** ***If, however, your data are only available upon request from the author(s), you must answer "No" to the first question below, and explain your exceptional situation in the text box provided.***<br><br>Do the authors confirm that all data underlying the findings described in their manuscript are fully available without restriction?	
Please describe where your data may be found, writing in full sentences. **Your answers should be entered into the box below and will be published in the form you provide them, if your manuscript is accepted.** If you are copying our sample text below, please ensure you replace any instances of XXX with the appropriate details.<br><br>• If your data are all contained within the paper and/or Supporting Information files, please state this in your answer below. For example, "All relevant data are within the paper and its Supporting Information files."<br>• If your data are held or will be held in a public repository, include URLs, accession numbers or DOIs. For example, "All XXX files are available from the XXX database (accession	The electron density maps and atomic coordinates have been deposited in the EM Data Bank and Protein Data Bank under accession codes EMD-36844, EMD-36845, EMD-36846, EMD-36847, EMD-36848, 8K35, 8K36, 8K37, 8K38 and 8K39.

number(s) XXX, XXX)." If this information will only be available after acceptance, please indicate this by ticking the box below.<br>• If neither of these applies but you are able to provide details of access elsewhere, with or without limitations, please do so in the box below. For example:<br><br>"Data are available from the XXX Institutional Data Access / Ethics Committee for researchers who meet the criteria for access to confidential data."<br><br>"Data are from the XXX study whose authors may be contacted at XXX."<br><br>* typeset	
Additional data availability information:	Tick here if the URLs/accession numbers/DOIs will be available only after acceptance of the manuscript for publication so that we can ensure their inclusion before publication.

Powered by Editorial Manager® and ProduXion Manager® from Aries Systems Corporation

Dear Editor Hodge,

Thank you for forwarding the reviewers' comments on our manuscript.

We appreciate the reviewers' critical review of our work and their constructive suggestions on how to improve the manuscript. We have strengthened the text and figures according to these comments and suggestions. New comparisons to the previous literatures and discussions of commonalities and divergences of the long-tailed phages have been added to the revised manuscript. The molprobity scores, r.m.s deviations, clash scores, rotamer outliers, ramachandrain plot, and EMDB/PDB IDs have been included in the new Table S4 (previous Table S3). The Q-scores are present in the PDB reports (see “supporting information” in our submission). Along with this letter, we have enclosed a point-by-point response to all of the comments and suggestions.

Please do not hesitate to contact us if you require any additional information.

Thank you for your thoughtful consideration of our work.

Sincerely,

Hongrong Liu, Ph.D.
School of Physics and Electronics,
Hunan Normal University
36 Lushan Road, Changsha, Hunan, China
E-mail: hrliu@hunnu.edu.cn

Lingpeng Cheng, Ph.D.
School of Physics and Electronics,
Hunan Normal University
36 Lushan Road, Changsha, Hunan, China
E-mail: lingpengcheng@hunnu.edu.cn

We would like to thank the reviewers for their critical review of our work and for their constructive suggestions on how to improve the manuscript. The comments and suggestions have been addressed, and the manuscript has been revised accordingly. To make the responses easy to follow, we have copied the original comments in black, and marked our responses in blue. The page and line numbers correspond to those in the revised article with changes highlighted.

Reviewer #1:

The manuscript "In situ structure of the connector and tail of a Siphophage" by Hao Xiao et al describes the structure of the phage lambda connector-tail complex in the mature virion by cryo-electron microscopy. The composite structure was assembled from the capsid with icosahedral symmetry imposed, the portal-connecter region with C12 symmetry, the tail tube using C6 symmetry, and the tail tip with C3 symmetry. This is a sensible strategy as the flexibility of the tail tube precludes solving phage particles in toto, while the head and tail-tip that bracket the tail tube are largely rigid. The structural data presented appear to be quite comprehensive with most of the known protein components partially or fully resolved, except where there is excess flexibility such as the very tail tip and much of the tape-measure protein. The quality of density appears to be sufficient for the atomic models generated although only small regions of fitting can be shown in Figures. As the density maps are not available to the reviewer, grey-scale sections through the structures would be useful for assessing noise, artefacts and overall quality - the only section is in Fig S7a through the tail tube - and (having been burnt before by papers from other groups) I insist on seeing such data before giving a recommendation for publication. In addition, a section through the putative iron atom (lines 247-250) should show significant density due to the high scattering power of iron, and would be an addition to Figure 4F. Nonetheless, this looks overall like a structural tour-de-force, especially for detailing the complex portal-connection organization and especially the tail-tip. What it lacks is a functional payoff from this new structural data, and while something of this sort is attempted in the Supplemental Material, it

is inadequate and a better attempt should be added at the end of the main text. If space is a consideration, I suggest shortening the structural details by focusing on the more significant findings in order to allow several paragraphs of conclusions. This is not a recommendation for new experimental data but for significant improvements in the presentation.

We present four gray-scale sections of the structures as follows. Panels A and B are sections of the tail tip. The features in the red circles are iron-sulfur clusters. Panel C is a section of the portal and rings of the adaptor, stopper, and tail

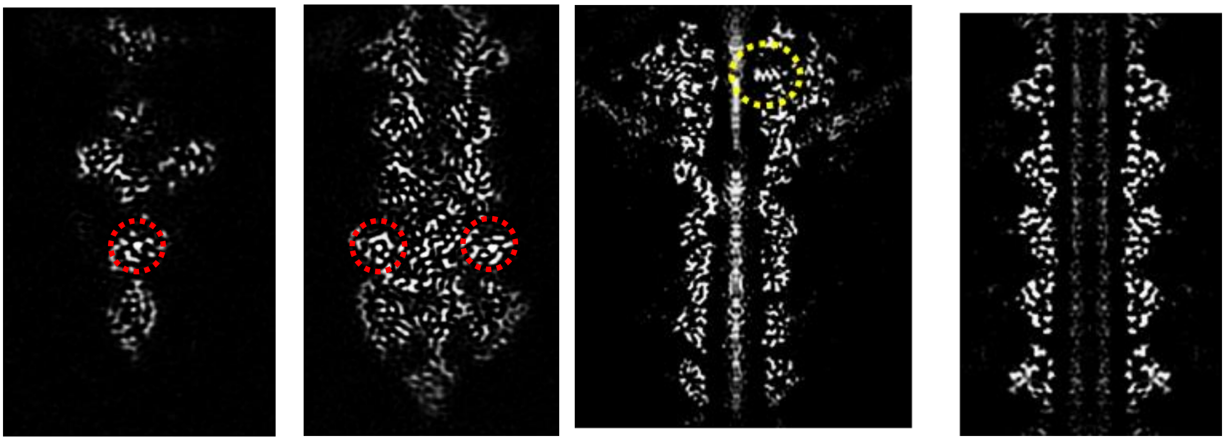

terminator. The feature in the yellow circle is an α-helix. Panel D is a section of tail tube. We have added the central section of the iron-binding site to the new Fig. 7F.

We have enhanced the manuscript by adding structural comparisons between the tail tips of lambda and other long-tail phages or tail-like machines. Our results suggest that their tail tip contains conserved domains that facilitate tail assembly, receptor binding, cell adsorption, and DNA retaining/releasing. It is intriguing that these domains are distributed in different tail tip proteins in different phages or tail-like machines (see the section "Structure of the tail tip complex" in the revised manuscript for details).

The structural similarity between the tail tips of lambda and T5 (Linares et al. *Structural basis of bacteriophage T5 infection trigger and E. coli cell wall perforation*. Science Advances. 2023;9) suggests that upon the receptor interaction, the tail tip of lambda would undergo structural changes similar to that of T5. The tail tips of

lambda and T5 share common domains of HDI–IV, HDII-insertion, plug, and two fibronectins (FNIIIs). For T5, all these domains are located in the baseplate hub protein pb3. By contrast, for lambda, the HDI is located in gpL; the HDII, HDIII, HDIV, two FNIIIs, and the C-terminal region of HDII-insertion are located in gpJ; the N-terminal region of HDII-insertion is located in gpL; and the plug is located in gpI. It is noteworthy that gene I is located between genes L and J, a gene location that corresponds to the position of the T5 pb3 plug domain, except for an inserted gene K (see the new Fig. S9). Therefore, we suggest that gpL, gpI, and the N-terminal region of gpJ (from HDII to FNIII-1 and -2) together correspond to T5 BHP pb3. In addition, the central fiber protein (pb4) and receptor binding protein (pb5) of T5 correspond to the central fiber domain and receptor binding domain of lambda gpJ, respectively.

Specific concerns and considerations:

1. The term "connecter" is used to describe the portal gp8, "adapter" gpW, and "stopper" gpFII, but this is not correct. The portal is separate from the connector, being part of the capsid and involved in DNA packaging, while the connecter is a region that joins the portal to the tail after packaging is complete. Thus, it would be more correct to describe the major components as the capsid, portal, connector, tail tube and tail-tip, and in particular, the parts presented here are the portal, connector and tail (implicitly including the tail tube and tail-tip). This should be reflected in the title and throughout the manuscript. For example, line 77:

The portal combines two rings of adaptor and stopper proteins to form the head-to-tail connector

This does not properly recognize the portal as a separate and critical component that functions not only in packaging and releasing DNA, but likely nucleates capsid assembly.

We thank the reviewer for pointing this out. Reviewer 2 also pointed out the same problem and suggested that we use “neck (connector)” to describe the adaptor and stopper. We have revised the manuscript accordingly.

2. Side-tail fibers are mentioned in passing (line 78, 229), but should be visible in the tail-tip structure, even as truncated knobs that most lambda lab strains include. Four side-tail fibers densities should be observed bound to gpL but none are noted. This is especially interesting in the context of the model that includes 4 trimers of the hub protein, gpL, which accounts for an apparent symmetry mismatch between a hexameric tail/trimeric tip to which the 4 side tail fibers bind.

We thank the reviewer for pointing out the absence of the side tail fiber in our structure. Reviewer 2 also raised the same question. The lambda phage we used is a laboratory strain and is called "lambda wild type" (Hendrix RW, Duda RL. Bacteriophage lambda PaPa: not the mother of all lambda phages. Science. 1992;258:1145-8). It lacks side tail fiber proteins (stf and tfa) as a result of a laboratory-acquired mutation. The lack of the side fiber proteins was further confirmed by our recent mass spectrometry experiment (page 6, lines 139-149; page 17, lines 473-478).

We have revised our manuscript accordingly.

3. Line 96: Our structure of the head is essentially identical to that reported by a recent study on the lambda head [32] except that our structure has higher resolution. This difference - 3.8A vs 3.5A - is really not worthy of comment. I suggest removing "except that our structure has higher resolution" and instead point out any significant improvements with biological meaning, if any (none are described).

We have deleted the line "except that our structure has higher resolution." Both icosahedral structures of the lambda head are essentially identical (see the figures below).

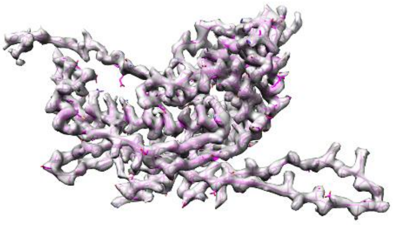

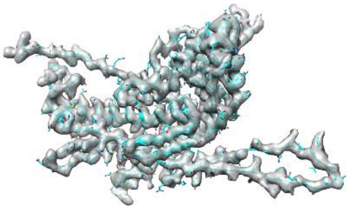

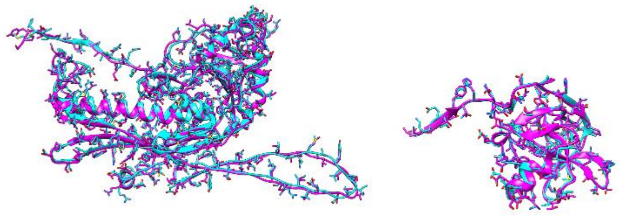

Top left: superimposition of our model of gpE on our density map (3.5Å resolution). Right: superimposition of model of gpE (7vik) by Wang et al. on their density map (by using local refinement, 3.7Å resolution, EMD-32011).

Bottom Left: superimposition of our gpE model (magenta) with the model by Wang et al. (cyan, PDB ID: 7vik). Bottom right: superimposition of our gpD model (magenta) with the model by Wang et al. (cyan, PDB ID: 7vik).

4. While this work is an advance on previous capsid and tail tube structures, and cites them, it doesn't provide any context about how the new structures differ from them, and in particular, what new functional insights are provided. More attention is given to comparison with existing NMR structures of monomers, or domains, which are of less relevance being unassembled in solution.

We have added the structural comparison between the tail tip complexes of lambda and other long-tail phages (including T5) or tail-like machines (see the section "Structure of the tail tip complex" (page 10) in the revised manuscript for details.

5. The argument (lines 208-214) that the dsDNA extends down the tail to the 3-4 gpV rings, where it meets the gpH tape measure polymer, is based on the internal tube density transitioning from hollow (gpH) to solid (dsDNA). However, density along a symmetry axis (the tail tube) is notoriously noisy, and one wonders if this is in fact where the portal-connect structure is joined to the separately-calculated tail tube structure. Additional evidence is needed to support this density assignment. Further, the tape-measure protein defines the length of the tail tube - how can it do this if it ends short of the tail tube itself? What then is governing the tail tube length? The other ideas raised, that the gpH-dsDNA interaction favors delivery and allows a

release signal to propagate from the tail tip through the tape-measure protein, do not require DNA to spill into the tail tube to make this interaction if the gpH is as long as the tail tube. This paragraph should be re-thought or omitted.

We agree with the reviewer and have deleted this part (page 10, lines 260-274).

6. Use of "Megatron" for the gpJ protein is new to me - I don't think this is in common use. The source seems to be ref [37] from 2020 which appropriated it from a character in the Transformers franchise, but the wording in this 2020 paper is unclear as to whether "Megatron" refers to one large multidomain protein, or several. Thus, I am also confused by line 242 that refers to Megatron in the context of gpL, which is not the "Megatron protein (gpJ)" labeled on Figure 1c. In addition, there is a completely different phage with this name - Mycobacterium phage Megatron - so I suggest "Megatron" should be dropped to avoid contributing further confusion.

We have renamed the gpJ as “central fiber protein” according to the nomenclature of lambda gpJ in a review (Davidson AR et al., 2012. Long Noncontractile Tail Machines of Bacteriophages. Adv Exp Med Biol 726).

We have revised the manuscript and figures accordingly.

7. There is an attempt at functional insights that derive from the new structure in the Supplementary information. In principle, this should be the conclusions of the main text so it's odd to read it here. However, its focus is on assembly when the paper has determined only the end product of assembly and has little to say about the steps to accomplish this. Thus, the assembly itself is inferred from other work, and much is speculative, vague and not novel. The order of accreting subunits isn't demonstrated beyond what is obvious - the head and tail assemble independently, packaging precedes binding of the head completion proteins (connector), and the tail binds to the head.

We have deleted the supplementary information.

8. General comments.

We thank the reviewer for helping us improve our manuscript.

Grammar could be improved in numerous places and some awkward phrasing re-thought - the authors should pay careful attention to what they write. Frequent repetition can also be avoided - for example, this redundancy in line 41:

During the packaging of the genome, double-stranded DNA (dsDNA) is packaged into the head…

We have deleted it (page 3, line 53).

And an example of mis-wording, on line 75 - the vertex is not replaced, nothing is replaced, instead one vertex is *occupied* by the portal:

One vertex of the icosahedron is replaced by a dodecameric portal,…

We have revised it (page 4, lines 95-96).

This makes no sense, although I understand the intent (line 240):

Three copies of gpL and three copies of gpJ, surrounded alternately forming the core of the trimeric conical tail tip.

We have deleted it (page 11, lines 295-296).

Reviewer #2:

The MS "In situ structure of the connector and tail of a Siphophage" describes the atomic structure of bacteriophage lambda, namely the "wild type" lambda or the fiberless laboratory strain, as determined by cryo-electron microscopy. The authors report the composition of the virion, the copy number of most component proteins, and the nature of interaction between some components of the virus particle.

Despite containing a wealth of atomic-level information and contrary to the authors' statement (the last sequence of the Abstract), the MS provides little insight into the infection mechanism or assembly of the lambda particle for several reasons. Infection mechanism.

"Wild type" (fiberless) lambda's infection process is well characterized functionally as lambda is a "model" phage for host receptor-induced DNA release. However, several processes remain poorly understood: a) how the interaction of the tail tip with the host LamB receptor opens the tube channel; b) structural transformation of the neck upon the initiation of DNA release; c) the function of side fibers and their role in the opening of the tube channel. Neither of these is studied in this paper.

We agree with the reviewer that we were unable to determine how the interaction of the tail tip with the host LamB receptor opens the tube channel and structural transformation of the neck upon the initiation of DNA release.

We have enhanced the manuscript by adding structural comparisons between the tail tips of lambda and other long-tail phages or tail-like machines. The structural similarity between the tail tips of lambda and T5 (Linares et al. *Structural basis of bacteriophage T5 infection trigger and E. coli cell wall perforation*. Science Advances. 2023;9) suggests that upon the receptor interaction, the tail tip of lambda would undergo structural changes similar to that of T5. The tail tips of lambda and T5 share the common domains of HDI–IV, HDII-insertion, plug, and two fibronectins (FNIIIs). For T5, all these domains are located in baseplate hub protein pb3. By contrast, for lambda, the HDI is located in gpL; the HDII, HDIII, HDIV, two FNIIIs, and the C-terminal region of HDII-insertion are located in gpJ; the N-terminal region of HDII-insertion is located in gpL; and the plug is located in gpI. In addition, the central fiber protein (pb4) and receptor binding protein (pb5) of T5 correspond to the central fiber domain and receptor binding domain of lambda gpJ, respectively.

Further structural comparisons between the tail tips of lambda and other long-tail phages or tail-like machines suggest that their tail tip contains conserved domains that facilitate tail assembly, receptor binding, cell adsorption, and DNA retaining/releasing. It is intriguing that these domains are distributed in different tail tip proteins for different phages or tail-like machines (see the section “Structure of the tail tip complex” in the revised manuscript for details).

In regard to the side tail fibers, our recent electron microscopy analysis of ultrathin sections of the lambda-infected E. coli cells showed that the lambda wild-

type particles were adsorbed perpendicular to the cell surface, although these particles lacked side tail fibers, suggesting that perpendicular adsorption did not rely on the participation of the side tail fibers (page 15, lines 418-426).

2. Assembly (and structure).

Unlike the laboratory-adapted and confusingly called "wild type" lambda, which lacks side fibers as a result of a laboratory-acquired mutation, the tail of the "real wild type" lambda carries four side fibers. These four side fibers are somehow attached to the sixfold (or threefold?) symmetric tail. How? The mystery of four side fibers remains unsolved.

We thank the reviewer for pointing out the absence of the side tail fiber in our structure. Reviewer 1 raised the same question. The lambda phage we used is a laboratory strain and is called “lambda wild type” (Hendrix RW, Duda RL. Bacteriophage lambda PaPa: not the mother of all lambda phages. Science. 1992;258:1145-8). It lacks side tail fiber proteins (stf and tfa) as a result of a laboratory-acquired mutation. The lack of the side fiber proteins was further confirmed by our recent mass spectrometry experiment (See Table S2).

We have revised the manuscript accordingly (page 4, line 102).

The second big question is the interaction of the DNA with the tape measure protein in the tube channel. In a mature lambda particle, one end of DNA molecule is thought to extend into the tube channel from the capsid. The authors assume it is the case here and they distinguish between the DNA and gpH densities in the tube channel by the presence of a lumen in the latter (in gpH). Such interpretation lacks rigor. Has the length of the DNA fragment, which extends into the channel, been determined experimentally earlier? How does that length correlate with the interpretation of the cryoEM map reported here? One way to distinguish DNA from protein is to use "bubblegram" imaging: https://www.science.org/doi/10.1126/science.1214120 Perhaps, something like that can be attempted here?

We thank the reviewer for pointing out this question. Reviewer 1 and reviewer 3 also raised the same question. We tried the "bubblegram" experiment but did not observe bubbles in the tail tube (see the figures below). Therefore, we have deleted this part (page 10, lines 260-274).

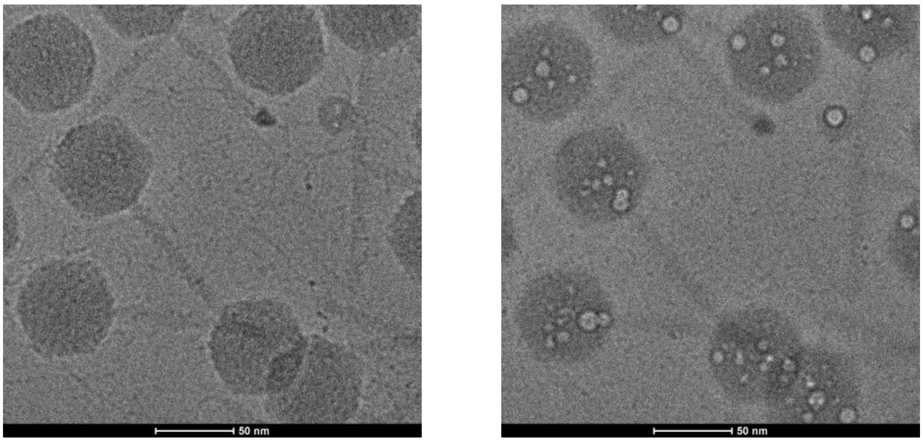

Cryo-EM images of the lambda particles with a electron dose of 60 e^-/Å^2 (left) and 120 e^-/Å^2 (right).

As a final note on assembly, I would like to point out that one structure, no matter the resolution, cannot explain the process of assembly. And yet the Supplementary Information supplied with this paper does just that - is displays a schematic of particle assembly. It even shows four side fibers that weren't in the particle studied here. The process of lambda particle assembly has been delineated in many publications over the years. Various intermediates have been isolated and characterized. This study contains no new experiments that describe such complexes.

We agree with the reviewer and have deleted the Supplementary Information and Fig. S11.

Besides the major concerns about the presentation of data and extrapolation of those results to subjects that have not been studied in this paper (as outlined

above), there are many minor concerns that can be fixed by editing and rewriting. Of those, I will highlight only a few significant ones.

First, the title is confusing. The authors describe the structure of the complete "WT" lambda that is free in solution. Nothing is in situ here. N.B. that a phage particle consists of a head (or capsid, not capsid shell, line 39), a tail, a neck, and tail fibers (or, more generally, receptor-binding proteins). The tail is connected to the head not by a tail-to-head connector, but by a neck. Two neck proteins that interact with the portal - gpW and gpFII - are, in fact, head proteins because they do not bind to isolated tails. They are head maturation proteins. So, these proteins cannot be part of the "in situ" tail, they are neck proteins.

We thank the reviewer for pointing this out. Reviewer 1 also pointed out the same problem and suggested we use “connector” to describe the adaptor and stopper. We have used the term “neck (connector)” to describe the adaptor and stopper in the revised manuscript. We have changed the title to “[illegible]nmetry-mismatch structure of the bacteriophage lambda.”

Second, the Introduction is a hodgepodge of sentences and concepts taken from different reviews and other papers. There is no flow and everything is just mixed together. Bacteriophage abundance (which is irrelevant for this paper) is immediately followed by a description of a particle structure with the words "pentameric", "icosahedral", "portal", "vertex", which are highly specialized terms only structure-versed people understand (and not all even). Then the DNA packaging is described in one sentence. Then, we learn about some mysterious conformation of the portal. Nothing about tail assembly though. Then we learn about different tail morphologies (shouldn't we have started with this?). And then, we learn that the tail is important for host recognition and attachment. Some general concepts and highly specialized terms are put together into unreadable sentences.

We have revised this paragraph (page 3).

Third, the Results and Discussion section is all about minute details how this residue interacts with that residue. This is irrelevant for our understanding of particle assembly or infection mechanism. These sort of descriptions should be published in a journal that specializes on protein structure. I strongly suggest removing all the minutia into the supplementary information and compare global properties of lambda tail proteins with those of other phages. By global properties I mean surface properties, hydrophobicity, surface charge distribution, etc. Things that are actually important for function.

We have deleted the minute details on how this residue interacts with that residue. We have moved Figs. S6 and S7 to the main text (the new Figs. 4 and 6).

Two points regarding terminology.

1. Beta-strand chain swapping/insertion is called beta-sheet augmentation, not beta-augmentation. See here: https://pubmed.ncbi.nlm.nih.gov/16828554/

We have revised them (page 7, line 184; page 8, line 209; page 13, line 355; legends of the new Figures 3, 5, and 7).

2. Near-atomic resolution describes a dataset in which at least some of the atoms are resolved. That is - the resolution limit must be higher that the length of at least some of the bonds in the studied molecule. For proteins, that would be ~1.4A. Resolutions lower than 1.4A are not near-atomic. The maps presented in this paper are great looking and appear to be easily interpretable in terms of amino acids, NOT atoms, so these maps a really far from "near-atomic". The highest resolution reported here is 3.2 A. This map contains only 8.3% (!) of the information expected in a near-atomic resolution map, which would be at 1.4 A resolution. All other maps are even further away from being "near atomic".

We have deleted all descriptions of “near-atomic resolution” in the manuscript.

One big positive features of the MS is that the figures are done very well. Very good amount of labels, good placement and clarity. This is much appreciated. However,

some of the more important figures are relegated to the Supplementary Information section at the expense of showing electron density of side chains and minutia of interactions that are functionally irrelevant. I think Fig. S3 should be in the main text. Fig. S5 is a good candidate for a main text figure as well. But in addition to the folds, the surfaces must be shown and their properties characterized. It's the surface that performs the function, not the spaghetti of the protein fold. Fig. S6 must be a main text figure. This is the only figure that deals with assembly as it shows how these rings are distinguished based on their electrostatic properties. Fig. S7 must be in the main text. I would vote for Fig. S9 to be in the main text as well. Fig. S11 should be published in a separate review paper.

We have moved Figs. S3, S6, S7, and S9 to the main text and have deleted Fig. S11.

Reviewer #3:

The manuscript entitled "In situ structure of the connector and tail of a Siphophage" by Xiao and co-authors has been submitted to the PLOS Biology journal. The authors present the structure of the Siphophage Lambda that infects the bacterial species Escherichia coli (E. coli). The structure has been obtained at resolutions varied from ~3.5 to 6 Angstroms between different components of the phage complex; a structure of the nearly complete phage was solved at 20 Angstrom resolution. The phage Lambda has been used for nearly two decades as a test object for usage verification methods of the cryo-electron microscopy, and software development. The authors have achieved some progress in the revelation of structural details allowing to trace the polypeptide chains of several phage proteins. This study provides an additional information on structural details of the Lambda phage and suggests some new ideas related to the phage structural organisation, that might be useful at analysis of other bacteriophages.

However, some issues must be addressed and comparison with the published structures should be done before the publication of this work:

The authors submitted the manuscript with rather unclear title: "In situ structure of the connector and tail of a Siphophage". It is easy to assume that the authors want their manuscript to be cited; in this case they must specify in the title which phage they have analysed. There are so many Siphoviridae phages, so readers will be interested in what was specifically new in this study.

We have changed the title to “Symmetry-mismatch structure of the bacteriophage lambda.”

The authors did not discuss or indicated why this phage was important and should be subjected for this research, since as a test sample this phage was used for quite long time. There are some problems with logistic of the manuscript which is rather inconsistent. In the abstract they move from the tail to the connector-tail complex and its symmetry, then again to the tail. The structure of the head was not mentioned. The mechanism of infection was mentioned, but it was not explained and properly discussed in the manuscript. The main part of the introduction is dedicated to the classification of phages: at the beginning the authors started from the phage head and moved their discussion towards the tails tip, but still it was not explained why it was important to study Siphoviridae phages and, specifically, the Lambda phage.

We have added the importance of studying the lambda phage: “The lambda phage is used as a model system and is important in the attainment of the current understanding of the sophisticated and complex mechanisms by which the expression of genesis is controlled, the understanding of DNA virus assembly, and the understanding of the molecular nature of lysogeny” (page 4, lines 86-90).

We have revised the abstract and introduction.

Results and discussion start from the head and overall organisation of the phage, but then the authors jumped to the connector, tail, tip, and details of structural organisation of the head were not provided. Apparently, a resolution of it was rather low and not comparable with the results published by G. Lander and J. Johnson in

2008 and by Wang C, Zeng J, Wang J. in 2022 (Structural basis of bacteriophage lambda capsid maturation. Structure, 2022 Apr;30(4):637-645.e3. DOI: 10.1016/j.str.2021.12.009. Resolution: 5.03).

We did not provide the details of the structural organization of the head because the high-resolution structure of the lambda head was previously published (Wang et al., *Structural basis of bacteriophage lambda capsid maturation*. Structure. 2022;30). Our models of gpD and gpE, which were built based on our head density map at a resolution of 3.5 Å (See Fig. S2B), are essentially identical to those reported by Wang et al (see the figures below). Our models of gpD and gpE are essentially identical to the subnanometer resolution structure reported by G. Lander et al. (see the figures below).

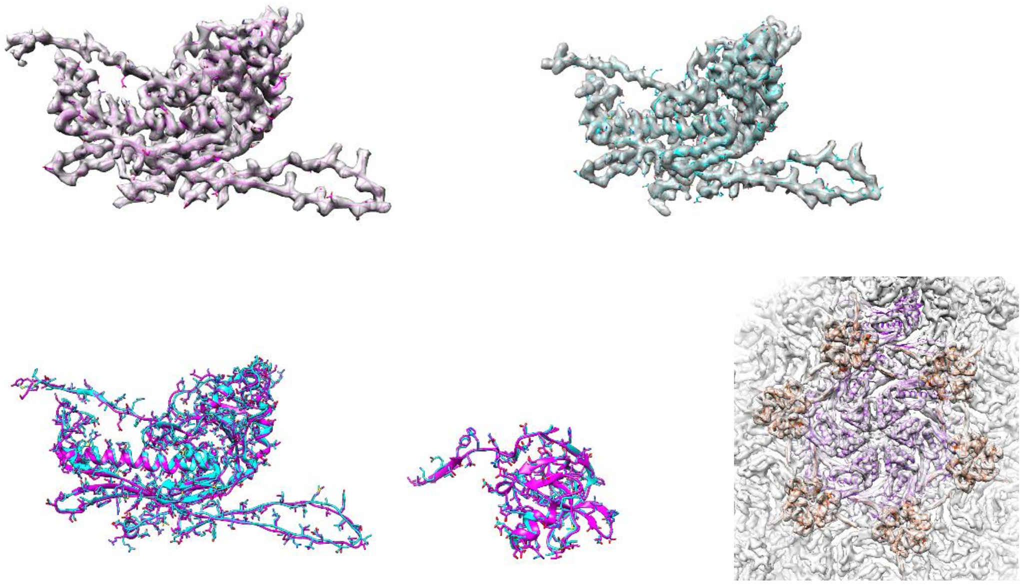

Top left: superimposition of our model of gpE on our density map (3.5Å resolution). Right: superimposition of model of gpE (7vik) by Wang et al. on their density map (by using local refinement, 3.7Å resolution, EMD-32011).

Bottom Left: superimposition of our gpE model (magenta) with the model by Wang et al. (cyan, PDB ID: 7vik). Middle: superimposition of our gpD model (magenta) with the model by Wang et al. (cyan, PDB ID: 7vik). Right: superimposition of our models of gpD (orange) and gpE (purple) on the density map by G. Lander et al.

The structure of the Lambda tail at 2.7 A resolution has been reported and deposited to the EMBD database in 2022 (EMD-25611, Prokhorov NS, Yang Q, Catalano CE, Morais MC). No comparison was done with previously reported results.

The model of gpV based on our tail tube density map at a resolution of 3.6 Å is very similar to the model based on the density map at a resolution of 2.7 Å (EMD-25611, see the Figures below).

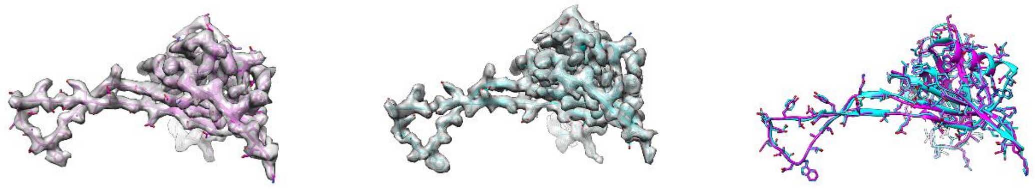

Left: superimposition of our model of gpV on our density map. Middle: superimposition of the model of gpV By Prokhorov et al. (PDB ID: 7t2e) on their density map (EMD-25611). Right: superimposition of our model (magenta) with their model (cyan, PDB ID: 7t2e).

Unfortunately, the methods are not complete: the authors wrote that they have used their own software packages. No information was provided if these packages are available for other users, where are the links to these packages, which parameters were used during structural analysis, how high was percentage of used data in the last reconstructions, where are the atomic models used in the fitting come from. The quality of the structures reported suggests that the authors were not able to trace de novo polypeptide chains, or the accession codes must be provided if the authors used some homologues structures.

The software package used for symmetry-mismatch reconstruction (including the tutorial and test images) can be downloaded from https://cryoem.hunnu.edu.cn. We have revised the manuscript accordingly (page 5, lines 125-126).

The percentages of the particle images used in the final reconstructions ranged from 66% to 80% (see the new Table S4).

We did not use homologous structures. Based on our density maps, we built de novo atomic models for the head proteins (gpE and gpD), the portal and neck

(including two 12-fold rings of the portal protein gpB and the adaptor protein gpW, and a 6-fold ring of the stopper protein gpFII), the tail tube (including a 6-fold ring of the tail terminal protein gpU and thirty-two 6-fold rings of the tail tube protein gpV), and the tail tip (including a 6-fold ring of the distal tail protein gpM, 3-fold trimers of the hub protein gpL, insertion protein gpI, central fiber protein gpJ, and a C-terminal α-helix of the tape measure protein gpH) (see the new Table S4).

What were the RMSDs between different portal proteins when the authors have done alignments: Figure S5, second raw. Where was the alignment done and according to which features? The authors have used for the SPP1 the atomic model of the 13-mer, why the authors did not use the 12-mer? The latest structure is of better quality, see 7Z4W (gp612 - gp1512 - gp166 complex).

We aligned the portal proteins using the MatchMaker of UCSF Chimera. The RMSDs between the portal proteins of lambda and SPP1, T7, and T4 are 2.52, 2.48, and 2.03 Å, respectively. We have replaced the SPP1 atomic model of the 13-mer with 12-mer (7Z4W) in Fig. S5 (now is the new S4 Fig), and revised the figure legend accordingly.

It is interesting that the authors suggested that the tape measure protein and DNA are interwinding within the central channel of the tail. One can wonder on which evidences this suggestion was made. Do the authors have any biochemical evidence that the first pieces of the ejected DNA are intertwined with the tape measure protein? Does the tape measure protein is injected into the host cells? Did the authors measured the diameter of the inner tube of the tails? it seems that it is significantly smaller if one will compare the diameter of the dsDNA complexed with the tape measure proteins … Would be essential to provide assessments of these values.

We have deleted the suggestion for the type measure protein gpH (page 10, lines 260-274).

The authors have to provide the standard table where the overall information on the data collection will be given, the quality of the fittings, how the models were obtained, values of the correspondence between maps and polypeptide chains, RMSD, Molprobity scores, and results from the Ramachandran plots (for all fitted proteins).

We have provided the information in the new Table S4.

The part of "the Cryo-EM and data collection" is very confusing, it seems that the authors are not familiar neither with the equipment they have used nor with the requirements for the publications. Typically, Quantifoil R2/1 copper grids (line 315) have the 2-nm thick continues carbon film, not 5-nm. If the authors have used 5 nm thick carbon layer, that have to be explained. and the proper number for this type of grids should be provided (see this and the other websites https://www.jenabioscience.com/about-us/news-blog/3342-cryo-em-grids-available).

We thank the reviewer for their critical review of our Materials and Methods.

The thickness of the Quantifoil Holey Carbon Film we used was about 20 nm (https://www.tedpella.com/calibration_html/QUANTIFOIL_TEM_Substrates.aspx). Our initial cryo-EM results showed that a small number of lambda particles entered the holes in the grid. To let the phage particles enter the holes, we covered the Quantifoil grids with a 5-nm-thick carbon film using a turbomolecular pumped coater (Q150TE, Quorum, UK).

We have revised the description and provided the information of the mesh number of the quantifoil grids (page 17, lines 480-484).

They have used unusual terminology "double-blotting". That must be explained what the authors have meant.

We have changed the term “double-blotting” to “After blotting the excess sample solution on the grid two times” (page 17, line 485).

The information on GIF is not complete. The microscope was equipped with a Gatan Quantum energy filter. What was the slit used at the data collection? What was the voltage in the microscope? What was a defocus range used at the data collection? The authors have to check how the magnification was consistent with the pixel size information. What was written in the MS is not consistent and does not correspond to the information given on websites of electron microscopes. Was the pixel size calibrated? If it was done, that has to be explained.

The Gatan Quantum energy filter with a slit width of 20 eV was used to remove inelastically scattered electrons, the electron microscope was operated at a voltage of 300 kV, and the defocus range was from 1.6 to 2.2 µm. The K3 detector was operated in the super-resolution mode, and automatic data collection was performed using the FEI EPU software with a magnification of 53,000 (physical pixel size: 1.36 Å) in the "super-resolution" mode, which resulted in a pixel size of 0.68 Å. The pixel size was calibrated using 500-nm diffraction grating replica and latex calibration standard.

We have revised the manuscript accordingly (page 17, lines 488-495).

Image processing: what was a size of boxes for the processing of the complete phage images? Sizes pf the boxes should be given in pixels (not in the angstroms) with indication of the pixel sizes. It seems that at analysis of the different elements of the phage the authors have used different pixel sizes and box sizes.

The box size for the images of the complete phage is 900×900 pixels (resampled to a pixel size of 5.44 Å). The box sizes for the tail tube images and tail tip images are 220×220 pixels. The box size for the neck images are 400×400 pixels.

We have revised the manuscript accordingly (page 18, lines 503-505; page 18, line 525; page 19, line 532; page 19, line 535).

What was special in analysis of the symmetry mismatch: the authors were "using our symmetry-mismatch reconstruction method". No details were provided on specific features of this approach.

The software package for symmetry-mismatch reconstruction (including the tutorial and test images) can be downloaded from https://cryoem.hunnu.edu.cn. We have revised the manuscript accordingly (page 5, lines 125-126).

The authors did not provide any details on what has been found. What kind interactions were revealed between proteins at pseudo equivalent positions? The authors did not explain how the portal proteins interact with the capsid.

We added the description of the asymmetric interactions between the portal and capsid (pages 6 and 7, lines 162-168), which is similar to those observed in structures of phages T4 and sf6 (Fang Q. et al. *Structural morphing in a symmetry-mismatched viral vertex*. Nat Commun. 2020,11:1713; Li F. et al. *High-resolution cryo-EM structure of the Shigella virus Sf6 genome delivery tail machine*. Sci Adv. 2022,8:eadc9641).

We also added the description of the pseudo 6-fold symmetrical interaction between the gpM ring and gpJ-gpL complex in the tail tip (page 11, lines 290-294). The hexameric gpM ring exihibits a pseudo 6-fold symmetry in order to adapt the interaction with the 3-fold symmetrical gpJ-gpL complex.

In general, the authors did not provide any conclusion related to their research. What have they found?

We have enhance the manuscript by adding structural comparisons between the tail tips of lambda and other long-tail phages or tail-like machines. Our results suggest that their tail tip contains conserved domains that facilitate tail assembly, receptor binding, cell adsorption, and DNA retaining/releasing. It is intriguing that these domains are distributed in different tail tip proteins in different phages or tail-like machines (see the section “Structure of the tail tip complex” in the revised manuscript for details).

The structural similarity between the tail tips of lambda and T5 (Linares et al. *Structural basis of bacteriophage T5 infection trigger and E. coli cell wall perforation*. Science Advances. 2023;9) suggests that the upon the receptor interaction, the tail

tip of lambda would undergo similar structural changes upon the receptor interaction similar to that of T5. The tail tips of lambda and T5 share common domains of HDI–IV, HDII-insertion, plug, and two fibronectins (FNIIIs). For T5, all these domains are located in the baseplate hub protein pb3. By contrast, for lambda, the HDI is located in gpL; the HDII, HDIII, HDIV, two FNIIIs, and the C-terminal region of HDII-insertion are located in gpJ; the N-terminal region of HDII-insertion is located in gpL; and the plug is located in gpI. It is noteworthy that gene I is located between genes L and J, a gene location that corresponds to the position of the T5 pb3 plug domain, except for an inserted gene K (see the new Fig. S9). Therefore, we suggest that gpL, gpI, and the N-terminal region of gpJ (from HDII to FNIII-1 and -2) together correspond to T5 BHP pb3. In addition, the central fiber protein (pb4) and receptor binding protein (pb5) of T5 correspond to the central fiber domain and receptor binding domain of lambda gpJ, respectively.

In addition, our recent electron microscopy analysis of ultrathin sections of the lambda infected E. coli cells showed that the lambda wild type particles were adsorbed perpendicular to the cell surface, although these particles lacked side tail fibers, suggesting that perpendicular adsorption did not rely on the participation of the side tail fibers (S11 Fig, page 15 lines 418--426).

The authors did not provide reports from the EMDB database, where the quality of the structures and fitting of the atomic models would be given. This information is essential for the result quality assessments.

We have uploaded the structures to PDB and EMDB. The reports from the EMDB database are included in the supporting information with the manuscript submitted.

Minor comments:

The authors should show FSC curves for all reconstructions and indicate what was a resolution at 0.5 and at 0.143 levels.

We have indicated the resolutions at 0.5 and at 0.143 levels in the FSC curves (see the new Fig. S1B).

Please rephrase: "The portal is surrounded by 12 copies of gpB, which exhibits the canonical portal fold of phages …". The portal is not "surrounded"… it is composed by 12 copies of the protein …

We have revised it (page 6, lines 154-155).

The authors did not provide the info on how symmetries of the different parts of the tail were verified.

The symmetries of the different parts of the tail were verified by the high resolution of the structures we obtained. The high resolution structure could not have been obtained if the symmetry was incorrectly applied during the reconstruction.

Portal proteins do not have a "funnel" shape.

We have deleted it (page 6, line 151).

Lines 209-215. "…the rod-like structure below the third gpV ring was hollow..." The authors have to indicate how big were the changes in the inner diameter of the tale tube, how big were changes in the densities of the tail rings and how much their diameters were changes? The sentence on lines 211-213 is not justified, since not information was provided.

We agree with the reviewer. Reviewer 1 and reviewer 2 also raised the same question. We have deleted this part (page 10, lines 260-274).

Lines 219-222, The statement is not supported by any biochemical information.

We agree with the reviewer. Reviewer 1 and reviewer 2 also raised the same question. We have deleted this statement (page 10, lines 260-274).

The authors write that "all data are fully available without restriction ". The data are not, available, no information on deposition was provided, no reports on the structure qualities and fittings.

We have uploaded the structures to PDB and EMDB. The reports from the EMDB database are included in the supporting information with the manuscript submitted (see Table S4).

Line 293. A part of the sentence is confusing, should be rephrased: "...formed by a cascade of gpH trimer..." . Here there is a linguistical problem: of which "cascade" are the authors talking? Is it a process, or a set of structural elements?

We have deleted “a cascade of”.

The English has to be checked.

We have checked the English of the manuscript again.

Manuscript Click here to access/download;Manuscript;Manuscript.docx

Symmetry-mismatch structure of the bacteriophage lambda

Hao Xiao[1,2,#], Le Tan[1,#], Zhixue Tan[1,#], Yewei Zhang[1], Wenyuan Chen[1], Xiaowu Li[3], Jingdong Song[2,*], Lingpeng Cheng[1,*], and Hongrong Liu[1,*]

[1] Institute of Interdisciplinary Studies, Key Laboratory for Matter Microstructure and Function of Hunan Province, Key Laboratory of Low-dimensional Quantum Structures and Quantum Control, School of Physics and Electronics, Hunan Normal University, Changsha, China.

[2] National Key Laboratory of Intelligent Tracking and Forecasting for Infectious Diseases, National Institute for Viral Disease Control and Prevention, Chinese Center for Disease Control and Prevention, Beijing, China.

[3] School of Electronics and Information Engineering, Hunan University of Science and Engineering, Yongzhou, China.

# These authors contributed equally to this work.

*Correspondence to: songjd@ivdc.chinacdc.cn, lingpengcheng@hunnu.edu.cn, and hrliu@hunnu.edu.cn

Data Availability Statement: The electron density maps and atomic coordinates have been deposited in the EM Data Bank and Protein Data Bank under accession codes EMD-36844, EMD-36845, EMD-36846, EMD-36847, EMD-36848, 8K35, 8K36, 8K37, 8K38 and 8K39.

Competing interests: The authors declare no conflict of interest.

Abstract

Siphophages have a long, flexible, and noncontractile tail that connects to the capsid through a neck. The phage tail is essential for host cell recognition and virus–host cell interactions; moreover, it serves as a channel for genome delivery during infection. However, the in situ high-resolution structure of the neck-tail complex of siphophages remains unknown. Here we present the in situ structure of the neck-tail complex of the siphophage lambda wild type, which lacks side tail fiber. The neck-tail complex comprises a channel formed by stacked 12-fold and 6-fold symmetrical rings and a 3-fold symmetrical tip. The interactions among DNA and a total of 246 tail protein molecules responsible for neck–tail assembly and stabilization have been characterized. The DNA in the neck is clamped by the adaptor protein rather than the stopper protein. Structural comparisons of the tail tips, the most diversified region across the lambda and other long-tailed phages or tail-like machines suggest that their tail tip contains conserved domains, which facilitate tail assembly, receptor binding, cell adsorption, and DNA retaining/releasing. These domains are distributed in different tail tip proteins in different phages or tail-like machines. The side tail fibers are not required for the perpendicular adsorption of lambda.

Introduction

Bacteriophages are the most abundant and diverse biological entities in the biosphere [1]. The majority of known phages belong to the order *Caudovirales* and contain a tail attached to the pentameric vertex of the icosahedral capsid (head) throug[illegible] portal or through a portal in complex with neck (connector) proteins [2]. According to the tail morphology, bacteriophages are divided into three families: Podoviridae (short tail), Myoviridae (long contractile straight tail), and Siphoviridae (long noncontractile flexible tail) [3]. The phage tail plays key roles in host cell recognition and interaction. In addition, the tail serves as a channel for viral genome delivery during infection; therefore, the tail structure is an extremely interesting study subject in terms of its assembly, host recognition, and cell wall perforation mechanisms [4].

The tail structure of phages belonging to Caudovirales phage has been studied extensively. The tails of Podophages and Myophages are straight; therefore they are suitable for high-resolution structural analyses. The tail structures of these phages ~~that~~ have been reported at high resolutions. These structures include: the isolated portal-tail complex of Podophage T7 [5] and the entire tail of Podophages T7, Pam1, sf6, and phi29 [6-9], the baseplate with two tail tube rings and sheath proteins of myophage T4 [10], and the tail tube of the Myophage T4 [11]. However, the resolution of the entire tail structure of siphophages is difficult to improve because of its intrinsic flexibility. Some monomeric tail proteins and isolated tail complexes have been resolved from subnanometer to atomic resolutions. For example, the structures of the monomeric siphophage tail and connector proteins have been determined using nuclear magnetic resonance (NMR) spectroscopy and X-ray crystallography [12-17]. In addition, the structures of the recombinant tail tubes, tail tips, and isolated tails of Siphophages have been determined by using X-ray crystallography and using cryo-electron microscopy (cryo-EM) focusing on relatively rigid regions of tail tubes, tail tips, or connectors [18-26]. The structures of a gene transfer agent (GTA, an evolutionarily related phage-like particle) [27] and type VI secretion systems (T6SS, phage tail-like bacterial nanomachines) [28, 29] have also been reported. However, the ~~in situ high-resolution~~ structures of the neck, tail tube, and tail tip in the Siphoviridae phages remain unknown, limiting the understanding of the assembly and infection mechanisms of siphophages.

The temperate phage lambda, which infects the bacterium *Escherichia coli*, belongs to

Siphoviridae. The lambda phage is used as a model system and is important in the attainment of the current understanding of the sophisticated and complex mechanisms by which the expression of genesis is controlled, the understanding of DNA virus assembly, and the understanding of the molecular nature of lysogeny [30]. Moreover, the lambda phage has various therapeutic and diagnostic applications [31]. The virion of the lambda phage comprises an icosahedral head and a long flexible tail [2]. The head consists of a total of 415 copies of coat protein gpE, 420 copies of cement protein gpD, 12 copies of portal protein gpB, and 48.5 kbp double-stranded DNA (dsDNA) [32]. The dodecameric portal is located at a unique icosahedral vertex and facilitates the transport of DNA in and out of the head during genome packaging and delivery [33]. The tail, which comprises the tail tube and tail tip, connects with the head through the neck. The portal, neck, and tail tube form a channel, in which the end of the dsDNA and an oligomer of tape measure protein are located [4, 34].

Here, we present the cryo-EM structure of the phage lambda, which is a laboratory-adapted strain and is called “lambda wild type” [35]. Our lambda structure allows the head proteins and most proteins in the portal, neck, tail tube, and tail tip to be modeled in atomic detail. These proteins include the head proteins gpE and gpD, the portal protein gpB, the neck proteins gpW, and gpFII, the tail tube proteins gpU and gpV_N, and tail tip proteins gpM, gpI, gpL, gpJ, and gpH (S1 Table). Our structure shows interactions among these 246 tail protein molecules responsible for the tail assembly, receptor binding, cell adsorption, and DNA retaining/releasing. Structural comparisons between the tail tips of lambda and other long-tailed phages or tail-like machines indicate that the major domains in the tail tips are structurally conserved, although they are distributed in different tail tip proteins in different phages or tail-like machines.

Results and Discussion

Overall structure of lambda phage

Cryo-EM image shows that the mature virion of the lambda phage comprises an icosahedral head with a long and flexible tail (S1A Fig). The head structure was reconstructed at a resolution of 3.5-Å by imposing the icosahedral symmetry (S1B and S2 Figs). The head comprises a DNA-containing capsid shell formed by the coat protein gpE and decorated by the

trimers of the cement protein gpD. Our structure of the head is essentially identical to that reported by a recent study on the lambda head [36]. We selected virion particles with a straight tail, and reconstructed the entire virion structure at a resolution of ~20 Å (Fig 1A and B) using our symmetry-mismatch reconstruction method [6, 37] (The software package can be downloaded from https://cryoem.hunnu.edu.cn). To improve the resolution of the tail structure, we used our local refinement and reconstruction method [6, 38] focusing on the portal, neck, tail tube, and tail tip. A structure of the portal–neck–tail complex at ~3.5 Å resolution was obtained by merging locally reconstructed structures (Fig 2). The tail, which comprises a long tail tube and a distal tail tip, attaches to the head through the neck (Fig 2A). The portal, neck and tail tube form a long channel, through which a rod-like structure passes (Fig 2A and B, Fig 1C). We build atomic models (S1 Table) for the portal (12-fold rings of the portal protein gpB), neck (including a 6-fold ring of the adaptor protein gpW and a 6-fold ring of the stopper protein gpFII), tail tube (including a 6-fold ring of the tail terminal protein gpU and thirty-two 6-fold rings of the tail tube protein gpV), and tail tip (including a 6-fold ring of the distal tail protein gpM, 3-fold trimers of the hub protein gpL, insertion protein gpI, central fiber protein gpJ, and an C-terminal α-helix of the tape measure protein gpH) (Figs 2 and 3, S3 Fig). Our mass spectrometry results (S2 Table) show that the lambda virion consists of proteins gpE, gpD, gpB, gpW, gpFII, gpU, gpV, gpM, gpH, gpL, gpI and gpJ, as well as tail completion protein gpZ and protease protein gpC. The side tail fiber proteins (stf and tfa) and gpK were not identified using mass spectrometry. The reason for the absence of the side tail fiber proteins is that the stf gene is disrupted in the genome of lambda wild type by frameshift mutation [35]. As for gpK, it is speculated that it is a nonstructural protein that is not present in the lambda virion. Among the identified lambda proteins, gpZ and gpC were not resolved in our structures. It is speculated that gpZ may lack symmetry in the tail. Indeed, gpZ homologous protein p143 in the siphophage T5 was observed to present as a monomer on the tail tip [26]. The gpC molecules might be distributed asymmetrically within the head.

Fig 1. Symmetry-mismatch reconstruction of the intact lambda virion. (A) Image of a lambda particle with a straight tail. (B) Overall view of an intact lambda phage at a resolution of approximately 20 Å. The numbers of the tail rings are labeled. (C) Slab view of the neck showing

the DNA within the neck (red arrow). (D) Density map (transparent) at a resolution of 3.5 Å superimposed on the atomic models of gpW (cyan ribbon) and gpFII (purple ribbon), showing the interactions between the Arg32 residue of gpW and dsDNA. (E) Density map (transparent) at a resolution of 3.5 Å superimposed on the atomic model of gpB (hot pink ribbon), showing the interactions between the Gln379 residue of gpB and dsDNA.

Fig 2. Structure of the lambda virion. (A) Left: scheme of the lambda virion. Each protein is shown using a different color and the numbers indicate the copy number of the proteins. Middle and right: surface and cut-open views of the lambda virion. The head, neck, tail tube, and tail tip are colored in grey, magenta, sky blue, and golden, respectively; one of the six protrusions of each ring of the tail tube is presented in blue to depict the right-handed helix assembly of the tail tube. (B) Surface and slab views of the portal (gpB), neck (gpW, and gpFII), tail terminator (gpU), and two tail tube (gpV_N) rings at a resolution of 3.5 Å. The thick dashed line marks the capsid border, and different colors have been used for different proteins. (C) Surface and cut-open views of the tail tip with a gpV_N ring. Different colors have been used for different proteins. (D-E) Superposition of the resolved models of gpI and gpH on their density maps (transparent) indicates the quality of the density maps.

Fig 3. Structure of the portal and neck. (A, B) Overall and slab view of the atomic model (ribbon) of the neck. The color codes for portal (gpB), adaptor (gpW), and stopper (gpFII) are identical to those in Fig. 2B. The gray rod represents the density of phage DNA. (C) Structure of gpB shown using various colors for its domains. (D) Density map (transparent) of the gpB tunnel loop superimposed on its atomic model. (E, F) Side and top views of the adaptor ring. A copy of gpW is shown using various colors for its domains. (G) Zoom-in view of the gpW structure in panel (E). (H) NMR structure of gpW in solution (PDB ID: 1HYW) showing that the β-hairpin domain undergoes a 90°rotation. (I) Zoomed-in view of the black box in panel F showing the hydrophobic interactions in the α-helix bundle domains of two adjacent gpW molecules. (J) C-arm of gpW interacts with gpB through β-sheet augmentation. (K, L) Side and top views of the stopper. One gpFII molecule is shown using various colors for its domains. (M) Zoomed-in view of gpFII in panel (K). (N) NMR structure of gpFII in solution (PDB ID: 1K0H). (O) Cross-section view of black column in panel A showing the interactions among gpFII, gpW, and DNA.

Structure of the portal and neck

The portal occupies one of the 12 vertexes of the icosahedral head. It is formed by 12 copies of gpB (Figs 2A, 2B, 3A, and 3B), which exhibits the canonical portal fold of phages belonging to the order *Caudovirales* (S4 Fig) [39]. Excluding [illegible]3 N-terminal residues, 20 C-terminal residues, and a loop (residues 303-319), which were missing at the outer surface of the portal, the resolved structure of gpB comprised four domains (Fig 3C): wing (residues 24-249 and 368-452), stem (residues 250-279, 343-367), clip (residues 280-342), and crown (residues 453-511). The tunnel loop, which was previously indicated to clamp dsDNA to avoid leakage during the phage maturation [33], was well resolved (Fig. 3C and 3D). The asymmetric reconstruction of the portal and capsid at a resolution of 4.0 Å (S1B Fig) showed the asymmetric interactions between the gpB and gpE subunits, as observed in the structures of phages sf6 and T4 [9, 40]. The portal–capsid interface was analyzed using the PISA server [41], which revealed the salt bridges between the gpB and gpE subunits (S5A and B Fig). Superimposition of the models of the 12 portal subunits revealed that a loop (residues 208-218) in the gpB wing domain underwent structural morphing to adapt to the portal–coat interactions (S5C Fig).

The neck comprises two rings composed of the adaptor and stopper proteins (Figs 2B, 3A, and 3B). The adaptor ring, which is formed by 12 copies of gpW, is assembled below the portal (Fig 2B). A total of 66 residues (4-68) of the 68-residue adaptor protein gpW were resolved. The structure of gpW comprises three domains: a β-hairpin (residues 23-36), a two-α-helix bundle (residues 4-22 and 37-54), and an extended C-arm (residues 55-68) (Fig 3E-G, and S3B Fig). The assembly of the adaptor relies on hydrophobic interactions among the subunits of gpW (Fig 3F, 3I). The interactions of the adjacent gpW subunits are further reinforced by interactions between the adjacent β-hairpin domains, which form a β-barrel (Fig 3E). Compared with the NMR structure of a gpW monomer in solution [42] the β-hairpin in our gpW structure undergoes a 90° rotation, from parallel to vertical with respect to the two-α-helix bundle domain (Fig 3G and H), which facilitates the formation of the β-barrel structure. The assembly of the adaptor on the portal mainly relies on the electrostatic interactions between the electropositive adaptor top and the electronegative portal bottom (Fig 4). In addition, the C-arm, which is absent from the NMR structure, interacts with residues 320-324 of the portal wing through β-sheet augmentation (Fig 3A and J).

Fig 4. Electrostatic potential surfaces of the interacting regions of two adjacent rings.

Six copies of the stopper protein gpFII are assembled into a 6-fold ring connecting the adaptor (Figs 2B, 3K, and 3L). We resolved residues 4-117 of the 117-residue protein gpFII (Fig 3K-M, and S3C Fig). The gpFII structure comprises four domains (Fig 3M): an N-terminal α-helix (residues 4-24), a β-sandwich (residues 25-41 and 63-107), a β-hairpin (residues 46-62) inserted in the β-sandwich, and a C-terminal loop (residues 108-117). A comparison of our gpFII structure with the NMR structure of gpFII monomer in solution [43] revealed that the structure of gpFII monomer in solution undergoes notable conformational changes (Fig 3M and N) to reach the assembled state. The N-terminal loop becomes the α-helix domain. Six copies of the α-helix domains form a ring, into which the β-barrel of the adaptor rests (Fig 3A, B, and O). Two other conformational changes are involved in the assembly of the tail tube ring (described below).

Structure of the tail tube

The tail tube is located below the neck. It comprises a 6-fold ring of the tail terminal protein gpU and 32 repetitively stacked 6-fold rings of tail tube protein gpV (Figs 1A and B, 2A, 5A, 5B). The 32 rings form a right-handed helix with an axial rise of ~42 Å and a twist of ~17.5° for two adjacent rings (Fig 2A). All 131 residues of gpU comprise an antiparallel five-strand β-sheet flanked by two α-helices, with an O-shaped loop (O-loop, residues 25-37) inserted between β1 and β2 and an extended-loop (E-loop, residues 45-58) inserted between β2 and β3 (Fig 5C, and S3D Fig). The tail tube connects with the neck through interaction between the ring of the stopper protein gpFII and that of the tail terminal protein gpU (Fig 2A and B). To adapt to ring-ring interactions, the long loop (residues 46-62) observed in the structure of gpFII monomer in solution [43] becomes a β-hairpin (Fig 3M and N), which inserts into the inner surface of the gpU ring and interacts with the O-loop of gpU through β-sheet augmentation (Fig 5D). The C-terminal loop (residues 46-62) observed in the structure of gpFII monomer in solution [43] rotates and electrostatically interacts with the O-loop of the adjacent gpU subunit (Fig 5D). The E-loop of gpU provides an attachment site for the major tail protein gpV through electrostatic interactions (Figs 5A and I, and 4). The surface of the gpU ring is strongly negatively charged (Figs 4 and 6E), probably facilitating the correct positioning of the

packaged DNA.

Fig 5. Structure of the tail tube. (A) Rings of gpFII, gpU, and gpV_N (only three rings of gpVN are shown). The color codes are identical to those in Fig. 2B, except for the fact that one gpU molecule and one gpV_N molecule are shown in rainbow colors, ranging from blue at the N-terminus to red at the C-terminus. (B) Top view of the gpU ring. One of the six gpU molecules is shown in rainbow colors. (C) Structure of the gpU molecule in the tail tube. (D) Zoomed-in view (center) of the red box in panel A showing β-sheet augmentation (left) and electrostatic (right) interactions between gpU and gpFII. (E) Structure of the gpV_N molecule present in the tail tube. (F) NMR structure of gpV_N in solution (right; PDB ID: 2k4q), showing conformational changes in the E-loop and N- and C- termini. (G) Top view of the hexameric gpV_N ring. One of the six gpU molecules is shown in rainbow colors. (H) Zoomed-in view (left) of the blue box in panel A showing electrostatic interactions (right) between two adjacent gpV_N rings. (I) Zoomed-in view (left panel) of the black box in panel A showing electrostatic interactions between gpU and gpV_N (right panel).

Fig 6. Density map of the tail tube. (A) Two-dimensional class average of the tail tube. White, cyan, and orange arrows indicate the rings of gpV_N and gpV_C and the rod of gpH or dsDNA, respectively. (B) Density map of the tail tube filtered to a resolution of 6 Å. Superposition of an atomic model of gpV_C (PDB DI: 2L04) on the protrusion (right). (C) Side and top views of the high-resolution density map of the tail tube. One monomer of gpV_N is shown in yellow. (D) Cut-open view of the tail tube. (E) Electron potential on the inner channel of the neck-tail complex.

Each of the 32 rings of gpV is assembled by six copies of the major tail protein gpV wrapped around an oligomer of the tape measure protein gpH (Fig 2B). GpV has two distinctive domains: the N-terminal domain (gpV_N, residues 3-156) and the C-terminal domain (gpV_C, residues 157-246). The atomic structures of the two individual domains in solution have been determined by NMR spectroscopy [16, 17]. Similar to the previously lower-resolution cryo-EM structure on the lambda tail tube [22], our structure filtered to 6 Å resolution revealed six protrusions around each gpV ring; each protrusion fits well with the gpV_C atomic model, suggesting that the protrusions represent the gpV_C domains (Fig 6A and B). At a higher resolution, the protrusions became unstructured, presumably due to their flexibility. The inner

tube structure could be reconstructed to a resolution of 3.5 Å, which enabled us to build an atomic model of the gpV_N domain (Fig 6C; S1B, S3E Figs). The gpV_N domain comprises a β-sandwich flanked by an α-helix. The β-sandwich is inserted by an E-loop (residues 50-78) between β3 and β4, and by an O-loop (residues 24-38) between β1 and β2 (Fig 5E; S3E Fig). Six copies of the β-sandwich form the core of the tail tube ring (Fig 5G). The outer surface of the ring is formed by the α-helix and O-loop. Our structure of gpV_N is similar to the previously reported NMR structure of gpV_N [16], however, the N-terminal, E- and the C-terminal loops are flexible in the NMR structure (Fig 5F). These loops resulted in electrostatic interactions between the adjacent gpV_N rings (Figs 5H, and 4) and between the gpU and gpV_N rings (Figs 4 and 5I). The flexibility of the tail tube may result from the interactions among the loops. The inner surface of the gpV ring mostly bears a negative charge (Fig 6E), similar to the tail tubes of phages T5 and 80α [15, 18], facilitating DNA transfer.

The architecture of gpV_N is structurally conserved across the phage tails (S6 Fig). The results of HHpred analysis indicated that the topological structure of lambda gpV_N matches those of SPP1 gp17.1, 80α gp53 and *Rhodobacter capsulatus* GTA (RcGTA) gp9 more than 99% probability (S3 Table) [18, 20, 27, 44], although their sequence identities are low (12% to 14%).

DNA in the neck-tail tube channel

The rod-like structure in the central channel of the neck and tail tube can be attributed to dsDNA and an oligomer of the tape measure protein gpH [4]. The genomic dsDNA of phages belonging to *Caudovirales* is packaged through the portal during phage assembly [45], and the end of the last packaged dsDNA is retained in the portal [39]. In phages belonging to *Caudovirales*, including siphophage SPP1 [12, 21], the narrowest part of the portal channel is formed by 12 tunnel loops. The tunnel loop, which clamps dsDNA in the portal tunnel in other phages [6, 21], does not interact closely with the dsDNA in lambda phage (Figs 1C, 1E, and 3B). In addition, the stopper ring (gpFII) of the lambda structure exhibits an open conformation (Fig 2B). This finding differs from that of previous structural studies reporting that the dsDNA end of SPP1 stops at the stopper ring (formed by gp16), at which the channel is closed by the loops of gp16 [21, 25]. It is intriguing that 12 arginine residues (residue 32), which are contributed by the 12 adaptor protein gpW molecules, clamp the dsDNA (Figs 1C, 1D, 2B, and 3O). This finding is consistent with a previous study suggesting that gpW is required for the

stabilization of lambda DNA within the head [42]. The gpV tube wraps around the gpH oligomer, which determines the length of the tail tube during assembly [46]. The gpH oligomer inside the tail tube cannot be well resolved probably because of its asymmetric assembly in the tail tube (Fig 6D).

Structure of the tail tip complex

The tail tip complex is located at the distal end of the siphophage tail, which is the most diversified tail region. This complex is essential for host recognition and DNA ejection [18]. It comprises six proteins: the distal tail protein gpM, hub protein gpL, central fiber protein gpJ, insertion protein gpI, and fiber proteins stf and tfa [34]. The side tail fiber proteins stf and tfa were absent from the lambda structure because stf is prevented in lambda wild type by the frameshift mutation [35].

Our structure revealed that the tip is similar to an inverted cone of 410 Å in length (Fig 2C, 7A-C). We resolved all 109 residues of the distal tail protein gpM, which has two conformers with minor differences in the E-loop (Fig 7D, S3F Fig). Except that the gpV$_N$ has an extended N-terminus and C-terminus, gpM is topologically identical to gpV$_N$ (S6 Fig). GpM also has a β-sandwich, which is inserted by an E-loop and flanked by an α-helix (Fig 7D). The O-loop of gpV$_N$ is absent from gpM because of the shorter N-terminus of gpM. Structural comparisons indicate that the architecture of the gpM domain is conserved across siphophage tails (S6 Fig). Six copies of the two gpM conformers form a 3-fold gpM ring, which anchors the tail tip to the 6-fold tail tube through the β-sandwich domain via electrostatic interactions (Figs 4, 7B and E). The other side of the gpM ring (E-loop) interacts with the 3-fold ring of the gpJ and gpL complex.

Fig 7. Structure of the tail tip. (A) Structure of the tail tip. The C-terminal region of gpJ, which was not resolved to the near-atomic resolution, is displayed using a density map (grey). (B) Top and bottom views of the gpM ring. One of the six copies of gpM is shown in rainbow colors. (C) Cross-section view of black column in panel A showing that the proteins gpJ and gpL form a trimeric conical tip. (D) Two conformers of gpM shown in rainbow colors. (E) Zoomed-in view (top left) of the blue box in panel A showing electrostatic interactions between gpM and gpV$_N$ (top right) and among gpM, gpJ, and gpL (bottom panels). (F) Structure of gpL shown using various colors for its

domains (left) and the zoomed-in view (top right) of the model of the iron–sulfur cluster superimposed on its density map (transparent). A central section of the iron-binding site was shown (bottom right). (G) Ribbon model of gpJ shown using various colors for its domains. (H) Side and top views of trimeric gpI (red) and gpH (golden). (I) β-sheet augmentation-mediated interactions between gpI (red) and gpJ (green).

According to the four "Hub Domain" (HD) nomenclature of the siphophage and myophage baseplate hub proteins (BHPs) [4], gpJ includes the domains of HDII, HDII-insertion, HDIII, and HDIV (Fig 7G). Structural comparison between gpJ and siphophage T5 BHP pb3 [26] revealed that (i) gpJ did not have the HDI domain, and the lambda HDI domain was located in gpL (described below); (ii) the gpJ HDIV domain had an additional β-sandwich (residues 483-569) inserted between β24 (residues 477-482) and β25 (residues 570-578) (S7A and B Fig); (iii) the gpJ HDII-insertion domain had a shorter N-terminus compared with T5 pb3. The missing N-terminus of the gpJ HD-insertion could be compensated by the C-terminal domain of gpL (S7C and D Fig, described below); and (iv) an additional Ig-like domain (residues 72-215) inserted between β4 (residues 65-71) and β13 (residues 216-223) of the gpJ HDII-insertion domain (Fig 7G; S7A and D Fig). The results of positive-specific iterative basic local alignment search tool analysis revealed that the Ig-like domain belonged to the fibronectin type III domain family [47]. Thus, we designate the Ig-like domain FNIII-A (Fig 7G; S7A Fig). Except for these differences, these gpJ domains are very similar to their counterparts in T5 [26] with RMSDs ranging from 1.5 to 3.0 Å (Fig 8).

Fig 8. Structural comparison between lambda, other phages, and tail-like machines. (A-F) Structure of the hub and central fiber proteins of lambda (gpL and gpJ), the baseplate hub or homologous proteins of phage T5 (7ZQB), phage 80α (PDB ID: 6V8I), T4 (PDB ID: 5IV5), T6SS (PDB ID: 6H3L), and RcGTA (PDB ID: 6TEH). The homologous domains in these proteins are in the same color. The N- and C- termini of these proteins were labeled. For the N- and C- termini of gpL and gpJ, see Fig S7A and C for separate views of gpJ and gpL.

The four-HD scaffold is followed by two Ig-like domains of the fibronectin type 3

sequence family (FNIII-1 and FNIII-2) in both lambda gpJ and T5 BHP pb3. The two consecutive FNIIIs are connected to the HDIV through a linker domain composed of a long loop and a short α-helix (Fig 8; S7A and B Fig). In the T5 tail tip, the three copies of the two FNIIIs from three copies of pb3 are separated, which is caused by the negative patch at the surface of the pb3 FNIIIs [26]. By contrast, in the lambda tail tip, the three copies of the two FNIIIs from three copies of gpJ form a closed cone-like distal end of the tail tube (Fig 7A). The close interactions between these FNIIIs are enforced by a three-helix bundle below (Fig 7A). These Ig-like domains, which exist ubiquitously in phage tails, may aid in the adhesion of phage particles to bacterial cell surfaces through weakly binding to carbohydrate cell wall components such as peptidoglycan or lipopolysaccharide [48].

The protein gpL contains an N-terminal gpV_N-like β-sandwich domain (residues 1-166), which corresponds to the HDI domain of phage T5 pb3. Therefore, we designate the N-terminal region as the HDI domain. Three copies of the gpL HDI domains and three copies of gpJ HDII domains form the last ring of the tail. This ring is electrostatically stacked on the E-loops of the gpM ring (Figs 4 and 7A). The E-loops of the six gpM molecules exhibit two conformations to adapt to the 3-fold symmetry of the last tail ring (Fig 7D). The C-terminal domain (residues154-232) of gpL is topologically similar to the N-terminus of the T5 pb3 HDII-insertion domain (residues 160-209; S7E Fig), that is to say, the C-terminal region of gpL (iron-binding domain) and the N-terminal region of gpJ together correspond to the T5 pb3 HDII-insertion domain (S7A, C, and D Fig).

In addition, the C-terminal domain of gpL is an iron-binding domain. A biochemical study revealed highly conserved cysteine residues coordinating an oxygen-sensitive $[4Fe\text{-}4S]^{2+}$ cluster in the C-terminal domain of gpL and homologous tip proteins of other siphopahges [49]. In our gpL structure, an additional density feature in the iron-binding domain, which was surrounded by four cysteine residues (Cys173, Cys182, Cys205 and Cys212) and could not be assigned to any main chain or side chain, could be modeled as the iron-sulfur cluster (Fig 7F). The iron-binding domain, which was previously hypothesized to be embedded inside the tail tip and help stabilize gpL in its assembled form [49], was found to be located at the outer surface of the tip (Fig 7A). Therefore, the functions of the iron-sulfur cluster, which is universally across gpL homologs [49], remain to be characterized.

The density in the tail tip lumen was modeled as a trimer of the gpI (residues 135-223) and a three-helix coiled coil belonging to gpH (residues 818-842; Figs 2D, 2E, 7C, and 7H). The gpI trimer forms a tripod to support the coiled coil in the lumen (Fig 7C and H), and each gpI molecule interacts with the HDII, HDII-insertion, and FNIII-A of gpJ through three β-sheet augmentations (Fig 7I). The gpH coiled coil terminates the rod-like structure in the tail tip lumen (Fig 2C). A sequence-based secondary structures prediction study [50] revealed that the α-helix is located at the C-terminus of helical gpH (S8 Fig). Tape measure protein is a multifunctional protein found in both *Siphoviridae* and *Myoviridae*. During tail assembly, tape measure protein interacts with chaperone proteins to initiate tail polymerization and determines the length of the assembled tail [46, 51, 52]. In addition, it may transmit the host-binding signal to the phage capsid and then exit from the tail and form a channel to span the host cell envelope for phage DNA delivery [15, 53, 54]. Our structure suggests that the gpH oligomer is formed by gpH trimer with the gpH C-terminus located in the tail tip. The gpI trimer serves as a plug to prevent the early release of the gpH oligomer and DNA from the tail tube (Fig 7H). In T5, a plug domain is inserted in the HDII-insertion domain of BHP. Three copies of the plug domains also form a tripod and serve to close the tube [26] in the same location as the gpI tripod in lambda (Fig 8A and B; S7B and D Fig). It is noteworthy that gene I is located between genes L and J, a gene location that corresponds to the position of the T5 pb3 plug domain, except for an inserted gene K (S9 Fig). Therefore, we suggest that gpL, gpI, and the N-terminal region of gpJ (from HDII to FNIII-1 and -2) together correspond to T5 BHP pb3 (S9 Fig). The resolved N-terminal region of gpI is highly hydrophobic (residues 137-GILFSLGASMVLGGVA-152), suggesting that it might interact with the bacterial outer membrane, as does the T5 pb3 plug domain [26].

Further structural comparisons of the gpL and gpJ proteins of lambda with the homologous proteins of phage T4 [10], phage 80α [18], RcGTA [27], and T6SS [28] revealed that they all contained four HD domains (Fig 8), suggesting they share a common evolutionary origin. However, T4, 80α, and T6SS lack HDII-insertion and FNIII domains. For T5, T4, 80α, and T6SS, the four HD domains are located within a single protein. By contrast, RcGTA has similar organization of the four HD domains to that of lambda. The RcGTA HDI domain is located in the N-terminal regions of gp13, while its rest three HD domains are located in gp15 (Fig 8F).

As in lambda, the RcGTA HDII-insertion domain is also distributed in two tail tip proteins: the N-terminal region of the HDII-insertion domain is located in the C-terminal region of gp13, while the C-terminal region of the HDII-insertion domain is located in the N-terminal region of gp15 (Fig 8F). Indeed, the arrangement of RcGTA genes 13, 14, and 15 [27] is very similar to that of lambda genes L, K, I, and J (S9 Fig). It is noteworthy that both lambda and RcGTA have iron-sulfur clusters in the tail tip protein (gpL/gp13). Among these tail tip proteins, only lambda gpJ and T5 pb3 contain FNIII domains. The insertion/deletion and distribution of domains in these tail tips reflect the long evolutionary history of these phages and phage tail-like machines.

In addition to the aforementioned domains corresponding to T5 BHP, gpJ also contains two additional domains in its C-terminal region: a central fiber domain and a receptor-binding domain, which are the counterparts of the T5 central fiber protein pb4 and the receptor binding protein pb5 respectively. These two domains were not resolved at high resolution (Fig 7A) due to their flexibility. The flexibility of the two tail tip domains may enable the receptor binding domain to explore larger spaces for the bacterial receptor. We could only model the N-terminal regions of the three central fiber domains as three-helix bundle based on the gpJ density map (Fig 7G). The helix bundle links the central fiber and receptor binding domains to the FNIII-2 domain (Fig 7A). We performed structure predictions for gpJ (S10A Fig) by using AlphaFold2 [55]. Comparison between the predicted gpJ structure and our gpJ structure in the tail tip showed that the domains of HDII, HDII-insertion, HDIII, and HDIV fit well (RMSD: 1.7 Å), except for the deviation of the FNIII-A, -1, and -2 domains (S10B Fig). If we segment the three FNIII domains from the predicted gpJ structure, they can fit well into their counterparts in our gpJ structure, with RMSDs ranging from 0.7 to 1.8 Å. The deviation of the FNIII-1 and -2 in the predicted gpJ structure was caused by the tilt of the linker domain (S10B Fig). Furthermore, the gpJ central fiber domain was predicted to be a β-strand rich region (S10A Fig). It is likely that these β-strands form β-helix in trimer, as the T5 central fiber protein pb4 [26]. The C-terminal tip of gpJ was predicted to be an Ig-like domain (S10A Fig), which could be attributed to the receptor-binding domain [56]. Through the receptor-binding domain, gpJ binds to the receptor protein LamB on the surface of the host cell [57-59]. The two gpJ domains were curved in the predicted structure (S10 Fig), in contrast, they appeared to be relatively straight in the

density map (Fig 7A). The difference between the predicted gpJ structure and our gpJ structure might reflect the conformational change of gpJ from the monomer state in solution to the assembled state. It can be inferred that the tail tip of lambda would undergo structural changes upon receptor interaction similar to the structural changes of T5 [26]. The receptor binding process may trigger conformational changes in gpJ, which presumably disrupt the interactions among gpL, gpI, and gpJ, thus initiating the release of gpH oligomer and DNA. Ultrathin sections of the lambda infected E. coli cells analyzed using electron microscopy showed that the lambda wild type particles adsorbed perpendicular to the cell surface (S11 Fig), although these particles lacked side tail fibers, suggesting that the perpendicular adsorption did not need the participation of the side tail fibers. This observation was in contrast to the adsorption of podophage T7, in which the six distal halves of the six side tail fibers extended vertically on the outer membrane to support the T7 particles, allowing them to orient perpendicularly to the cell surface [60]. It is probable that the conformational changes of gpL, gpI, gpJ, and the released gpH are sufficient to facilitate the lambda head and tail tube to orient perpendicularly to the cell surface.

Materials and Methods

Bacteriophage lambda purification

The E. coli strain MG1655 (American Type Culture Collection ID 47076) was grown in Luria-Bertani Broth (tryptone, 10 g; yeast extract, 5 g; and NaCl, 10 g/L) for 24h at 37°C. The cultured strain was re-cultured at 37°C for 4h. During the logarithmic growth of *E. coli*, the bacterial cells were incubated with lambda W60 phages for 4h at 37°C. The phage culture was lysed using chloroform. Subsequently, the cell debris was removed through centrifugation at 6000 × g for 20min at 8°C. The supernatant was enriched through polyethylene glycol (PEG) 8000 precipitation (10% w/v PEG in 1M NaCl) overnight at 4°C. The precipitated phages were resuspended in phage buffer (10 mM $MgSO_4$ and 50 mM Tris-HCl, pH 7.4) and then purified by cesium chloride density gradient centrifugation at 90,000 × g for 2h at 8°C. After centrifugation, bands containing phage particles were collected and dialyzed in phage buffer (10 mM $MgSO_4$ and 50 mM Tris-HCl, pH 7.4) overnight. Final, purified infected lambda phages were in ice-water for cryo-sample processing.

Ultrathin section of lambda-adsorbed cells

The lambda phage and bacteria mixture was incubated in 37℃ water bath for 15 min and then pelleted by low speed centrifugation. The pellets were fixed with 2% paraformaldehyde-2.5% glutaraldehyde buffered in 0.1 M sodium cacodylate overnight and post fixed with 1% osmium tetroxide for 2 hrs. Then the samples were dehydrated with gradient alcohol of increasing concentration until 100%. Finally, the samples were infiltrated and embedded in epoxy resin. The resin blocks were cut using ultramicrotome to get 80nm thickness sections which were stained with 1% uranyl acetate and 0.4% lead citrate, respectively. The ultrathin sections were examined under an FEI TF20 200 kV electron microscope.

Mass spectrometry analysis

The purified lambda phage was run on a 4-12% SDS-PAGE, and the Coomassie-stained bands were manually excised for mass spectrometry analysis. The mass spectrometry data processing and analyses were performed using Q Exactive mass spectrometer (Thermo Scientific) and software package Proteome Discovery version 1.4. The Sequest HT search engine was used for sequence searches in the MS/MS spectra against the Uniprot database.

Cryo-EM and data collection

An aliquot of 3 µL purified lambda phages was applied to 400-mesh Quantifoil R2/1 copper grids, which were covered with an additional 5-nm-thick layer of continuous carbon using a Q150T turbomolecular pumped coater (Quorum Technologies, UK) and glow-discharged for 20 s. The grids were loaded into an FEI Vitrobot (temperature, 8 °C, humidity, 100%, and blot time, 3.5 s). After blotting the excess solution on the grid two times, the grids were plunge-frozen using solid–liquid ethane and stored in liquid nitrogen until subsequent analyses. Cryo-EM data were recorded using the 300 kV Titan Krios G3i microscope equipped with a Gatan imaging filter and a K3 summit direct electron detector, and a Cs corrector. The electron microscope was operated at 300 kV voltage, the Gatan imaging filter was used with a slit width of 20 eV to remove inelastically scattered electrons, and the K3 detector was operated in the super-resolution mode. The pixel size was calibrated using 500-nm diffraction grating replica and latex calibration standard. Automatic data were collected using the FEI EPU software at a magnification of 53,000 (physical pixel size: 1.36 Å), which resulted in a pixel size of 0.68 Å per pixel. The accumulated dose of each movie was 32 $e^{-}/Å^{2}$, and the defocus

values of the images ranged from 1.6 to 2.2 μm. Finally, 5513 movies were collected; each movie stack comprised 32 image frames.

Image processing

Icosahedral reconstruction for the head. The defocus and astigmatism values of each cryo-EM micrograph were calculated by using GCTF [61] in RELION software [62]. A total of 97,030 particle images of the lambda head (box size 1024×1024 pixels) were boxed out from 5,513 cryo-EM images using the software ETHAN [63]. The icosahedral head of lambda was reconstructed by using our programs [64] based on the common-line algorithm [65, 66].

Symmetry-mismatch and local reconstructions. We manually selected 10,220 images (box size 900×900 pixels) of the complete lambda particles with straight tails from the cryo-EM images, which were re-sampled to a pixel size of 5.44 Å. Based on the orientation and center parameters of each particle image obtained from the icosahedral reconstruction, we reconstructed the intact asymmetric structure of the phage lambda using our symmetry-mismatch reconstruction method [6, 37]. Briefly, for each particle image, we first localized the unique vertex (portal–neck) by searching the regions of 12 icosahedral vertices, which were determined in the icosahedral reconstruction step. Then, a low-resolution structure of the phage with the tail was obtained. The resolution was further improved through the iteration of the projection-refinement-reconstruction step. In this step, we selected one of the 60 possible icosahedral orientations for each particle image. The iteration was continued until the orientations of all particle images were stabilized and the phage structure (~20 Å resolution) could not be improved further. The aforementioned steps were performed for all 97,030 particle images. Thus, we obtained a phage structure with the portal–neck and the proximal part of the tail tube but without the distal part of the tail (because the tail is flexible) at a resolution of ~6 Å. Next, we segmented the portal–neck from the phage structure to use it as an initial model for local refinement and reconstruction; thus we determined the structure of the gpB (portal) and gpW (adaptor) rings at a resolution of 3.2 Å by imposing the 12-fold symmetry and the structure of the gpFII, gpU, and two proximal gpV rings at a resolution of 3.5 Å.

Local reconstructions of the tail tube and neck. The tail tube comprises 32 repetitive hexameric rings. To increase the particle number, we manually selected 181,657 tail tube particles by using the EMAN Helix software [67] with a box size of 220×220 pixels. Using the

low-resolution density map, which was segmented from the intact virion, as an initial model, we performed two-dimensional (2D) classification and three-dimensional (3D) reconstruction by using RELION software [62]. A total of 121,571 particle images were selected for performing auto-refinement and 3D reconstruction with the C6 symmetry imposed. Finally we improved resolution of the tail tube structure to 3.48 Å. The tail neck was reconstructed using the same procedure as the tail tube except that the box size of the neck images was 400×400 pixels.

Local reconstruction of the tail tip complex. We preformed the local reconstruction of the tail tip complex by using RELION software [62] (S12 Fig). First, 70,745 tail tip particle images with a box size of 220×220 pixels were manually selected for 2D classification to exclude non-tip and other irrelevant segments. The classification results indicated that the centers mostly varies across particles. Using a randomly generated cylinder density as the initial model, we selected 58,215 particles from 2D classifications to perform 3D classification with the C3 symmetry. Thus, we obtained six types of low-resolution structures with varying center positions (S12E Fig). Furthermore, we performed Z-axis translation to correct the center of each type on the basis of the first type and re-extracted tail tip images. Subsequently, 54,385 tail tip images were selected for 3D refinement with the C3 symmetry imposed. Thus we obtained a structure of the tail tip complex at a resolution of 3.72 Å. Finally, we refined the contrast transfer function to improve the resolution of the tail tip structure to 3.44 Å.

Model building

Using the COOT software [68], We manually built the atomic models of proteins gpB, gpW, gpFII, gpU, gpV$_N$, gpM, gpL, gpJ, gpI and gpH (residues 818-849) on the basis of our cryo-EM density map. Furthermore, we refined the models through in real-space refinement, implemented in Phenix [69]. The refinement and validation statistics are presented (S4 Table).

Acknowledgements

Cryo-EM data collection was carried out at the Shuimu BioSciences Ltd. We thank Fanhao Meng, Zhenqian Guo and other staff members at the Shuimu BioSciences for data collections.

Author contributions

H.L., L.C., and J.S. designed the research; H.X., J.S. produced the lambda sample and prepared cryo-EM grids; H.X., L.T., J.S. collected the cryo-EM data; H.X., L.T., Z.T., Y.Z., W.C., X.L., L.C., and H.L. analyzed data; H.X., L.T., L.C., and H.L. wrote the paper, with further edits from all authors.

References

1. Wommack KE, Colwell RR. Virioplankton: viruses in aquatic ecosystems. Microbiol Mol Biol Rev. 2000;64(1):69-114.
2. Ackermann HW. 5500 Phages examined in the electron microscope. Arch Virol. 2007;152(2):227-43.
3. Ackermann HW. Bacteriophage observations and evolution. Res Microbiol. 2003;154(4):245-51.
4. Linares R, Arnaud CA, Degroux S, Schoehn G, Breyton C. Structure, function and assembly of the long, flexible tail of siphophages. Curr Opin Virol. 2020;45:34-42.
5. Cuervo A, Fabrega-Ferrer M, Machon C, Conesa JJ, Fernandez FJ, Perez-Luque R, et al. Structures of T7 bacteriophage portal and tail suggest a viral DNA retention and ejection mechanism. Nat Commun. 2019;10(1):3746.
6. Chen WY, Xiao H, Wang XR, Song SL, Han Z, Li XW, et al. Structural changes of a bacteriophage upon DNA packaging and maturation. Protein Cell. 2020;11(5):374-9.
7. Zhang JT, Yang F, Du K, Li WF, Chen Y, Jiang YL, et al. Structure and assembly pattern of a freshwater short-tailed cyanophage Pam1. Structure. 2022;30(2):240-51 e4.
8. Xu J, Wang D, Gui M, Xiang Y. Structural assembly of the tailed bacteriophage varphi29. Nat Commun. 2019;10(1):2366.
9. Li F, Hou CD, Yang R, Whitehead R, 3rd, Teschke CM, Cingolani G. High-resolution cryo-EM structure of the Shigella virus Sf6 genome delivery tail machine. Sci Adv. 2022;8(49):eadc9641.
10. Taylor NMI, Prokhorov NS, Guerrero-Ferreira RC, Shneider MM, Browning C, Goldie KN, et al. Structure of the T4 baseplate and its function in triggering sheath contraction. Nature. 2016;533(7603):346-+.
11. Zheng WL, Wang FB, Taylor NMI, Guerrero-Ferreira RC, Leiman PG, Egelman EH. Refined Cryo-EM Structure of the T4 Tail Tube: Exploring the Lowest Dose Limit. Structure. 2017;25(9):1436-+.
12. Lhuillier S, Gallopin M, Gilquin B, Brasiles S, Lancelot N, Letellier G, et al. Structure of bacteriophage SPP1 head-to-tail connection reveals mechanism for viral DNA gating. P Natl Acad Sci USA. 2009;106(21):8507-12.
13. Veesler D, Robin G, Lichiere J, Auzat I, Tavares P, Bron P, et al. Crystal Structure of Bacteriophage SPP1 Distal Tail Protein (gp19.1) A BASEPLATE HUB PARADIGM IN GRAM-POSITIVE INFECTING PHAGES. Journal of Biological Chemistry. 2010;285(47):36666-73.
14. Langlois C, Ramboarina S, Cukkemane A, Auzat I, Chagot B, Gilquin B, et al. Bacteriophage SPP1 Tail Tube Protein Self-assembles into beta-Structure-rich Tubes. Journal of Biological Chemistry. 2015;290(6):3836-49.
15. Arnaud CA, Effantin G, Vives C, Engilberge S, Bacia M, Boulanger P, et al. Bacteriophage T5 tail tube structure suggests a trigger mechanism for Siphoviridae DNA ejection. Nat Commun. 2017;8.
16. Pell LG, Kanelis V, Donaldson LW, Howell PL, Davidson AR. The phage lambda major tail protein structure reveals a common evolution for long-tailed phages and the type VI bacterial secretion system. Proc Natl Acad Sci U S A. 2009;106(11):4160-5.
17. Pell LG, Gasmi-Seabrook GM, Morais M, Neudecker P, Kanelis V, Bona D, et al. The solution structure of the C-terminal Ig-like domain of the bacteriophage lambda tail tube protein. J Mol Biol. 2010;403(3):468-79.
18. Kizziah JL, Manning KA, Dearborn AD, Dokland T. Structure of the host cell recognition and penetration machinery of a Staphylococcus aureus bacteriophage. Plos Pathog. 2020;16(2).
19. Hardy JM, Dunstan RA, Grinter R, Belousoff MJ, Wang J, Pickard D, et al. The architecture and

stabilisation of flagellotropic tailed bacteriophages. Nat Commun. 2020;11(1):3748.
20. Zinke M, Sachowsky KAA, Oster C, Zinn-Justin S, Ravelli R, Schroder GF, et al. Architecture of the flexible tail tube of bacteriophage SPP1. Nat Commun. 2020;11(1):5759.
21. Chaban Y, Lurz R, Brasiles S, Cornilleau C, Karreman M, Zinn-Justin S, et al. Structural rearrangements in the phage head-to-tail interface during assembly and infection. Proc Natl Acad Sci U S A. 2015;112(22):7009-14.
22. Campbell PL, Duda RL, Nassur J, Conway JF, Huet A. Mobile Loops and Electrostatic Interactions Maintain the Flexible Tail Tube of Bacteriophage Lambda. J Mol Biol. 2020;432(2):384-95.
23. Sciara G, Bebeacua C, Bron P, Tremblay D, Ortiz-Lombardia M, Lichiere J, et al. Structure of lactococcal phage p2 baseplate and its mechanism of activation. P Natl Acad Sci USA. 2010;107(15):6852-7.
24. Veesler D, Spinelli S, Mahony J, Lichiere J, Blangy S, Bricogne G, et al. Structure of the phage TP901-1 1.8 MDa baseplate suggests an alternative host adhesion mechanism. P Natl Acad Sci USA. 2012;109(23):8954-8.
25. Orlova EV, Gowen B, Droge A, Stiege A, Weise F, Lurz R, et al. Structure of a viral DNA gatekeeper at 10 A resolution by cryo-electron microscopy. Embo J. 2003;22(6):1255-62.
26. Linares R, Arnaud CA, Effantin G, Darnault C, Epalle NH, Boeri Erba E, et al. Structural basis of bacteriophage T5 infection trigger and E. coli cell wall perforation. Sci Adv. 2023;9(12):eade9674.
27. Bardy P, Fuzik T, Hrebik D, Pantucek R, Thomas Beatty J, Plevka P. Structure and mechanism of DNA delivery of a gene transfer agent. Nat Commun. 2020;11(1).
28. Quentin D, Ahmad S, Shanthamoorthy P, Mougous JD, Whitney JC, Raunser S. Mechanism of loading and translocation of type VI secretion system effector Tse6. Nat Microbiol. 2018;3(10):1142-52.
29. Spinola-Amilibia M, Davo-Siguero I, Ruiz FM, Santillana E, Medrano FJ, Romero A. The structure of VgrG1 from Pseudomonas aeruginosa, the needle tip of the bacterial type VI secretion system. Acta Crystallogr D Struct Biol. 2016;72(Pt 1):22-33.
30. Casjens SR, Hendrix RW. Bacteriophage lambda: Early pioneer and still relevant. Virology. 2015;479:310-30.
31. Catalano CE. Bacteriophage lambda: The path from biology to theranostic agent. Wires Nanomed Nanobi. 2018;10(5).
32. Lander GC, Evilevitch A, Jeembaeva M, Potter CS, Carragher B, Johnson JE. Bacteriophage lambda stabilization by auxiliary protein gpD: Timing, location, and mechanism of attachment determined by cryo-EM. Structure. 2008;16(9):1399-406.
33. Rao VB, Fokine A, Fang QL. The remarkable viral portal vertex: structure and a plausible model for mechanism. Curr Opin Virol. 2021;51:65-73.
34. Rajagopala SV, Casjens S, Uetz P. The protein interaction map of bacteriophage lambda. Bmc Microbiol. 2011;11.
35. Hendrix RW, Duda RL. Bacteriophage lambda PaPa: not the mother of all lambda phages. Science. 1992;258(5085):1145-8.
36. Wang C, Zeng JW, Wang JW. Structural basis of bacteriophage lambda capsid maturation. Structure. 2022;30(4).
37. Liu HR, Cheng LP. Cryo-EM shows the polymerase structures and a nonspooled genome within a dsRNA virus. Science. 2015;349(6254):1347-50.
38. Chen WY, Xiao H, Wang L, Wang XR, Tan ZX, Han Z, et al. Structural changes in bacteriophage T7 upon receptor-induced genome ejection. P Natl Acad Sci USA. 2021;118(37).

39. Prevelige PE, Cortines JR. Phage assembly and the special role of the portal protein. Curr Opin Virol. 2018;31:66-73.
40. Fang Q, Tang WC, Tao P, Mahalingam M, Fokine A, Rossmann MG, et al. Structural morphing in a symmetry-mismatched viral vertex. Nat Commun. 2020;11(1):1713.
41. Krissinel E, Henrick K. Inference of macromolecular assemblies from crystalline state. J Mol Biol. 2007;372(3):774-97.
42. Maxwell KL, Yee AA, Booth V, Arrowsmith CH, Gold M, Davidson AR. The solution structure of bacteriophage lambda protein W, a small morphogenetic protein possessing a novel fold. J Mol Biol. 2001;308(1):9-14.
43. Maxwell KL, Yee AA, Arrowsmith CH, Gold M, Davidson AR. The solution structure of the bacteriophage lambda head-tail joining protein, gpFII. J Mol Biol. 2002;318(5):1395-404.
44. Zimmermann L, Stephens A, Nam SZ, Rau D, Kubler J, Lozajic M, et al. A Completely Reimplemented MPI Bioinformatics Toolkit with a New HHpred Server at its Core. J Mol Biol. 2018;430(15):2237-43.
45. Zinke M, Schroder GF, Lange A. Major tail proteins of bacteriophages of the order Caudovirales. J Biol Chem. 2022;298(1):101472.
46. Katsura I. Determination of bacteriophage lambda tail length by a protein ruler. Nature. 1987;327(6117):73-5.
47. Altschul SF, Madden TL, Schaffer AA, Zhang J, Zhang Z, Miller W, et al. Gapped BLAST and PSI-BLAST: a new generation of protein database search programs. Nucleic Acids Res. 1997;25(17):3389-402.
48. Fraser JS, Yu Z, Maxwell KL, Davidson AR. Ig-like domains on bacteriophages: a tale of promiscuity and deceit. J Mol Biol. 2006;359(2):496-507.
49. Tam W, Pell LG, Bona D, Tsai A, Dai XX, Edwards AM, et al. Tail tip proteins related to bacteriophage lambda gpL coordinate an iron-sulfur cluster. J Mol Biol. 2013;425(14):2450-62.
50. Buchan DWA, Minneci F, Nugent TCO, Bryson K, Jones DT. Scalable web services for the PSIPRED Protein Analysis Workbench. Nucleic Acids Research. 2013;41(W1):W349-W57.
51. Katsura I, Kuhl PW. A regulator protein for the length determination of bacteriophage lambda tail. J Supramol Struct. 1974;2(2-4):239-53.
52. Xu J, Hendrix RW, Duda RL. Chaperone-Protein Interactions That Mediate Assembly of the Bacteriophage Lambda Tail to the Correct Length. J Mol Biol. 2014;426(5):1004-18.
53. Mahony J, Alqarni M, Stockdale S, Spinelli S, Feyereisen M, Cambillau C, et al. Functional and structural dissection of the tape measure protein of lactococcal phage TP901-1. Sci Rep-Uk. 2016;6.
54. Cumby N, Reimer K, Mengin-Lecreulx D, Davidson AR, Maxwell KL. The phage tail tape measure protein, an inner membrane protein and a periplasmic chaperone play connected roles in the genome injection process of E. coli phage HK97. Mol Microbiol. 2015;96(3):437-47.
55. Jumper J, Evans R, Pritzel A, Green T, Figurnov M, Ronneberger O, et al. Highly accurate protein structure prediction with AlphaFold. Nature. 2021;596(7873):583-9.
56. Wang J, Hofnung M, Charbit A. The C-terminal portion of the tail fiber protein of bacteriophage lambda is responsible for binding to LamB, its receptor at the surface of Escherichia coli K-12. J Bacteriol. 2000;182(2):508-12.
57. Roessner CA, Struck DK, Ihler GM. Morphology of complexes formed between bacteriophage lambda and structures containing the lambda receptor. J Bacteriol. 1983;153(3):1528-34.
58. Roessner CA, Ihler GM. Proteinase sensitivity of bacteriophage lambda tail proteins gpJ and pH in complexes with the lambda receptor. J Bacteriol. 1984;157(1):165-70.
59. Roessner CA, Ihler GM. Formation of transmembrane channels in liposomes during injection of

lambda DNA. J Biol Chem. 1986;261(1):386-90.

60. Hu B, Margolin W, Molineux IJ, Liu J. The bacteriophage t7 virion undergoes extensive structural remodeling during infection. Science. 2013;339(6119):576-9.

61. Zhang K. Gctf: Real-time CTF determination and correction. J Struct Biol. 2016;193(1):1-12.

62. Scheres SHW. RELION: Implementation of a Bayesian approach to cryo-EM structure determination. J Struct Biol. 2012;180(3):519-30.

63. Kivioja T, Ravantti J, Verkhovsky A, Ukkonen E, Bamford D. Local average intensity-based method for identifying spherical particles in electron micrographs. J Struct Biol. 2000;131(2):126-34.

64. Li X, Zhou N, Chen W, Zhu B, Wang X, Xu B, et al. Near-Atomic Resolution Structure Determination of a Cypovirus Capsid and Polymerase Complex Using Cryo-EM at 200kV. J Mol Biol. 2017;429(1):79-87.

65. Thuman-Commike PA, Chiu W. Improved common line-based icosahedral particle image orientation estimation algorithms. Ultramicroscopy. 1997;68(4):231-55.

66. Fuller SD, Butcher SJ, Cheng RH, Baker TS. Three-dimensional reconstruction of icosahedral particles--the uncommon line. J Struct Biol. 1996;116(1):48-55.

67. Ludtke SJ, Baldwin PR, Chiu W. EMAN: semiautomated software for high-resolution single-particle reconstructions. J Struct Biol. 1999;128(1):82-97.

68. Emsley P, Lohkamp B, Scott WG, Cowtan K. Features and development of Coot. Acta Crystallogr D Biol Crystallogr. 2010;66(Pt 4):486-501.

69. Adams PD, Afonine PV, Bunkoczi G, Chen VB, Davis IW, Echols N, et al. PHENIX: a comprehensive Python-based system for macromolecular structure solution. Acta Crystallogr D Biol Crystallogr. 2010;66(Pt 2):213-21.

Supporting information

S1 Fig. Cryo-EM image and Fourier shell correlation curves. (A) Cryo-EM image of mature lambda phage showing its long flexible tail. (B) Estimated structural resolutions of the local reconstructions of the gpB and gpW with an imposed symmetry of 12 folds (black line), the gpFII, gpU, and gpVN with an imposed symmetry of 6 folds (red line), the tail tube (gpVN) with an imposed symmetry of 6 folds (blue line), the tail tip complex with an imposed symmetry of 3 folds (magenta line), and the portal-capsid without symmetry imposed (cyan line).

S2 Fig. Reconstruction of the icosahedral head. (A) Overall view of the icosahedral head structure. Seven copies of the major capsid protein gpE arranged in an asymmetric unit are shown in purple, green, hot pink, cornflower blue, cyan, dark green, and magenta and six trimers of the cement protein gpD surrounding the asymmetric unit are shown in orange. (B) Zoomed-in view of the asymmetric unit in panel A. The 5-, 3-, and 2-fold axes are labeled. (C-D) Density maps (grey) of the major capsid protein gpE and the trimer of the cement protein gpD superimposed on their atomic models (ribbons).

S3 Fig. Density maps (transparent or mesh) of proteins gpB (A), gpW (B), gpFII (C), gpU (D), gpVN (E), gpM (F), gpL (G), and gpJ (H) superimposed on their atomic models.

S4 Fig. Comparison among the portal proteins of lambda, SPP1 (Protein Data Bank [PDB] ID: 7Z4W), T7 (PDB ID: 7BOU), and T4 (PDB ID: 3JA7). The RMSDs between the portal proteins of lambda and SPP1, T7, and T4 are 2.52, 2.48, and 2.03 Å, respectively.

S5 Fig. Portal–capsid interactions in the symmetry-mismatched binding interface. (A) A schematic representation of the portal and the surrounding capsomers. The 12 portal gpB subunits (identified as 1 to 12) are in orange and cyan. The five M subunits (M1 to M5) and five N subunits (N1 to N5) of the major capsid protein gpE are in magenta and dark blue. The red arrowheads indicate salt bridges between the portal and coat subunits. (B) Salt bridges between the portal and coat subunits. The atomic models (ribbon) are superimposed on their density maps (transparent). (C) Superimposition of the twelve portal subunits revealed the structural morphing loop (residues 208-218) in gpB.

S6 Fig. Structural comparison among lambda, T4, 80α, SPP1, RcGTA, and T5 in terms of the N-termini of tail tube proteins (left) and the distal tail proteins (right). All models are shown in rainbow colors, ranging from blue at the N-termini to red at the C-termini, and the redundant parts are shown in grey.

S7 Fig. Structural comparison between lambda gpJ, gpL, and T5 pb3 (baseplate hub protein). The color code for domains is identical to that in Fig 7G. (A) Structure of gpJ. (B) Structure of T5 BHP pb3. (C) Structure of gpL. (D) Complex of a copy of gpJ, gpL, and gpI in the lambda tail tip. (E) Structural comparison between the C-terminal domain of the lambda gpL (residues154-232) and the N-terminus of the T5 pb3 HDII-insertion domain shows that they are is topologically similar.

S8 Fig. Secondary structure of tape measure protein (gpH) predicted using the PSIPRED (version 4.0) tool.

S9 Fig. Gene arrangement of the counterpart proteins of T5, lambda, and RcGTA. Genes encoding proteins of homologous domains are shown in the same pattern and color. The regions in white are unstructured.

S10 Fig. Comparison between our gpJ structure and predicted gpJ structure. (A) Structure of the gpJ structure predicted by AlphaFold. The color code for domains is identical to that in Fig 7G except that the central fiber and receptor binding domains are in pink and conflower blue,

respectively. (B) Comparison of our gpJ structure (cyan) and predicted gpJ structure (magenta) shows the conformational change between them.

S11 Fig. Ultrathin sections images of the lambda infected E. coli cells. The scale bars represent 200 nm.

S12 Fig. Tail tip reconstructed using RELION. First, we manually selected a total of 70,745 tail tip particles (A, B) and performed 2D classification to exclude irrelevant images (C). Then, using a randomly generated cylinder density as the initial model (D), we selected a total of 58,215 particles from the 2D classifications to perform 3D classification with a C3 symmetry and obtained a total of six types of low-resolution structures (E). Third, we re-extracted tail tip images (F) according to the centers of each type. Fourth, we performed 2D classification (G) and 3D auto-refinement with a C3 symmetry to obtain a density map of the tail tip complex at a resolution of 3.72 Å (H). Finally, we refined the contrast transfer function to improve the resolution of the tail tip structure to 3.44 Å (I).

S1 Table. Modeled proteins of the siphophage lambda.

S2 Table. Detected proteins of the siphophage lambda by mass spectrometry.

S3 Table. Results of the HHpred analysis of the tail protein sequences of lambda phage. For each protein, the most relevant hits are shown with the matched residues, Protein Data Bank ID, chain identifier, HHpred probability (%), E-value, and percent sequence identity in the matched regions.

S4 Table. Refinement and model statistics.

S1 Appendix. The wwwPDB EM validation reports of lambda portal-capsid, portal-adaptor, neck, tail tube, and tail tip complex.

Fig 1. Symmetry-mismatch reconstruction of the intact lambda virion.

Click here to access/download;Figure;Fig 1.tif

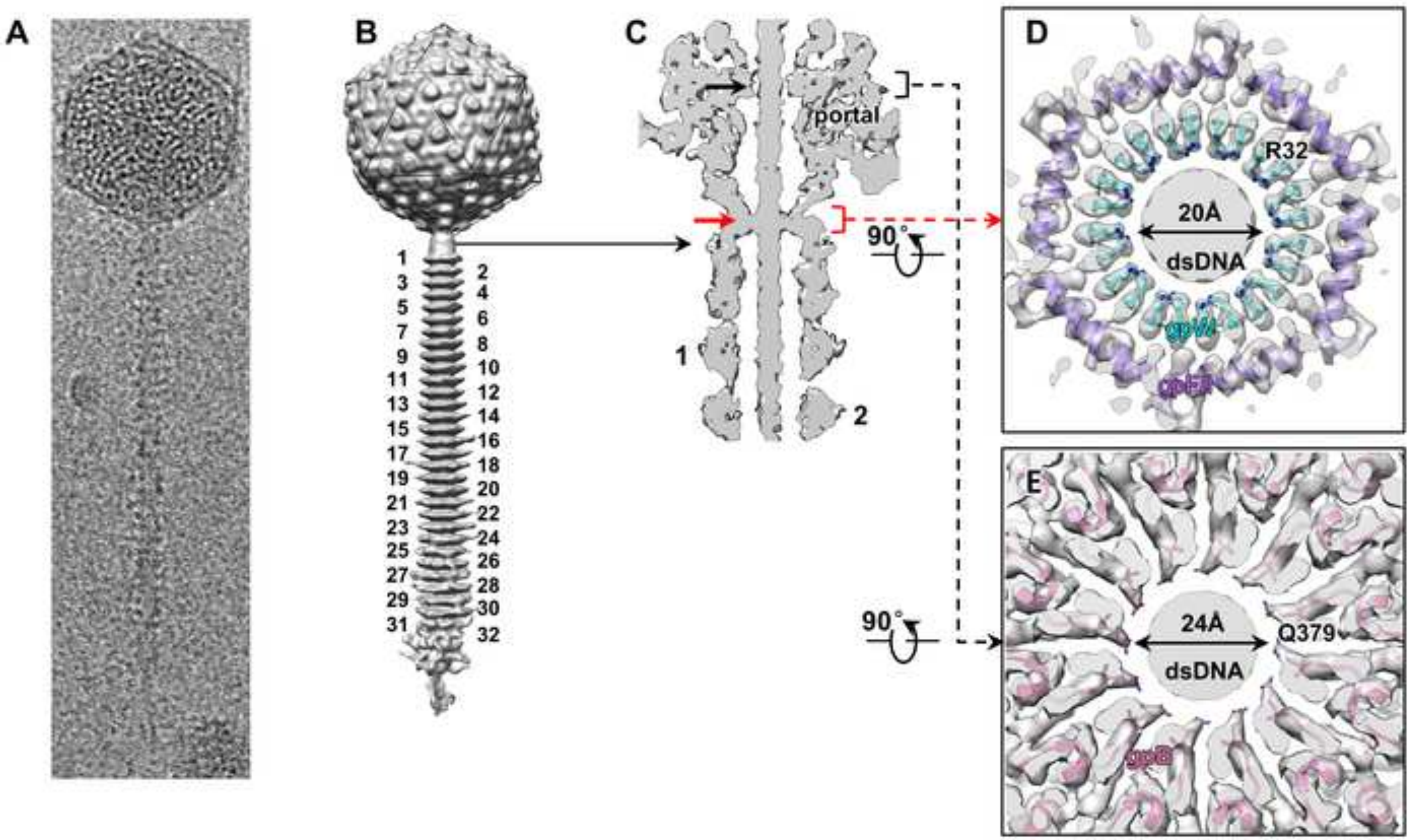

Fig 2. Structure of the lambda virion.

Click here to access/download;Figure;Fig 2.tif

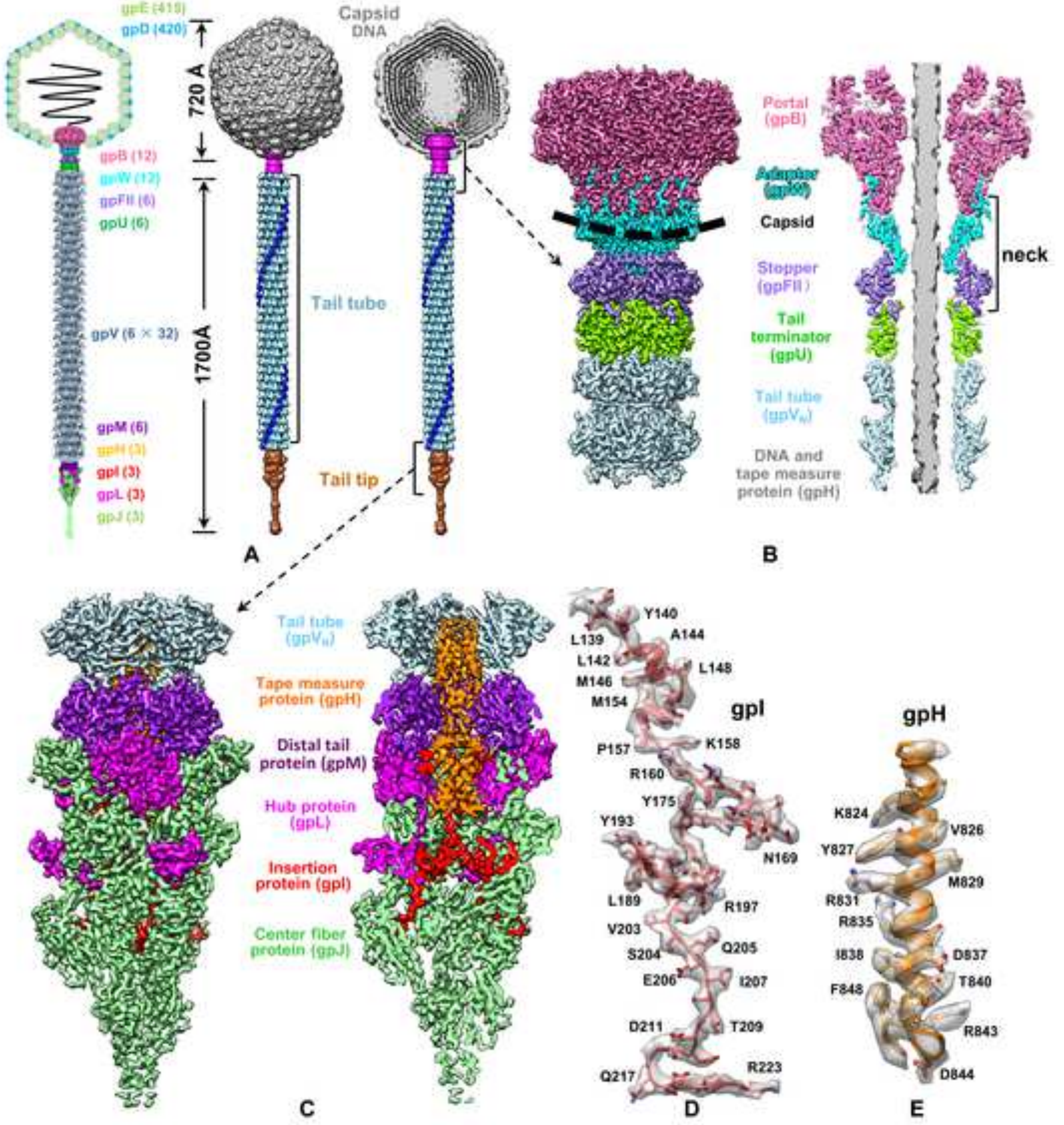

Fig 3. Structure of the portal and neck. Click here to access/download;Figure;Fig 3.tif

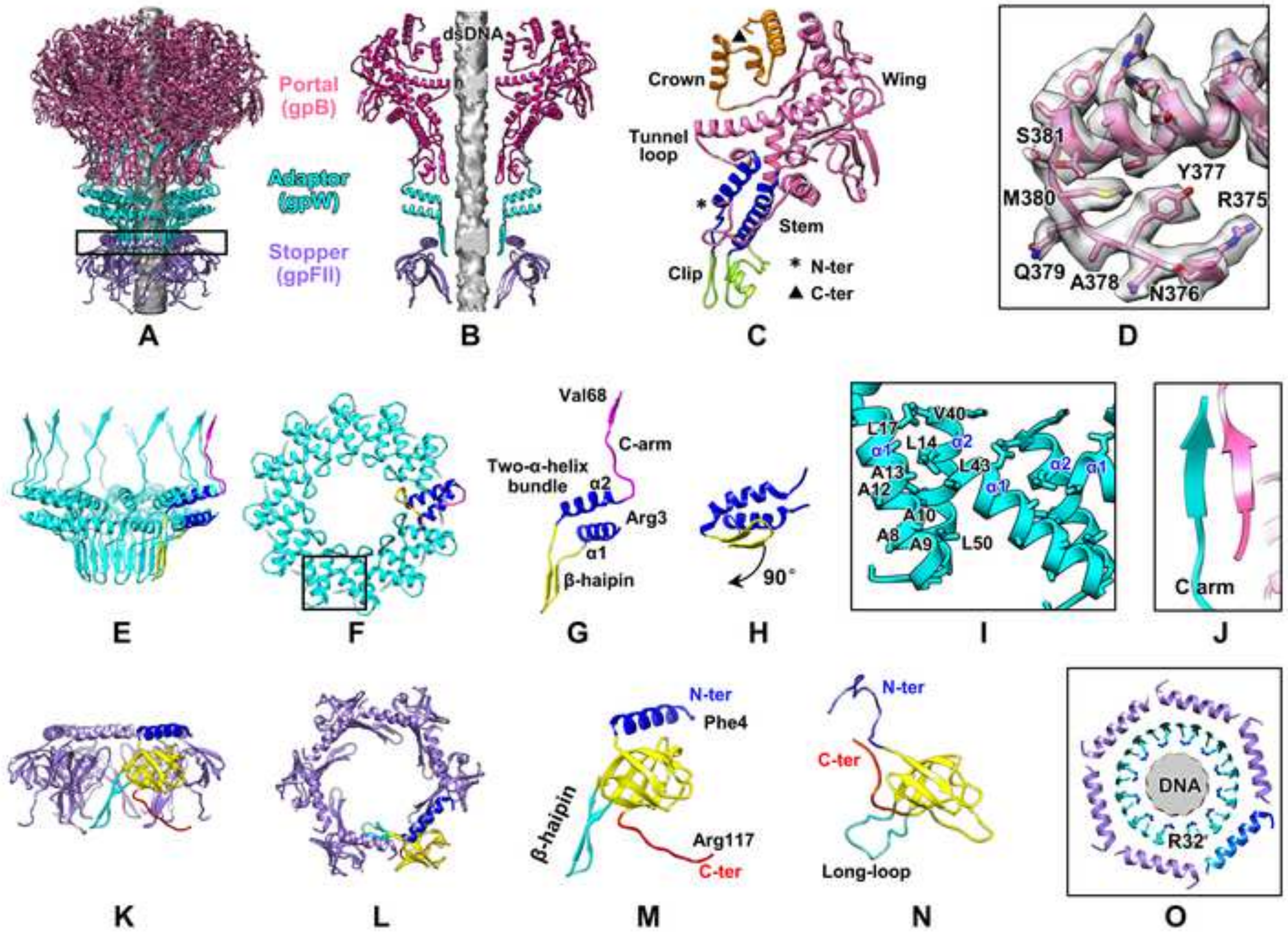

Fig 4. Electrostatic potential surfaces of the interacting regions of two adjacent rings.

Click here to access/download;Figure;Fig 4.tif

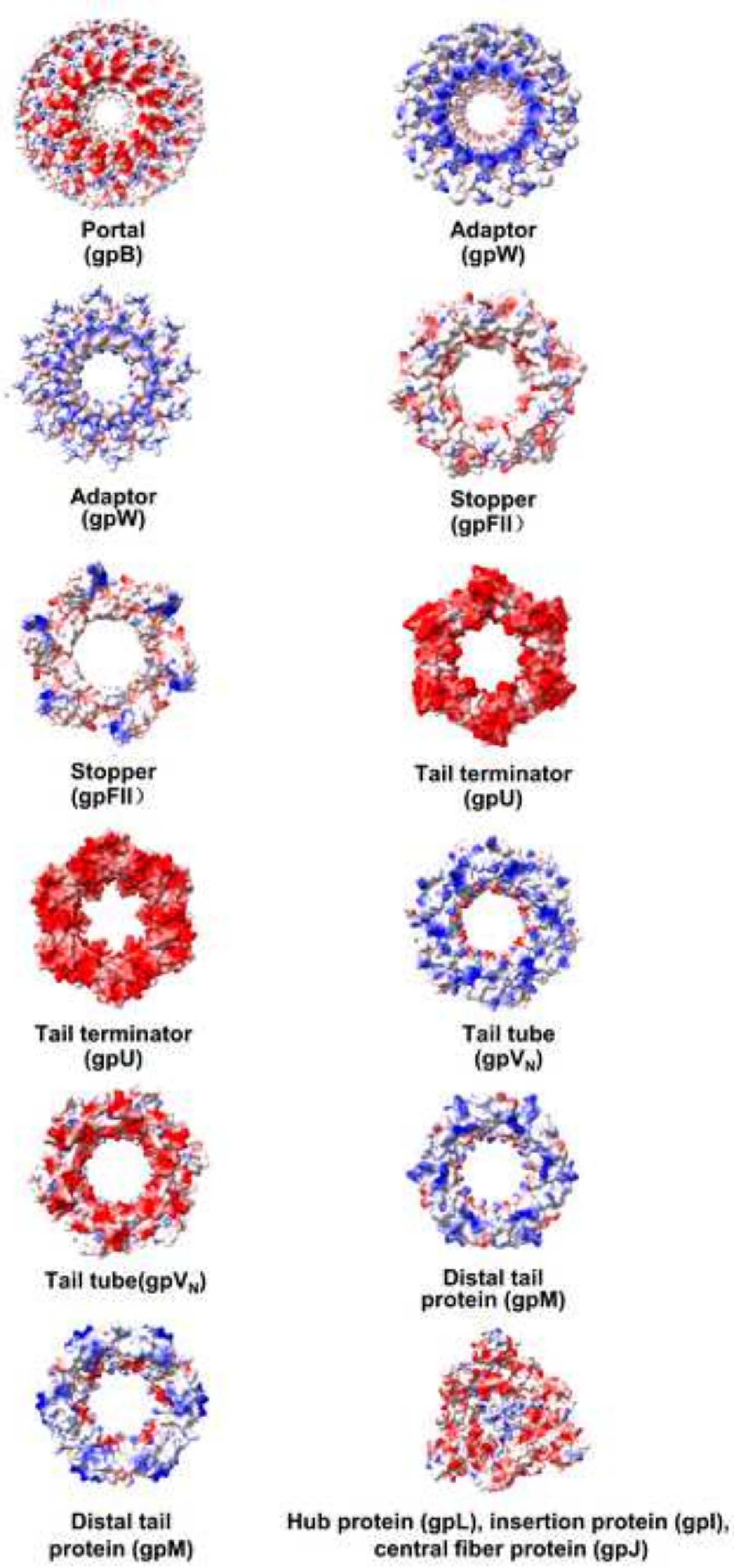

Fig 5. Structure of the tail tube.

Click here to access/download;Figure;Fig 5.tif

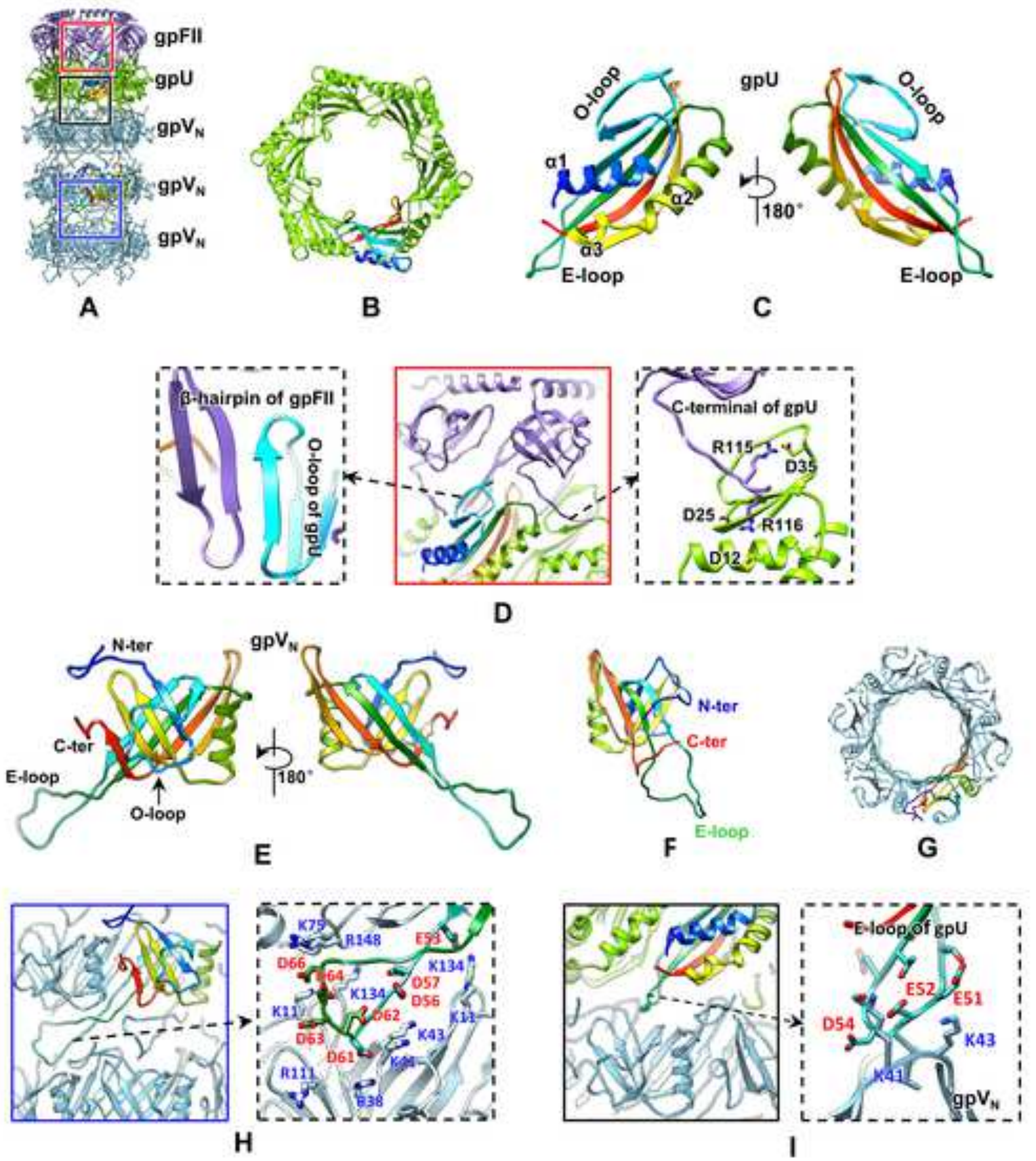

Fig 6. Density map of the tail tube.

Click here to access/download;Figure;Fig 6.tif

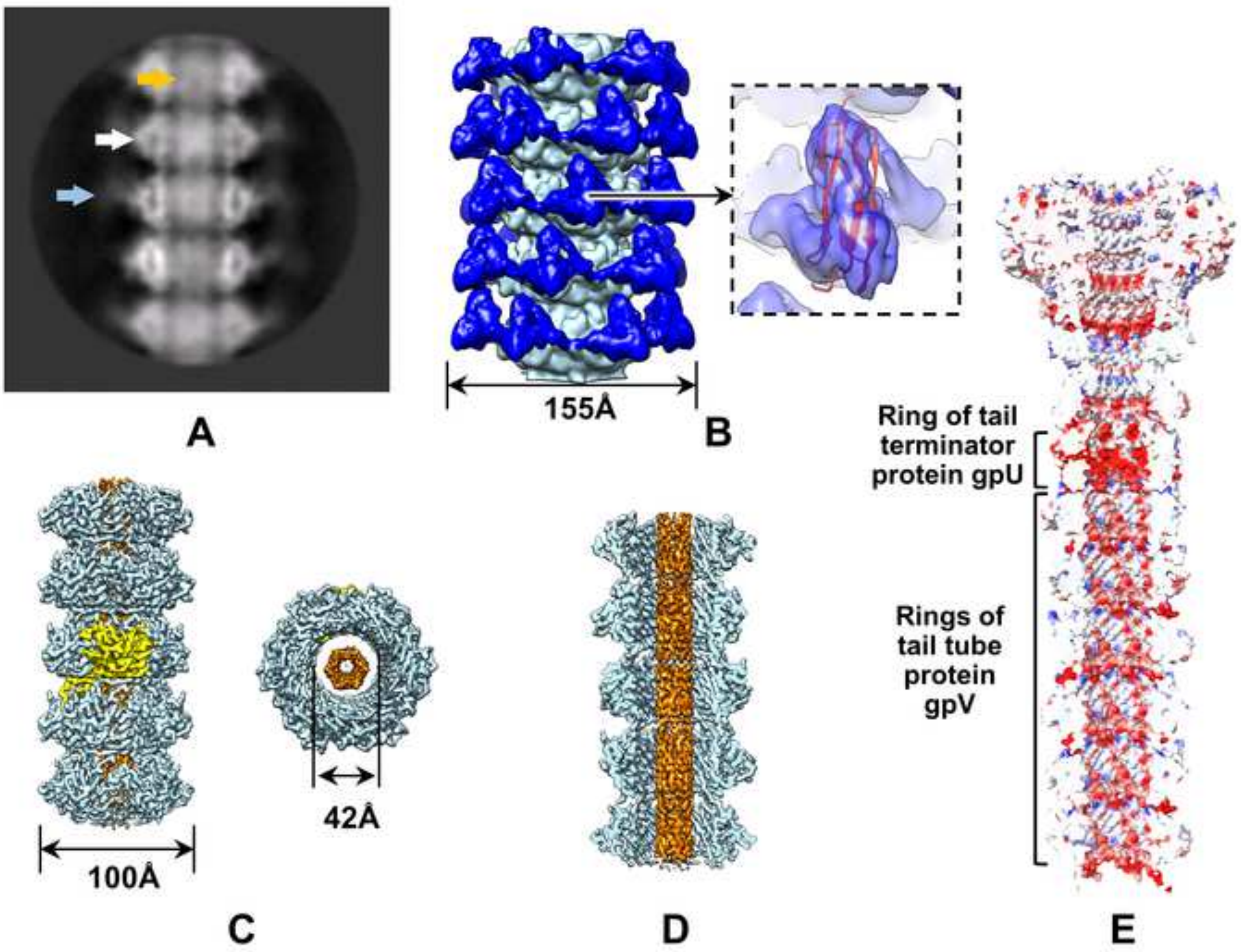

Fig 7. Structure of the tail tip.

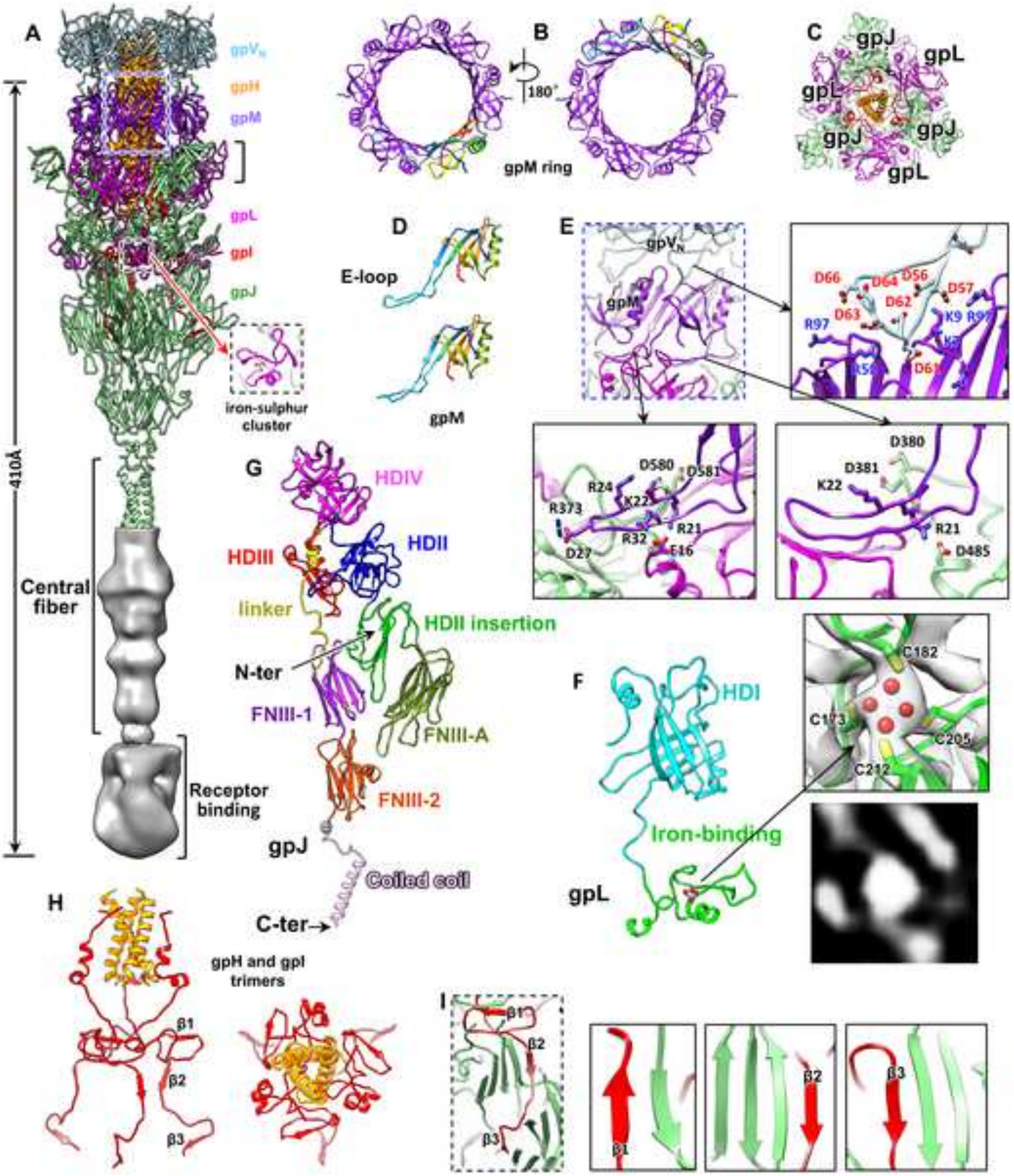

Fig 8. Structural comparison between lambda, other phages, and tail-like machines.

Click here to access/download;Figure;Fig 8.tif

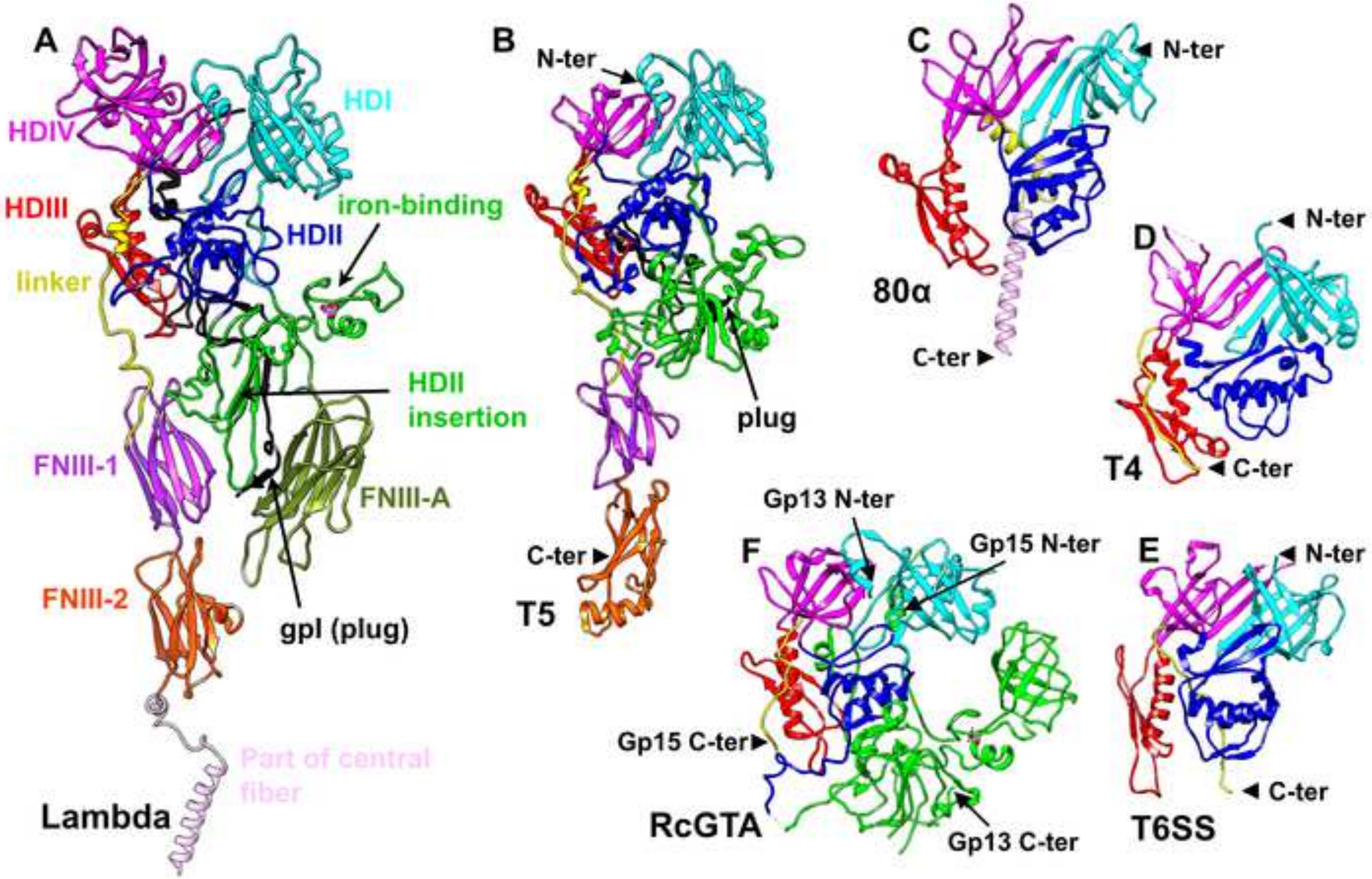

S1 Fig. Cryo-EM image and Fourier shell correlation curves.

Click here to access/download
Supporting Information
S1 Fig.tif

S2 Fig. Reconstruction of the icosahedral head.

Click here to access/download
Supporting Information
S2 Fig.tif

S3 Fig. Density maps (transparent or mesh) of proteins gpB (A), gpW (B), gpFII (C), gpU (D), gpVN (E), gpM (F), gpL (G), and gpJ

Click here to access/download
Supporting Information
S3 Fig.tif

S4 Fig. Comparison among the portal proteins of lambda, SPP1, T7, and T4.

Click here to access/download
Supporting Information
S4 Fig.tif

S5 Fig. Portal–capsid interactions in the symmetry-mismatched binding interface.

Click here to access/download
Supporting Information
S5 Fig.tif

S6 Fig. Structural comparison among lambda, T4, 80α, SPP1, RcGTA, and T5 in terms of the N-termini of tail tube proteins (left)

Click here to access/download
Supporting Information
S6 Fig.tif

S7 Fig. Structural comparison between lambda gpJ, gpL, and T5 pb3 (baseplate hub protein).

Click here to access/download
Supporting Information
S7 Fig.tif

S8 Fig. Secondary structure of tape measure protein (gpH) predicted using the PSIPRED (version 4.0) tool.

Click here to access/download
Supporting Information
S8 Fig.tif

S9 Fig. Gene arrangement of the counterpart proteins of T5, lambda, and RcGTA.

Click here to access/download
Supporting Information
S9 Fig.tif

S10 Fig. Comparison between our gpJ structure and predicted gpJ structure.

Click here to access/download
Supporting Information
S10 Fig.tif

S11 Fig. Ultrathin sections images of the lambda infected E. coli cells.

Click here to access/download
Supporting Information
S11 Fig.tif

S12 Fig. Tail tip reconstructed using RELION.

Click here to access/download
Supporting Information
S12 Fig.tif

S1 Table. Modeled proteins of the siphophage lambda.

Click here to access/download
Supporting Information
S1 Table.pdf

S2 Table. Detected proteins of the siphophage lambda by mass spectrometry.

Click here to access/download
Supporting Information
S2 Table.pdf

S3 Table. Results of the HHpred analysis of the tail protein sequences of lambda phage.

Click here to access/download
Supporting Information
S3 Table.pdf

S4 Table. Refinement and model statistics.

Click here to access/download
Supporting Information
S4 Table.pdf

S1 Appendix. The wwwPDB EM validation reports of lambda portal-capsid, portal-adaptor, neck, tail tube, and tail tip complex.

Click here to access/download

Supporting Information - Compressed/ZIP File Archive

PDB Validation Report.zip

Symmetry-mismatch structure of the bacteriophage lambda

~~In situ structure of the connector and tail of a Siphophage~~

Hao Xiao[1,2,#], Le Tan[1,#], Zhixue Tan[1,#], Yewei Zhang[1], Wenyuan Chen[1], Xiaowu Li[3], Jingdong Song[2,*], Lingpeng Cheng[1,*], and Hongrong Liu[1,*]

[1] Institute of Interdisciplinary Studies, Key Laboratory for Matter Microstructure and Function of Hunan Province, Key Laboratory of Low-dimensional Quantum Structures and Quantum Control, School of Physics and Electronics, Hunan Normal University, Changsha, China.

[2] National Key Laboratory of Intelligent Tracking and Forecasting for Infectious Diseases~~State Key Laboratory of Infectious Disease Prevention and Control~~, National Institute for Viral Disease Control and Prevention, Chinese Center for Disease Control and Prevention, Beijing, China.

[3] School of Electronics and Information Engineering, Hunan University of Science and Engineering, Yongzhou, China.

# These authors contributed equally to this work.

*Correspondence to: songjd@ivdc.chinacdc.cn, lingpengcheng@hunnu.edu.cn, and hrliu@hunnu.edu.cn

Data Availability Statement: The electron density maps and atomic coordinates have been deposited in the EM Data Bank and Protein Data Bank under accession code~~ ~~s EMD-36844, EMD-36845, EMD-36846, EMD-36847, EMD-36848, 8K35, 8K36, 8K37, 8K38 and 8K39~~EMD-xxxx, EMD-xxxx, EMD-xxxx, EMD-xxxx, xxxx, and xxxx~~.

Competing interests: The authors declare no conflict of interest.

Abstract

Siphophages have a long, flexible, and noncontractile tail~~, which~~ that connects to the capsid through a ~~head-to-tail connector~~neck. The phage tail is essential for host cell recognition and virus–host cell interactions; moreover, it serves as a channel for genome delivery during infection. However, the in situ high-resolution structure of the ~~connector~~neck-tail complex of siphophages remains unknown. Here we present the in situ structure of the ~~connector~~neck-tail complex of the siphophage lambda wild type, which lacks side tail fiber ~~at a near-atomic resolution~~. The ~~connector~~neck-tail complex comprises a channel formed by stacked 12-fold and 6-fold symmetrical rings and a 3-fold symmetrical tip. The interactions among DNA and a total of 246 tail protein molecules responsible for ~~connector~~neck ~~–~~tail assembly and stabilization have been characterized. The DNA in the neck is clamped by the adaptor protein rather than the stopper protein.~~Lambda DNA penetrates into the tail tube from the connector after tail assembly, which ensures a stable interaction between DNA and tape measure protein. The coiled coil of tape measure protein gpH and the trimer of insertion protein gpI form a plug to prevent the early release of the gpH oligomer and DNA.~~ Structural comparisons of the tail tips, the most diversified region across the lambda and other long-tailed phages or tail-like machines suggest that their tail tip contains conserved domains, which facilitate tail assembly, receptor binding, cell adsorption, and DNA retaining/releasing. These domains are distributed in different tail tip proteins in different phages or tail-like machines. The side tail fibers are not required for the perpendicular adsorption of lambda.~~Our structure provides insights into the assembly and infection mechanisms of the Siphophage.~~

Introduction

Bacteriophages are the most abundant and diverse biological entities in the biosphere [1]. The majority of known phages belong to the order *Caudovirales* and contain a tail attached to the pentameric vertex of the icosahedral capsid ~~shell~~ (head) through a portal or through a portal in complex with ~~head completion~~neck (connector) proteins ~~(head-to-tail connector complex) embedded in the vertex~~ [2]. According to the tail morphology, bacteriophages are divided into three families: Podoviridae (short tail), Myoviridae (long contractile straight tail), and Siphoviridae (long noncontractile flexible tail) [3]. ~~During the packaging of the genome, double-stranded DNA (dsDNA) is packaged into the head through the portal with an ATP-dependent terminase complex motor [4]. When the head is full, the terminase complex disassociates from the portal, the genome is retained in the head by conformational change of the portal [5]or by assembly of the head completion proteins. The attachment of an assembled phage tail to the portal or the connector completes the phage assembly. According to the tail morphology, bacteriophages are divided into three families: Podoviridae (short tail), Myoviridae (long contractile straight tail), and Siphoviridae (long noncontractile flexible tail) [3].~~ The phage tail plays key roles in host cell recognition and interaction. In addition, the tail serves as a channel for viral genome delivery during infection; therefore, the tail structure is an extremely interesting study subject in terms of its assembly, host recognition, and cell wall perforation mechanisms [4].

The tail structure of phages belonging to Caudovirales phage has been studied extensively. The tails of Podophages and Myophages are straight; therefore they are suitable for high-resolution structural analyses. The tail structures of these phages that have been reported at ~~near-atomic~~ high resolutions. These structures include: the isolated portal-tail complex of Podophage T7 [5] and the entire tail of Podophages T7, Pam1, sf6, and phi29 [6-9], the baseplate with two tail tube rings and sheath proteins of myophage T4 [10], and the tail tube of the Myophage T4 [11]. However, the resolution of the entire tail structure of siphophages is difficult to improve because of its intrinsic flexibility. Some monomeric tail proteins and isolated tail complexes have been resolved from subnanometer to atomic resolutions. For example, the structures of the monomeric siphophage tail and connector proteins have been determined using nuclear magnetic resonance (NMR) spectroscopy and X-ray crystallography

[12-17]. In addition, the structures of the recombinant tail tubes, tail tips, and isolated tails of Siphophages have been determined ~~at near-atomic and lower resolutions~~ by using X-ray crystallography and using cryo-electron microscopy (cryo-EM) focusing on relatively rigid regions of tail tubes, tail tips, or ~~head-to-tail~~ connectors [18-26]. The ~~S~~structures of a gene transfer agent (GTA, an evolutionarily related phage-like particle) [27] and type VI secretion systems (T6SS, phage tail-like bacterial nanomachines) [28, 29] have also been reported. However, the in situ high-resolution structures of the~~entire connector~~ neck, tail tube, and tail tip ~~complex~~ in the Siphoviridae phages remain~~s~~ unknown, limiting ~~our~~ the understanding of the assembly and infection mechanisms of ~~S~~siphophages.

The temperate phage lambda, which infects the bacterium *Escherichia coli*, belongs to Siphoviridae. The lambda phage is used as a model system and is important in the attainment of the current understanding of the sophisticated and complex mechanisms by which the expression of genesis is controlled, the understanding of DNA virus assembly, and the understanding of the molecular nature of lysogeny~~It is used as a model system in studies on the assembly of DNA viruses and the molecular nature of lysogeny~~ [30]. Moreover, the lambda phage has various therapeutic and diagnostic applications [31]. The virion of the lambda phage comprises an icosahedral head and a long flexible tail [2]. The head~~, which is formed by~~ consists of a total of 415 ~~and 420~~ copies of coat protein gpE, ~~and~~ 420 copies of cement protein gpD, 12 copies of portal protein gpB~~ respectively~~, ~~contains~~ and~~a~~ 48.5 kbp double-stranded DNA (dsDNA) [32]. The ~~One vertex of the icosahedron is replaced by a~~ dodecameric portal is located at a unique icosahedral vertex~~, which~~ and facilitates the transport of DNA in and out of the head during genome packaging and delivery [33]. The tail, which comprises the tail tube and tail~~,~~ tip, ~~and side fibers,~~ connects with the head through the neck~~connector~~. The ~~connector~~ portal, neck, and tail tube form a channel, in which the end of the dsDNA and an oligomer of tape measure protein are located [4, 34].

Here, we present the cryo-EM structure of the phage~~entire~~ lambda, which is a laboratory-adapted strain and is called "lambda wild type" [35]~~phage at near-atomic resolution.~~~~,~~ Our lambda structure allow~~ing~~s the head proteins and most proteins in the ~~head-to-tail connector~~portal, neck, tail tube, and tail tip to be modeled in atomic detail. These proteins include the head proteins gpE and gpD, the ~~connector~~ portal protein~~s~~ gpB, the neck proteins

gpW, and gpFII, the tail tube proteins gpU and gpV_N, and tail tip proteins gpM, gpI, gpL, ~~and~~ gpJ, and gpH (S1 Table). Our structure shows interactions among these 246 tail protein molecules responsible for the tail assembly, receptor binding, cell adsorption, and DNA retaining/releasing~~assembly and stabilization.~~ ~~, and reveals mechanisms of DNA retaining and releasing in this long and flexible tail machine.~~ Structural comparisons between the tail tips of lambda and other long-tailed phages or tail-like machines indicate that the major domains in the tail tips are structurally conserved, although they are distributed in different tail tip proteins in different phages or tail-like machines.

Results and Discussion

Overall structure of lambda phage

Cryo-EM image shows that the mature virion of the lambda phage comprises an icosahedral head with a long and flexible tail (S1A Fig). The head structure was reconstructed at a resolution of 3.5-Å by imposing the icosahedral symmetry (S1B and S2~~A-2D~~ Figs). The head comprises a DNA-containing capsid shell formed by the coat protein gpE and decorated by the trimers of the cement protein gpD. Our structure of the head is essentially identical to that reported by a recent study on the lambda head [36]~~ except that our structure has higher resolution~~. We selected virion particles with a straight tail, and reconstructed the entire virion structure at a resolution of ~20 Å (Fig ~~S3~~1A and ~~3~~B~~ Fig~~) using our symmetry-mismatch reconstruction method [6, 37] (The software package can be downloaded from https://cryoem.hunnu.edu.cn). To improve the resolution of the tail structure, we used our local refinement and reconstruction method [6, 38] focusing on the portal, ~~connector~~neck, tail tube, and tail tip. A structure of the ~~connector~~portal–neck–tail complex at ~3.5 Å resolution was obtained by merging locally reconstructed structures (Fig ~~1~~2). The tail, which comprises ~~32 rings~~a long tail tube and a distal tail tip, attaches to the head through the ~~head-to-tail connector~~ neck (~~S3B~~ Fig 2A). The ~~connector~~ portal, neck and tail tube form a long channel, through which a rod-like structure passes (Fig ~~1~~2A and B, ~~S3C~~ Fig 1C). We build atomic models (S1 Table) for the ~~connector~~ portal (12-fold rings of the portal protein gpB), neck (including a 6-fold ring of ~~two~~the ~~12-fold rings of the portal protein gpB and the~~ adaptor protein gpW~~,~~ and a 6-fold ring of the stopper protein gpFII), tail tube (including a 6-fold ring of the tail terminal protein gpU

and thirty-two 6-fold rings of the tail tube protein gpV), and tail tip (including a 6-fold ring of the distal tail protein gpM, ~~four~~ 3-fold trimers of the hub protein gpL, insertion protein gpI, ~~megatron~~ central fiber protein gpJ, and an C-terminal α-helix of the tape measure protein gpH~~,~~ ~~respectively~~) (Figs ~~1~~ 2 and ~~2~~3, ~~S4~~ S3 Fig). Our mass spectrometry results (S2 Table) show that the lambda virion consists of proteins gpE, gpD, gpB, gpW, gpFII, gpU, gpV, gpM, gpH, gpL, gpI and gpJ, as well as tail completion protein gpZ and protease protein gpC. The side tail fiber proteins (stf and tfa) and gpK were not identified using mass spectrometry. The reason for the absence of the side tail fiber proteins is that the stf gene is disrupted in the genome of lambda wild type by frameshift mutation [35]. As for gpK, it is speculated that it is a nonstructural protein that is not present in the lambda virion. Among the identified lambda proteins, gpZ and gpC were not resolved in our structures. It is speculated that gpZ may lack symmetry in the tail. Indeed, gpZ homologous protein p143 in the siphophage T5 was observed to present as a monomer on the tail tip [26]. The gpC molecules might be distributed asymmetrically within the head. [37]

Structure of the ~~connector~~portal and neck

~~The head-to-tail connector, which is a funnel-shaped complex occupying 1 of the 12 vertexes of the icosahedral head, provides a platform for tail attachment and facilitates the DNA packaging and ejection. The connector comprises three rings of the portal, adaptor, and stopper proteins (Figs 1B, and 2A, 2B).~~ The portal occupies one of the 12 vertexes of the icosahedral head. It is ~~surrounded~~ formed by 12 copies of gpB (Figs 2A, 2B, 3A, and 3B), which exhibits the canonical portal fold of phages belonging to the order *Caudovirales* (S~~5~~4 Fig) [39]. Excluding 23 N-terminal residues, 20 C-terminal residues, and ~~two~~ a loop~~s~~ (residues ~~213-216, and~~ 303-319), which were missing at the outer surface of the portal, the resolved structure of gpB comprised four domains (Fig ~~2C~~3C): wing (residues 24-249 and 368-452), stem (residues 250-279, 343-367), clip (residues 280-342), and crown (residues 453-511). The tunnel loop, which was previously indicated to clamp dsDNA to avoid leakage during the phage maturation [33], was well resolved (Fig. ~~2C~~3C~~,~~ and 3~~2~~D). The asymmetric reconstruction of the portal and capsid at a resolution of 4.0 Å (S1B Fig) showed the asymmetric interactions between the gpB and gpE subunits, as observed in the structures of phages sf6 and T4 [9, 40]. The portal–capsid interface was analyzed using the PISA server [41], which revealed the salt bridges

between the gpB and gpE subunits (S5A and B Fig). Superimposition of the models of the 12 portal subunits revealed that a loop (residues 208-218) in the gpB wing domain underwent structural morphing to adapt to the portal–coat interactions (S5C Fig).

The neck comprises two rings composed of the adaptor and stopper proteins (Figs 2B, 3A, and 3B). The adaptor ring, which is formed by 12 copies of gpW, is assembled below the portal (Fig ~~1B~~2B). A total of 66 residues (~~3~~4-68) of the 68-residue adaptor protein gpW were resolved. The structure of gpW comprises three domains: a β-hairpin (residues 23-36), a two-α-helix bundle (residues 4-22 and 37-54), and an extended C-arm (residues 55-68) (Fig ~~2E~~3E-~~2~~G, and ~~S4B~~ S3B Fig). The assembly of the adaptor relies on hydrophobic interactions among the subunits of gpW (Fig 3F, 3I). ~~The hydrophobic side chains of gpW include Leu7, Ala8, Ala9, Ala10, Leu14, and Leu17 in α1, and Val40, Leu43, Ile47, and Leu50 in α2 (Fig 2F, 2I).~~ The interactions of the adjacent gpW subunits are further reinforced by interactions between the adjacent β-hairpin domains, which form a β-barrel (Fig ~~2E~~3E). Compared with the NMR structure of a gpW monomer in solution [42] the β-hairpin in our gpW structure undergoes a 90° rotation, from parallel to vertical with respect to the two-α-helix bundle domain (Fig ~~2G~~3G~~,~~ ~~2~~ and H), which facilitates the formation of the β-barrel structure. The assembly of the adaptor on the portal mainly relies on the electrostatic interactions between the electropositive adaptor top and the electronegative portal bottom (~~S6~~ Fig 4). In addition, the C-arm, which is absent from the NMR structure, interacts with residues 320-324 of the portal wing through β-sheet augmentation (Fig ~~2A~~3A and~~,~~ ~~2~~J).

Six copies of the stopper protein gpFII are assembled into a 6-fold ring connecting the adaptor (Figs ~~1B~~2B, ~~2~~3K, and 3L). We resolved residues 4-117 of the 117-residue protein gpFII (Fig ~~2K~~3K-~~2~~M, and ~~S4C~~ S3C Fig). The gpFII structure comprises four domains (Fig ~~2M~~3M): an N-terminal α-helix (residues 4-24), a β-sandwich (residues 25-41 and 63-107), a β-hairpin (residues 46-62) inserted in the β-sandwich, and a C-terminal loop (residues 108-117). A comparison of our gpFII structure with the NMR structure of gpFII monomer in solution [43] revealed that the structure of gpFII monomer in solution undergoes notable conformational changes (Fig ~~2M~~3M~~, 2~~ and N) to reach the assembled state. The N-terminal loop becomes the α-helix domain. Six copies of the α-helix domains form a ring, into which the β-barrel of the adaptor rests (Fig 3~~2~~A, B, and O). Two other conformational changes are involved in the

assembly of the tail tube ring (described below).

Structure of the tail tube

The tail tube is located below the ~~connector~~neck. It comprises a 6-fold ring of the tail terminal protein gpU and 32 repetitively stacked 6-fold rings of tail tube protein gpV (Figs 1A and B, ~~1A~~2A, ~~3A~~5A, ~~3B~~5B). The 32 rings form a right-handed helix with an axial rise of ~42 Å and a twist of ~17.5° for two adjacent rings (Fig ~~1A~~2A~~, 1~~). All 131 residues of gpU comprise an antiparallel five-strand β-sheet flanked by two α-helices, with an O-shaped loop (O-loop, residues 25-37) inserted between β1 and β2 and an extended-loop (E-loop, residues 45-58) inserted between β2 and β3 (Fig ~~3C~~5C, and ~~S4D~~ S3D Fig). The tail tube connects with the ~~connector~~ neck through interaction between the ring of the stopper protein gpFII and that of the tail terminal protein gpU (Fig ~~1A~~2A~~, 1~~ and B). To adapt to ring-ring interactions, the long loop (residues 46-62) observed in the structure of gpFII monomer in solution [43] becomes a β-hairpin (Fig ~~2M~~3M~~, 2~~ and N), which inserts into the inner surface of the gpU ring and interacts with the O-loop of gpU through β-sheet augmentation (Fig ~~3D~~5D). The C-terminal loop (residues 46-62) observed in the structure of gpFII monomer in solution [43] rotates and electrostatically interacts with the O-loop of the adjacent gpU subunit (Fig ~~3D~~5D). The E-loop of gpU provides an attachment site for the major tail protein gpV through electrostatic interactions (Figs ~~3A~~5A~~,~~ ~~3~~and I, and ~~S6 Fig~~4). The surface of the gpU ring is strongly negatively charged (Figs 4 and 6E), probably facilitating the correct positioning of the packaged DNA.

Each of the 32 rings of gpV is assembled by six copies of the major tail protein gpV wrapped around an oligomer of the tape measure protein gpH (Fig ~~1B~~2B). GpV has two distinctive domains: the N-terminal domain (gpV$_{N}$, residues 3-156) and the C-terminal domain (gpV$_{C}$, residues 157-246). The atomic structures of the two individual domains in solution have been determined by NMR spectroscopy [16, 17]. Similar to the previously lower-resolution cryo-EM structure on the lambda tail tube [22], our structure filtered to 6 Å resolution revealed six protrusions around each gpV ring; each protrusion fits well with the gpV$_{C}$ atomic model ~~(PDB ID: 2L04)~~, suggesting that the protrusions represent the gpV$_{C}$ domains (Fig ~~S7~~6A and~~,~~ ~~7~~B~~ Fig~~). At a higher resolution, the protrusions became unstructured, presumably due to their flexibility. The inner tube structure could be reconstructed to a resolution of 3.5 Å, which

enabled us to build an atomic model of the gpV$_N$ domain (Fig 6C; S1B, S~~4~~3E~~, 7C, 7D~~ Figs). The gpV$_N$ domain comprises a β-sandwich flanked by an α-helix. The β-sandwich is inserted by an ~~T~~E-loop (residues 50-78) between β3 and β4, and by an O-loop (~~T~~residues 24-38) between β1 and β2 (Fig ~~3E~~5E~~, ; S4E~~ S3E Fig). Six copies of the β-sandwich form the core of the tail tube ring (Fig ~~3G~~5G). The outer surface of the ring is formed by the α-helix and O-loop. Our structure of gpV$_N$ is similar to the previously reported NMR structure of gpV$_N$ [16], however, the N-terminal, ~~T~~E- and the C-terminal loops are flexible in the NMR structure (Fig ~~3F~~5F). These loops resulted in electrostatic interactions between the adjacent gpV$_N$ rings (Figs ~~3H~~5H, and ~~S6 Fig~~4) and between the gpU and gpV$_N$ rings (Figs ~~3I,~~ 4 and 5I~~S6 Fig~~). The flexibility of the tail tube may result from the interactions among the loops. The inner surface of the gpV ring mostly bears a negative charge (~~S7F~~ Fig 6E), similar to the tail tubes of phages T5 and 80α [15, 18], facilitating DNA transfer.

The architecture of gpV$_N$ is structurally conserved across the phage tails (~~S8~~ S6 Fig). The results of HHpred analysis indicated that the topological structure of lambda gpV$_N$ matches ~~(>99%)~~ those of SPP1 gp17.1, 80α gp53 and *Rhodobacter capsulatus* ~~gene transfer agent~~GTA (RcGTA) gp9 more than 99% probability (~~S2~~ S3 Table) [18, 20, 27, 44], although their sequence identities are low (12% to 14%).

DNA in the ~~connector~~neck-tail tube channel

The rod-like structure in the central channel of the ~~connector~~ neck and tail tube can be attributed to dsDNA and an oligomer of the tape measure protein gpH [4]. The genomic dsDNA of phages belonging to *Caudovirales* is packaged through the portal during phage assembly [45], and the end of the last packaged dsDNA is retained in the portal [39]. In phages belonging to *Caudovirales*, including siphophage SPP1 [12, 21], the narrowest part of the portal channel is formed by 12 tunnel loops. The tunnel loop, which clamps dsDNA in the portal tunnel in other phages [6, 21], does not interact closely with the dsDNA in lambda phage (Figs 1C, 1E, and ~~2~~3B~~, and S3C, 3E Fig~~). In addition, the stopper ring (gpFII) of the lambda structure exhibits an open conformation (Fig ~~1B~~2B). This finding differs from that of previous structural studies reporting that the dsDNA end of SPP1 stops at the stopper ring (formed by gp16), at which the channel is closed by the loops of gp16 [21, 25]. It is intriguing that 12 arginine residues (residue 32), which are contributed by the 12 adaptor protein gpW molecules, clamp the dsDNA (Figs

1C, 1D, ~~1B~~2B, and ~~2O~~3O~~, and S3C, 3D Fig~~). This finding is consistent with a previous study suggesting that gpW is required for the stabilization of lambda DNA within the head [42]. The gpV tube wraps around the gpH oligomer, which determines the length of the tail tube during assembly [46]. The gpH oligomer inside the tail tube cannot be well resolved probably because of its asymmetric assembly in the tail tube (Fig ~~S7~~6D~~ Fig~~). ~~In our structure, the rod-like structure below the third gpV ring was hollow (S7E Fig). We excluded the possibility that the hollow rod was due to the flexibility of the tail tube because the density levels of tube rings around solid and hollow parts of the rod are identical. These findings suggest that the hollow part of the rod is the gpH oligomer, and the dsDNA end is located at the interface between the third and fourth gpV rings. Such organization of DNA and the gpH oligomer in the channel is consistent with an earlier biochemical result suggesting that lambda DNA penetrates into the tail tube from the connector after tail assembly~~ [48]~~, but differs from a structural analysis of the isolated connector-tail complex of siphophage SPP1, suggesting that the closed stopper ring separates the DNA and the gpH oligomer in the channel~~ [23]~~. Our findings favors a previous posit that the penetration of the siphophage DNA into the tail tube after the tail assembly leads to a stable interaction between the DNA and tape measure protein, which facilitates DNA delivery~~ [49]~~. This structural organization is also consistent with a previous proposal that host-binding information is propagated to the capsid through the tape measure protein in the tail tube~~ [17]~~.~~

Structure of the tail tip complex

The tail tip complex is located at the distal end of the siphophage tail, which is the most diversified tail region. This complex is essential for host recognition and DNA ejection [18]. It comprises six proteins~~ (Fig 1C, 4A)~~: the distal tail protein gpM, hub protein gpL, ~~megatron~~ central fiber protein gpJ, insertion protein gpI, and fiber proteins stf and tfa [34]. ~~Except t~~The side tail fiber proteins stf and tfa~~, we resolved all other tail tip proteins~~ were absent from the lambda structure because stf is prevented in lambda wild type by the frameshift mutation [35].

Our structure revealed that the tip is similar to an inverted cone of 410 Å in length (Fig ~~1C~~2C, ~~4A~~7A-~~4~~C). We resolved all ~~the~~ 109 residues of the distal tail protein gpM, which has two conformers with minor differences in the E-loop (Fig ~~4D~~7D, S~~3~~4F Fig).~~ Six copies of gpM assemble into a ring (Fig 4B), which anchors the tail tip to the 6-fold tail tube through~~

~~electrostatic interaction (Fig 4E, S6 Fig).~~ Except that the gpV_N has an extended N-terminus and C-terminus, gpM is topologically identical to gpV_N (~~S8~~ S6 Fig). GpM also has a β-sandwich, which is inserted by an ~~T~~E-loop and flanked by an α-helix (Fig 7~~4~~D). The ~~T~~O-loop of gpV_N is absent from gpM because of the shorter N-terminus of gpM. Structural comparisons indicate that the architecture of the gpM domain is conserved across siphophage tails (~~S8~~ S6 Fig). Six copies of the two gpM conformers form a 3-fold gpM ring, which anchors the tail tip to the 6-fold tail tube through the β-sandwich domain via electrostatic interactions (Figs 4, 7B and E). The other side of the gpM ring (E-loop) interacts with the 3-fold ring of the gpJ and gpL complex.

~~The gpM ring is followed by gpL and gpJ. Three copies of gpL and three copies of gpJ, surrounded alternately forming the core of the trimeric conical tail tip.~~According to the four "Hub Domain" (HD) nomenclature of the siphophage and myophage baseplate hub proteins (BHPs) [4], gpJ includes the domains of HDII, HDII-insertion, HDIII, and HDIV (Fig 7G). Structural comparison between gpJ and siphophage T5 BHP pb3 [26] revealed that (i) gpJ did not have the HDI domain, and the lambda HDI domain was located in gpL (described below); (ii) the gpJ HDIV domain had an additional β-sandwich (residues 483-569) inserted between β24 (residues 477-482) and β25 (residues 570-578) (S7A and B Fig); (iii) the gpJ HDII-insertion domain had a shorter N-terminus compared with T5 pb3. The missing N-terminus of the gpJ HD-insertion could be compensated by the C-terminal domain of gpL (S7C and D Fig, described below); and (iv) an additional Ig-like domain (residues 72-215) inserted between β4 (residues 65-71) and β13 (residues 216-223) of the gpJ HDII-insertion domain (Fig 7G; S7A and D Fig). The results of positive-specific iterative basic local alignment search tool analysis revealed that the ~~two~~ Ig-like domains belonged to the fibronectin type III domain family [47]. Thus, we designate the Ig-like domain FNIII-A (Fig 7G; S7A Fig). Except for these differences, these gpJ domains are very similar to their counterparts in T5 [26] with RMSDs ranging from 1.5 to 3.0 Å (Fig 8).

The four-HD scaffold is followed by two Ig-like domains of the fibronectin type 3 sequence family (FNIII-1 and FNIII-2) in both lambda gpJ and T5 BHP pb3. The two consecutive FNIIIs are connected to the HDIV through a linker domain composed of a long loop and a short α-helix (Fig 8; S7A and B Fig). In the T5 tail tip, the three copies of the two

FNIIIs from three copies of pb3 are separated, which is caused by the negative patch at the surface of the pb3 FNIIIs [26]. By contrast, in the lambda tail tip, the three copies of the two FNIIIs from three copies of gpJ form a closed cone-like distal end of the tail tube (Fig 7A). The close interactions between these FNIIIs are enforced by a three-helix bundle below (Fig 7A). These Ig-like domains, which exist ubiquitously in phage tails, may aid in the adhesion of phage particles to bacterial cell surfaces through weakly binding to carbohydrate cell wall components such as peptidoglycan or lipopolysaccharide [48].

The protein gpL contains an N-terminal gpV_N-like β-sandwich domain (residues 1-166), which corresponds to the HDI domain of phage T5 pb3. Therefore, we designate the N-terminal region as the HDI domain. Three copies of the gpL HDI domains and three copies of gpJ HDII domains ~~,~~form the last ring of the tail. This ring is electrostatically stacked on the E-loops of the gpM ring (Figs 4 and 7A). The E-loops of the six gpM molecules exhibit two conformations to adapt to the 3-fold symmetry of the last tail ring (Fig 7D). The C-terminal domain (residues154-232) of gpL is topologically similar to the N-terminus of the T5 pb3 HDII-insertion domain (residues 160-209; S7E Fig), that is to say, the C-terminal region of gpL (iron-binding domain) and the N-terminal region of gpJ together correspond to the T5 pb3 HDII-insertion domain (S7A, C, and D Fig).

In addition, the C-terminal domain of gpL is an iron-binding domain. ~~We resolved all the 232 residues of the hub protein gpL (Fig 4F, S4G Fig). According to the nomenclature of the megatron protein of RcGTA~~ [29]~~, protein gpL can be divided into an attachment domain (residues 1-166) and an iron-binding domain (residues167-232). The attachment domain contains a gpV_N-like β-sandwich.~~ A biochemical study revealed highly conserved cysteine residues coordinating an oxygen-sensitive $[4Fe\text{-}4S]^{2+}$ cluster in the C-terminal domain of ~~lambda~~ gpL and homologous tip proteins of other siphopahges [49]. In our gpL structure, an additional density feature in the iron-binding domain, which was surrounded by four cysteine residues (Cys173, Cys182, Cys205 and Cys212) and could not be assigned to any main chain or side chain, could be modeled as the iron-sulfur cluster (Fig ~~4F~~7F). The iron-binding domain, which was previ~~s~~ously hypothesized to be embedded inside the tail tip and help stabilize gpL in its assembled form [49], was found to be located at the outer surface of the tip (Fig 7~~4~~A). Therefore, the functions of the iron-sulfur cluster, which is universally across gpL homologs

[49], remain to be characterized.~~Structural comparisons of the gpL and gpJ proteins of lambda with the baseplate proteins of phage T4 [12], phage 80α [20], RcGTA [29], and type VI secretion system[30] ~~[31]~~ revealed that both hub and central fiber proteins contain domains homologous to the baseplate proteins (S9 Fig, and S2 Table), suggesting they share common evolutionary origins. ~~

The density in the tail tip lumen was modeled as a trimer of the gpI (residues 135-223) and a three-helix coiled coil~~ comprising three α-helices~~ belonging to gpH (residues 818-842; Figs ~~1D~~2D, ~~1E~~2E, ~~and 4C~~7C, and ~~4H~~7H). The gpI trimer forms a tripod to support the coiled coil in the lumen (Fig ~~4C~~7C and~~,~~ 4H), and each ~~foot of the tripod~~gpI molecule interacts with the HDII, ~~gpJ~~ HDII-insertion, and FNIII-A of gpJ through three β-sheet augmentations~~ in the central, adhesion-like, and Ig-like A domains~~ (Fig 7~~4~~I). The gpH coiled coil terminates the rod-like structure in the tail tip lumen (Fig ~~1C~~2C). A sequence-based secondary structures prediction study [50] revealed that the α-helix is located at the C-terminus of helical gpH (~~S10~~ S8 Fig). Tape measure protein is a multifunctional protein found in both *Siphoviridae* and *Myoviridae*. During tail assembly, tape measure protein interacts with chaperone proteins to initiate tail polymerization and determines the length of the assembled tail [46, 51, 52]. In addition, it may transmit the host-binding signal to the phage capsid and then exit from the tail and form a channel to span the host cell envelope for phage DNA delivery [15, 53, 54]. Our structure suggests that the gpH oligomer is formed by ~~a cascade of~~ gpH trimer with the gpH C-terminus located in the tail tip. The ~~coiled coil and tripod~~ gpI trimer ~~together~~ serves as a plug to prevent the early release of the gpH oligomer and DNA from the tail tube (Fig 7H). In T5, a plug domain is inserted in the HDII-insertion domain of BHP. Three copies of the plug domains also form a tripod and serve to close the tube [26] in the same location as the gpI tripod in lambda (Fig 8A and B; S7B and D Fig). It is noteworthy that gene I is located between genes L and J, a gene location that corresponds to the position of the T5 pb3 plug domain, except for an inserted gene K (S9 Fig). Therefore, we suggest that gpL, gpI, and the N-terminal region of gpJ (from HDII to FNIII-1 and -2) together correspond to T5 BHP pb3 (S9 Fig). The resolved N-terminal region of gpI is highly hydrophobic (residues 137-GILFSLGASMVLGGVA-152), suggesting that it might interact with the bacterial outer membrane, as does the T5 pb3 plug domain [26].

Further structural comparisons of the gpL and gpJ proteins of lambda with the homologous

proteins of phage T4 [10], phage 80α [18], RcGTA [27], and T6SS [28] revealed that they all contained four HD domains (Fig 8), suggesting they share a common evolutionary origin. However, T4, 80α, and T6SS lack HDII-insertion and FNIII domains. For T5, T4, 80α, and T6SS, the four HD domains are located within a single protein. By contrast, RcGTA has similar organization of the four HD domains to that of lambda. The RcGTA HDI domain is located in the N-terminal regions of gp13, while its rest three HD domains are located in gp15 (Fig 8F). As in lambda, the RcGTA HDII-insertion domain is also distributed in two tail tip proteins: the N-terminal region of the HDII-insertion domain is located in the C-terminal region of gp13, while the C-terminal region of the HDII-insertion domain is located in the N-terminal region of gp15 (Fig 8F). Indeed, the arrangement of RcGTA genes 13, 14, and 15 [27] is very similar to that of lambda genes L, K, I, and J (S9 Fig). It is noteworthy that both lambda and RcGTA have iron-sulfur clusters in the tail tip protein (gpL/gp13). Among these tail tip proteins, only lambda gpJ and T5 pb3 contain FNIII domains. The insertion/deletion and distribution of domains in these tail tips reflect the long evolutionary history of these phages and phage tail-like machines.

In addition to the aforementioned domains corresponding to T5 BHP, gpJ also contains two additional domains in its C-terminal region: a central fiber domain and a receptor-binding domain, which are the counterparts of the T5 central fiber protein pb4 and the receptor binding protein pb5 respectively. These two domains were not resolved at high resolution (Fig 7A) due to their flexibility. The flexibility of the two tail tip domains may enable the receptor binding domain to explore larger spaces for the bacterial receptor. We could only model the N-terminal regions of the three central fiber domains as three-helix bundle based on the gpJ density map (Fig 7G). The helix bundle links the central fiber and receptor binding domains to the FNIII-2 domain (Fig 7A). We performed structure predictions for gpJ (S10A Fig) by using AlphaFold2 [55]. Comparison between the predicted gpJ structure and our gpJ structure in the tail tip showed that the domains of HDII, HDII-insertion, HDIII, and HDIV fit well (RMSD: 1.7 Å), except for the deviation of the FNIII-A, -1, and -2 domains (S10B Fig). If we segment the three FNIII domains from the predicted gpJ structure, they can fit well into their counterparts in our gpJ structure, with RMSDs ranging from 0.7 to 1.8 Å. The deviation of the FNIII-1 and -2 in the predicted gpJ structure was caused by the tilt of the linker domain (S10B Fig). Furthermore,

the gpJ central fiber domain was predicted to be a β-strand rich region (S10A Fig). It is likely that these β-strands form β-helix in trimer, as the T5 central fiber protein pb4 [26]. The C-terminal tip of gpJ was predicted to be an Ig-like domain (S10A Fig), which could be attributed to the receptor-binding domain [56]. Through the receptor-binding domain, gpJ binds to the receptor protein LamB on the surface of the host cell [57-59]. The two gpJ domains were curved in the predicted structure (S10 Fig), in contrast, they appeared to be relatively straight in the density map (Fig 7A). The difference between the predicted gpJ structure and our gpJ structure might reflect the conformational change of gpJ from the monomer state in solution to the assembled state. It can be inferred that the tail tip of lambda would undergo structural changes upon receptor interaction similar to the structural changes of T5 [26]. ~~During infection,~~ T~~t~~he receptor binding process may triggers~~s~~ conformational changes in gpJ, which presumably disrupt the interactions ~~between~~ among gpL, gpI, and gpJ, thus initiating the release of gpH oligomer and DNA. Ultrathin sections of the lambda infected E. coli cells analyzed using electron microscopy showed that the lambda wild type particles adsorbed perpendicular to the cell surface (S11 Fig), although these particles lacked side tail fibers, suggesting that the perpendicular adsorption did not need the participation of the side tail fibers. This observation was in contrast to the adsorption of podophage T7, in which the six distal halves of the six side tail fibers extended vertically on the outer membrane to support the T7 particles, allowing them to orient perpendicularly to the cell surface [60]. It is probable that the conformational changes of gpL, gpI, gpJ, and the released gpH are sufficient to facilitate the lambda head and tail tube to orient perpendicularly to the cell surface.

~~According to the nomenclature of the megatron protein of RcGTA [29] and gp5 protein of phage T4 [12], the megatron protein gpJ can be divided into eight domains: an adhesin-like domain (residues 1-83 and 209-240), two Ig-like domains A (residues 84-208) and B (residues, 609-712), a central domain (residues 241-482 and 568-608), two oligosaccharide-binding domains A (residues 483-567) and B (residues 713-808), a lysozyme domain (residues 809-872), and a receptor binding (tail fiber) domain (residues 873-1132). We resolved 851 N-terminal residues of the 1132-residue multi-domain protein gpJ (Fig 4G, S4H Fig). The central domain of gpJ contains a β-sandwich. Alternately arranged β-sandwiches of the gpJ central and gpL attachment domains form a pseudo 6-fold ring, which is electrostatically stacked on the~~

TE-loops of the gpM ring (Fig 4C, and S6 Fig). The pseudo 6-fold ring is surrounded by the β-sandwich of the oligosaccharide-binding domain A, which may serve as a ligand binding site for side fiber attachment [64]. The Ig-like domains A and B lie at the middle of the tail tip. The Ig-like domain A of the gpJ trimer is represented by three protrusions on the outer surface of the tail tip, which may facilitate protein-carbohydrate interactions during phage infection [51]. Three copies each of the Ig-like domain B and oligosaccharide-binding domain B form a narrowed end of the tail channel. The receptor binding domain and C-terminal region of the lysozyme domain could not be resolved to the near-atomic resolution. A low resolution density map indicated that they form a nodule and a knob connecting to the helix bundle of the lysozyme domain (Fig 4A).

Lambda gpJ is not only an initiator of tail assembly but also a receptor binding protein. Through its receptor-binding domain, gpJ binds to the receptor protein LamB on the surface of the host cell [60-62].

Materials and Methods

Bacteriophage lambda purification

The E. coli strain MG1655 (American Type Culture Collection ID 47076) was grown in Luria–-Bertani Broth (tryptone, 10 g; yeast extract, 5 g; and NaCl, 10 g/L) for 24h at 37°C. The cultured strain was re-cultured at 37°C for 4h. During the logarithmic growth of *E. coli*, the bacterial cells were incubated with lambda W60 phages for 4h at 37°C. The phage culture was lysed using chloroform. Subsequently, the cell debris was removed through centrifugation at 6000 × g for 20min at 8°C. The supernatant was enriched through polyethylene glycol (PEG) 8000 precipitation (10% w/v PEG in 1M NaCl) overnight at 4°C. The precipitated phages were resuspended in phage buffer (10 mM $MgSO_4$ and 50 mM Tris-HCl, pH 7.4) and then purified by cesium chloride density gradient centrifugation at 90,000 × g for 2h at 8°C. After centrifugation, bands containing phage particles were collected and dialyzed in phage buffer (10 mM $MgSO_4$ and 50 mM Tris-HCl, pH 7.4) overnight. Final, purified infected lambda phages were in ice-water for cryo-sample processing.

Ultrathin section of lambda-adsorbed cells

The lambda phage and bacteria mixture was incubated in 37°C water bath for 15 min and

then pelleted by low speed centrifugation. The pellets were fixed with 2% paraformaldehyde-2.5% glutaraldehyde buffered in 0.1 M sodium cacodylate overnight and post fixed with 1% osmium tetroxide for 2 hrs. Then the samples were dehydrated with gradient alcohol of increasing concentration until 100%. Finally, the samples were infiltrated and embedded in epoxy resin. The resin blocks were cut using ultramicrotome to get 80nm thickness sections which were stained with 1% uranyl acetate and 0.4% lead citrate, respectively. The ultrathin sections were examined under an FEI TF20 200 kV electron microscope.

Mass spectrometry analysis

The purified lambda phage was run on a 4-12% SDS-PAGE, and the Coomassie-stained bands were manually excised for mass spectrometry analysis. The mass spectrometry data processing and analyses were performed using Q Exactive mass spectrometer (Thermo Scientific) and software package Proteome Discovery version 1.4. The Sequest HT search engine was used for sequence searches in the MS/MS spectra against the Uniprot database.

Cryo-EM and data collection

An aliquot of 3 μL purified lambda phages was applied to 400-mesh Quantifoil R2/1 copper grids ~~covered by a 5-nm-thick layer of continuous carbon that had been glow-discharged for 20 s~~, which~~.~~ were covered with an additional 5-nm-thick layer of continuous carbon using a Q150T turbomolecular pumped coater (Quorum Technologies, UK) and glow-discharged for 20 s. The grids were loaded into an FEI Vitrobot (temperature, 8 °C, humidity, 100%, and blot time, ~~,~~ 3.5 s). After ~~double~~ blotting the excess solution on the grid two times, the grids were plunge-~~d~~ frozen ~~by~~ using solid–~~-~~liquid ethane and stored in liquid nitrogen until subsequent analyses. Cryo-EM data were recorded using the 300 kV Titan Krios G3i microscope equipped with a Gatan imaging filter and a K3 summit direct electron detector, and a Cs corrector. The electron microscope was operated at 300 kV voltage, the Gatan imaging filter was used with a slit width of 20 eV to remove inelastically scattered electrons, and ~~T~~the K3 detector was operated in the super-resolution mode~~l~~. The pixel size was calibrated using 500-nm diffraction grating replica and latex calibration standard. Automatic data were collected using the FEI EPU software at a magnification of 53,000 (physical pixel size: 1.36 Å), which resulted in a pixel size of 0.68 Å per pixel. The accumulated dose of each movie was 32 $e^-/Å^2$, and the defocus values of the images ranged from 1.6 to 2.2 μm. Finally, 5513 movies were collected; each

movie stack comprised 32 image frames.

Image processing

Icosahedral reconstruction for the head. The defocus and astigmatism values of each cryo-EM micrograph were calculated by using GCTF [61] in RELION software [62]. A total of 97,030 particle images of the ~~phage~~ lambda head (box size 1024×1024 pixels) were boxed out from 5,513 cryo-EM images using the software ETHAN [63]. The icosahedral head of lambda was reconstructed by using our programs [64] based on the common-line algorithm [65, 66].

Symmetry-mismatch and local reconstructions. We manually selected 10,220 images (box size 900×900 pixels) of the complete lambda particles with straight tails from the cryo-EM images, which were re-sampled to a pixel size of 5.44 Å. Based on the orientation and center parameters of each particle image obtained from the icosahedral reconstruction, we ~~manually selected 10,220 particles with straight tail to~~ reconstructed the intact asymmetric structure of the phage lambda using our symmetry-mismatch reconstruction method [6, 37]. Briefly, for each particle image, we first localized the unique vertex (portal–neck~~connector~~) by searching the regions of 12 icosahedral vertices, which were determined in the icosahedral reconstruction step. Then, a low-resolution structure of the phage with the tail was obtained. The resolution was further improved through the iteration of the projection-refinement-reconstruction step. In this step, we selected one of the 60 possible icosahedral orientations for each particle image. The iteration was continued until the orientations of all particle images were stabilized and the phage structure (~20 Å resolution) could not be improved further. The aforementioned steps were performed for all 97,030 particle images. Thus, we obtained a phage structure with the portal–neck ~~connector~~ and the proximal part of the tail tube but without the distal part of the tail (because the tail is flexible) at a resolution of ~6 Å. Next, we segmented the portal–neck ~~connector~~ from the phage structure to use it as an initial model for local refinement and reconstruction; thus we determined the structure of the gpB (portal) and gpW (adaptor) rings at a resolution of 3.2 Å by imposing the 12-fold symmetry and the structure of the gpFII, gpU, and two proximal gpV rings at a resolution of 3.5 Å.

Local reconstructions of the tail tube and neck. The tail tube comprises 32 repetitive hexameric rings. To increase the particle number, we manually selected 181,657 tail tube particles by using the EMAN Helix software [67] with a box size of ~~300~~220×~~300~~ 220 ~~Å~~pixels.

Using the low-resolution density map, which was segmented from the intact virion, as an initial model, we performed two-dimensional (2D) classification and three-dimensional (3D) reconstruction by using RELION software [62]. A total of 121,571 particle images were selected for performing auto-refinement and 3D reconstruction with the C6 symmetry imposed. Finally we improved resolution of the tail tube structure to 3.48 Å. The tail neck was reconstructed using the same procedure as the tail tube except that the box size of the neck images was 400×400 pixels.

Local reconstruction of the tail tip complex. We preformed the local reconstruction of the tail tip complex by using RELION software [62] (S12~~3~~ Fig). First, 70,745 tail tip particle images with a box size of 220×220 pixels were manually selected for 2D classification to exclude non-tip ~~or~~ and other irrelevant segments. The classification results indicated that the centers mostly varies across particles. Using a randomly generated cylinder density as the initial model, we selected 58,215 particles from 2D classifications to perform 3D classification with the C3 symmetry. Thus, we obtained six types of low-resolution structures with varying center positions (S12~~3~~E Fig). Furthermore, we performed Z-axis translation to correct the center of each type on the basis of the first type and re-extracted tail tip images. Subsequently, 54,385 tail tip images were selected for 3D refinement with the C3 symmetry imposed. Thus we obtained a structure of the tail tip complex at a resolution of 3.72 Å. Finally, we refined the contrast transfer function to improve the resolution of the tail tip structure to 3.44 Å.

Model building

Using the COOT software [68], We manually built the atomic models of proteins gpB, gpW, gpFII, gpU, gpV_N, gpM, gpL, gpJ, gpI and gpH (residues 849-818) on the basis of our cryo-EM density map. Furthermore, we refined the models through in real-space refinement, implemented in Phenix [69]. The refinement and validation statistics are presented (~~S3~~ S4 Table).

Acknowledgements

Cryo-EM data collection was carried out at the Shuimu BioSciences Ltd. We thank Fanhao Meng, Zhenqian Guo and other staff members at the Shuimu BioSciences for data collections.

Author contributions

H.L., L.C., and J.S. designed the research; H.X., J.S. produced the lambda sample and prepared cryo-EM grids; H.X., L.T., J.S. collected the cryo-EM data; H.X., L.T., Z.T., Y.Z., W.C., X.L., L.C., and H.L. analyzed data; H.X., L.T., L.C., and H.L. wrote the paper, with further edits from all authors.

References

1. Wommack KE, Colwell RR. Virioplankton: viruses in aquatic ecosystems. Microbiol Mol Biol Rev. 2000;64(1):69-114.

2. Ackermann HW. 5500 Phages examined in the electron microscope. Arch Virol. 2007;152(2):227-43.

3. Ackermann HW. Bacteriophage observations and evolution. Res Microbiol. 2003;154(4):245-51.

4. Linares R, Arnaud CA, Degroux S, Schoehn G, Breyton C. Structure, function and assembly of the long, flexible tail of siphophages. Curr Opin Virol. 2020;45:34-42.

5. Cuervo A, Fabrega-Ferrer M, Machon C, Conesa JJ, Fernandez FJ, Perez-Luque R, et al. Structures of T7 bacteriophage portal and tail suggest a viral DNA retention and ejection mechanism. Nat Commun. 2019;10(1):3746.

6. Chen WY, Xiao H, Wang XR, Song SL, Han Z, Li XW, et al. Structural changes of a bacteriophage upon DNA packaging and maturation. Protein Cell. 2020;11(5):374-9.

7. Zhang JT, Yang F, Du K, Li WF, Chen Y, Jiang YL, et al. Structure and assembly pattern of a freshwater short-tailed cyanophage Pam1. Structure. 2022;30(2):240-51 e4.

8. Xu J, Wang D, Gui M, Xiang Y. Structural assembly of the tailed bacteriophage varphi29. Nat Commun. 2019;10(1):2366.

9. Li F, Hou CD, Yang R, Whitehead R, 3rd, Teschke CM, Cingolani G. High-resolution cryo-EM structure of the Shigella virus Sf6 genome delivery tail machine. Sci Adv. 2022;8(49):eadc9641.

10. Taylor NMI, Prokhorov NS, Guerrero-Ferreira RC, Shneider MM, Browning C, Goldie KN, et al. Structure of the T4 baseplate and its function in triggering sheath contraction. Nature. 2016;533(7603):346-+.

11. Zheng WL, Wang FB, Taylor NMI, Guerrero-Ferreira RC, Leiman PG, Egelman EH. Refined Cryo-EM Structure of the T4 Tail Tube: Exploring the Lowest Dose Limit. Structure. 2017;25(9):1436-+.

12. Lhuillier S, Gallopin M, Gilquin B, Brasiles S, Lancelot N, Letellier G, et al. Structure of bacteriophage SPP1 head-to-tail connection reveals mechanism for viral DNA gating. P Natl Acad Sci USA. 2009;106(21):8507-12.

13. Veesler D, Robin G, Lichiere J, Auzat I, Tavares P, Bron P, et al. Crystal Structure of Bacteriophage SPP1 Distal Tail Protein (gp19.1) A BASEPLATE HUB PARADIGM IN GRAM-POSITIVE INFECTING PHAGES. Journal of Biological Chemistry. 2010;285(47):36666-73.

14. Langlois C, Ramboarina S, Cukkemane A, Auzat I, Chagot B, Gilquin B, et al. Bacteriophage SPP1 Tail Tube Protein Self-assembles into beta-Structure-rich Tubes. Journal of Biological Chemistry. 2015;290(6):3836-49.

15. Arnaud CA, Effantin G, Vives C, Engilberge S, Bacia M, Boulanger P, et al. Bacteriophage T5 tail tube structure suggests a trigger mechanism for Siphoviridae DNA ejection. Nat Commun. 2017;8.

16. Pell LG, Kanelis V, Donaldson LW, Howell PL, Davidson AR. The phage lambda major tail protein structure reveals a common evolution for long-tailed phages and the type VI bacterial secretion system. Proc Natl Acad Sci U S A. 2009;106(11):4160-5.

17. Pell LG, Gasmi-Seabrook GM, Morais M, Neudecker P, Kanelis V, Bona D, et al. The solution structure of the C-terminal Ig-like domain of the bacteriophage lambda tail tube protein. J Mol Biol. 2010;403(3):468-79.

18. Kizziah JL, Manning KA, Dearborn AD, Dokland T. Structure of the host cell recognition and penetration machinery of a Staphylococcus aureus bacteriophage. Plos Pathog. 2020;16(2).

19. Hardy JM, Dunstan RA, Grinter R, Belousoff MJ, Wang J, Pickard D, et al. The architecture and

stabilisation of flagellotropic tailed bacteriophages. Nat Commun. 2020;11(1):3748.

20. Zinke M, Sachowsky KAA, Oster C, Zinn-Justin S, Ravelli R, Schroder GF, et al. Architecture of the flexible tail tube of bacteriophage SPP1. Nat Commun. 2020;11(1):5759.

21. Chaban Y, Lurz R, Brasiles S, Cornilleau C, Karreman M, Zinn-Justin S, et al. Structural rearrangements in the phage head-to-tail interface during assembly and infection. Proc Natl Acad Sci U S A. 2015;112(22):7009-14.

22. Campbell PL, Duda RL, Nassur J, Conway JF, Huet A. Mobile Loops and Electrostatic Interactions Maintain the Flexible Tail Tube of Bacteriophage Lambda. J Mol Biol. 2020;432(2):384-95.

23. Sciara G, Bebeacua C, Bron P, Tremblay D, Ortiz-Lombardia M, Lichiere J, et al. Structure of lactococcal phage p2 baseplate and its mechanism of activation. P Natl Acad Sci USA. 2010;107(15):6852-7.

24. Veesler D, Spinelli S, Mahony J, Lichiere J, Blangy S, Bricogne G, et al. Structure of the phage TP901-1 1.8 MDa baseplate suggests an alternative host adhesion mechanism. P Natl Acad Sci USA. 2012;109(23):8954-8.

25. Orlova EV, Gowen B, Droge A, Stiege A, Weise F, Lurz R, et al. Structure of a viral DNA gatekeeper at 10 A resolution by cryo-electron microscopy. Embo J. 2003;22(6):1255-62.

26. Linares R, Arnaud CA, Effantin G, Darnault C, Epalle NH, Boeri Erba E, et al. Structural basis of bacteriophage T5 infection trigger and E. coli cell wall perforation. Sci Adv. 2023;9(12):eade9674.

27. Bardy P, Fuzik T, Hrebik D, Pantucek R, Thomas Beatty J, Plevka P. Structure and mechanism of DNA delivery of a gene transfer agent. Nat Commun. 2020;11(1).

28. Quentin D, Ahmad S, Shanthamoorthy P, Mougous JD, Whitney JC, Raunser S. Mechanism of loading and translocation of type VI secretion system effector Tse6. Nat Microbiol. 2018;3(10):1142-52.

29. Spinola-Amilibia M, Davo-Siguero I, Ruiz FM, Santillana E, Medrano FJ, Romero A. The structure of VgrG1 from Pseudomonas aeruginosa, the needle tip of the bacterial type VI secretion system. Acta Crystallogr D Struct Biol. 2016;72(Pt 1):22-33.

30. Casjens SR, Hendrix RW. Bacteriophage lambda: Early pioneer and still relevant. Virology. 2015;479:310-30.

31. Catalano CE. Bacteriophage lambda: The path from biology to theranostic agent. Wires Nanomed Nanobi. 2018;10(5).

32. Lander GC, Evilevitch A, Jeembaeva M, Potter CS, Carragher B, Johnson JE. Bacteriophage lambda stabilization by auxiliary protein gpD: Timing, location, and mechanism of attachment determined by cryo-EM. Structure. 2008;16(9):1399-406.

33. Rao VB, Fokine A, Fang QL. The remarkable viral portal vertex: structure and a plausible model for mechanism. Curr Opin Virol. 2021;51:65-73.

34. Rajagopala SV, Casjens S, Uetz P. The protein interaction map of bacteriophage lambda. Bmc Microbiol. 2011;11.

35. Hendrix RW, Duda RL. Bacteriophage lambda PaPa: not the mother of all lambda phages. Science. 1992;258(5085):1145-8.

36. Wang C, Zeng JW, Wang JW. Structural basis of bacteriophage lambda capsid maturation. Structure. 2022;30(4).

37. Liu HR, Cheng LP. Cryo-EM shows the polymerase structures and a nonspooled genome within a dsRNA virus. Science. 2015;349(6254):1347-50.

38. Chen WY, Xiao H, Wang L, Wang XR, Tan ZX, Han Z, et al. Structural changes in bacteriophage T7 upon receptor-induced genome ejection. P Natl Acad Sci USA. 2021;118(37).

39. Prevelige PE, Cortines JR. Phage assembly and the special role of the portal protein. Curr Opin Virol. 2018;31:66-73.
40. Fang Q, Tang WC, Tao P, Mahalingam M, Fokine A, Rossmann MG, et al. Structural morphing in a symmetry-mismatched viral vertex. Nat Commun. 2020;11(1):1713.
41. Krissinel E, Henrick K. Inference of macromolecular assemblies from crystalline state. J Mol Biol. 2007;372(3):774-97.
42. Maxwell KL, Yee AA, Booth V, Arrowsmith CH, Gold M, Davidson AR. The solution structure of bacteriophage lambda protein W, a small morphogenetic protein possessing a novel fold. J Mol Biol. 2001;308(1):9-14.
43. Maxwell KL, Yee AA, Arrowsmith CH, Gold M, Davidson AR. The solution structure of the bacteriophage lambda head-tail joining protein, gpFII. J Mol Biol. 2002;318(5):1395-404.
44. Zimmermann L, Stephens A, Nam SZ, Rau D, Kubler J, Lozajic M, et al. A Completely Reimplemented MPI Bioinformatics Toolkit with a New HHpred Server at its Core. J Mol Biol. 2018;430(15):2237-43.
45. Zinke M, Schroder GF, Lange A. Major tail proteins of bacteriophages of the order Caudovirales. J Biol Chem. 2022;298(1):101472.
46. Katsura I. Determination of bacteriophage lambda tail length by a protein ruler. Nature. 1987;327(6117):73-5.
47. Altschul SF, Madden TL, Schaffer AA, Zhang J, Zhang Z, Miller W, et al. Gapped BLAST and PSI-BLAST: a new generation of protein database search programs. Nucleic Acids Res. 1997;25(17):3389-402.
48. Fraser JS, Yu Z, Maxwell KL, Davidson AR. Ig-like domains on bacteriophages: a tale of promiscuity and deceit. J Mol Biol. 2006;359(2):496-507.
49. Tam W, Pell LG, Bona D, Tsai A, Dai XX, Edwards AM, et al. Tail tip proteins related to bacteriophage lambda gpL coordinate an iron-sulfur cluster. J Mol Biol. 2013;425(14):2450-62.
50. Buchan DWA, Minneci F, Nugent TCO, Bryson K, Jones DT. Scalable web services for the PSIPRED Protein Analysis Workbench. Nucleic Acids Research. 2013;41(W1):W349-W57.
51. Katsura I, Kuhl PW. A regulator protein for the length determination of bacteriophage lambda tail. J Supramol Struct. 1974;2(2-4):239-53.
52. Xu J, Hendrix RW, Duda RL. Chaperone-Protein Interactions That Mediate Assembly of the Bacteriophage Lambda Tail to the Correct Length. J Mol Biol. 2014;426(5):1004-18.
53. Mahony J, Alqarni M, Stockdale S, Spinelli S, Feyereisen M, Cambillau C, et al. Functional and structural dissection of the tape measure protein of lactococcal phage TP901-1. Sci Rep-Uk. 2016;6.
54. Cumby N, Reimer K, Mengin-Lecreulx D, Davidson AR, Maxwell KL. The phage tail tape measure protein, an inner membrane protein and a periplasmic chaperone play connected roles in the genome injection process of E. coli phage HK97. Mol Microbiol. 2015;96(3):437-47.
55. Jumper J, Evans R, Pritzel A, Green T, Figurnov M, Ronneberger O, et al. Highly accurate protein structure prediction with AlphaFold. Nature. 2021;596(7873):583-9.
56. Wang J, Hofnung M, Charbit A. The C-terminal portion of the tail fiber protein of bacteriophage lambda is responsible for binding to LamB, its receptor at the surface of Escherichia coli K-12. J Bacteriol. 2000;182(2):508-12.
57. Roessner CA, Struck DK, Ihler GM. Morphology of complexes formed between bacteriophage lambda and structures containing the lambda receptor. J Bacteriol. 1983;153(3):1528-34.
58. Roessner CA, Ihler GM. Proteinase sensitivity of bacteriophage lambda tail proteins gpJ and pH in complexes with the lambda receptor. J Bacteriol. 1984;157(1):165-70.
59. Roessner CA, Ihler GM. Formation of transmembrane channels in liposomes during injection of

lambda DNA. J Biol Chem. 1986;261(1):386-90.
60. Hu B, Margolin W, Molineux IJ, Liu J. The bacteriophage t7 virion undergoes extensive structural remodeling during infection. Science. 2013;339(6119):576-9.
61. Zhang K. Gctf: Real-time CTF determination and correction. J Struct Biol. 2016;193(1):1-12.
62. Scheres SHW. RELION: Implementation of a Bayesian approach to cryo-EM structure determination. J Struct Biol. 2012;180(3):519-30.
63. Kivioja T, Ravantti J, Verkhovsky A, Ukkonen E, Bamford D. Local average intensity-based method for identifying spherical particles in electron micrographs. J Struct Biol. 2000;131(2):126-34.
64. Li X, Zhou N, Chen W, Zhu B, Wang X, Xu B, et al. Near-Atomic Resolution Structure Determination of a Cypovirus Capsid and Polymerase Complex Using Cryo-EM at 200kV. J Mol Biol. 2017;429(1):79-87.
65. Thuman-Commike PA, Chiu W. Improved common line-based icosahedral particle image orientation estimation algorithms. Ultramicroscopy. 1997;68(4):231-55.
66. Fuller SD, Butcher SJ, Cheng RH, Baker TS. Three-dimensional reconstruction of icosahedral particles--the uncommon line. J Struct Biol. 1996;116(1):48-55.
67. Ludtke SJ, Baldwin PR, Chiu W. EMAN: semiautomated software for high-resolution single-particle reconstructions. J Struct Biol. 1999;128(1):82-97.
68. Emsley P, Lohkamp B, Scott WG, Cowtan K. Features and development of Coot. Acta Crystallogr D Biol Crystallogr. 2010;66(Pt 4):486-501.
69. Adams PD, Afonine PV, Bunkoczi G, Chen VB, Davis IW, Echols N, et al. PHENIX: a comprehensive Python-based system for macromolecular structure solution. Acta Crystallogr D Biol Crystallogr. 2010;66(Pt 2):213-21.

Figure legends

Fig 1. Symmetry-mismatch reconstruction of the intact lambda virion. (A) Image of a lambda particle with a straight tail. (B) Overall view of an intact lambda phage at a resolution of approximately 20 Å. The numbers of the tail rings are labeled. (C) Slab view of the neck showing the DNA within the neck (red arrow). (D) Density map (transparent) at a resolution of 3.5 Å superimposed on the atomic models of gpW (cyan ribbon) and gpFII (purple ribbon), showing the interactions between the Arg32 residue of gpW and dsDNA. (E) Density map (transparent) at a resolution of 3.5 Å superimposed on the atomic model of gpB (hot pink ribbon), showing the interactions between the Gln379 residue of gpB and dsDNA.

Fig 2. Structure of the lambda virion. (A) Left: scheme of the lambda virion. Each protein is shown using a different color and the numbers indicate the copy number of the proteins. Middle and

right: surface and cut-open views of the lambda virion. The head, neck, tail tube, and tail tip are colored in grey, magenta, sky blue, and golden, respectively; one of the six protrusions of each ring of the tail tube is presented in blue to depict the right-handed helix assembly of the tail tube. (B) Surface and slab views of the portal (gpB), neck (gpW, and gpFII), tail terminator (gpU), and two tail tube (gpV$_N$) rings at a resolution of 3.5 Å. The thick dashed line marks the capsid border, and different colors have been used for different proteins. (C) Surface and cut-open views of the tail tip with a gpV$_N$ ring. Different colors have been used for different proteins. (D-E) Superposition of the resolved models of gpI and gpH on their density maps (transparent) indicates the quality of the density maps.

Fig 3. Structure of the portal and neck. (A, B) Overall and slab view of the atomic model (ribbon) of the neck. The color codes for portal (gpB), adaptor (gpW), and stopper (gpFII) are identical to those in Fig. 2B. The gray rod represents the density of phage DNA. (C) Structure of gpB shown using various colors for its domains. (D) Density map (transparent) of the gpB tunnel loop superimposed on its atomic model. (E, F) Side and top views of the adaptor ring. A copy of gpW is shown using various colors for its domains. (G) Zoom-in view of the gpW structure in panel (E). (H) NMR structure of gpW in solution (PDB ID: 1HYW) showing that the β-hairpin domain undergoes a 90°rotation. (I) Zoomed-in view of the black box in panel F showing the hydrophobic interactions in the α-helix bundle domains of two adjacent gpW molecules. (J) C-arm of gpW interacts with gpB through β-sheet augmentation. (K, L) Side and top views of the stopper. One gpFII molecule is shown using various colors for its domains. (M) Zoomed-in view of gpFII in panel (K). (N) NMR structure of gpFII in solution (PDB ID: 1K0H). (O) Cross-section view of black column in panel A showing the interactions among gpFII, gpW, and DNA.

Fig 4. Electrostatic potential surfaces of the interacting regions of two adjacent rings.

Fig 5. Structure of the tail tube. (A) Rings of gpFII, gpU, and gpV$_N$ (only three rings of gpVN are shown). The color codes are identical to those in Fig. 2B, except for the fact that one gpU molecule and one gpV$_N$ molecule are shown in rainbow colors, ranging from blue at the N-terminus to red at the C-terminus. (B) Top view of the gpU ring. One of the six gpU molecules is shown in rainbow colors. (C) Structure of the gpU molecule in the tail tube. (D) Zoomed-in view (center) of the red box in panel A showing β-sheet augmentation (left) and electrostatic (right) interactions between gpU and gpFII. (E) Structure of the gpV$_N$ molecule present in the tail tube. (F) NMR structure of

gpV_N in solution (right; PDB ID: 2k4q), showing conformational changes in the E-loop and N- and C- termini. (G) Top view of the hexameric gpV_N ring. One of the six gpU molecules is shown in rainbow colors. (H) Zoomed-in view (left) of the blue box in panel A showing electrostatic interactions (right) between two adjacent gpV_N rings. (I) Zoomed-in view (left panel) of the black box in panel A showing electrostatic interactions between gpU and gpV_N (right panel).

Fig 6. Density map of the tail tube. (A) Two-dimensional class average of the tail tube. White, cyan, and orange arrows indicate the rings of gpV_N and gpV_C and the rod of gpH or dsDNA, respectively. (B) Density map of the tail tube filtered to a resolution of 6 Å. Superposition of an atomic model of gpV_C (PDB DI: 2L04) on the protrusion (right). (C) Side and top views of the high-resolution density map of the tail tube. One monomer of gpV_N is shown in yellow. (D) Cut-open view of the tail tube. (E) Electron potential on the inner channel of the neck-tail complex.

Fig 7. Structure of the tail tip. (A) Structure of the tail tip. The C-terminal region of gpJ, which was not resolved to the near-atomic resolution, is displayed using a density map (grey). (B) Top and bottom views of the gpM ring. One of the six copies of gpM is shown in rainbow colors. (C) Cross-section view of black column in panel A showing that the proteins gpJ and gpL form a trimeric conical tip. (D) Two conformers of gpM shown in rainbow colors. (E) Zoomed-in view (top left) of the blue box in panel A showing electrostatic interactions between gpM and gpV_N (top right) and among gpM, gpJ, and gpL (bottom panels). (F) Structure of gpL shown using various colors for its domains (left) and the zoomed-in view (top right) of the model of the iron–sulfur cluster superimposed on its density map (transparent). A central section of the iron-binding site was shown (bottom right). (G) Ribbon model of gpJ shown using various colors for its domains. (H) Side and top views of trimeric gpI (red) and gpH (golden). (I) β-sheet augmentation-mediated interactions between gpI (red) and gpJ (green).

Fig 8. Structural comparison between lambda, other phages, and tail-like machines. (A-F) Stucture of the hub and central fiber proteins of lambda (gpL and gpJ), the baseplate hub or homologous proteins of phage T5 (7ZQB), phage 80α (PDB ID: 6V8I), T4 (PDB ID: 5IV5), T6SS (PDB ID: 6H3L), and RcGTA (PDB ID: 6TEH). The homologous domains in these proteins are in the same color. The N- and C- termini of these proteins were labeled. For the N- and C- termini of gpL and gpJ, see Fig S7A and C for separate views of gpJ and gpL.

Supporting information

S1 Fig. Cryo-EM image and Fourier shell correlation curves. (A) Cryo-EM image of mature lambda phage showing its long flexible tail. (B) Estimated structural resolutions of the local reconstructions of the gpB and gpW with an imposed symmetry of 12 folds (black line), the gpFII, gpU, and gpVN with an imposed symmetry of 6 folds (red line), the tail tube (gpVN) with an imposed symmetry of 6 folds (blue line), the tail tip complex with an imposed symmetry of 3 folds (magenta line), and the portal-capsid without symmetry imposed (cyan line).

S2 Fig. Reconstruction of the icosahedral head. (A) Overall view of the icosahedral head structure. Seven copies of the major capsid protein gpE arranged in an asymmetric unit are shown in purple, green, hot pink, cornflower blue, cyan, dark green, and magenta and six trimers of the cement protein gpD surrounding the asymmetric unit are shown in orange. (B) Zoomed-in view of the asymmetric unit in panel A. The 5-, 3-, and 2-fold axes are labeled. (C-D) Density maps (grey) of the major capsid protein gpE and the trimer of the cement protein gpD superimposed on their atomic models (ribbons).

S3 Fig. Density maps (transparent or mesh) of proteins gpB (A), gpW (B), gpFII (C), gpU (D), gpVN (E), gpM (F), gpL (G), and gpJ (H) superimposed on their atomic models.

S4 Fig. Comparison among the portal proteins of lambda, SPP1 (Protein Data Bank [PDB] ID: 7Z4W), T7 (PDB ID: 7BOU), and T4 (PDB ID: 3JA7). The RMSDs between the portal proteins of lambda and SPP1, T7, and T4 are 2.52, 2.48, and 2.03 Å, respectively.

S5 Fig. Portal–capsid interactions in the symmetry-mismatched binding interface. (A) A schematic representation of the portal and the surrounding capsomers. The 12 portal gpB subunits (identified as 1 to 12) are in orange and cyan. The five M subunits (M1 to M5) and five N subunits (N1 to N5) of the major capsid protein gpE are in magenta and dark blue. The red arrowheads indicate salt bridges between the portal and coat subunits. (B) Salt bridges between the portal and coat subunits. The atomic models (ribbon) are superimposed on their density maps (transparent). (C) Superimposition of the twelve portal subunits revealed the structural morphing loop (residues 208-218) in gpB.

S6 Fig. Structural comparison among lambda, T4, 80α, SPP1, RcGTA, and T5 in terms of the N-termini of tail tube proteins (left) and the distal tail proteins (right). All models are shown in

rainbow colors, ranging from blue at the N-termini to red at the C-termini, and the redundant parts are shown in grey.

S7 Fig. Structural comparison between lambda gpJ, gpL, and T5 pb3 (baseplate hub protein). The color code for domains is identical to that in Fig 7G. (A) Structure of gpJ. (B) Structure of T5 BHP pb3. (C) Structure of gpL. (D) Complex of a copy of gpJ, gpL, and gpI in the lambda tail tip. (E) Structural comparison between the C-terminal domain of the lambda gpL (residues154-232) and the N-terminus of the T5 pb3 HDII-insertion domain shows that they are is topologically similar.

S8 Fig. Secondary structure of tape measure protein (gpH) predicted using the PSIPRED (version 4.0) tool.

S9 Fig. Gene arrangement of the counterpart proteins of T5, lambda, and RcGTA. Genes encoding proteins of homologous domains are shown in the same pattern and color. The regions in white are unstructured.

S10 Fig. Comparison between our gpJ structure and predicted gpJ structure. (A) Structure of the gpJ structure predicted by AlphaFold. The color code for domains is identical to that in Fig 7G except that the central fiber and receptor binding domains are in pink and conflower blue, respectively. (B) Comparison of our gpJ structure (cyan) and predicted gpJ structure (magenta) shows the conformational change between them.

S11 Fig. Ultrathin sections images of the lambda infected E. coli cells. The scale bars represent 200 nm.

S12 Fig. Tail tip reconstructed using RELION. First, we manually selected a total of 70,745 tail tip particles (A, B) and performed 2D classification to exclude irrelevant images (C). Then, using a randomly generated cylinder density as the initial model (D), we selected a total of 58,215 particles from the 2D classifications to perform 3D classification with a C3 symmetry and obtained a total of six types of low-resolution structures (E). Third, we re-extracted tail tip images (F) according to the centers of each type. Fourth, we performed 2D classification (G) and 3D auto-refinement with a C3 symmetry to obtain a density map of the tail tip complex at a resolution of 3.72 Å (H). Finally, we refined the contrast transfer function to improve the resolution of the tail tip structure to 3.44 Å (I).

S1 Table. Modeled proteins of the siphophage lambda.

S2 Table. Detected proteins of the siphophage lambda by mass spectrometry.

S3 Table. Results of the HHpred analysis of the tail protein sequences of lambda phage. For

each protein, the most relevant hits are shown with the matched residues, Protein Data Bank ID, chain identifier, HHpred probability (%), E-value, and percent sequence identity in the matched regions.

S4 Table. Refinement and model statistics.

S1 Appendix. The wwwPDB EM validation reports of lambda portal-capsid, portal-adaptor, neck, tail tube, and tail tip complex.
