# Supplementary material for: Structure of the siphophage neck–Tail complex suggests that conserved tail tip proteins facilitate receptor binding and tail assembly
Source: PLoS Biol. 2023 Dec 14;21(12):e3002441. doi: 10.1371/journal.pbio.3002441 (PMC10721106; doi:10.1371/journal.pbio.3002441)
Supplement: S1 Appendix — (ZIP) [file pbio.3002441.s021.zip › PDB Validation Report.zip!/D_1300038908_report-neck.pdf]

Full wwPDB EM Validation Report (i)

Jul 19, 2023 – 04:46 PM JST

PDB ID	:	8K37
EMDB ID	:	EMD-36846
Title	:	Structure of the bacteriophage lambda neck
Deposited on	:	2023-07-14
Resolution	:	3.50 Å(reported)

This wwPDB validation report is for manuscript review

This is a Full wwPDB EM Validation Report.

This report is produced by the wwPDB biocuration pipeline after annotation of the structure.

We welcome your comments at *validation@mail.wwpdb.org*
A user guide is available at
https://www.wwpdb.org/validation/2017/EMValidationReportHelp
with specific help available everywhere you see the (i) symbol.

The types of validation reports are described at
http://www.wwpdb.org/validation/2017/FAQs#types.

For Manuscript Review

The following versions of software and data (see references (i)) were used in the production of this report:

EMDB validation analysis	:	0.0.1.dev50
MolProbity	:	4.02b-467
Percentile statistics	:	20191225.v01 (using entries in the PDB archive December 25th 2019)
MapQ	:	1.9.9
Ideal geometry (proteins)	:	Engh & Huber (2001)
Ideal geometry (DNA, RNA)	:	Parkinson et al. (1996)
Validation Pipeline (wwPDB-VP)	:	2.34

1 Overall quality at a glance (i)

The following experimental techniques were used to determine the structure:
ELECTRON MICROSCOPY

The reported resolution of this entry is 3.50 Å.

Percentile scores (ranging between 0-100) for global validation metrics of the entry are shown in the following graphic. The table shows the number of entries on which the scores are based.

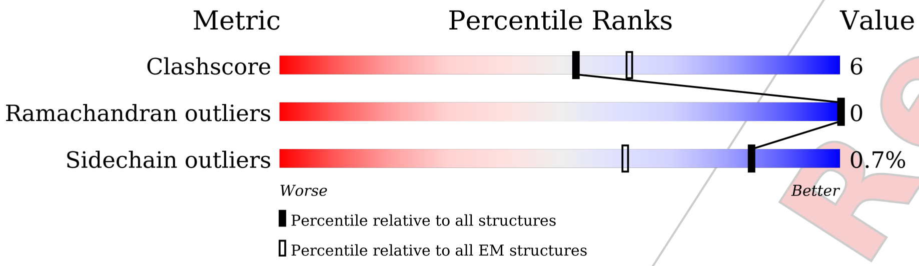

Metric	Whole archive (#Entries)	EM structures (#Entries)
Clashscore	158937	4297
Ramachandran outliers	154571	4023
Sidechain outliers	154315	3826

The table below summarises the geometric issues observed across the polymeric chains and their fit to the map. The red, orange, yellow and green segments of the bar indicate the fraction of residues that contain outliers for >=3, 2, 1 and 0 types of geometric quality criteria respectively. A grey segment represents the fraction of residues that are not modelled. The numeric value for each fraction is indicated below the corresponding segment, with a dot representing fractions <=5% The upper red bar (where present) indicates the fraction of residues that have poor fit to the EM map (all-atom inclusion < 40%). The numeric value is given above the bar.

Mol	Chain	Length	Quality of chain
1	G	246	• 54% 9% 37%
1	H	246	• 56% 7% 37%
1	I	246	• 55% 7% 37%
1	J	246	• 54% 9% 37%
1	K	246	• 56% 7% 37%
1	L	246	• 54% 9% 37%
2	A	131	• 79% 21%
2	B	131	• 78% 21% •

Continued on next page...

For Manuscript Review

Continued from previous page...

Mol	Chain	Length	Quality of chain
2	C	131	76% 24%
2	D	131	81% 19%
2	E	131	80% 20%
2	F	131	84% 16%
3	M	117	89% 9% •
3	N	117	85% 12% •
3	O	117	84% 14% •
3	P	117	85% 12% •
3	Q	117	85% 12% •
3	R	117	87% 10% •

For Manuscript Review

2 Entry composition (i)

There are 3 unique types of molecules in this entry. The entry contains 18528 atoms, of which 0 are hydrogens and 0 are deuteriums.

In the tables below, the AltConf column contains the number of residues with at least one atom in alternate conformation and the Trace column contains the number of residues modelled with at most 2 atoms.

- Molecule 1 is a protein called Tail tube protein.

Mol	Chain	Residues	Atoms					AltConf	Trace
			Total	C	N	O	S		
1	G	154	1181	741	200	237	3	0	0
1	H	154	1181	741	200	237	3	0	0
1	I	154	1181	741	200	237	3	0	0
1	J	154	1181	741	200	237	3	0	0
1	K	154	1181	741	200	237	3	0	0
1	L	154	1181	741	200	237	3	0	0

- Molecule 2 is a protein called Tail tube terminator protein.

Mol	Chain	Residues	Atoms					AltConf	Trace
			Total	C	N	O	S		
2	A	131	1031	655	159	212	5	0	0
2	B	131	1031	655	159	212	5	0	0
2	C	131	1031	655	159	212	5	0	0
2	D	131	1031	655	159	212	5	0	0
2	E	131	1031	655	159	212	5	0	0
2	F	131	1031	655	159	212	5	0	0

- Molecule 3 is a protein called Head-tail connector protein FII.

Mol	Chain	Residues	Atoms					AltConf	Trace
			Total	C	N	O	S		
3	M	114	876	537	164	173	2	0	0

Continued on next page...

For Manuscript Review

Continued from previous page...

Mol	Chain	Residues	Atoms					AltConf	Trace
			Total	C	N	O	S		
3	N	114	876	537	164	173	2	0	0
3	O	114	876	537	164	173	2	0	0
3	P	114	876	537	164	173	2	0	0
3	Q	114	876	537	164	173	2	0	0
3	R	114	876	537	164	173	2	0	0

For Manuscript Review

3 Residue-property plots ⓘ

These plots are drawn for all protein, RNA, DNA and oligosaccharide chains in the entry. The first graphic for a chain summarises the proportions of the various outlier classes displayed in the second graphic. The second graphic shows the sequence view annotated by issues in geometry and atom inclusion in map density. Residues are color-coded according to the number of geometric quality criteria for which they contain at least one outlier: green = 0, yellow = 1, orange = 2 and red = 3 or more. A red diamond above a residue indicates a poor fit to the EM map for this residue (all-atom inclusion < 40%). Stretches of 2 or more consecutive residues without any outlier are shown as a green connector. Residues present in the sample, but not in the model, are shown in grey.

- Molecule 1: Tail tube protein

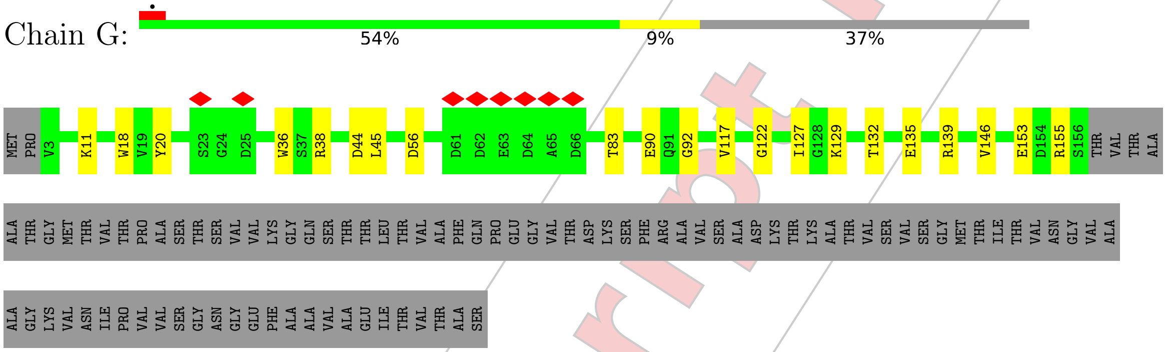

- Molecule 1: Tail tube protein

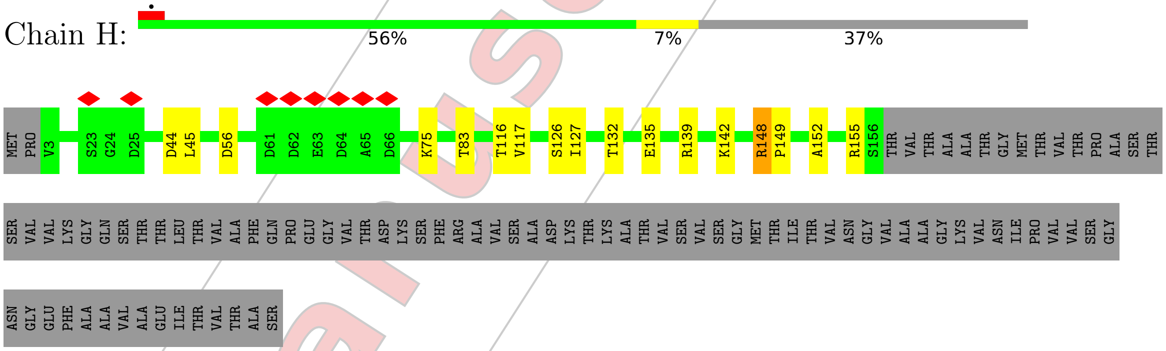

- Molecule 1: Tail tube protein

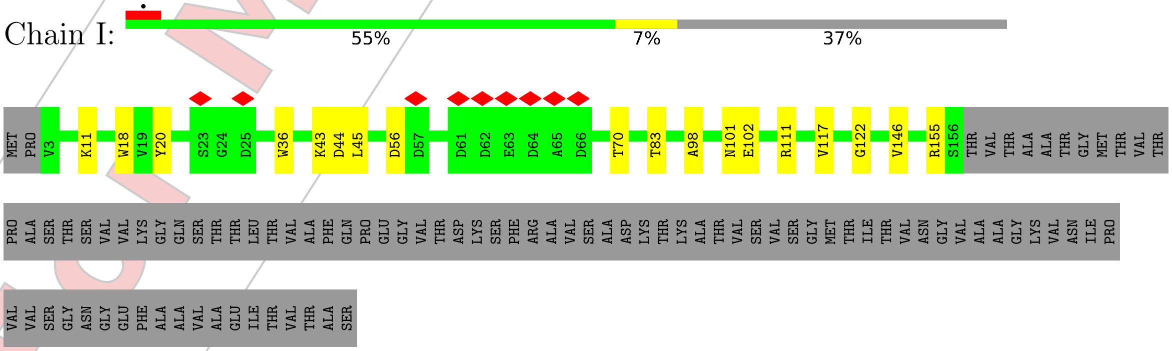

For Manuscript Review

• Molecule 1: Tail tube protein

Chain J: 54% 9% 37%

MET PRO V3 W18 V19 Y20 S23 G24 D25 W36 S37 R38 K43 D44 L45 D56 D61 D62 E63 D64 A65 D66 T70 T83 A98 E102 V117 G122 I127 T132 A133 K134 E135 R139 V146 E153 D154 R155 S156 THR VAL THR ALA

ALA THR GLY MET THR VAL THR PRO ALA SER THR SER VAL VAL LYS GLY GLN SER THR THR LEU THR VAL ALA PHE GLN PRO GLU GLY VAL THR ASP LYS SER PHE ARG ALA VAL SER ALA ASP LYS THR LYS ALA THR VAL SER VAL SER GLY MET THR ILE THR VAL ASN GLY VAL ALA

ALA GLY LYS VAL ASN ILE PRO VAL VAL SER GLY ASN GLY GLU PHE ALA ALA VAL ALA GLU ILE THR VAL THR ALA SER

• Molecule 1: Tail tube protein

Chain K: 56% 7% 37%

MET PRO V3 S23 G24 D25 D44 L45 D56 D61 D62 E63 D64 A65 D66 W67 K75 T83 V117 S126 I127 T132 E135 R139 K142 V146 E153 D154 R155 S156 THR VAL THR ALA ALA THR GLY MET THR VAL THR PRO ALA SER THR SER

VAL VAL LYS GLY GLN SER THR THR LEU THR VAL ALA PHE GLN PRO GLU GLY VAL THR ASP LYS SER PHE ARG ALA VAL SER ALA ASP LYS THR LYS ALA THR VAL SER VAL SER GLY MET THR ILE THR VAL ASN GLY VAL ALA ALA GLY LYS VAL ASN ILE PRO VAL VAL SER GLY ASN

GLY GLU PHE ALA ALA VAL ALA GLU ILE THR VAL THR ALA SER

• Molecule 1: Tail tube protein

Chain L: 54% 9% 37%

MET PRO V3 W18 V19 Y20 K21 G22 S23 G24 D25 W36 S37 R38 D44 L45 D56 D57 D61 D62 E63 D64 A65 D66 T70 T83 A98 N101 E102 R111 V117 G122 K134 V146 G147 R148 R155 S156 THR VAL THR ALA ALA THR GLY

MET THR VAL THR PRO ALA SER THR SER VAL VAL LYS GLY GLN SER THR THR LEU THR VAL ALA PHE GLN PRO GLU GLY VAL THR ASP LYS SER PHE ARG ALA VAL SER ALA ASP LYS THR LYS ALA THR VAL SER VAL SER GLY MET THR ILE THR VAL ASN GLY VAL ALA ALA GLY LYS

VAL ASN ILE PRO VAL VAL SER GLY ASN GLY GLU PHE ALA ALA VAL ALA GLU ILE THR VAL THR ALA SER

• Molecule 2: Tail tube terminator protein

Chain A: 79% 21%

M1 D18 T22 P28 A29 V30 E33 A34 D35 F36 V39 E52 S55 D56 V66 F67 L68 Q71 V72 P73 D74 S75 E76 M81 I92 L95 I99 D108 Y109 R110 R111 D112 D113 D114 W118 L123 I127 E130 M131

• Molecule 2: Tail tube terminator protein

Chain B: 78% 21%

For Manuscript Review

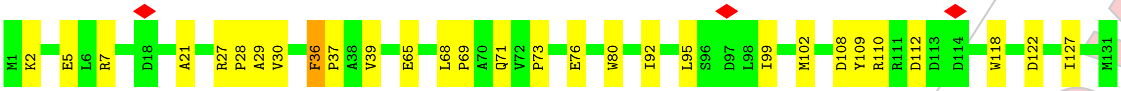

• Molecule 2: Tail tube terminator protein

Chain C:

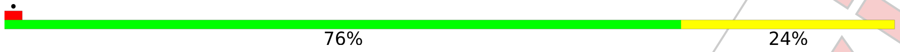

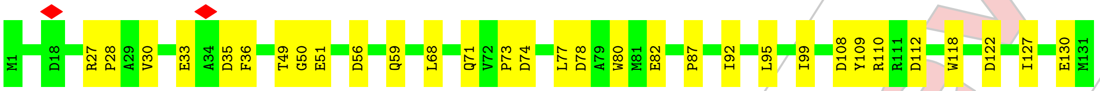

• Molecule 2: Tail tube terminator protein

Chain D:

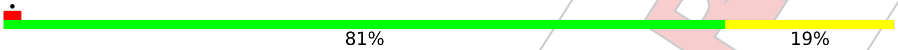

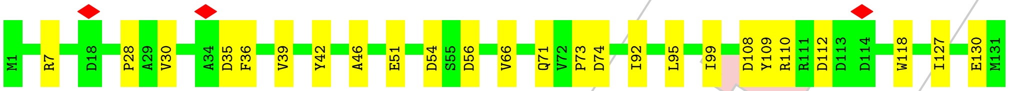

• Molecule 2: Tail tube terminator protein

Chain E:

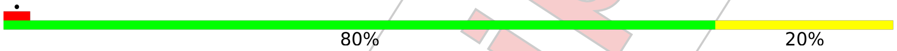

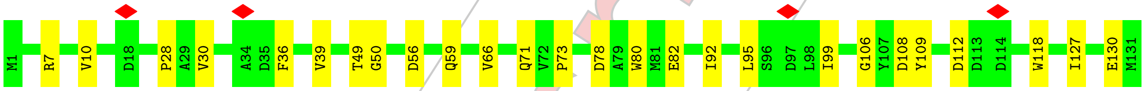

• Molecule 2: Tail tube terminator protein

Chain F:

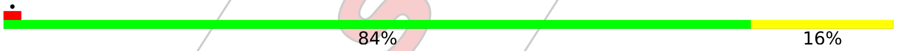

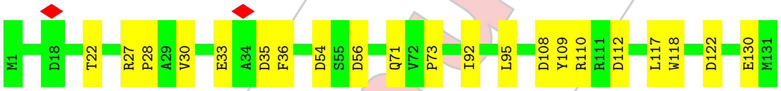

• Molecule 3: Head-tail connector protein FII

Chain M:

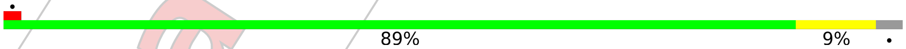

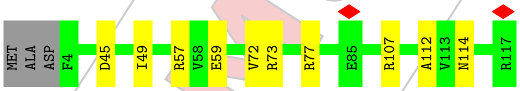

• Molecule 3: Head-tail connector protein FII

Chain N:

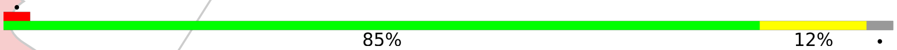

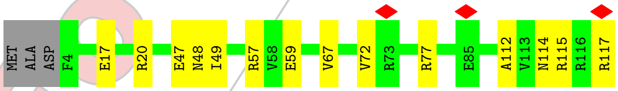

• Molecule 3: Head-tail connector protein FII

For Manuscript Review

Chain O:

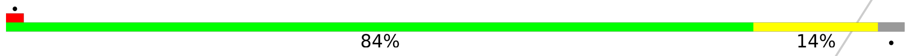

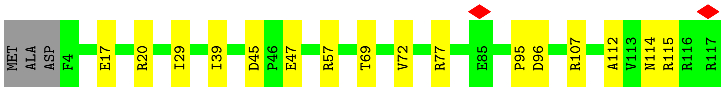

• Molecule 3: Head-tail connector protein FII

Chain P:

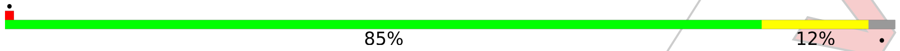

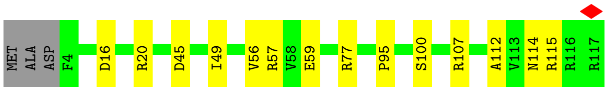

• Molecule 3: Head-tail connector protein FII

Chain Q:

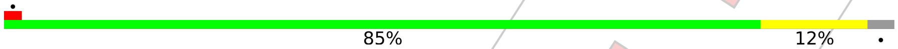

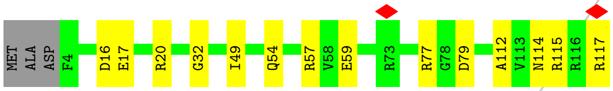

• Molecule 3: Head-tail connector protein FII

Chain R:

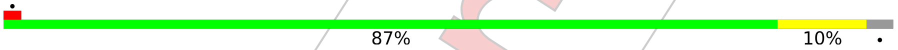

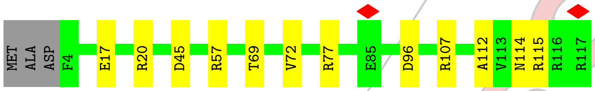

For Manuscript Review

4 Experimental information ⓘ

Property	Value	Source
EM reconstruction method	SINGLE PARTICLE	Depositor
Imposed symmetry	POINT, Not provided	
Number of particles used	75098	Depositor
Resolution determination method	FSC 0.143 CUT-OFF	Depositor
CTF correction method	NONE	Depositor
Microscope	FEI TITAN KRIOS	Depositor
Voltage (kV)	300	Depositor
Electron dose $(e^-/\text{Å}^2)$	32	Depositor
Minimum defocus (nm)	1600	Depositor
Maximum defocus (nm)	2200	Depositor
Magnification	Not provided	
Image detector	GATAN K3 (6k x 4k)	Depositor
Maximum map value	22.078	Depositor
Minimum map value	-11.846	Depositor
Average map value	-0.006	Depositor
Map value standard deviation	1.080	Depositor
Recommended contour level	4.5	Depositor
Map size (Å)	489.6, 489.6, 489.6	wwPDB
Map dimensions	360, 360, 360	wwPDB
Map angles (°)	90.0, 90.0, 90.0	wwPDB
Pixel spacing (Å)	1.36, 1.36, 1.36	Depositor

For Manuscript Review

5 Model quality (i)

5.1 Standard geometry (i)

The Z score for a bond length (or angle) is the number of standard deviations the observed value is removed from the expected value. A bond length (or angle) with $|Z| > 5$ is considered an outlier worth inspection. RMSZ is the root-mean-square of all Z scores of the bond lengths (or angles).

Mol	Chain	Bond lengths		Bond angles	
		RMSZ	#$\|Z\|$ >5	RMSZ	#$\|Z\|$ >5
1	G	0.33	0/1210	0.51	0/1650
1	H	0.33	0/1210	0.52	0/1650
1	I	0.33	0/1210	0.51	0/1650
1	J	0.33	0/1210	0.52	0/1650
1	K	0.33	0/1210	0.51	0/1650
1	L	0.33	0/1210	0.51	0/1650
2	A	0.39	0/1057	0.53	0/1444
2	B	0.38	0/1057	0.59	0/1444
2	C	0.38	0/1057	0.54	0/1444
2	D	0.38	0/1057	0.57	0/1444
2	E	0.38	0/1057	0.54	0/1444
2	F	0.37	0/1057	0.52	0/1444
3	M	0.33	0/892	0.58	0/1208
3	N	0.33	0/892	0.58	0/1208
3	O	0.33	0/892	0.58	0/1208
3	P	0.33	0/892	0.58	0/1208
3	Q	0.33	0/892	0.57	0/1208
3	R	0.34	0/892	0.58	0/1208
All	All	0.35	0/18954	0.54	0/25812

There are no bond length outliers.

There are no bond angle outliers.

There are no chirality outliers.

There are no planarity outliers.

5.2 Too-close contacts (i)

In the following table, the Non-H and H(model) columns list the number of non-hydrogen atoms and hydrogen atoms in the chain respectively. The H(added) column lists the number of hydrogen atoms added and optimized by MolProbity. The Clashes column lists the number of clashes within the asymmetric unit, whereas Symm-Clashes lists symmetry-related clashes.

For Manuscript Review

Mol	Chain	Non-H	H(model)	H(added)	Clashes	Symm-Clashes
1	G	1181	0	1130	14	0
1	H	1181	0	1130	10	0
1	I	1181	0	1130	12	0
1	J	1181	0	1130	17	0
1	K	1181	0	1130	13	0
1	L	1181	0	1130	17	0
2	A	1031	0	959	18	0
2	B	1031	0	959	20	0
2	C	1031	0	959	24	0
2	D	1031	0	959	15	0
2	E	1031	0	959	16	0
2	F	1031	0	959	15	0
3	M	876	0	835	8	0
3	N	876	0	835	11	0
3	O	876	0	835	12	0
3	P	876	0	835	11	0
3	Q	876	0	835	10	0
3	R	876	0	835	9	0
All	All	18528	0	17544	201	0

The all-atom clashscore is defined as the number of clashes found per 1000 atoms (including hydrogen atoms). The all-atom clashscore for this structure is 6.

All (201) close contacts within the same asymmetric unit are listed below, sorted by their clash magnitude.

Atom-1	Atom-2	Interatomic distance (Å)	Clash overlap (Å)
1:H:148:ARG:NH1	1:H:149:PRO:O	2.22	0.71
1:H:44:ASP:OD1	1:H:45:LEU:N	2.25	0.69
2:A:35:ASP:OD2	3:P:115:ARG:NH2	2.25	0.69
1:K:44:ASP:OD1	1:K:45:LEU:N	2.25	0.69
2:F:35:ASP:OD2	3:O:115:ARG:NH2	2.26	0.68
2:B:65:GLU:HG3	2:B:122:ASP:HB3	1.75	0.68
3:P:16:ASP:OD2	3:P:20:ARG:NH2	2.28	0.67
1:G:117:VAL:O	1:G:155:ARG:NH2	2.27	0.67
1:J:117:VAL:O	1:J:155:ARG:NH2	2.28	0.66
2:B:21:ALA:HB1	2:B:37:PRO:HB2	1.77	0.66
1:G:44:ASP:OD1	1:G:45:LEU:N	2.29	0.65
2:F:28:PRO:O	3:O:114:ASN:ND2	2.29	0.65
1:I:44:ASP:OD1	1:I:45:LEU:N	2.29	0.65
2:E:30:VAL:HG11	3:M:57:ARG:HH21	1.61	0.65
1:J:44:ASP:OD1	1:J:45:LEU:N	2.30	0.64
2:A:28:PRO:O	3:P:114:ASN:ND2	2.30	0.64

Continued on next page...

For Manuscript Review

Continued from previous page...

Atom-1	Atom-2	Interatomic distance (Å)	Clash overlap (Å)
2:C:28:PRO:O	3:R:114:ASN:ND2	2.30	0.64
3:Q:16:ASP:OD2	3:Q:20:ARG:NH2	2.30	0.64
2:D:39:VAL:HG22	2:D:66:VAL:HG12	1.78	0.63
2:A:39:VAL:HG22	2:A:66:VAL:HG12	1.81	0.63
1:H:117:VAL:O	1:H:155:ARG:NH2	2.32	0.62
1:L:117:VAL:O	1:L:155:ARG:NH2	2.33	0.62
1:L:134:LYS:HD2	2:E:50:GLY:HA3	1.81	0.62
2:A:92:ILE:HB	2:A:95:LEU:HD12	1.82	0.62
2:B:71:GLN:OE1	3:P:77:ARG:NH2	2.32	0.62
2:E:71:GLN:OE1	3:M:77:ARG:NH2	2.32	0.62
1:K:117:VAL:O	1:K:155:ARG:NH2	2.33	0.61
2:C:71:GLN:OE1	3:Q:77:ARG:NH2	2.33	0.61
2:D:30:VAL:HG11	3:R:57:ARG:HH21	1.65	0.61
1:L:44:ASP:OD1	1:L:45:LEU:N	2.34	0.61
1:I:117:VAL:O	1:I:155:ARG:NH2	2.34	0.60
2:A:74:ASP:HB2	2:F:27:ARG:H	1.66	0.60
2:D:92:ILE:HB	2:D:95:LEU:HD12	1.83	0.60
2:C:35:ASP:OD2	3:R:115:ARG:NH2	2.35	0.60
2:C:95:LEU:HG	2:C:99:ILE:HD12	1.82	0.59
2:B:28:PRO:O	3:Q:114:ASN:ND2	2.35	0.58
2:E:28:PRO:O	3:N:114:ASN:ND2	2.37	0.58
2:F:71:GLN:OE1	3:N:77:ARG:NH2	2.37	0.57
2:E:95:LEU:HD22	2:E:99:ILE:HD12	1.85	0.57
1:K:44:ASP:HB3	1:K:83:THR:HG22	1.87	0.57
1:L:44:ASP:HB3	1:L:83:THR:HG22	1.86	0.57
2:F:92:ILE:HB	2:F:95:LEU:HD12	1.87	0.56
2:C:99:ILE:HD13	2:C:127:ILE:HD12	1.86	0.56
1:I:18:TRP:NE1	1:I:111:ARG:HD3	2.21	0.56
1:H:44:ASP:HB3	1:H:83:THR:HG22	1.88	0.56
1:G:44:ASP:HB3	1:G:83:THR:HG22	1.88	0.56
2:B:73:PRO:HG2	3:P:112:ALA:HB3	1.87	0.56
2:B:95:LEU:HD22	2:B:99:ILE:HD12	1.87	0.55
2:B:30:VAL:HG11	3:P:57:ARG:HH21	1.71	0.55
1:G:122:GLY:HA2	1:G:146:VAL:HG12	1.89	0.55
1:J:38:ARG:NH2	2:D:54:ASP:OD2	2.39	0.55
1:H:116:THR:HB	1:H:152:ALA:HB1	1.88	0.55
1:J:44:ASP:HB3	1:J:83:THR:HG22	1.88	0.55
2:E:99:ILE:HD13	2:E:127:ILE:HD12	1.88	0.55
1:L:18:TRP:NE1	1:L:111:ARG:HD3	2.21	0.54
1:J:122:GLY:HA2	1:J:146:VAL:HG12	1.90	0.54
1:L:21:LYS:HD3	1:L:37:SER:HB2	1.89	0.54

Continued on next page...

For Manuscript Review

Continued from previous page...

Atom-1	Atom-2	Interatomic distance (Å)	Clash overlap (Å)
3:N:49:ILE:HG22	3:N:59:GLU:HA	1.89	0.54
1:I:44:ASP:HB3	1:I:83:THR:HG22	1.90	0.54
2:A:71:GLN:OE1	3:O:77:ARG:NH2	2.41	0.54
1:H:132:THR:HB	1:H:135:GLU:HB2	1.90	0.53
2:E:73:PRO:HG2	3:M:112:ALA:HB3	1.89	0.53
2:A:56:ASP:O	2:A:130:GLU:HG3	2.09	0.53
2:D:71:GLN:OE1	3:R:77:ARG:NH2	2.42	0.53
2:B:21:ALA:CB	2:B:37:PRO:HB2	2.39	0.52
2:B:27:ARG:H	2:C:74:ASP:HB2	1.75	0.52
2:F:108:ASP:OD1	2:F:109:TYR:N	2.42	0.52
1:L:122:GLY:HA2	1:L:146:VAL:HG22	1.92	0.52
1:K:67:TRP:CE2	1:L:148:ARG:HG3	2.45	0.52
2:A:99:ILE:HD13	2:A:127:ILE:HD12	1.91	0.51
3:N:17:GLU:OE1	3:N:20:ARG:NH1	2.40	0.51
3:R:45:ASP:OD1	3:R:107:ARG:NH2	2.43	0.51
2:C:108:ASP:OD1	2:C:109:TYR:N	2.42	0.51
3:O:69:THR:HG21	3:O:96:ASP:OD1	2.12	0.50
2:A:33:GLU:HA	2:A:36:PHE:HD1	1.77	0.50
2:A:30:VAL:HG11	3:O:57:ARG:HH21	1.75	0.50
2:A:52:GLU:OE2	2:A:55:SER:OG	2.29	0.50
3:Q:49:ILE:HG22	3:Q:59:GLU:HA	1.94	0.50
1:I:70:THR:H	1:J:146:VAL:HG23	1.78	0.49
2:C:30:VAL:HG11	3:Q:57:ARG:HH21	1.77	0.49
1:I:56:ASP:N	1:I:56:ASP:OD1	2.46	0.49
1:I:122:GLY:HA2	1:I:146:VAL:HG22	1.94	0.49
2:D:28:PRO:O	3:M:114:ASN:ND2	2.42	0.49
3:P:45:ASP:OD1	3:P:107:ARG:NH2	2.39	0.49
2:D:56:ASP:O	2:D:130:GLU:HG3	2.12	0.49
1:G:146:VAL:HG23	1:L:70:THR:H	1.78	0.49
2:D:99:ILE:HD13	2:D:127:ILE:HD12	1.94	0.49
1:J:20:TYR:HB2	1:J:36:TRP:CZ3	2.48	0.49
2:C:49:THR:OG1	2:C:59:GLN:HG3	2.12	0.49
1:J:98:ALA:O	1:J:102:GLU:HG3	2.12	0.48
2:A:108:ASP:OD1	2:A:109:TYR:N	2.46	0.48
1:L:56:ASP:N	1:L:56:ASP:OD1	2.45	0.48
2:E:78:ASP:O	2:E:82:GLU:HG2	2.13	0.48
2:F:30:VAL:HG11	3:N:57:ARG:HH21	1.78	0.48
3:O:17:GLU:OE2	3:O:20:ARG:NH1	2.43	0.48
3:Q:32:GLY:HA3	3:Q:79:ASP:OD1	2.14	0.48
1:J:43:LYS:NZ	2:D:51:GLU:OE2	2.34	0.48
3:M:49:ILE:HG22	3:M:59:GLU:HA	1.96	0.48

Continued on next page...

For Manuscript Review

Continued from previous page...

Atom-1	Atom-2	Interatomic distance (Å)	Clash overlap (Å)
1:J:134:LYS:HZ2	2:C:50:GLY:HA3	1.79	0.48
1:G:20:TYR:HB2	1:G:36:TRP:CZ3	2.49	0.48
2:A:73:PRO:HG2	3:O:112:ALA:HB3	1.95	0.48
2:B:92:ILE:HB	2:B:95:LEU:HD12	1.95	0.48
1:J:132:THR:HB	1:J:135:GLU:HB2	1.96	0.47
2:B:99:ILE:HD13	2:B:127:ILE:HD12	1.96	0.47
2:C:33:GLU:N	2:C:33:GLU:OE1	2.47	0.47
2:C:33:GLU:HA	2:C:36:PHE:HD1	1.78	0.47
3:O:45:ASP:OD1	3:O:107:ARG:NH2	2.47	0.47
2:F:73:PRO:HG2	3:N:112:ALA:HB3	1.96	0.47
1:G:56:ASP:OD1	1:G:56:ASP:N	2.47	0.47
1:H:126:SER:HB3	1:H:142:LYS:HB2	1.97	0.47
2:D:108:ASP:OD1	2:D:109:TYR:N	2.48	0.47
1:H:56:ASP:N	1:H:56:ASP:OD1	2.47	0.47
1:I:98:ALA:O	1:I:102:GLU:HG3	2.14	0.47
2:A:22:THR:HG21	2:A:35:ASP:HB2	1.96	0.47
3:M:73:ARG:HH11	3:M:73:ARG:HG3	1.80	0.47
2:D:73:PRO:HG2	3:R:112:ALA:HB3	1.95	0.47
1:J:18:TRP:CD2	1:J:38:ARG:HG3	2.49	0.47
2:B:68:LEU:HD13	2:B:76:GLU:OE1	2.15	0.47
3:N:72:VAL:O	3:N:72:VAL:HG12	2.15	0.46
1:K:126:SER:HB3	1:K:142:LYS:HB2	1.97	0.46
2:B:7:ARG:NH2	2:C:78:ASP:OD2	2.48	0.46
3:R:17:GLU:OE2	3:R:20:ARG:NH1	2.43	0.46
2:B:112:ASP:HB2	2:B:118:TRP:CE2	2.51	0.46
2:C:82:GLU:O	2:C:87:PRO:HD3	2.16	0.46
2:A:33:GLU:HA	2:A:36:PHE:CD1	2.50	0.46
2:C:27:ARG:H	2:D:74:ASP:HB2	1.80	0.46
2:E:108:ASP:OD1	2:E:109:TYR:N	2.48	0.46
3:N:47:GLU:HB3	3:O:95:PRO:HG2	1.98	0.46
2:B:108:ASP:OD1	2:B:109:TYR:N	2.49	0.46
3:R:69:THR:HG21	3:R:96:ASP:OD1	2.16	0.46
1:K:56:ASP:OD1	1:K:56:ASP:N	2.47	0.45
2:C:92:ILE:HB	2:C:95:LEU:HB2	1.98	0.45
1:G:132:THR:HB	1:G:135:GLU:HB2	1.98	0.45
1:J:56:ASP:N	1:J:56:ASP:OD1	2.47	0.45
2:B:2:LYS:HB2	2:B:5:GLU:OE1	2.15	0.45
2:F:56:ASP:O	2:F:130:GLU:HG3	2.15	0.45
3:O:47:GLU:HB3	3:P:95:PRO:HG2	1.98	0.45
1:I:43:LYS:HE3	2:C:51:GLU:OE2	2.15	0.45
1:K:67:TRP:NE1	1:L:148:ARG:HG3	2.32	0.45

Continued on next page...

For Manuscript Review

Continued from previous page...

Atom-1	Atom-2	Interatomic distance (Å)	Clash overlap (Å)
2:F:117:LEU:HD11	3:N:48:ASN:OD1	2.15	0.45
2:C:73:PRO:HG2	3:Q:112:ALA:HB3	1.99	0.45
2:A:112:ASP:HB2	2:A:118:TRP:CE2	2.52	0.45
2:C:68:LEU:HD12	2:C:77:LEU:HD23	1.97	0.45
2:E:39:VAL:HG22	2:E:66:VAL:HG23	1.99	0.45
3:Q:17:GLU:OE2	3:Q:20:ARG:NH1	2.49	0.45
1:J:153:GLU:OE1	1:J:153:GLU:N	2.50	0.45
2:C:56:ASP:O	2:C:130:GLU:HG3	2.17	0.45
3:M:45:ASP:OD1	3:M:107:ARG:NH2	2.42	0.44
1:L:20:TYR:HB2	1:L:36:TRP:CZ3	2.53	0.44
3:O:29:ILE:HD11	3:O:39:ILE:HD12	1.98	0.44
2:B:36:PHE:CD1	2:B:69:PRO:HG3	2.52	0.44
2:D:112:ASP:HB2	2:D:118:TRP:CE2	2.53	0.44
1:G:153:GLU:N	1:G:153:GLU:OE1	2.50	0.44
2:E:92:ILE:HB	2:E:95:LEU:HD12	2.00	0.44
2:E:7:ARG:HA	2:E:10:VAL:HG12	1.98	0.44
2:A:68:LEU:HD13	2:A:76:GLU:OE2	2.18	0.43
2:A:81:MET:HG3	2:A:123:LEU:HD12	1.99	0.43
1:J:70:THR:H	1:K:146:VAL:HG23	1.82	0.43
2:F:33:GLU:HA	2:F:36:PHE:HD1	1.83	0.43
2:C:112:ASP:HB2	2:C:118:TRP:CE2	2.54	0.43
2:C:122:ASP:OD1	2:C:122:ASP:N	2.52	0.43
2:B:27:ARG:HD2	2:B:27:ARG:HA	1.87	0.43
1:J:18:TRP:CE2	1:J:38:ARG:HG3	2.54	0.43
1:I:20:TYR:HB2	1:I:36:TRP:CZ3	2.53	0.43
1:G:127:ILE:HG23	1:G:139:ARG:HD2	2.01	0.43
2:E:112:ASP:HB2	2:E:118:TRP:CE2	2.54	0.43
1:G:18:TRP:CZ3	1:G:38:ARG:HB2	2.54	0.42
1:G:11:LYS:HE2	2:B:102:MET:O	2.19	0.42
2:F:22:THR:HG21	2:F:35:ASP:HB2	2.00	0.42
3:P:49:ILE:HG22	3:P:59:GLU:HA	2.00	0.42
1:L:18:TRP:CZ3	1:L:38:ARG:HB2	2.54	0.42
2:F:122:ASP:OD1	2:F:122:ASP:N	2.46	0.42
3:O:72:VAL:HG12	3:O:72:VAL:O	2.20	0.42
2:C:95:LEU:HD12	2:C:95:LEU:HA	1.89	0.42
1:J:127:ILE:HG23	1:J:139:ARG:HD2	2.02	0.42
1:K:62:ASP:N	1:K:62:ASP:OD1	2.53	0.42
1:K:132:THR:HB	1:K:135:GLU:HB2	2.01	0.42
2:C:27:ARG:NH2	3:Q:54:GLN:OE1	2.53	0.41
1:I:11:LYS:HB3	1:I:11:LYS:HE2	1.91	0.41
3:Q:115:ARG:HH11	3:Q:117:ARG:NH2	2.18	0.41

Continued on next page...

For Manuscript Review

Continued from previous page...

Atom-1	Atom-2	Interatomic distance (Å)	Clash overlap (Å)
2:B:29:ALA:O	3:P:56:VAL:HA	2.21	0.41
2:F:112:ASP:HB2	2:F:118:TRP:CE2	2.55	0.41
2:D:46:ALA:O	2:E:106:GLY:HA3	2.20	0.41
2:E:49:THR:OG1	2:E:59:GLN:HG3	2.21	0.41
2:E:56:ASP:O	2:E:130:GLU:HG3	2.20	0.41
2:D:7:ARG:HD3	2:D:42:TYR:HA	2.03	0.41
3:M:72:VAL:HG12	3:M:72:VAL:O	2.20	0.41
3:R:72:VAL:O	3:R:72:VAL:HG12	2.19	0.41
3:P:16:ASP:OD2	3:P:100:SER:OG	2.36	0.41
1:G:90:GLU:HG2	1:G:92:GLY:H	1.86	0.41
1:K:153:GLU:OE1	1:K:153:GLU:N	2.53	0.41
1:H:127:ILE:HG23	1:H:139:ARG:HD2	2.04	0.40
1:K:75:LYS:HE2	1:L:101:ASN:O	2.21	0.40
1:L:38:ARG:NH2	2:F:54:ASP:OD1	2.54	0.40
1:G:129:LYS:HB2	1:G:129:LYS:HE3	1.81	0.40
1:H:75:LYS:HE2	1:I:101:ASN:O	2.21	0.40
1:K:127:ILE:HG23	1:K:139:ARG:HD2	2.03	0.40
1:L:20:TYR:HB2	1:L:36:TRP:CH2	2.57	0.40
3:N:67:VAL:HG21	3:N:72:VAL:HG21	2.03	0.40
3:N:115:ARG:HH11	3:N:117:ARG:NH2	2.19	0.40
1:L:98:ALA:O	1:L:102:GLU:HG3	2.20	0.40

There are no symmetry-related clashes.

5.3 Torsion angles (i)

5.3.1 Protein backbone (i)

In the following table, the Percentiles column shows the percent Ramachandran outliers of the chain as a percentile score with respect to all PDB entries followed by that with respect to all EM entries.

The Analysed column shows the number of residues for which the backbone conformation was analysed, and the total number of residues.

Mol	Chain	Analysed	Favoured	Allowed	Outliers	Percentiles	
1	G	152/246 (62%)	149 (98%)	3 (2%)	0	100	100
1	H	152/246 (62%)	151 (99%)	1 (1%)	0	100	100
1	I	152/246 (62%)	150 (99%)	2 (1%)	0	100	100
1	J	152/246 (62%)	150 (99%)	2 (1%)	0	100	100

Continued on next page...

For Manuscript Review

Continued from previous page...

Mol	Chain	Analysed	Favoured	Allowed	Outliers	Percentiles	
1	K	152/246 (62%)	150 (99%)	2 (1%)	0	100	100
1	L	152/246 (62%)	151 (99%)	1 (1%)	0	100	100
2	A	129/131 (98%)	123 (95%)	6 (5%)	0	100	100
2	B	129/131 (98%)	122 (95%)	7 (5%)	0	100	100
2	C	129/131 (98%)	124 (96%)	5 (4%)	0	100	100
2	D	129/131 (98%)	126 (98%)	3 (2%)	0	100	100
2	E	129/131 (98%)	126 (98%)	3 (2%)	0	100	100
2	F	129/131 (98%)	124 (96%)	5 (4%)	0	100	100
3	M	112/117 (96%)	108 (96%)	4 (4%)	0	100	100
3	N	112/117 (96%)	109 (97%)	3 (3%)	0	100	100
3	O	112/117 (96%)	111 (99%)	1 (1%)	0	100	100
3	P	112/117 (96%)	110 (98%)	2 (2%)	0	100	100
3	Q	112/117 (96%)	108 (96%)	4 (4%)	0	100	100
3	R	112/117 (96%)	110 (98%)	2 (2%)	0	100	100
All	All	2358/2964 (80%)	2302 (98%)	56 (2%)	0	100	100

There are no Ramachandran outliers to report.

5.3.2 Protein sidechains (i)

In the following table, the Percentiles column shows the percent sidechain outliers of the chain as a percentile score with respect to all PDB entries followed by that with respect to all EM entries.

The Analysed column shows the number of residues for which the sidechain conformation was analysed, and the total number of residues.

Mol	Chain	Analysed	Rotameric	Outliers	Percentiles	
1	G	125/196 (64%)	125 (100%)	0	100	100
1	H	125/196 (64%)	124 (99%)	1 (1%)	81	91
1	I	125/196 (64%)	125 (100%)	0	100	100
1	J	125/196 (64%)	125 (100%)	0	100	100
1	K	125/196 (64%)	125 (100%)	0	100	100
1	L	125/196 (64%)	125 (100%)	0	100	100
2	A	109/109 (100%)	108 (99%)	1 (1%)	78	90

Continued on next page...

For Manuscript Review

Continued from previous page...

Mol	Chain	Analysed	Rotameric	Outliers	Percentiles	
2	B	109/109 (100%)	105 (96%)	4 (4%)	34	65
2	C	109/109 (100%)	107 (98%)	2 (2%)	59	81
2	D	109/109 (100%)	106 (97%)	3 (3%)	43	72
2	E	109/109 (100%)	107 (98%)	2 (2%)	59	81
2	F	109/109 (100%)	108 (99%)	1 (1%)	78	90
3	M	92/94 (98%)	92 (100%)	0	100	100
3	N	92/94 (98%)	92 (100%)	0	100	100
3	O	92/94 (98%)	92 (100%)	0	100	100
3	P	92/94 (98%)	92 (100%)	0	100	100
3	Q	92/94 (98%)	92 (100%)	0	100	100
3	R	92/94 (98%)	92 (100%)	0	100	100
All	All	1956/2394 (82%)	1942 (99%)	14 (1%)	84	93

All (14) residues with a non-rotameric sidechain are listed below:

Mol	Chain	Res	Type
1	H	148	ARG
2	A	110	ARG
2	B	36	PHE
2	B	39	VAL
2	B	80	TRP
2	B	110	ARG
2	C	80	TRP
2	C	110	ARG
2	D	35	ASP
2	D	36	PHE
2	D	110	ARG
2	E	36	PHE
2	E	80	TRP
2	F	110	ARG

Sometimes sidechains can be flipped to improve hydrogen bonding and reduce clashes. There are no such sidechains identified.

5.3.3 RNA ⓘ

There are no RNA molecules in this entry.

For Manuscript Review

5.4 Non-standard residues in protein, DNA, RNA chains (i)

There are no non-standard protein/DNA/RNA residues in this entry.

5.5 Carbohydrates (i)

There are no monosaccharides in this entry.

5.6 Ligand geometry (i)

There are no ligands in this entry.

5.7 Other polymers (i)

There are no such residues in this entry.

5.8 Polymer linkage issues (i)

There are no chain breaks in this entry.

For Manuscript Review

6 Map visualisation (i)

This section contains visualisations of the EMDB entry EMD-36846. These allow visual inspection of the internal detail of the map and identification of artifacts.

Images derived from a raw map, generated by summing the deposited half-maps, are presented below the corresponding image components of the primary map to allow further visual inspection and comparison with those of the primary map.

6.1 Orthogonal projections (i)

6.1.1 Primary map

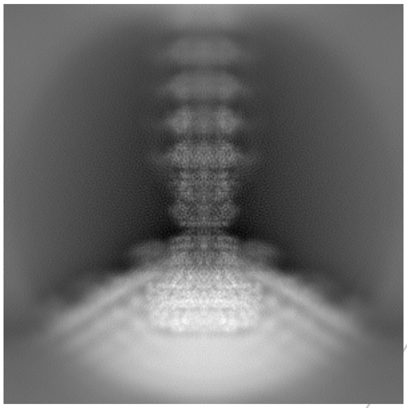

X

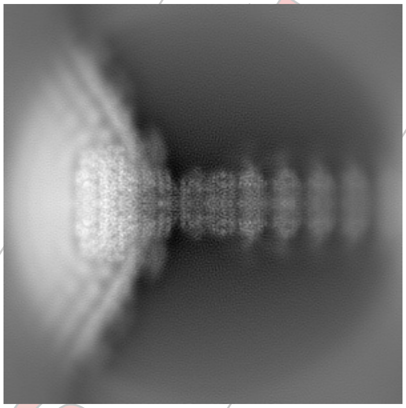

Y

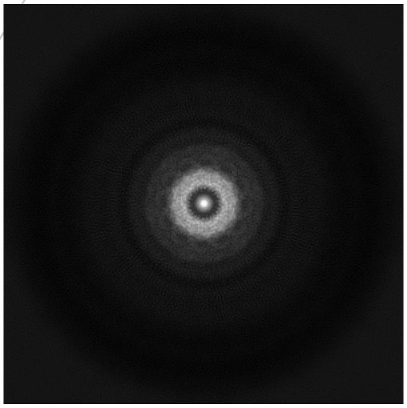

Z

6.1.2 Raw map

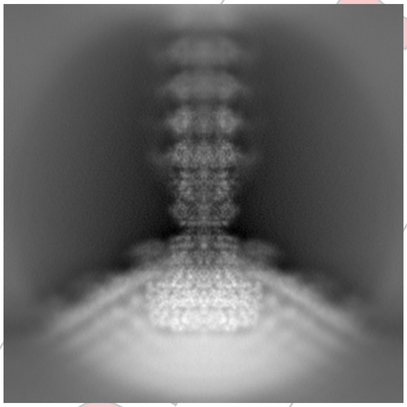

X

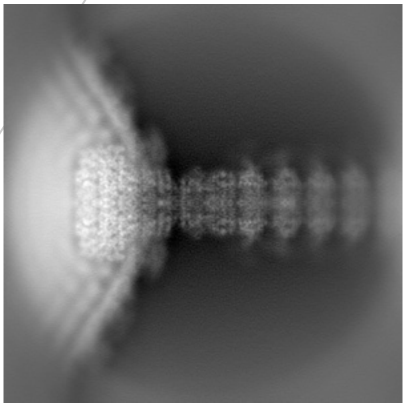

Y

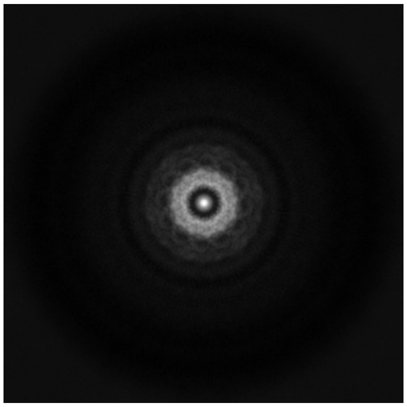

Z

The images above show the map projected in three orthogonal directions.

For Manuscript Review

6.2 Central slices (i)

6.2.1 Primary map

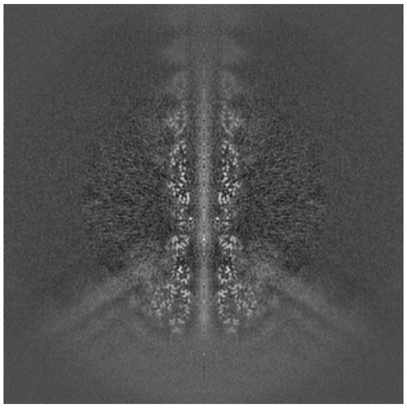
X Index: 180

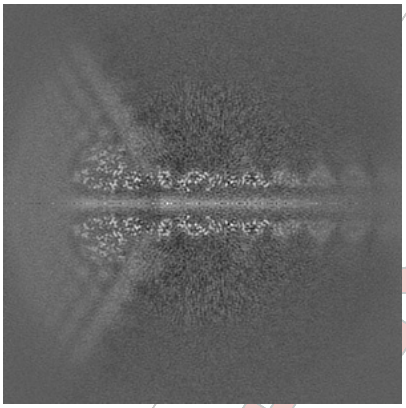
Y Index: 180

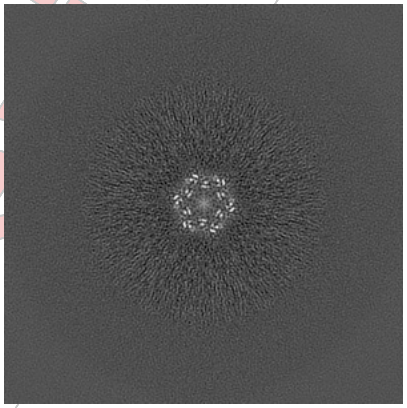
Z Index: 180

6.2.2 Raw map

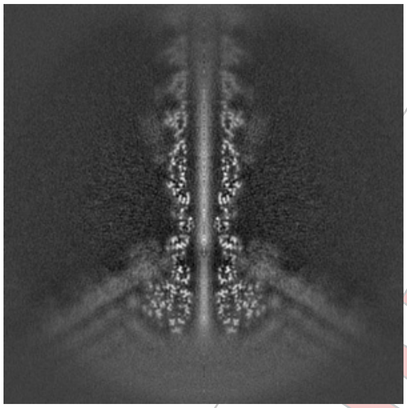
X Index: 180

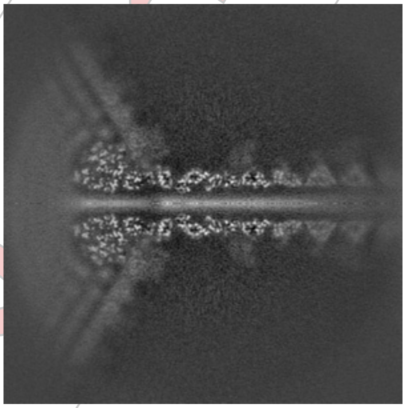
Y Index: 180

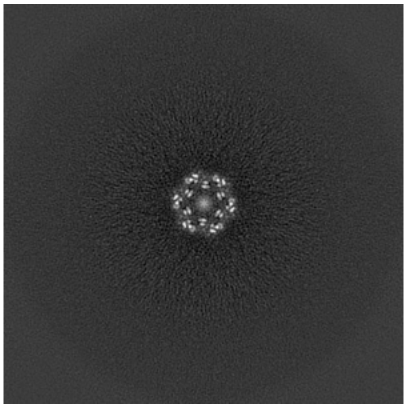
Z Index: 180

The images above show central slices of the map in three orthogonal directions.

For Manuscript Review

6.3 Largest variance slices (i)

6.3.1 Primary map

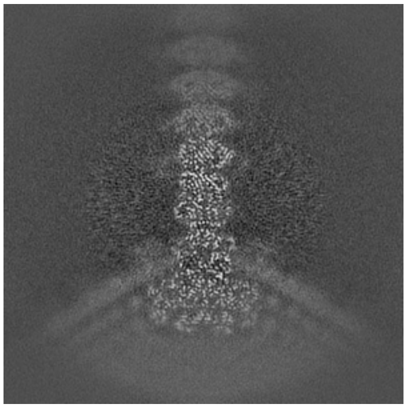
X Index: 163

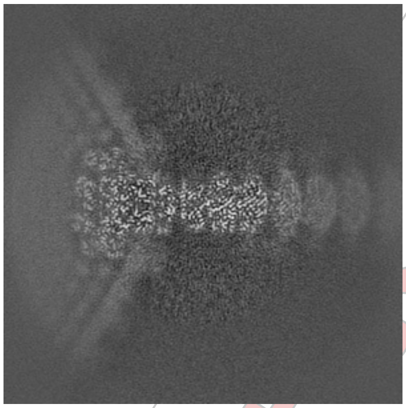
Y Index: 162

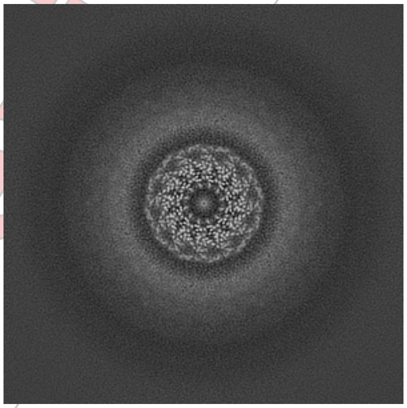
Z Index: 105

6.3.2 Raw map

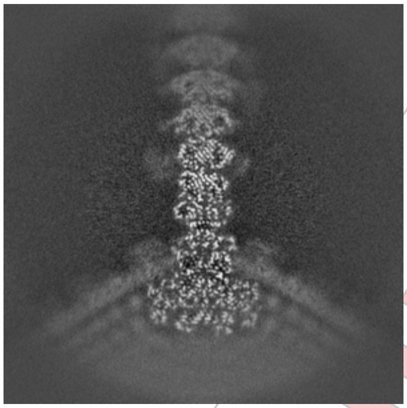
X Index: 163

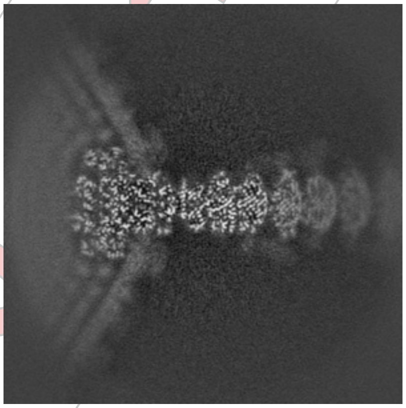
Y Index: 162

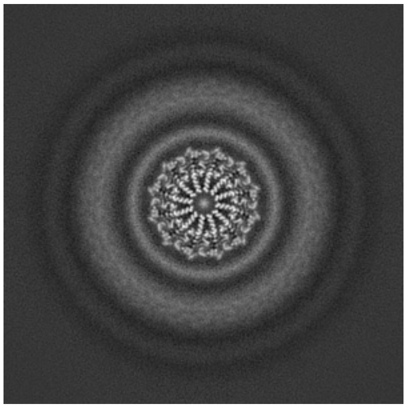
Z Index: 92

The images above show the largest variance slices of the map in three orthogonal directions.

For Manuscript Review

6.4 Orthogonal standard-deviation projections (False-color) (i)

6.4.1 Primary map

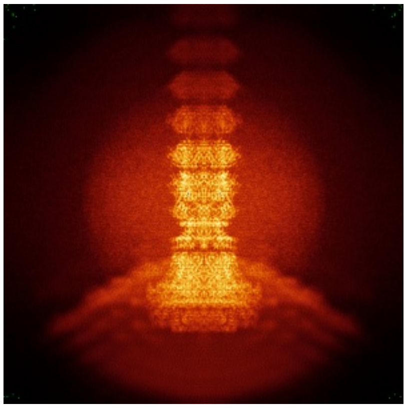
X

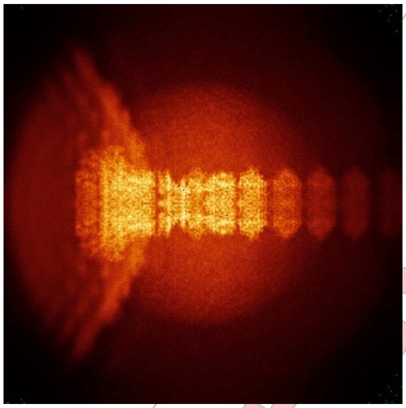
Y

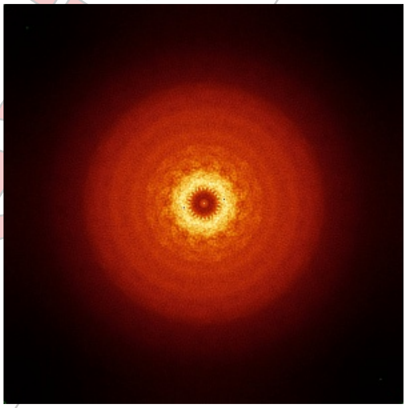
Z

6.4.2 Raw map

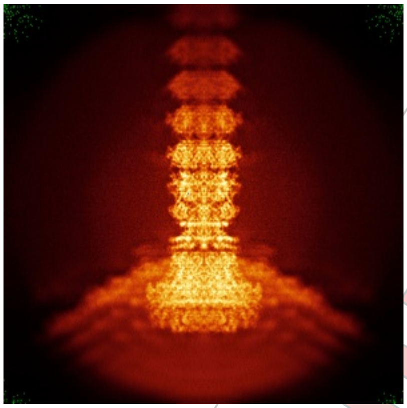
X

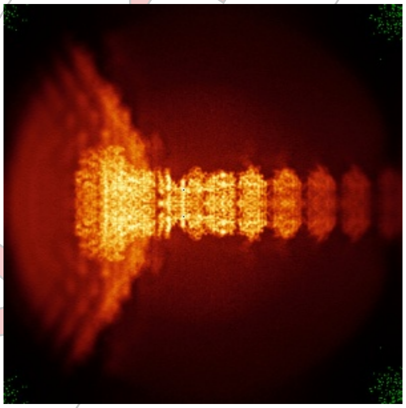
Y

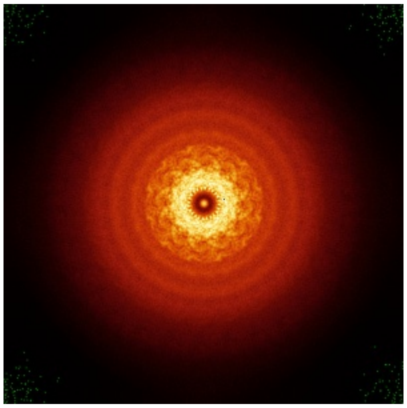
Z

The images above show the map standard deviation projections with false color in three orthogonal directions. Minimum values are shown in green, max in blue, and dark to light orange shades represent small to large values respectively.

For Manuscript Review

6.5 Orthogonal surface views (i)

6.5.1 Primary map

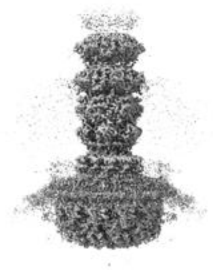

X

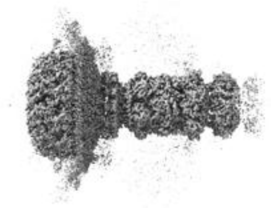

Y

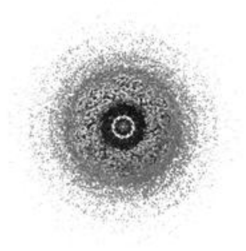

Z

The images above show the 3D surface view of the map at the recommended contour level 4.5. These images, in conjunction with the slice images, may facilitate assessment of whether an appropriate contour level has been provided.

6.5.2 Raw map

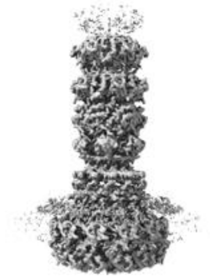

X

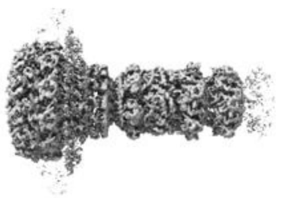

Y

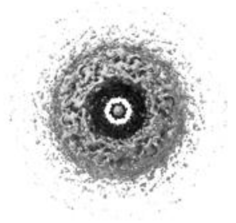

Z

These images show the 3D surface of the raw map. The raw map's contour level was selected so that its surface encloses the same volume as the primary map does at its recommended contour level.

6.6 Mask visualisation (i)

This section was not generated. No masks/segmentation were deposited.

For Manuscript Review

7 Map analysis (i)

This section contains the results of statistical analysis of the map.

7.1 Map-value distribution (i)

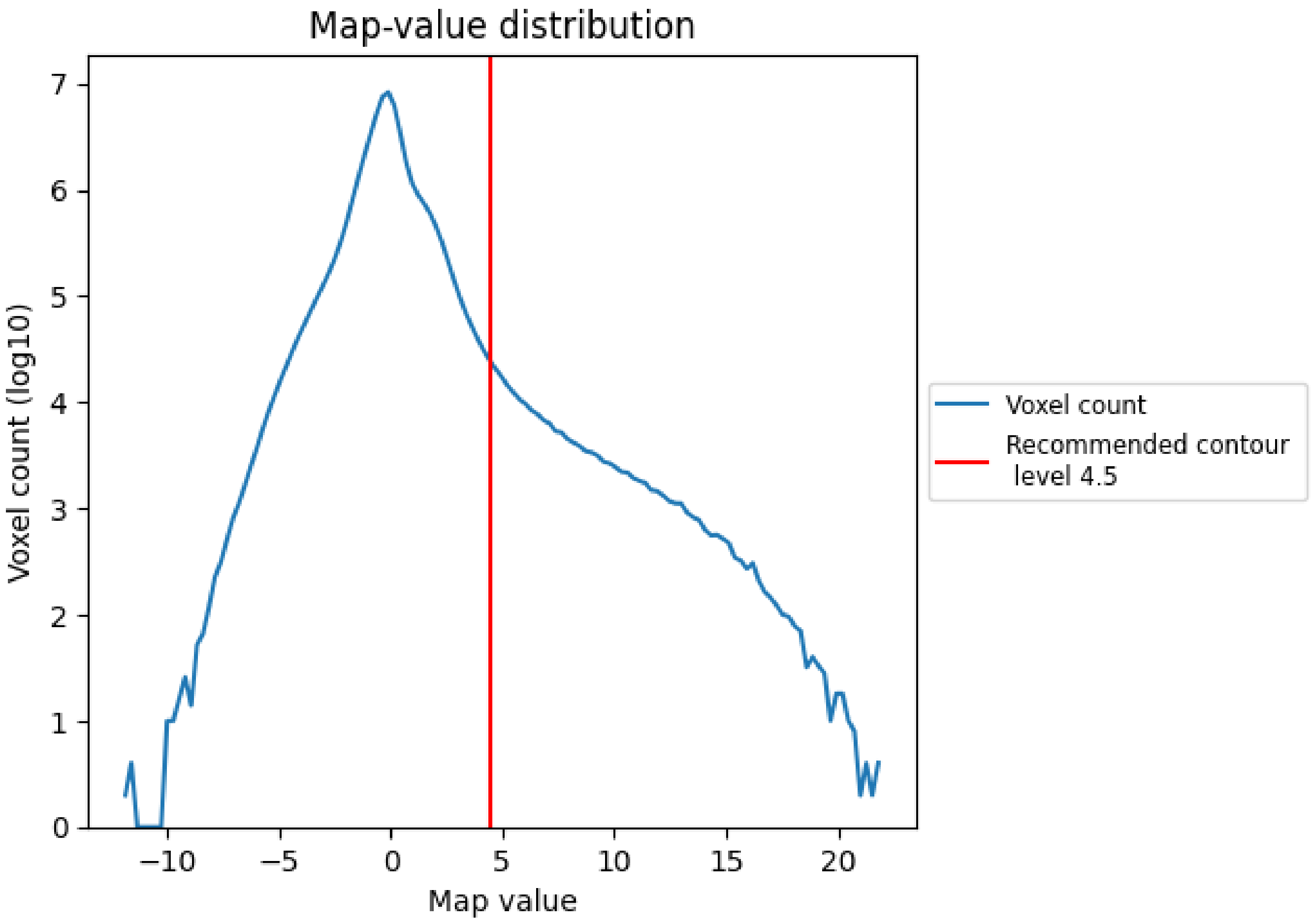

The map-value distribution is plotted in 128 intervals along the x-axis. The y-axis is logarithmic. A spike in this graph at zero usually indicates that the volume has been masked.

For Manuscript Review

7.2 Volume estimate (i)

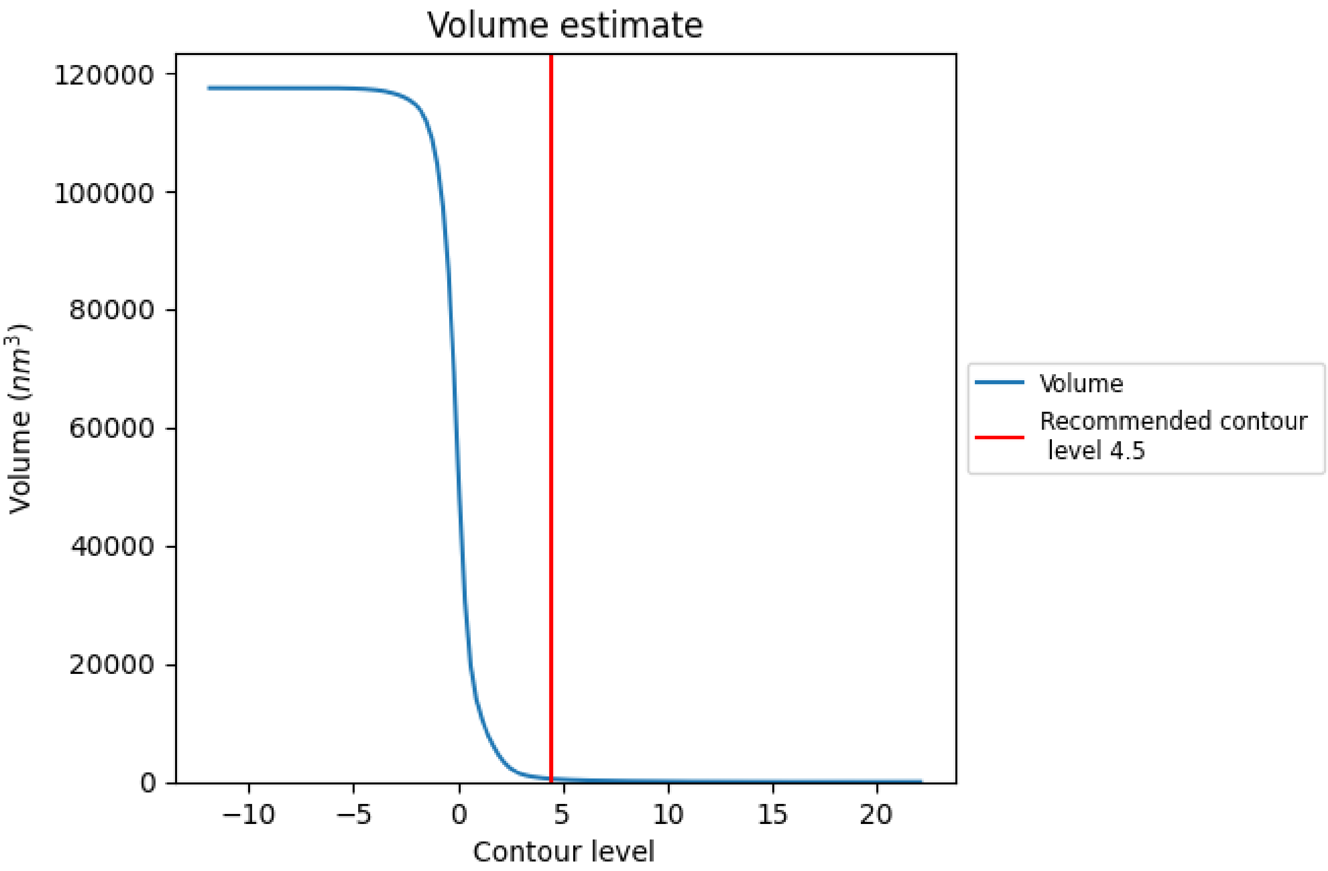

The volume at the recommended contour level is 503 nm^3; this corresponds to an approximate mass of 454 kDa.

The volume estimate graph shows how the enclosed volume varies with the contour level. The recommended contour level is shown as a vertical line and the intersection between the line and the curve gives the volume of the enclosed surface at the given level.

For Manuscript Review

7.3 Rotationally averaged power spectrum ⓘ

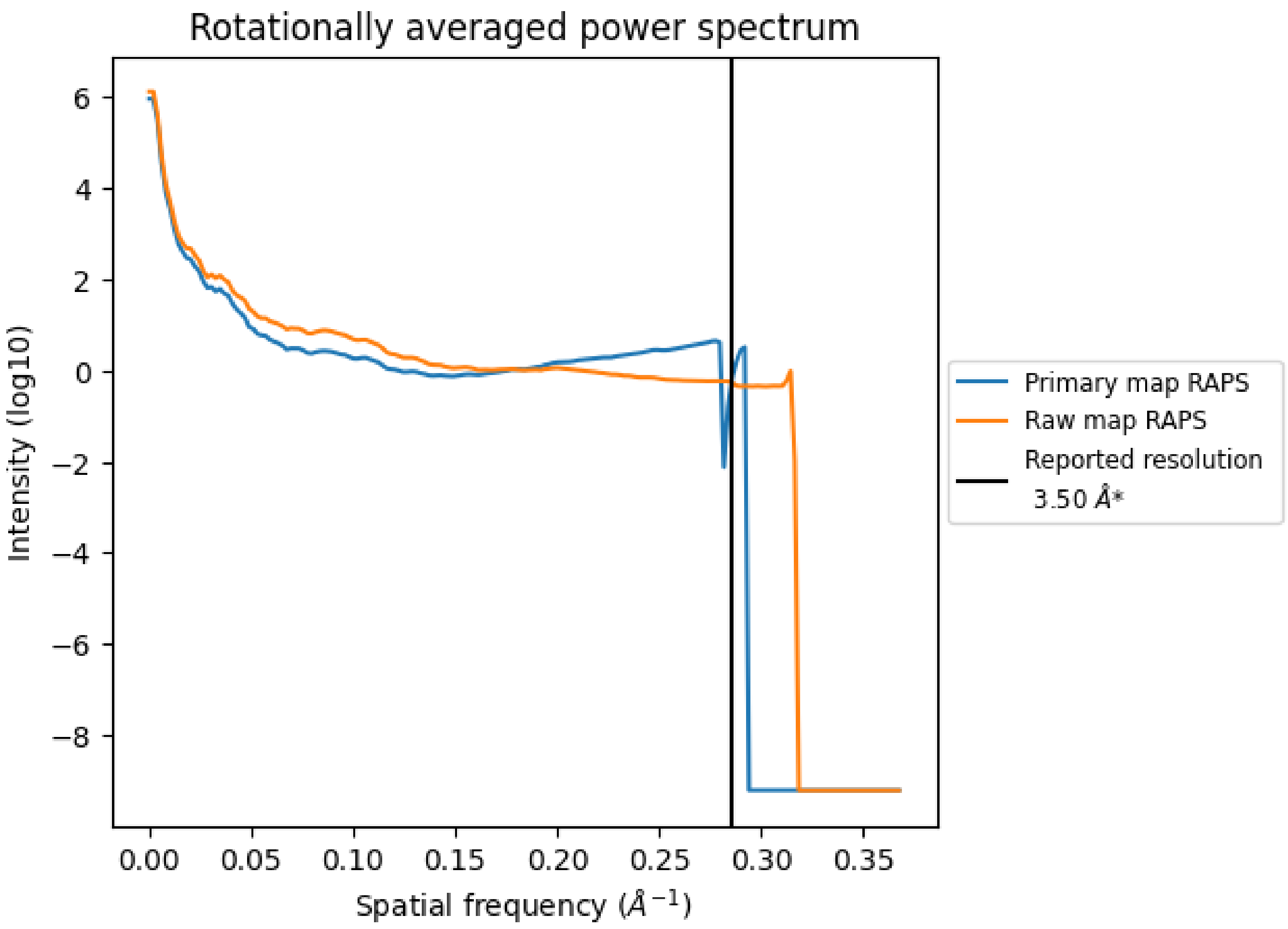

*Reported resolution corresponds to spatial frequency of 0.286 $Å^{-1}$

For Manuscript Review

8 Fourier-Shell correlation (i)

Fourier-Shell Correlation (FSC) is the most commonly used method to estimate the resolution of single-particle and subtomogram-averaged maps. The shape of the curve depends on the imposed symmetry, mask and whether or not the two 3D reconstructions used were processed from a common reference. The reported resolution is shown as a black line. A curve is displayed for the half-bit criterion in addition to lines showing the 0.143 gold standard cut-off and 0.5 cut-off.

8.1 FSC (i)

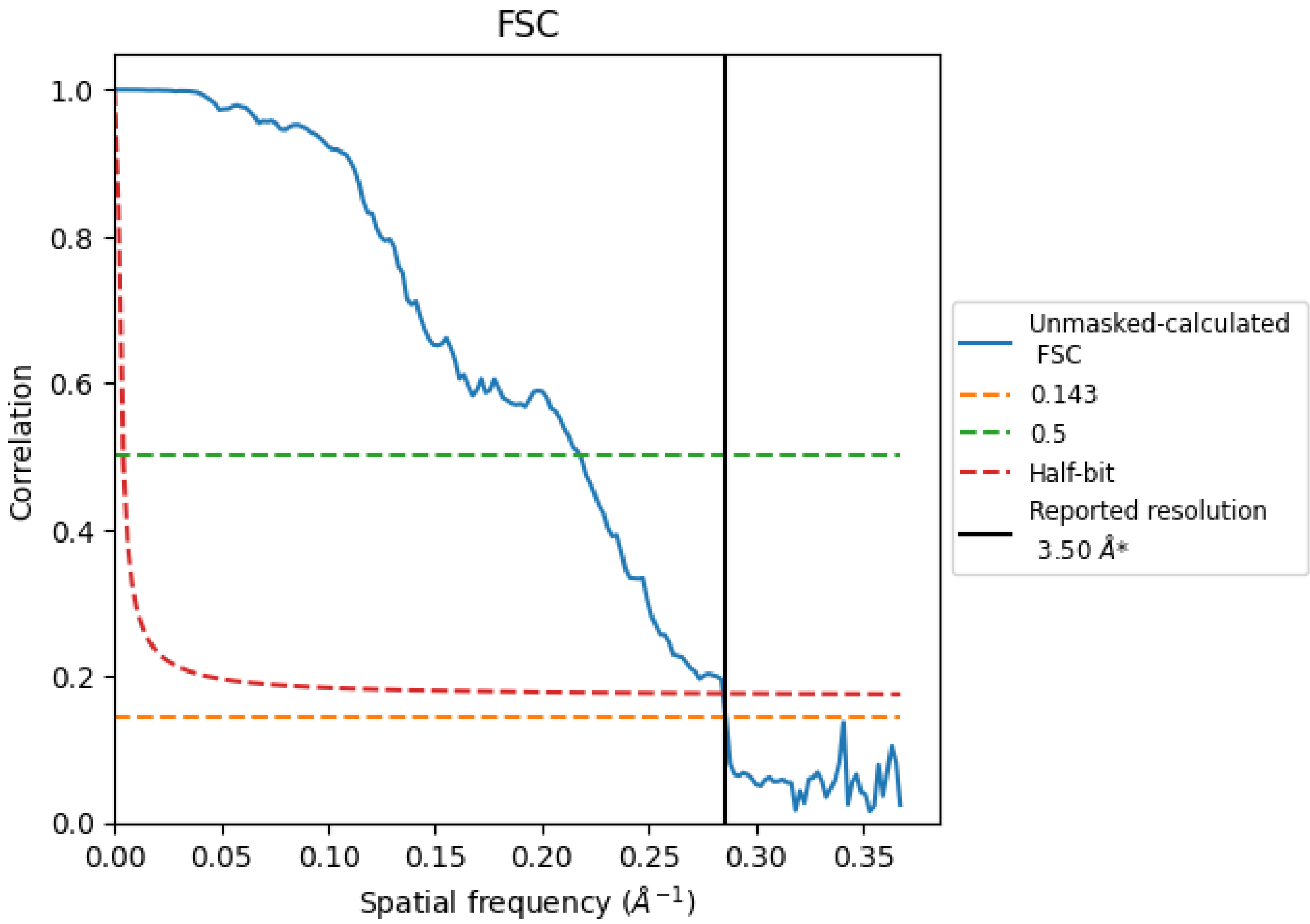

*Reported resolution corresponds to spatial frequency of 0.286 $Å^{-1}$

For Manuscript Review

8.2 Resolution estimates (i)

Resolution estimate (Å)	Estimation criterion (FSC cut-off)		
	0.143	0.5	Half-bit
Reported by author	3.50	-	-
Author-provided FSC curve	-	-	-
Unmasked-calculated*	3.50	4.59	3.51

*Resolution estimate based on FSC curve calculated by comparison of deposited half-maps.

For Manuscript Review

9 Map-model fit (i)

This section contains information regarding the fit between EMDB map EMD-36846 and PDB model 8K37. Per-residue inclusion information can be found in section 3 on page 6.

9.1 Map-model overlay (i)

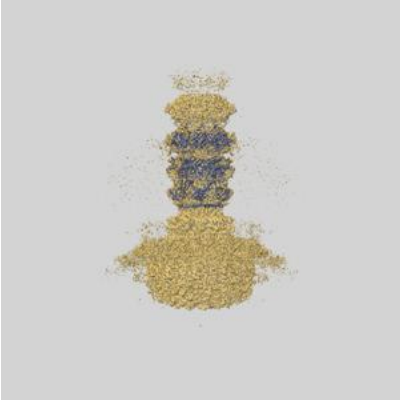

X

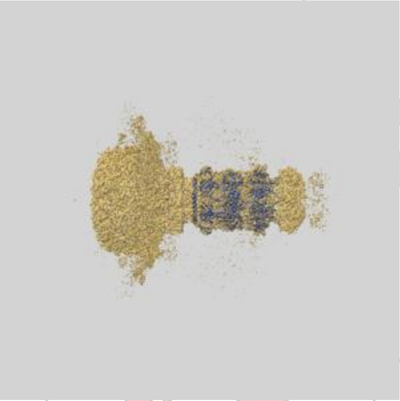

Y

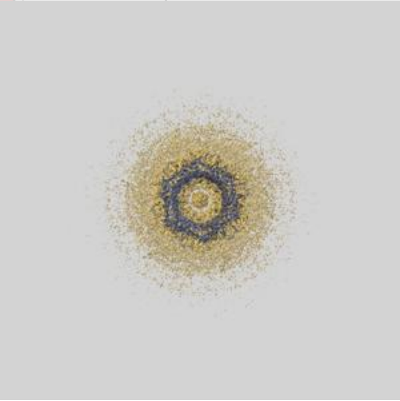

Z

The images above show the 3D surface view of the map at the recommended contour level 4.5 at 50% transparency in yellow overlaid with a ribbon representation of the model coloured in blue. These images allow for the visual assessment of the quality of fit between the atomic model and the map.

For Manuscript Review

9.2 Q-score mapped to coordinate model (i)

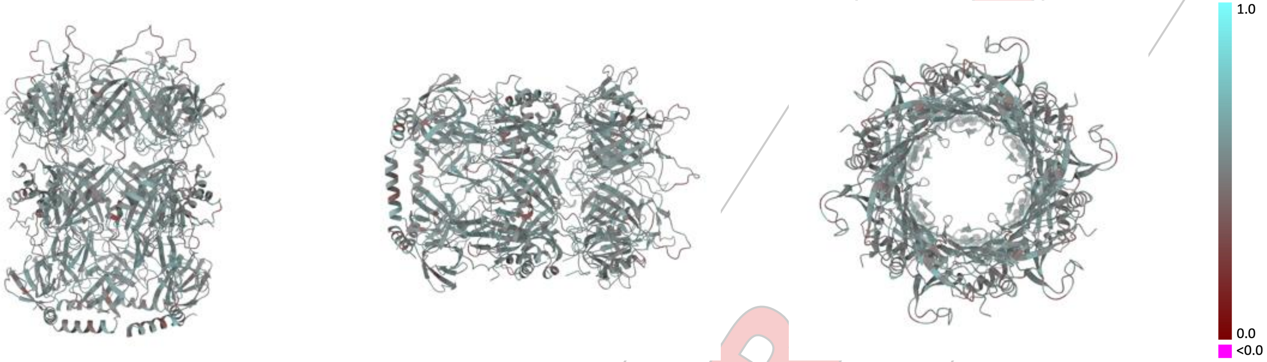

X Y Z

The images above show the model with each residue coloured according its Q-score. This shows their resolvability in the map with higher Q-score values reflecting better resolvability. Please note: Q-score is calculating the resolvability of atoms, and thus high values are only expected at resolutions at which atoms can be resolved. Low Q-score values may therefore be expected for many entries.

9.3 Atom inclusion mapped to coordinate model (i)

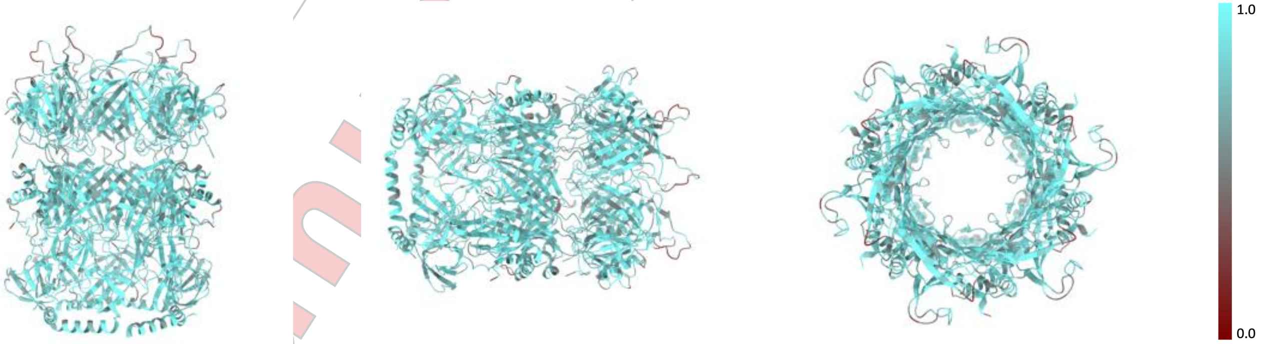

X Y Z

The images above show the model with each residue coloured according to its atom inclusion. This shows to what extent they are inside the map at the recommended contour level (4.5).

For Manuscript Review

9.4 Atom inclusion (i)

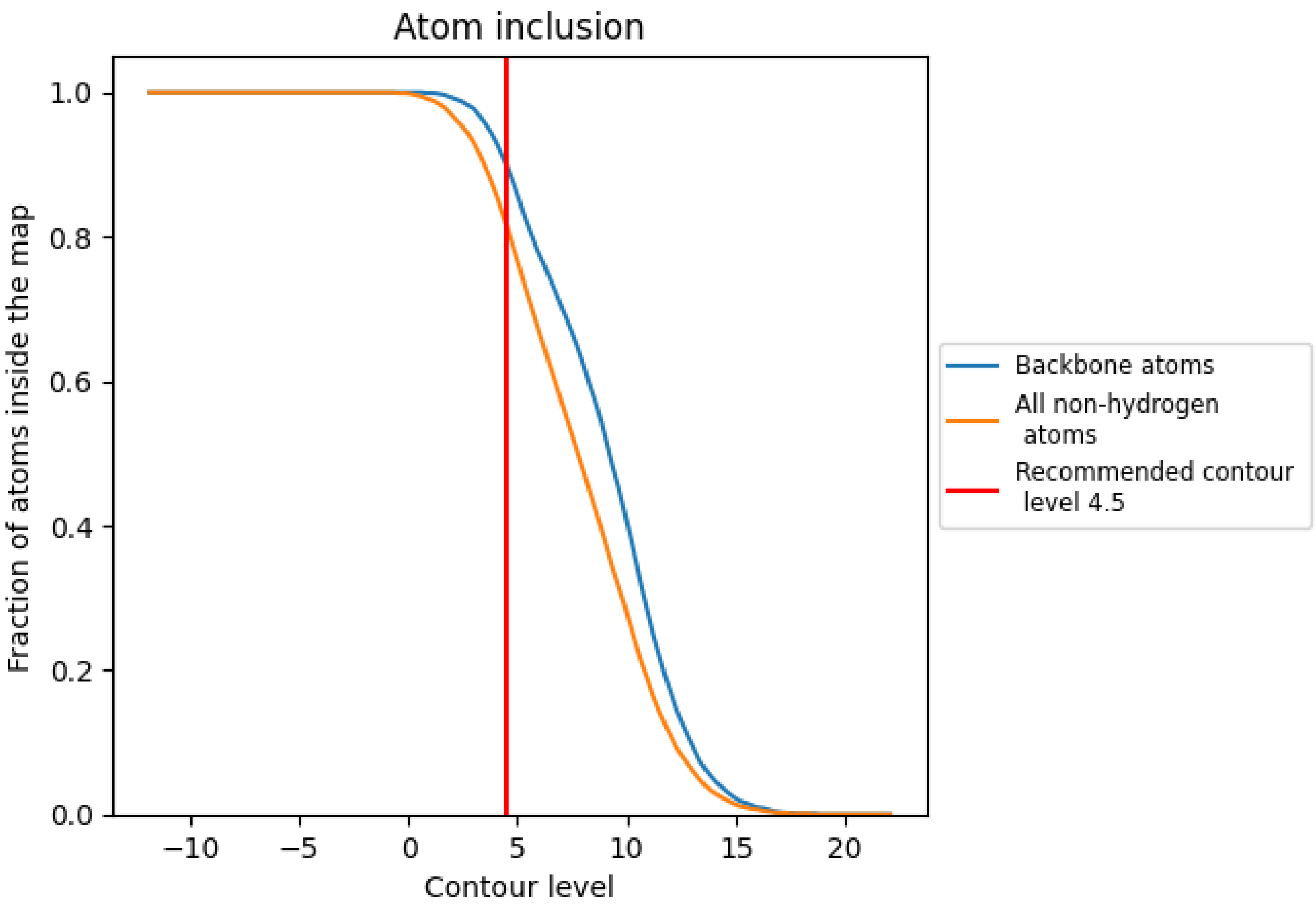

At the recommended contour level, 90% of all backbone atoms, 82% of all non-hydrogen atoms, are inside the map.

For Manuscript Review

9.5 Map-model fit summary (i)

The table lists the average atom inclusion at the recommended contour level (4.5) and Q-score for the entire model and for each chain.

Chain	Atom inclusion	Q-score
All	0.8160	0.5180
A	0.8260	0.5170
B	0.8320	0.5110
C	0.8240	0.5200
D	0.8220	0.5190
E	0.8340	0.5140
F	0.8230	0.5230
G	0.8000	0.5160
H	0.7970	0.5130
I	0.7990	0.5170
J	0.7940	0.5160
K	0.7950	0.5170
L	0.8020	0.5120
M	0.8260	0.5230
N	0.8300	0.5260
O	0.8260	0.5210
P	0.8250	0.5260
Q	0.8330	0.5250
R	0.8270	0.5230

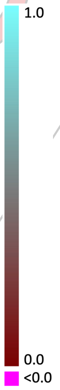

For Manuscript Review
