# Supplementary material for: Structure of the siphophage neck–Tail complex suggests that conserved tail tip proteins facilitate receptor binding and tail assembly
Source: PLoS Biol. 2023 Dec 14;21(12):e3002441. doi: 10.1371/journal.pbio.3002441 (PMC10721106; doi:10.1371/journal.pbio.3002441)
Supplement: S1 Appendix — (ZIP) [file pbio.3002441.s021.zip › PDB Validation Report.zip!/D_1300038909_report-portal-adaptor.pdf]

Full wwPDB EM Validation Report (i)

Jul 19, 2023 – 05:12 PM JST

PDB ID	:	8K38
EMDB ID	:	EMD-36847
Title	:	The structure of bacteriophage lambda portal-adaptor
Deposited on	:	2023-07-14
Resolution	:	3.20 Å(reported)

This wwPDB validation report is for manuscript review

This is a Full wwPDB EM Validation Report.

This report is produced by the wwPDB biocuration pipeline after annotation of the structure.

We welcome your comments at *validation@mail.wwpdb.org*
A user guide is available at
https://www.wwpdb.org/validation/2017/EMValidationReportHelp
with specific help available everywhere you see the (i) symbol.

The types of validation reports are described at
http://www.wwpdb.org/validation/2017/FAQs#types.

The following versions of software and data (see references (i)) were used in the production of this report:

EMDB validation analysis	:	0.0.1.dev50
MolProbity	:	4.02b-467
Percentile statistics	:	20191225.v01 (using entries in the PDB archive December 25th 2019)
MapQ	:	1.9.9
Ideal geometry (proteins)	:	Engh & Huber (2001)
Ideal geometry (DNA, RNA)	:	Parkinson et al. (1996)
Validation Pipeline (wwPDB-VP)	:	2.34

For Manuscript Review

1 Overall quality at a glance (i)

The following experimental techniques were used to determine the structure:
ELECTRON MICROSCOPY

The reported resolution of this entry is 3.20 Å.

Percentile scores (ranging between 0-100) for global validation metrics of the entry are shown in the following graphic. The table shows the number of entries on which the scores are based.

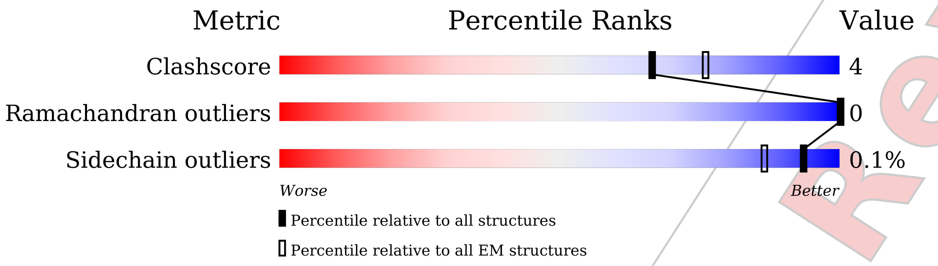

Metric	Whole archive (#Entries)	EM structures (#Entries)
Clashscore	158937	4297
Ramachandran outliers	154571	4023
Sidechain outliers	154315	3826

The table below summarises the geometric issues observed across the polymeric chains and their fit to the map. The red, orange, yellow and green segments of the bar indicate the fraction of residues that contain outliers for >=3, 2, 1 and 0 types of geometric quality criteria respectively. A grey segment represents the fraction of residues that are not modelled. The numeric value for each fraction is indicated below the corresponding segment, with a dot representing fractions <=5% The upper red bar (where present) indicates the fraction of residues that have poor fit to the EM map (all-atom inclusion < 40%). The numeric value is given above the bar.

Mol	Chain	Length	Quality of chain
1	A	533	78% 9% 12%
1	B	533	80% 8% 12%
1	C	533	79% 9% 12%
1	D	533	79% 8% 12%
1	E	533	79% 8% 12%
1	F	533	78% 10% 12%
1	G	533	78% 10% 12%
1	H	533	78% 10% 12%

Continued on next page...

For Manuscript Review

Continued from previous page...

Mol	Chain	Length	Quality of chain
1	I	533	78% 10% 12%
1	J	533	78% 10% 12%
1	K	533	80% 8% 12%
1	L	533	79% 8% 12%
2	M	68	• 90% 6% •
2	N	68	• 84% 12% •
2	O	68	• 71% 25% •
2	P	68	• 88% 7% •
2	Q	68	• 81% 15% •
2	R	68	• 68% 28% •
2	S	68	87% 9% •
2	T	68	• 78% 18% •
2	U	68	• 69% 26% •
2	V	68	87% 9% •
2	W	68	• 79% 16% •
2	X	68	• 71% 25% •

For Manuscript Review

2 Entry composition (i)

There are 2 unique types of molecules in this entry. The entry contains 50172 atoms, of which 0 are hydrogens and 0 are deuteriums.

In the tables below, the AltConf column contains the number of residues with at least one atom in alternate conformation and the Trace column contains the number of residues modelled with at most 2 atoms.

- Molecule 1 is a protein called Portal protein B.

Mol	Chain	Residues	Atoms					AltConf	Trace
1	A	468	Total 3675	C 2294	N 663	O 695	S 23	0	0
1	B	468	Total 3675	C 2294	N 663	O 695	S 23	0	0
1	C	468	Total 3675	C 2294	N 663	O 695	S 23	0	0
1	D	468	Total 3675	C 2294	N 663	O 695	S 23	0	0
1	E	468	Total 3675	C 2294	N 663	O 695	S 23	0	0
1	F	468	Total 3675	C 2294	N 663	O 695	S 23	0	0
1	G	468	Total 3675	C 2294	N 663	O 695	S 23	0	0
1	H	468	Total 3675	C 2294	N 663	O 695	S 23	0	0
1	I	468	Total 3675	C 2294	N 663	O 695	S 23	0	0
1	J	468	Total 3675	C 2294	N 663	O 695	S 23	0	0
1	K	468	Total 3675	C 2294	N 663	O 695	S 23	0	0
1	L	468	Total 3675	C 2294	N 663	O 695	S 23	0	0

- Molecule 2 is a protein called Head completion protein.

Mol	Chain	Residues	Atoms					AltConf	Trace
2	M	65	Total 506	C 313	N 96	O 95	S 2	0	0
2	N	65	Total 506	C 313	N 96	O 95	S 2	0	0
2	O	65	Total 506	C 313	N 96	O 95	S 2	0	0

Continued on next page...

For Manuscript Review

Continued from previous page...

Mol	Chain	Residues	Atoms					AltConf	Trace
2	P	65	Total 506	C 313	N 96	O 95	S 2	0	0
2	Q	65	Total 506	C 313	N 96	O 95	S 2	0	0
2	R	65	Total 506	C 313	N 96	O 95	S 2	0	0
2	S	65	Total 506	C 313	N 96	O 95	S 2	0	0
2	T	65	Total 506	C 313	N 96	O 95	S 2	0	0
2	U	65	Total 506	C 313	N 96	O 95	S 2	0	0
2	V	65	Total 506	C 313	N 96	O 95	S 2	0	0
2	W	65	Total 506	C 313	N 96	O 95	S 2	0	0
2	X	65	Total 506	C 313	N 96	O 95	S 2	0	0

For Manuscript Review

3 Residue-property plots (i)

These plots are drawn for all protein, RNA, DNA and oligosaccharide chains in the entry. The first graphic for a chain summarises the proportions of the various outlier classes displayed in the second graphic. The second graphic shows the sequence view annotated by issues in geometry and atom inclusion in map density. Residues are color-coded according to the number of geometric quality criteria for which they contain at least one outlier: green = 0, yellow = 1, orange = 2 and red = 3 or more. A red diamond above a residue indicates a poor fit to the EM map for this residue (all-atom inclusion < 40%). Stretches of 2 or more consecutive residues without any outlier are shown as a green connector. Residues present in the sample, but not in the model, are shown in grey.

- Molecule 1: Portal protein B

Chain A:

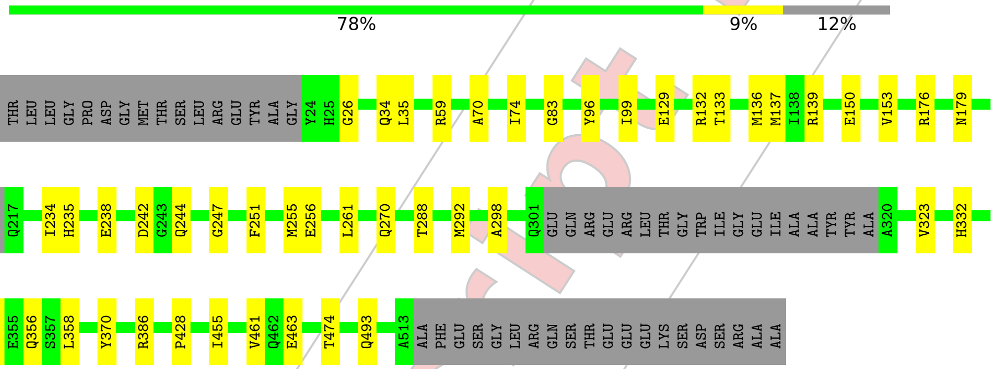

- Molecule 1: Portal protein B

Chain B:

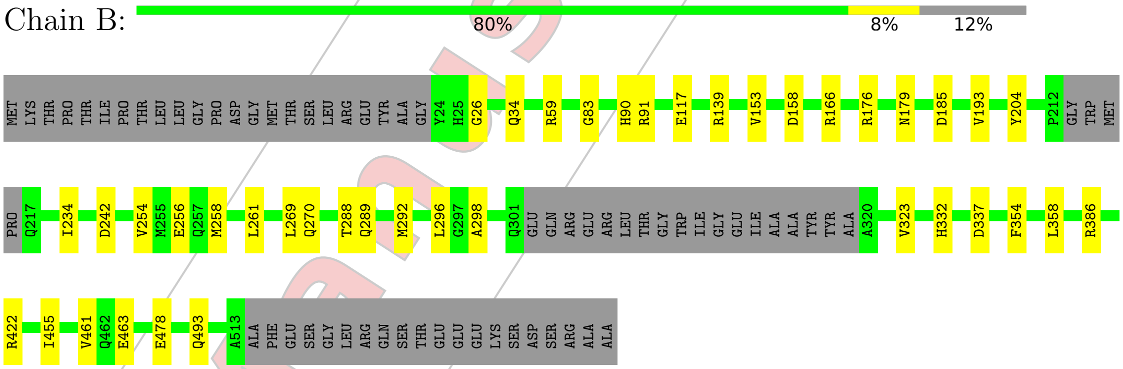

- Molecule 1: Portal protein B

Chain C:

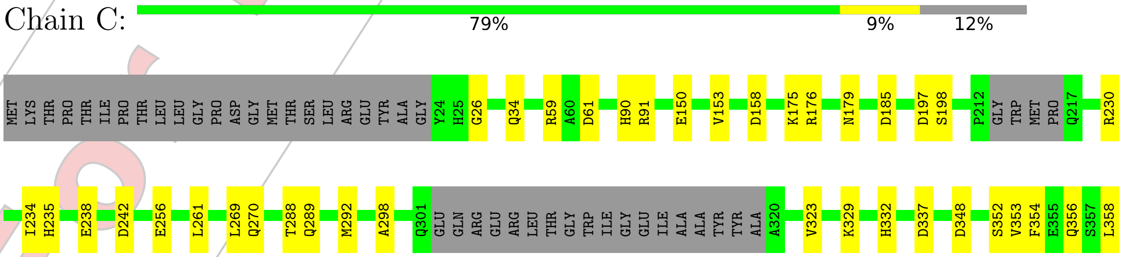

For Manuscript Review

A364 Y370 R386 E417 I455 V461 Q462 E463 T474 R501 A513 ALA PHE GLU SER GLY LEU ARG GLN SER THR GLU GLU GLU LYS SER ASP SER ARG ALA ALA

- Molecule 1: Portal protein B

Chain D: 79% 8% 12%

MET LYS THR PRO THR ILE PRO THR LEU LEU GLY PRO ASP GLY MET THR SER LEU ARG GLU TYR ALA GLY Y24 H25 G26 Q34 L35 R59 A70 I74 G83 H90 R91 T133 M136 R139 R176 N179 D185 P212 GLY TRP MET PRO Q217

E238 D242 G243 Q244 G247 F251 M255 E256 L261 Q270 L286 D287 T288 M292 A298 Q301 GLU GLN ARG GLU ARG LEU THR GLY TRP ILE GLY GLU ILE ALA ALA TYR TYR ALA A320 V323 H332 D337 D346 T347 D348 N349 F354 E355 Q356

S357 L358 Y370 R375 R386 I455 V461 Q462 E463 T474 Q493 A513 ALA PHE GLU SER GLY LEU ARG GLN SER THR GLU GLU GLU LYS SER ASP SER ARG ALA ALA

- Molecule 1: Portal protein B

Chain E: 79% 8% 12%

MET LYS THR PRO THR ILE PRO THR LEU LEU GLY PRO ASP GLY MET THR SER LEU ARG GLU TYR ALA GLY Y24 H25 G26 Q34 R59 G83 L88 S89 H90 R91 R139 F152 V153 R166 R176 N179 D185 V193 Y204 P212 GLY TRP MET PRO

Q217 F233 I234 D242 V254 M255 E256 Q257 M258 L261 L269 Q270 T288 Q289 M292 L296 G297 A298 Q301 GLU GLN ARG GLU ARG LEU THR GLY TRP ILE GLY GLU ILE ALA ALA TYR TYR ALA A320 V323 K329 H332 D337 D348 S352 V353

F354 L358 R386 R422 W447 I455 V461 Q462 E463 Q493 A513 ALA PHE GLU SER GLY LEU ARG GLN SER THR GLU GLU GLU LYS SER ASP SER ARG ALA ALA

- Molecule 1: Portal protein B

Chain F: 78% 10% 12%

MET LYS THR PRO THR ILE PRO THR LEU LEU GLY PRO ASP GLY MET THR SER LEU ARG GLU TYR ALA GLY Y24 H25 G26 Q34 R59 A60 D61 H90 R91 F107 V111 E150 V153 D158 R166 K175 R176 N179 D185 D197 S198 P212 GLY

TRP MET PRO Q217 R230 I234 H235 E238 E256 L261 L269 Q270 T288 Q289 M292 A298 Q301 GLU GLN ARG GLU ARG LEU THR GLY TRP ILE GLY GLU ILE ALA ALA TYR TYR ALA A320 V323 K329 H332 D337 D346 N349 V353

F354 E355 Q356 S357 L358 A364 Y370 R386 E417 R422 I455 V461 Q462 E463 T474 V494 T497 R501 A513 ALA PHE GLU SER GLY LEU ARG GLN SER THR GLU GLU GLU LYS SER ASP SER ARG ALA ALA

- Molecule 1: Portal protein B

Chain G: 78% 10% 12%

MET LYS THR PRO THR ILE PRO THR LEU LEU GLY PRO ASP GLY MET THR SER LEU ARG GLU TYR ALA GLY Y24 H25 G26 Q34 L35 R59 A70 I74 G83 H90 R91 E117 T133 M136 R139 V153 D158 R176 N179 D185 S207

For Manuscript Review

P212 GLY TRP MET PRO Q217 I234 E238 Q244 G247 F251 M255 E256 L261 Q270 L286 D287 T288 M292 A298 Q301 GLU GLN ARG GLU ARG LEU THR GLY TRP ILE GLY GLU ILE ALA ALA TYR TYR ALA A320 V323 K329 H332

D337 D346 T347 D348 N349 F354 E355 Q356 S357 L358 Y370 R375 R386 S434 F435 I455 V461 Q462 E463 T474 Q493 R501 A513 ALA PHE GLU SER GLY LEU LEU ARG GLN SER THR GLU GLU GLU LYS SER ASP SER ARG ALA ALA

- Molecule 1: Portal protein B

Chain H: 78% 10% 12%

MET LYS THR PRO THR ILE PRO THR LEU LEU GLY PRO ASP GLY MET THR SER LEU ARG GLU TYR ALA GLY Y24 H25 G26 Q34 R59 G83 L88 S89 H90 R91 E110 R139 F152 V153 D158 R166 N179 D185 V193 Y204 P212 GLY TRP

MET PRO Q217 F233 I234 D242 V254 M255 E256 Q257 M258 L261 L269 Q270 T288 Q289 M292 L296 G297 A298 Q301 GLU GLN ARG GLU ARG LEU THR GLY TRP ILE GLY GLU ILE ALA ALA TYR TYR ALA A320 V323 K329 H332 D337 F354

L358 R386 E418 A419 I420 V421 R422 V425 T426 L427 R432 F433 W447 I455 V461 Q462 E463 E478 Q493 A513 ALA PHE GLU SER GLY LEU ARG GLN SER THR GLU GLU GLU LYS SER ASP SER ARG ALA ALA

- Molecule 1: Portal protein B

Chain I: 78% 10% 12%

MET LYS THR PRO THR ILE PRO THR LEU LEU GLY PRO ASP GLY MET THR SER LEU ARG GLU TYR ALA GLY Y24 H25 G26 Q34 R59 A60 D61 D62 N66 H90 R91 F107 V111 E150 V153 D158 K175 R176 N179 D185 G199 P212 GLY

TRP MET PRO Q217 R230 I234 H235 E238 D242 E256 L261 L269 Q270 I273 T288 Q289 M292 A298 Q301 GLU GLN ARG GLU ARG LEU THR GLY TRP ILE GLY GLU ILE ALA ALA TYR TYR ALA A320 V323 K329 H332 D337 D346

T347 D348 N349 F354 E355 Q356 S357 L358 Y370 R386 K401 E417 I455 V461 Q462 E463 T474 R501 A513 ALA PHE GLU SER GLY LEU ARG GLN SER THR GLU GLU GLU LYS SER ASP SER ARG ALA ALA

- Molecule 1: Portal protein B

Chain J: 78% 10% 12%

MET LYS THR PRO THR ILE PRO THR LEU LEU GLY PRO ASP GLY MET THR SER LEU ARG GLU TYR ALA GLY Y24 H25 G26 Q34 L35 R59 A70 I74 G83 H90 R91 A106 E110 E129 T133 M136 R139 V153 R176 N179 D185

P212 GLY TRP MET PRO Q217 I234 E238 D242 G243 Q244 G247 F251 M255 E256 L261 L269 Q270 L286 D287 T288 M292 A298 Q301 GLU GLN ARG GLU ARG LEU THR GLY TRP ILE GLY GLU ILE ALA ALA TYR TYR ALA A320 V323 R324 L325

K329 H332 D337 D346 N349 F354 E355 Q356 S357 L358 Y370 R386 I455 V461 Q462 E463 T474 Q493 A513 ALA PHE GLU SER GLY LEU ARG GLN SER THR GLU GLU GLU LYS SER ASP SER ARG ALA ALA

- Molecule 1: Portal protein B

Chain K: 80% 8% 12%

For Manuscript Review

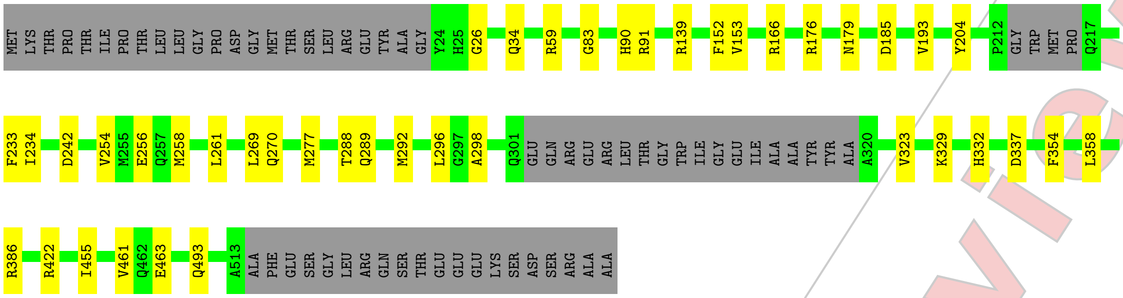

• Molecule 1: Portal protein B

Chain L:

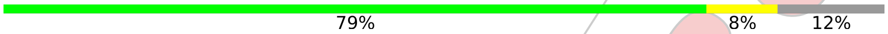

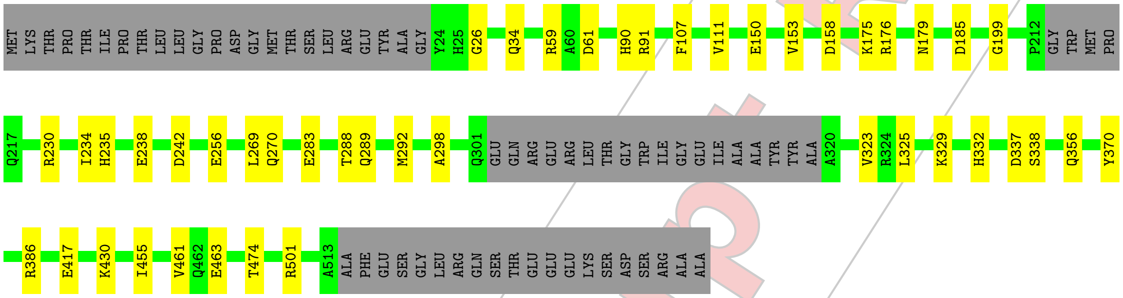

• Molecule 2: Head completion protein

Chain M:

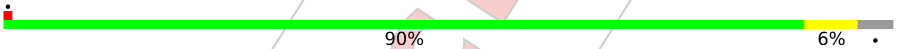

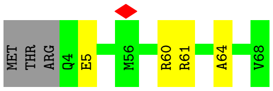

• Molecule 2: Head completion protein

Chain N:

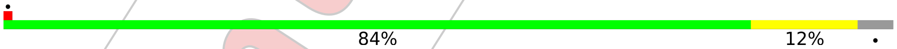

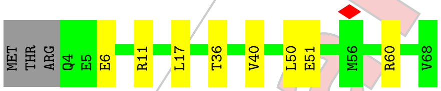

• Molecule 2: Head completion protein

Chain O:

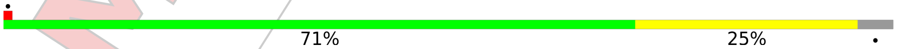

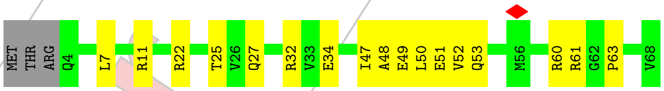

• Molecule 2: Head completion protein

Chain P:

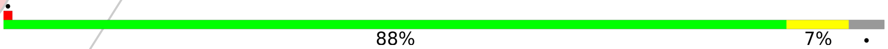

For Manuscript Review

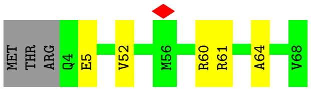

• Molecule 2: Head completion protein

Chain Q:

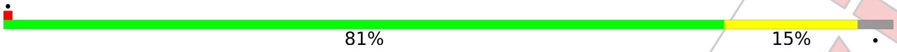

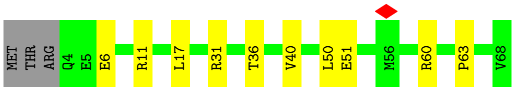

• Molecule 2: Head completion protein

Chain R:

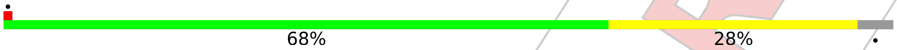

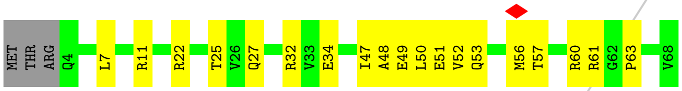

• Molecule 2: Head completion protein

Chain S:

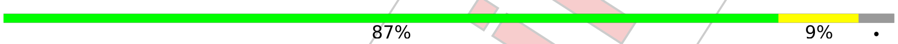

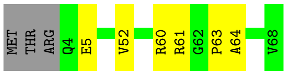

• Molecule 2: Head completion protein

Chain T:

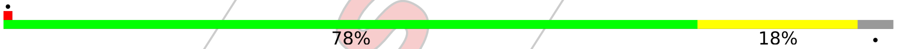

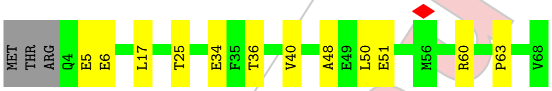

• Molecule 2: Head completion protein

Chain U:

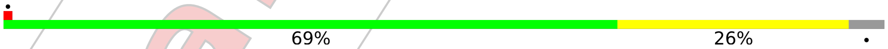

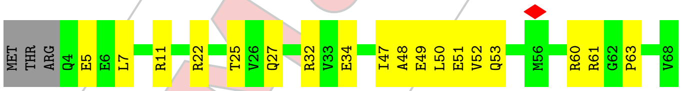

• Molecule 2: Head completion protein

Chain V:

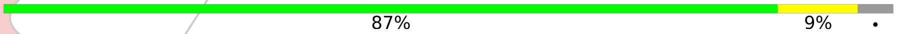

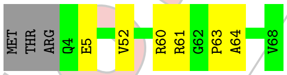

• Molecule 2: Head completion protein

For Manuscript Review

Chain W:

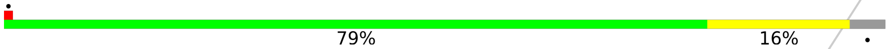

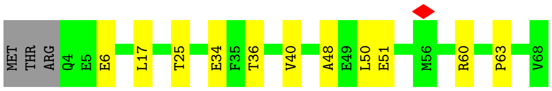

• Molecule 2: Head completion protein

Chain X:

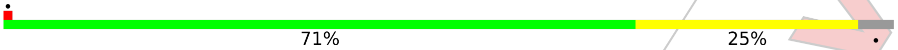

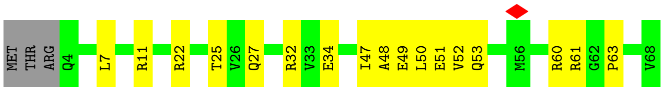

For Manuscript Review

4 Experimental information (i)

Property	Value	Source
EM reconstruction method	SINGLE PARTICLE	Depositor
Imposed symmetry	POINT, Not provided	
Number of particles used	72868	Depositor
Resolution determination method	FSC 0.143 CUT-OFF	Depositor
CTF correction method	NONE	Depositor
Microscope	FEI TITAN KRIOS	Depositor
Voltage (kV)	300	Depositor
Electron dose $(e^-/\text{Å}^2)$	32	Depositor
Minimum defocus (nm)	1600	Depositor
Maximum defocus (nm)	2200	Depositor
Magnification	Not provided	
Image detector	GATAN K3 (6k x 4k)	Depositor
Maximum map value	39.886	Depositor
Minimum map value	-21.829	Depositor
Average map value	0.003	Depositor
Map value standard deviation	1.466	Depositor
Recommended contour level	5.5	Depositor
Map size (Å)	544.0, 544.0, 544.0	wwPDB
Map dimensions	400, 400, 400	wwPDB
Map angles (°)	90.0, 90.0, 90.0	wwPDB
Pixel spacing (Å)	1.36, 1.36, 1.36	Depositor

For Manuscript Review

5 Model quality (i)

5.1 Standard geometry (i)

The Z score for a bond length (or angle) is the number of standard deviations the observed value is removed from the expected value. A bond length (or angle) with $|Z| > 5$ is considered an outlier worth inspection. RMSZ is the root-mean-square of all Z scores of the bond lengths (or angles).

Mol	Chain	Bond lengths		Bond angles	
		RMSZ	#|Z| >5	RMSZ	#|Z| >5
1	A	0.28	0/3755	0.48	0/5072
1	B	0.28	0/3755	0.49	0/5072
1	C	0.27	0/3755	0.48	0/5072
1	D	0.26	0/3755	0.48	0/5072
1	E	0.27	0/3755	0.49	0/5072
1	F	0.26	0/3755	0.49	0/5072
1	G	0.26	0/3755	0.48	0/5072
1	H	0.29	0/3755	0.49	0/5072
1	I	0.26	0/3755	0.48	0/5072
1	J	0.26	0/3755	0.48	0/5072
1	K	0.26	0/3755	0.49	0/5072
1	L	0.26	0/3755	0.48	0/5072
2	M	0.32	0/511	0.56	0/685
2	N	0.32	0/511	0.55	0/685
2	O	0.31	0/511	0.58	0/685
2	P	0.32	0/511	0.56	0/685
2	Q	0.32	0/511	0.56	0/685
2	R	0.31	0/511	0.58	0/685
2	S	0.32	0/511	0.55	0/685
2	T	0.31	0/511	0.56	0/685
2	U	0.31	0/511	0.57	0/685
2	V	0.32	0/511	0.55	0/685
2	W	0.32	0/511	0.56	0/685
2	X	0.31	0/511	0.57	0/685
All	All	0.27	0/51192	0.49	0/69084

There are no bond length outliers.

There are no bond angle outliers.

There are no chirality outliers.

There are no planarity outliers.

For Manuscript Review

5.2 Too-close contacts (i)

In the following table, the Non-H and H(model) columns list the number of non-hydrogen atoms and hydrogen atoms in the chain respectively. The H(added) column lists the number of hydrogen atoms added and optimized by MolProbity. The Clashes column lists the number of clashes within the asymmetric unit, whereas Symm-Clashes lists symmetry-related clashes.

Mol	Chain	Non-H	H(model)	H(added)	Clashes	Symm-Clashes
1	A	3675	0	3524	36	0
1	B	3675	0	3524	35	0
1	C	3675	0	3524	38	0
1	D	3675	0	3524	37	0
1	E	3675	0	3524	36	0
1	F	3675	0	3524	39	0
1	G	3675	0	3524	40	0
1	H	3675	0	3524	40	0
1	I	3675	0	3524	40	0
1	J	3675	0	3524	39	0
1	K	3675	0	3524	34	0
1	L	3675	0	3524	36	0
2	M	506	0	516	5	0
2	N	506	0	516	6	0
2	O	506	0	516	14	0
2	P	506	0	516	6	0
2	Q	506	0	516	8	0
2	R	506	0	516	14	0
2	S	506	0	516	7	0
2	T	506	0	516	8	0
2	U	506	0	516	13	0
2	V	506	0	516	7	0
2	W	506	0	516	8	0
2	X	506	0	516	14	0
All	All	50172	0	48480	395	0

The all-atom clashscore is defined as the number of clashes found per 1000 atoms (including hydrogen atoms). The all-atom clashscore for this structure is 4.

All (395) close contacts within the same asymmetric unit are listed below, sorted by their clash magnitude.

Atom-1	Atom-2	Interatomic distance (Å)	Clash overlap (Å)
1:E:463:GLU:HG3	1:F:461:VAL:HG21	1.71	0.72
1:G:34:GLN:NE2	1:H:256:GLU:OE2	2.22	0.72
1:A:34:GLN:NE2	1:B:256:GLU:OE2	2.21	0.72

Continued on next page...

For Manuscript Review

Continued from previous page...

Atom-1	Atom-2	Interatomic distance (Å)	Clash overlap (Å)
1:H:463:GLU:HG3	1:I:461:VAL:HG21	1.72	0.72
1:J:34:GLN:NE2	1:K:256:GLU:OE2	2.22	0.72
2:T:25:THR:HG22	2:T:34:GLU:HG3	1.70	0.72
1:D:34:GLN:NE2	1:E:256:GLU:OE2	2.22	0.72
1:B:463:GLU:HG3	1:C:461:VAL:HG21	1.73	0.71
1:K:34:GLN:NE2	1:L:256:GLU:OE2	2.23	0.70
1:K:463:GLU:HG3	1:L:461:VAL:HG21	1.72	0.70
1:B:34:GLN:NE2	1:C:256:GLU:OE2	2.23	0.70
1:E:34:GLN:NE2	1:F:256:GLU:OE2	2.23	0.70
2:W:25:THR:HG22	2:W:34:GLU:HG3	1.74	0.70
1:H:34:GLN:NE2	1:I:256:GLU:OE2	2.23	0.69
1:G:34:GLN:OE1	1:I:270:GLN:NE2	2.28	0.66
1:B:90:HIS:O	1:B:91:ARG:NH1	2.30	0.65
1:A:463:GLU:HG3	1:B:461:VAL:HG21	1.77	0.65
1:E:166:ARG:HE	1:E:422:ARG:HH21	1.45	0.65
1:F:34:GLN:OE1	1:H:270:GLN:NE2	2.29	0.65
1:B:166:ARG:HE	1:B:422:ARG:HH21	1.45	0.65
1:E:90:HIS:O	1:E:91:ARG:NH1	2.30	0.65
1:A:270:GLN:NE2	1:K:34:GLN:OE1	2.30	0.65
1:F:463:GLU:HG3	1:G:461:VAL:HG21	1.79	0.65
1:H:90:HIS:O	1:H:91:ARG:NH1	2.30	0.65
1:K:90:HIS:O	1:K:91:ARG:NH1	2.30	0.65
1:A:461:VAL:HG21	1:L:463:GLU:HG3	1.78	0.65
1:H:34:GLN:OE1	1:J:270:GLN:NE2	2.30	0.64
1:I:242:ASP:OD2	1:J:176:ARG:NH1	2.31	0.64
1:K:166:ARG:HE	1:K:422:ARG:HH21	1.45	0.64
1:A:34:GLN:OE1	1:C:270:GLN:NE2	2.27	0.64
1:J:463:GLU:HG3	1:K:461:VAL:HG21	1.79	0.64
1:D:34:GLN:OE1	1:F:270:GLN:NE2	2.28	0.64
1:A:176:ARG:NH1	1:L:242:ASP:OD2	2.31	0.64
2:U:11:ARG:NH2	2:U:51:GLU:OE2	2.29	0.63
2:R:11:ARG:NH2	2:R:51:GLU:OE2	2.30	0.63
1:C:34:GLN:OE1	1:E:270:GLN:NE2	2.29	0.63
1:C:242:ASP:OD2	1:D:176:ARG:NH1	2.31	0.63
1:H:166:ARG:HE	1:H:422:ARG:HH21	1.45	0.63
1:E:34:GLN:OE1	1:G:270:GLN:NE2	2.29	0.63
1:C:463:GLU:HG3	1:D:461:VAL:HG21	1.80	0.63
2:O:11:ARG:NH2	2:O:51:GLU:OE2	2.29	0.63
1:B:34:GLN:OE1	1:D:270:GLN:NE2	2.29	0.63
1:B:292:MET:HG2	1:B:296:LEU:HD12	1.80	0.63
1:I:463:GLU:HG3	1:J:461:VAL:HG21	1.81	0.62

Continued on next page...

For Manuscript Review

Continued from previous page...

Atom-1	Atom-2	Interatomic distance (Å)	Clash overlap (Å)
1:F:90:HIS:O	1:F:91:ARG:NH1	2.33	0.61
1:E:179:ASN:ND2	1:E:185:ASP:OD1	2.34	0.61
1:I:90:HIS:O	1:I:91:ARG:NH1	2.33	0.61
1:B:270:GLN:NE2	1:L:34:GLN:OE1	2.29	0.61
1:B:179:ASN:ND2	1:B:185:ASP:OD1	2.34	0.61
1:J:34:GLN:OE1	1:L:270:GLN:NE2	2.28	0.61
1:H:179:ASN:ND2	1:H:185:ASP:OD1	2.34	0.60
1:I:34:GLN:OE1	1:K:270:GLN:NE2	2.29	0.60
2:X:11:ARG:NH2	2:X:51:GLU:OE2	2.29	0.60
1:D:179:ASN:ND2	1:D:185:ASP:OD1	2.32	0.60
1:C:90:HIS:O	1:C:91:ARG:NH1	2.34	0.60
1:K:292:MET:HG2	1:K:296:LEU:HD12	1.83	0.60
1:E:292:MET:HG2	1:E:296:LEU:HD12	1.84	0.60
1:H:292:MET:HG2	1:H:296:LEU:HD12	1.84	0.60
1:G:26:GLY:O	1:H:59:ARG:NH1	2.35	0.60
1:C:197:ASP:OD1	1:C:198:SER:N	2.35	0.60
1:D:26:GLY:O	1:E:59:ARG:NH1	2.34	0.59
2:X:25:THR:HG22	2:X:34:GLU:HG3	1.84	0.59
1:K:179:ASN:ND2	1:K:185:ASP:OD1	2.34	0.59
1:I:332:HIS:O	2:U:60:ARG:NH2	2.36	0.59
1:F:332:HIS:O	2:R:60:ARG:NH2	2.36	0.58
1:E:261:LEU:HD13	1:E:358:LEU:HD12	1.85	0.58
1:K:261:LEU:HD13	1:K:358:LEU:HD12	1.85	0.58
1:F:197:ASP:OD1	1:F:198:SER:N	2.37	0.58
1:C:26:GLY:O	1:D:59:ARG:NH1	2.37	0.57
1:J:133:THR:H	1:J:136:MET:HE3	1.70	0.57
1:L:332:HIS:O	2:X:60:ARG:NH2	2.37	0.57
1:B:261:LEU:HD13	1:B:358:LEU:HD12	1.85	0.57
1:J:26:GLY:O	1:K:59:ARG:NH1	2.35	0.57
1:H:261:LEU:HD13	1:H:358:LEU:HD12	1.85	0.57
1:A:26:GLY:O	1:B:59:ARG:NH1	2.36	0.57
1:D:463:GLU:HG3	1:E:461:VAL:HG21	1.87	0.57
1:L:230:ARG:HH21	1:L:417:GLU:HG2	1.70	0.57
2:X:7:LEU:HD13	2:X:50:LEU:HD22	1.87	0.57
2:U:7:LEU:HD13	2:U:50:LEU:HD22	1.87	0.57
1:F:26:GLY:O	1:G:59:ARG:NH1	2.37	0.57
2:R:7:LEU:HD13	2:R:50:LEU:HD22	1.87	0.57
1:I:26:GLY:O	1:J:59:ARG:NH1	2.38	0.56
1:A:59:ARG:NH1	1:L:26:GLY:O	2.38	0.56
1:C:332:HIS:O	2:O:60:ARG:NH2	2.38	0.56
2:O:7:LEU:HD13	2:O:50:LEU:HD22	1.87	0.56

Continued on next page...

For Manuscript Review

Continued from previous page...

Atom-1	Atom-2	Interatomic distance (Å)	Clash overlap (Å)
1:I:261:LEU:HD13	1:I:358:LEU:HD12	1.88	0.56
1:F:34:GLN:NE2	1:G:256:GLU:OE2	2.39	0.56
1:H:153:VAL:HB	1:H:234:ILE:HB	1.88	0.56
1:J:251:PHE:O	1:J:255:MET:HG3	2.06	0.56
2:U:47:ILE:O	2:U:51:GLU:HG3	2.05	0.56
1:E:153:VAL:HB	1:E:234:ILE:HB	1.88	0.56
2:R:47:ILE:O	2:R:51:GLU:HG3	2.06	0.56
2:R:48:ALA:O	2:R:52:VAL:HG23	2.06	0.56
1:B:153:VAL:HB	1:B:234:ILE:HB	1.88	0.56
1:D:133:THR:H	1:D:136:MET:HE3	1.70	0.56
2:O:47:ILE:O	2:O:51:GLU:HG3	2.05	0.56
2:Q:6:GLU:HB2	2:Q:50:LEU:HD21	1.88	0.56
2:X:48:ALA:O	2:X:52:VAL:HG23	2.06	0.56
2:N:6:GLU:HB2	2:N:50:LEU:HD21	1.88	0.55
1:A:251:PHE:O	1:A:255:MET:HG3	2.06	0.55
1:B:26:GLY:O	1:C:59:ARG:NH1	2.39	0.55
1:H:26:GLY:O	1:I:59:ARG:NH1	2.39	0.55
2:U:48:ALA:O	2:U:52:VAL:HG23	2.06	0.55
1:G:133:THR:H	1:G:136:MET:HE3	1.70	0.55
1:D:251:PHE:O	1:D:255:MET:HG3	2.06	0.55
1:C:230:ARG:HH21	1:C:417:GLU:HG2	1.72	0.55
1:I:230:ARG:HH21	1:I:417:GLU:HG2	1.72	0.55
1:K:386:ARG:HG2	1:L:455:ILE:O	2.06	0.55
1:K:153:VAL:HB	1:K:234:ILE:HB	1.89	0.55
2:O:48:ALA:O	2:O:52:VAL:HG23	2.06	0.55
2:X:47:ILE:O	2:X:51:GLU:HG3	2.05	0.55
1:F:179:ASN:ND2	1:F:185:ASP:OD1	2.39	0.55
2:X:49:GLU:OE2	2:X:53:GLN:NE2	2.37	0.55
1:E:386:ARG:HG2	1:F:455:ILE:O	2.07	0.55
1:G:176:ARG:NH1	1:G:207:SER:O	2.40	0.55
1:H:332:HIS:O	2:T:60:ARG:NH2	2.40	0.55
1:H:386:ARG:HG2	1:I:455:ILE:O	2.07	0.55
1:K:26:GLY:O	1:L:59:ARG:NH1	2.39	0.55
1:A:261:LEU:HD13	1:A:358:LEU:HD12	1.89	0.54
1:J:261:LEU:HD13	1:J:358:LEU:HD12	1.89	0.54
1:B:332:HIS:O	2:N:60:ARG:NH2	2.40	0.54
1:K:337:ASP:OD2	2:W:60:ARG:NH1	2.35	0.54
1:G:261:LEU:HD13	1:G:358:LEU:HD12	1.89	0.54
1:G:463:GLU:HG3	1:H:461:VAL:HG21	1.89	0.54
1:L:150:GLU:OE1	1:L:235:HIS:NE2	2.39	0.54
1:C:150:GLU:OE1	1:C:235:HIS:NE2	2.39	0.54

Continued on next page...

For Manuscript Review

Continued from previous page...

Atom-1	Atom-2	Interatomic distance (Å)	Clash overlap (Å)
1:C:34:GLN:NE2	1:D:256:GLU:OE2	2.39	0.54
1:C:179:ASN:ND2	1:C:185:ASP:OD1	2.40	0.54
1:D:286:LEU:HD22	2:P:52:VAL:HG11	1.90	0.54
1:E:26:GLY:O	1:F:59:ARG:NH1	2.39	0.54
1:I:179:ASN:ND2	1:I:185:ASP:OD1	2.40	0.54
1:L:179:ASN:ND2	1:L:185:ASP:OD1	2.40	0.54
1:B:337:ASP:OD2	2:N:60:ARG:NH1	2.35	0.54
1:E:332:HIS:O	2:Q:60:ARG:NH2	2.41	0.54
1:E:337:ASP:OD2	2:Q:60:ARG:NH1	2.36	0.54
1:F:230:ARG:HH21	1:F:417:GLU:HG2	1.72	0.54
1:K:332:HIS:O	2:W:60:ARG:NH2	2.40	0.54
1:C:261:LEU:HD13	1:C:358:LEU:HD12	1.90	0.54
1:D:261:LEU:HD13	1:D:358:LEU:HD12	1.89	0.54
1:K:166:ARG:NE	1:K:422:ARG:HH21	2.06	0.54
1:K:298:ALA:HB2	1:K:323:VAL:HG22	1.90	0.54
1:G:286:LEU:HD22	2:S:52:VAL:HG11	1.90	0.53
1:B:242:ASP:OD2	1:C:176:ARG:NH1	2.41	0.53
1:F:261:LEU:HD13	1:F:358:LEU:HD12	1.90	0.53
1:B:298:ALA:HB2	1:B:323:VAL:HG22	1.91	0.53
1:E:242:ASP:OD2	1:F:176:ARG:NH1	2.40	0.53
1:I:153:VAL:HB	1:I:234:ILE:HB	1.91	0.53
2:T:6:GLU:HB2	2:T:50:LEU:HD21	1.91	0.53
1:F:150:GLU:OE1	1:F:235:HIS:NE2	2.40	0.53
1:H:298:ALA:HB2	1:H:323:VAL:HG22	1.90	0.53
1:F:153:VAL:HB	1:F:234:ILE:HB	1.91	0.53
1:I:34:GLN:NE2	1:J:256:GLU:OE2	2.39	0.53
2:W:6:GLU:HB2	2:W:50:LEU:HD21	1.91	0.52
1:B:386:ARG:HG2	1:C:455:ILE:O	2.09	0.52
1:E:298:ALA:HB2	1:E:323:VAL:HG22	1.90	0.52
1:H:166:ARG:NE	1:H:422:ARG:HH21	2.07	0.52
1:K:242:ASP:OD2	1:L:176:ARG:NH1	2.41	0.52
1:B:166:ARG:NE	1:B:422:ARG:HH21	2.07	0.52
1:C:354:PHE:HB2	1:D:348:ASP:HB2	1.90	0.52
2:O:49:GLU:OE2	2:O:53:GLN:NE2	2.37	0.52
2:R:49:GLU:OE2	2:R:53:GLN:NE2	2.38	0.52
1:I:150:GLU:OE1	1:I:235:HIS:NE2	2.39	0.52
1:L:90:HIS:O	1:L:91:ARG:NH1	2.43	0.52
1:J:286:LEU:HD22	2:V:52:VAL:HG11	1.91	0.52
1:A:256:GLU:OE2	1:L:34:GLN:NE2	2.39	0.51
1:E:166:ARG:NE	1:E:422:ARG:HH21	2.07	0.51
1:D:337:ASP:OD2	2:P:60:ARG:NH1	2.32	0.51

Continued on next page...

For Manuscript Review

Continued from previous page...

Atom-1	Atom-2	Interatomic distance (Å)	Clash overlap (Å)
2:U:25:THR:HG22	2:U:34:GLU:HG3	1.91	0.51
1:G:90:HIS:O	1:G:91:ARG:NH1	2.44	0.51
2:R:25:THR:HG22	2:R:34:GLU:HG3	1.93	0.51
1:I:107:PHE:O	1:I:111:VAL:HG12	2.11	0.51
1:H:337:ASP:OD2	2:T:60:ARG:NH1	2.35	0.51
1:J:332:HIS:O	2:V:60:ARG:NH2	2.44	0.51
1:D:298:ALA:HB2	1:D:323:VAL:HG22	1.93	0.50
1:J:346:ASP:HB3	1:J:349:ASN:HB3	1.93	0.50
1:D:90:HIS:O	1:D:91:ARG:NH1	2.45	0.50
1:L:107:PHE:O	1:L:111:VAL:HG12	2.11	0.50
2:O:25:THR:HG22	2:O:34:GLU:HG3	1.93	0.50
1:H:418:GLU:HG2	1:H:422:ARG:HD2	1.92	0.50
1:J:90:HIS:O	1:J:91:ARG:NH1	2.44	0.50
1:G:346:ASP:HB3	1:G:349:ASN:HB3	1.94	0.50
1:A:298:ALA:HB2	1:A:323:VAL:HG22	1.93	0.50
1:C:153:VAL:HB	1:C:234:ILE:HB	1.93	0.50
1:G:251:PHE:O	1:G:255:MET:HG3	2.11	0.50
2:U:49:GLU:OE2	2:U:53:GLN:NE2	2.38	0.50
1:A:99:ILE:HD12	1:A:428:PRO:HG2	1.94	0.50
1:G:332:HIS:O	2:S:60:ARG:NH2	2.45	0.50
1:J:298:ALA:HB2	1:J:323:VAL:HG22	1.93	0.50
1:J:386:ARG:HG2	1:K:455:ILE:O	2.12	0.50
1:F:501:ARG:HG3	1:F:501:ARG:HH11	1.77	0.50
1:G:298:ALA:HB2	1:G:323:VAL:HG22	1.92	0.50
1:A:455:ILE:O	1:L:386:ARG:HG2	2.12	0.49
1:B:83:GLY:O	1:B:139:ARG:NH1	2.45	0.49
2:Q:11:ARG:NH2	2:Q:51:GLU:OE2	2.40	0.49
1:A:346:ASP:HB3	1:A:349:ASN:HB3	1.93	0.49
1:D:332:HIS:O	2:P:60:ARG:NH2	2.45	0.49
1:F:386:ARG:HG2	1:G:455:ILE:O	2.12	0.49
1:L:153:VAL:HB	1:L:234:ILE:HB	1.93	0.49
1:L:298:ALA:HB2	1:L:323:VAL:HG22	1.94	0.49
1:A:332:HIS:O	2:M:60:ARG:NH2	2.45	0.49
1:G:386:ARG:HG2	1:H:455:ILE:O	2.12	0.49
1:F:107:PHE:O	1:F:111:VAL:HG12	2.11	0.49
1:K:354:PHE:HE2	1:L:269:LEU:HD13	1.78	0.49
1:H:242:ASP:OD2	1:I:176:ARG:NH1	2.41	0.49
1:I:386:ARG:HG2	1:J:455:ILE:O	2.12	0.49
1:C:298:ALA:HB2	1:C:323:VAL:HG22	1.94	0.48
2:N:11:ARG:NH2	2:N:51:GLU:OE2	2.40	0.48
1:F:364:ALA:HA	1:G:375:ARG:HE	1.78	0.48

Continued on next page...

For Manuscript Review

Continued from previous page...

Atom-1	Atom-2	Interatomic distance (Å)	Clash overlap (Å)
1:I:61:ASP:OD2	1:I:175:LYS:NZ	2.41	0.48
1:I:298:ALA:HB2	1:I:323:VAL:HG22	1.95	0.48
1:A:132:ARG:HA	1:A:136:MET:HE3	1.94	0.48
1:J:337:ASP:OD2	2:V:60:ARG:NH1	2.35	0.48
1:F:337:ASP:OD2	2:R:60:ARG:NH1	2.36	0.48
1:K:83:GLY:O	1:K:139:ARG:NH1	2.46	0.48
2:X:61:ARG:HG2	2:X:61:ARG:HH11	1.78	0.48
1:C:386:ARG:HG2	1:D:455:ILE:O	2.13	0.48
1:A:386:ARG:HG2	1:B:455:ILE:O	2.14	0.48
1:C:337:ASP:OD2	2:O:60:ARG:NH1	2.37	0.48
1:J:244:GLN:HE21	1:J:247:GLY:HA2	1.79	0.48
1:E:354:PHE:HE2	1:F:269:LEU:HD13	1.77	0.48
1:F:354:PHE:HB2	1:G:348:ASP:HB2	1.95	0.48
1:D:242:ASP:OD2	1:E:176:ARG:NH1	2.45	0.47
1:F:298:ALA:HB2	1:F:323:VAL:HG22	1.94	0.47
1:L:337:ASP:OD2	2:X:60:ARG:NH1	2.37	0.47
2:O:61:ARG:HG2	2:O:61:ARG:HH11	1.78	0.47
1:C:292:MET:SD	2:P:64:ALA:HB2	2.55	0.47
1:C:348:ASP:OD2	1:C:352:SER:OG	2.33	0.47
1:H:193:VAL:HG22	1:H:204:TYR:HD1	1.80	0.47
1:H:83:GLY:O	1:H:139:ARG:NH1	2.46	0.47
1:D:354:PHE:HE2	1:E:269:LEU:HD13	1.79	0.47
1:E:193:VAL:HG22	1:E:204:TYR:HD1	1.80	0.47
1:I:501:ARG:HG3	1:I:501:ARG:HH11	1.80	0.47
1:F:292:MET:SD	2:S:64:ALA:HB2	2.55	0.47
1:H:354:PHE:HE2	1:I:269:LEU:HD13	1.79	0.47
1:A:244:GLN:HE21	1:A:247:GLY:HA2	1.80	0.47
1:L:501:ARG:HG3	1:L:501:ARG:HH11	1.80	0.47
1:E:83:GLY:O	1:E:139:ARG:NH1	2.47	0.47
1:K:193:VAL:HG22	1:K:204:TYR:HD1	1.80	0.46
1:L:289:GLN:HG3	2:M:61:ARG:HE	1.80	0.46
1:B:193:VAL:HG22	1:B:204:TYR:HD1	1.80	0.46
1:D:346:ASP:HB3	1:D:349:ASN:HB3	1.97	0.46
1:C:501:ARG:HG3	1:C:501:ARG:HH11	1.80	0.46
1:H:158:ASP:OD1	1:H:158:ASP:N	2.49	0.46
1:B:354:PHE:HE2	1:C:269:LEU:HD13	1.79	0.46
1:F:353:VAL:HG23	1:G:348:ASP:HB3	1.97	0.46
1:G:354:PHE:HE2	1:H:269:LEU:HD13	1.81	0.46
1:I:292:MET:SD	2:V:64:ALA:HB2	2.55	0.46
1:I:337:ASP:OD2	2:U:60:ARG:NH1	2.36	0.46
1:L:292:MET:SD	2:M:64:ALA:HB2	2.55	0.46

Continued on next page...

For Manuscript Review

Continued from previous page...

Atom-1	Atom-2	Interatomic distance (Å)	Clash overlap (Å)
1:F:158:ASP:OD1	1:F:158:ASP:N	2.49	0.46
1:D:386:ARG:HG2	1:E:455:ILE:O	2.16	0.46
1:J:356:GLN:HG2	1:J:370:TYR:OH	2.16	0.46
1:A:179:ASN:ND2	1:A:185:ASP:OD1	2.49	0.45
1:D:244:GLN:HE21	1:D:247:GLY:HA2	1.80	0.45
1:G:179:ASN:ND2	1:G:185:ASP:OD1	2.50	0.45
1:H:432:ARG:HG2	1:H:433:PHE:CE1	2.51	0.45
2:U:27:GLN:HG3	2:U:32:ARG:HB2	1.97	0.45
1:A:493:GLN:HG3	1:L:474:THR:HG22	1.99	0.45
1:C:61:ASP:OD2	1:C:175:LYS:NZ	2.42	0.45
1:D:354:PHE:HB2	1:E:348:ASP:HB2	1.99	0.45
1:G:356:GLN:HG2	1:G:370:TYR:OH	2.17	0.45
1:J:179:ASN:ND2	1:J:185:ASP:OD1	2.49	0.45
1:D:356:GLN:HG2	1:D:370:TYR:OH	2.17	0.45
1:F:61:ASP:OD2	1:F:175:LYS:NZ	2.41	0.45
1:A:337:ASP:OD2	2:M:60:ARG:NH1	2.32	0.45
1:I:158:ASP:OD1	1:I:158:ASP:N	2.48	0.45
1:C:353:VAL:HG23	1:D:348:ASP:HB3	1.98	0.45
1:J:106:ALA:O	1:J:110:GLU:HG3	2.17	0.45
2:O:27:GLN:HG3	2:O:32:ARG:HB2	1.99	0.44
2:R:27:GLN:HG3	2:R:32:ARG:HB2	1.98	0.44
2:W:48:ALA:O	2:W:51:GLU:HG2	2.17	0.44
1:B:289:GLN:HG3	2:O:61:ARG:HE	1.82	0.44
1:A:354:PHE:HE2	1:B:269:LEU:HD13	1.83	0.44
1:A:356:GLN:HG2	1:A:370:TYR:OH	2.18	0.44
2:T:48:ALA:O	2:T:51:GLU:HG2	2.17	0.44
1:B:288:THR:OG1	2:O:60:ARG:HA	2.18	0.44
1:J:354:PHE:HE2	1:K:269:LEU:HD13	1.83	0.44
1:A:242:ASP:OD2	1:B:176:ARG:NH1	2.47	0.44
1:B:478:GLU:OE1	1:B:478:GLU:HA	2.18	0.44
1:C:356:GLN:HG2	1:C:370:TYR:OH	2.18	0.43
1:J:83:GLY:O	1:J:139:ARG:NH1	2.51	0.43
1:G:83:GLY:O	1:G:139:ARG:NH1	2.51	0.43
1:G:329:LYS:HB3	2:S:63:PRO:HB2	1.99	0.43
1:H:478:GLU:HA	1:H:478:GLU:OE1	2.18	0.43
1:J:329:LYS:HB3	2:V:63:PRO:HB2	1.99	0.43
1:A:83:GLY:O	1:A:139:ARG:NH1	2.51	0.43
1:B:254:VAL:O	1:B:258:MET:HG3	2.19	0.43
1:B:354:PHE:HB2	1:C:348:ASP:HB2	2.01	0.43
1:C:474:THR:HG22	1:D:493:GLN:HG3	2.00	0.43
1:J:35:LEU:HD11	1:K:256:GLU:HG3	2.00	0.43

Continued on next page...

For Manuscript Review

Continued from previous page...

Atom-1	Atom-2	Interatomic distance (Å)	Clash overlap (Å)
1:F:166:ARG:HG3	1:F:422:ARG:HH12	1.83	0.43
1:F:288:THR:OG1	2:S:60:ARG:HA	2.19	0.43
2:W:17:LEU:HD21	2:W:40:VAL:HG22	2.00	0.43
1:G:158:ASP:OD1	1:G:158:ASP:N	2.50	0.43
1:K:254:VAL:O	1:K:258:MET:HG3	2.19	0.43
1:K:289:GLN:HG3	2:X:61:ARG:HE	1.82	0.43
2:X:27:GLN:HG3	2:X:32:ARG:HB2	2.00	0.43
1:I:289:GLN:HG3	2:V:61:ARG:HE	1.84	0.43
1:G:153:VAL:HB	1:G:234:ILE:HB	2.01	0.43
1:G:244:GLN:HE21	1:G:247:GLY:HA2	1.83	0.43
1:H:289:GLN:HG3	2:U:61:ARG:HE	1.84	0.43
1:K:277:MET:HE2	1:L:325:LEU:HA	2.00	0.43
2:N:36:THR:HG22	2:O:22:ARG:HB3	2.00	0.43
2:T:36:THR:HG22	2:U:22:ARG:HB3	2.01	0.43
2:W:36:THR:HG22	2:X:22:ARG:HB3	2.01	0.43
1:C:288:THR:OG1	2:P:60:ARG:HA	2.19	0.43
1:G:70:ALA:O	1:G:74:ILE:HG12	2.19	0.43
1:L:283:GLU:HB3	1:L:338:SER:HB3	2.01	0.43
1:L:288:THR:OG1	2:M:60:ARG:HA	2.19	0.43
2:T:17:LEU:HD21	2:T:40:VAL:HG22	2.01	0.43
1:F:474:THR:HG22	1:G:493:GLN:HG3	2.00	0.43
1:G:337:ASP:OD2	2:S:60:ARG:NH1	2.32	0.43
1:A:70:ALA:O	1:A:74:ILE:HG12	2.19	0.42
1:D:83:GLY:O	1:D:139:ARG:NH1	2.51	0.42
1:G:288:THR:O	1:G:292:MET:HG3	2.19	0.42
1:F:289:GLN:HG3	2:S:61:ARG:HE	1.84	0.42
1:F:356:GLN:HG2	1:F:370:TYR:OH	2.18	0.42
1:J:242:ASP:OD2	1:K:176:ARG:NH1	2.50	0.42
2:R:56:MET:SD	2:R:57:THR:OG1	2.72	0.42
1:D:288:THR:O	1:D:292:MET:HG3	2.19	0.42
1:F:346:ASP:HB3	1:F:349:ASN:HB3	2.02	0.42
1:H:110:GLU:HG2	1:H:425:VAL:HG12	2.01	0.42
2:N:17:LEU:HD21	2:N:40:VAL:HG22	2.02	0.42
1:D:70:ALA:O	1:D:74:ILE:HG12	2.19	0.42
2:Q:17:LEU:HD21	2:Q:40:VAL:HG22	2.02	0.42
1:G:35:LEU:HD11	1:H:256:GLU:HG3	2.01	0.42
1:I:273:ILE:HG21	1:J:325:LEU:HD13	2.01	0.42
1:J:153:VAL:HB	1:J:234:ILE:HB	2.01	0.42
1:I:474:THR:HG22	1:J:493:GLN:HG3	2.01	0.42
1:A:35:LEU:HD11	1:B:256:GLU:HG3	2.01	0.42
1:C:364:ALA:HA	1:D:375:ARG:HE	1.85	0.42

Continued on next page...

For Manuscript Review

Continued from previous page...

Atom-1	Atom-2	Interatomic distance (Å)	Clash overlap (Å)
2:Q:36:THR:HG22	2:R:22:ARG:HB3	2.00	0.42
1:E:348:ASP:OD2	1:E:352:SER:OG	2.36	0.42
1:H:288:THR:OG1	2:U:60:ARG:HA	2.20	0.42
1:E:152:PHE:HB3	1:E:233:PHE:CZ	2.55	0.42
1:E:289:GLN:HG3	2:R:61:ARG:HE	1.84	0.42
1:I:288:THR:OG1	2:V:60:ARG:HA	2.19	0.42
1:J:91:ARG:HA	1:J:91:ARG:HD3	1.91	0.42
1:C:289:GLN:HG3	2:P:61:ARG:HE	1.84	0.42
1:E:254:VAL:O	1:E:258:MET:HG3	2.20	0.42
1:D:35:LEU:HD11	1:E:256:GLU:HG3	2.01	0.41
1:D:238:GLU:O	1:D:238:GLU:HG3	2.20	0.41
1:L:329:LYS:HB3	2:X:63:PRO:HB2	2.02	0.41
1:J:70:ALA:O	1:J:74:ILE:HG12	2.19	0.41
1:L:61:ASP:OD2	1:L:175:LYS:NZ	2.40	0.41
1:A:96:TYR:CE1	1:B:117:GLU:HG3	2.55	0.41
1:F:494:VAL:O	1:F:497:THR:HG22	2.20	0.41
1:H:254:VAL:O	1:H:258:MET:HG3	2.19	0.41
1:H:354:PHE:HB2	1:I:348:ASP:HB2	2.02	0.41
1:L:430:LYS:HD3	1:L:430:LYS:N	2.35	0.41
1:C:329:LYS:HB3	2:O:63:PRO:HB2	2.03	0.41
1:A:150:GLU:OE1	1:A:235:HIS:NE2	2.52	0.41
1:C:158:ASP:OD1	1:C:158:ASP:N	2.49	0.41
1:C:238:GLU:HG3	1:C:238:GLU:O	2.20	0.41
1:A:153:VAL:HB	1:A:234:ILE:HB	2.01	0.41
1:B:158:ASP:OD1	1:B:158:ASP:N	2.49	0.41
1:I:329:LYS:HB3	2:U:63:PRO:HB2	2.02	0.41
1:I:346:ASP:HB3	1:I:349:ASN:HB3	2.03	0.41
1:K:152:PHE:HB3	1:K:233:PHE:CZ	2.55	0.41
1:L:238:GLU:HG3	1:L:238:GLU:O	2.20	0.41
1:G:434:SER:OG	1:G:435:PHE:N	2.53	0.41
1:D:474:THR:HG22	1:E:493:GLN:HG3	2.03	0.41
1:H:152:PHE:HB3	1:H:233:PHE:CZ	2.55	0.41
1:J:474:THR:HG22	1:K:493:GLN:HG3	2.03	0.41
1:A:238:GLU:O	1:A:238:GLU:HG3	2.20	0.41
1:A:474:THR:HG22	1:B:493:GLN:HG3	2.03	0.41
1:F:238:GLU:O	1:F:238:GLU:HG3	2.20	0.41
1:F:329:LYS:HB3	2:R:63:PRO:HB2	2.03	0.41
1:I:356:GLN:HG2	1:I:370:TYR:OH	2.20	0.41
1:D:91:ARG:HA	1:D:91:ARG:HD3	1.91	0.41
1:G:238:GLU:O	1:G:238:GLU:HG3	2.20	0.41
1:G:474:THR:HG22	1:H:493:GLN:HG3	2.03	0.41

Continued on next page...

For Manuscript Review

Continued from previous page...

Atom-1	Atom-2	Interatomic distance (Å)	Clash overlap (Å)
1:K:288:THR:OG1	2:X:60:ARG:HA	2.21	0.41
1:K:329:LYS:HB3	2:W:63:PRO:HB2	2.03	0.41
1:L:158:ASP:OD1	1:L:158:ASP:N	2.48	0.41
1:A:129:GLU:HG2	1:L:199:GLY:HA3	2.04	0.40
1:E:329:LYS:HB3	2:Q:63:PRO:HB2	2.04	0.40
1:G:91:ARG:HA	1:G:91:ARG:HD3	1.91	0.40
1:I:401:LYS:HA	1:I:401:LYS:HD2	1.92	0.40
2:Q:31:ARG:H	2:Q:31:ARG:HG3	1.74	0.40
1:A:133:THR:O	1:A:137:MET:HG3	2.21	0.40
1:A:288:THR:O	1:A:292:MET:HG3	2.20	0.40
1:E:288:THR:OG1	2:R:60:ARG:HA	2.20	0.40
1:I:62:ASP:OD1	1:I:66:ASN:ND2	2.55	0.40
1:I:199:GLY:HA3	1:J:129:GLU:HG2	2.03	0.40
1:I:354:PHE:HE2	1:J:269:LEU:HD13	1.86	0.40
1:J:288:THR:O	1:J:292:MET:HG3	2.21	0.40
1:E:88:LEU:HD13	1:E:447:TRP:CH2	2.56	0.40
1:L:356:GLN:HG2	1:L:370:TYR:OH	2.20	0.40
1:G:501:ARG:NH1	1:G:501:ARG:HG2	2.37	0.40
1:H:329:LYS:HB3	2:T:63:PRO:HB2	2.04	0.40
1:H:420:ILE:HD13	1:H:427:LEU:HD21	2.02	0.40
1:J:238:GLU:O	1:J:238:GLU:HG3	2.20	0.40
1:H:88:LEU:HD13	1:H:447:TRP:CH2	2.57	0.40
1:I:238:GLU:O	1:I:238:GLU:HG3	2.20	0.40

There are no symmetry-related clashes.

5.3 Torsion angles (i)

5.3.1 Protein backbone (i)

In the following table, the Percentiles column shows the percent Ramachandran outliers of the chain as a percentile score with respect to all PDB entries followed by that with respect to all EM entries.

The Analysed column shows the number of residues for which the backbone conformation was analysed, and the total number of residues.

Mol	Chain	Analysed	Favoured	Allowed	Outliers	Percentiles	
1	A	462/533 (87%)	455 (98%)	7 (2%)	0	100	100
1	B	462/533 (87%)	454 (98%)	8 (2%)	0	100	100

Continued on next page...

For Manuscript Review

Continued from previous page...

Mol	Chain	Analysed	Favoured	Allowed	Outliers	Percentiles	
1	C	462/533 (87%)	456 (99%)	6 (1%)	0	100	100
1	D	462/533 (87%)	454 (98%)	8 (2%)	0	100	100
1	E	462/533 (87%)	453 (98%)	9 (2%)	0	100	100
1	F	462/533 (87%)	455 (98%)	7 (2%)	0	100	100
1	G	462/533 (87%)	456 (99%)	6 (1%)	0	100	100
1	H	462/533 (87%)	454 (98%)	8 (2%)	0	100	100
1	I	462/533 (87%)	456 (99%)	6 (1%)	0	100	100
1	J	462/533 (87%)	454 (98%)	8 (2%)	0	100	100
1	K	462/533 (87%)	453 (98%)	9 (2%)	0	100	100
1	L	462/533 (87%)	456 (99%)	6 (1%)	0	100	100
2	M	63/68 (93%)	62 (98%)	1 (2%)	0	100	100
2	N	63/68 (93%)	61 (97%)	2 (3%)	0	100	100
2	O	63/68 (93%)	62 (98%)	1 (2%)	0	100	100
2	P	63/68 (93%)	62 (98%)	1 (2%)	0	100	100
2	Q	63/68 (93%)	61 (97%)	2 (3%)	0	100	100
2	R	63/68 (93%)	62 (98%)	1 (2%)	0	100	100
2	S	63/68 (93%)	62 (98%)	1 (2%)	0	100	100
2	T	63/68 (93%)	61 (97%)	2 (3%)	0	100	100
2	U	63/68 (93%)	62 (98%)	1 (2%)	0	100	100
2	V	63/68 (93%)	62 (98%)	1 (2%)	0	100	100
2	W	63/68 (93%)	61 (97%)	2 (3%)	0	100	100
2	X	63/68 (93%)	62 (98%)	1 (2%)	0	100	100
All	All	6300/7212 (87%)	6196 (98%)	104 (2%)	0	100	100

There are no Ramachandran outliers to report.

5.3.2 Protein sidechains (i)

In the following table, the Percentiles column shows the percent sidechain outliers of the chain as a percentile score with respect to all PDB entries followed by that with respect to all EM entries.

The Analysed column shows the number of residues for which the sidechain conformation was analysed, and the total number of residues.

For Manuscript Review

Mol	Chain	Analysed	Rotameric	Outliers	Percentiles	
1	A	377/428 (88%)	377 (100%)	0	100	100
1	B	377/428 (88%)	377 (100%)	0	100	100
1	C	377/428 (88%)	377 (100%)	0	100	100
1	D	377/428 (88%)	377 (100%)	0	100	100
1	E	377/428 (88%)	377 (100%)	0	100	100
1	F	377/428 (88%)	377 (100%)	0	100	100
1	G	377/428 (88%)	376 (100%)	1 (0%)	92	96
1	H	377/428 (88%)	377 (100%)	0	100	100
1	I	377/428 (88%)	377 (100%)	0	100	100
1	J	377/428 (88%)	377 (100%)	0	100	100
1	K	377/428 (88%)	377 (100%)	0	100	100
1	L	377/428 (88%)	377 (100%)	0	100	100
2	M	51/54 (94%)	50 (98%)	1 (2%)	55	80
2	N	51/54 (94%)	51 (100%)	0	100	100
2	O	51/54 (94%)	51 (100%)	0	100	100
2	P	51/54 (94%)	50 (98%)	1 (2%)	55	80
2	Q	51/54 (94%)	51 (100%)	0	100	100
2	R	51/54 (94%)	51 (100%)	0	100	100
2	S	51/54 (94%)	50 (98%)	1 (2%)	55	80
2	T	51/54 (94%)	50 (98%)	1 (2%)	55	80
2	U	51/54 (94%)	50 (98%)	1 (2%)	55	80
2	V	51/54 (94%)	50 (98%)	1 (2%)	55	80
2	W	51/54 (94%)	51 (100%)	0	100	100
2	X	51/54 (94%)	51 (100%)	0	100	100
All	All	5136/5784 (89%)	5129 (100%)	7 (0%)	93	98

All (7) residues with a non-rotameric sidechain are listed below:

Mol	Chain	Res	Type
1	G	117	GLU
2	M	5	GLU
2	P	5	GLU
2	S	5	GLU
2	T	5	GLU
2	U	5	GLU

Continued on next page...

For Manuscript Review

Continued from previous page...

Mol	Chain	Res	Type
2	V	5	GLU

Sometimes sidechains can be flipped to improve hydrogen bonding and reduce clashes. All (1) such sidechains are listed below:

Mol	Chain	Res	Type
2	Q	27	GLN

5.3.3 RNA (i)

There are no RNA molecules in this entry.

5.4 Non-standard residues in protein, DNA, RNA chains (i)

There are no non-standard protein/DNA/RNA residues in this entry.

5.5 Carbohydrates (i)

There are no monosaccharides in this entry.

5.6 Ligand geometry (i)

There are no ligands in this entry.

5.7 Other polymers (i)

There are no such residues in this entry.

5.8 Polymer linkage issues (i)

There are no chain breaks in this entry.

For Manuscript Review

6 Map visualisation (i)

This section contains visualisations of the EMDB entry EMD-36847. These allow visual inspection of the internal detail of the map and identification of artifacts.

Images derived from a raw map, generated by summing the deposited half-maps, are presented below the corresponding image components of the primary map to allow further visual inspection and comparison with those of the primary map.

6.1 Orthogonal projections (i)

6.1.1 Primary map

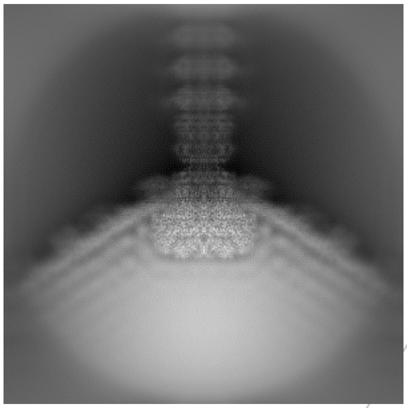

X

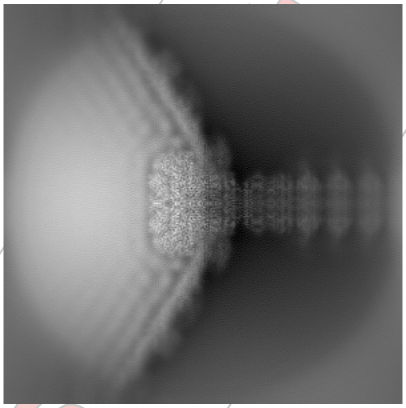

Y

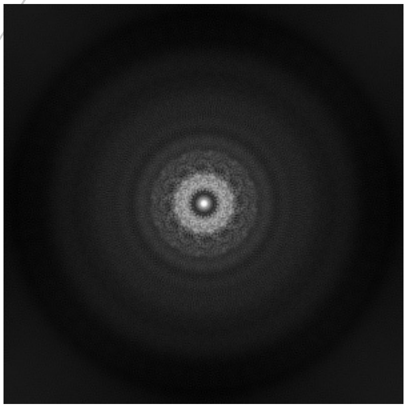

Z

6.1.2 Raw map

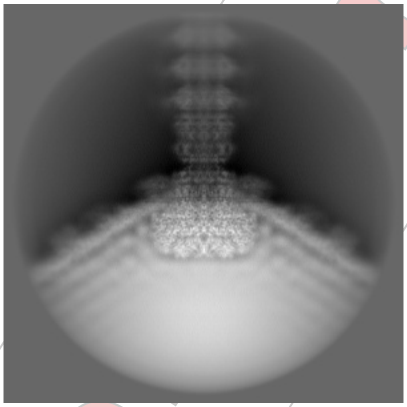

X

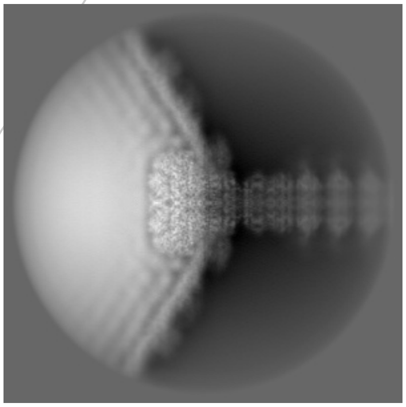

Y

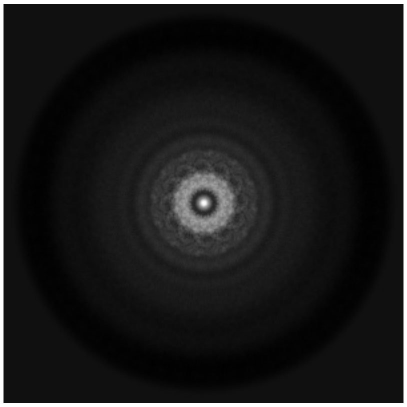

Z

The images above show the map projected in three orthogonal directions.

For Manuscript Review

6.2 Central slices (i)

6.2.1 Primary map

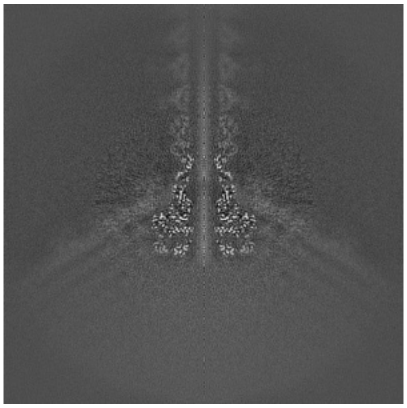
X Index: 200

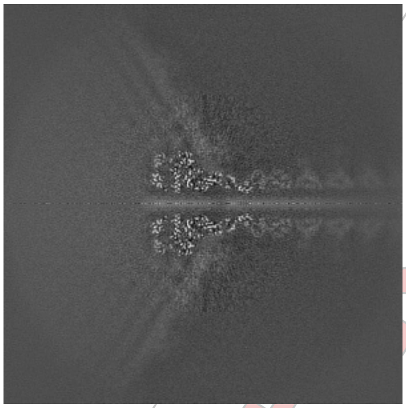
Y Index: 200

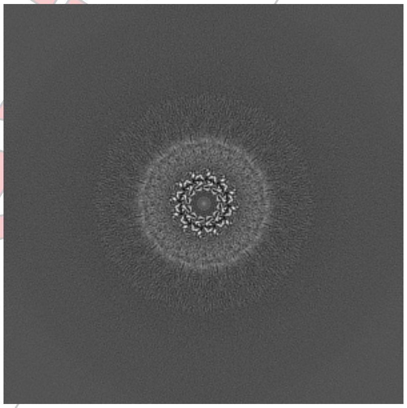
Z Index: 200

6.2.2 Raw map

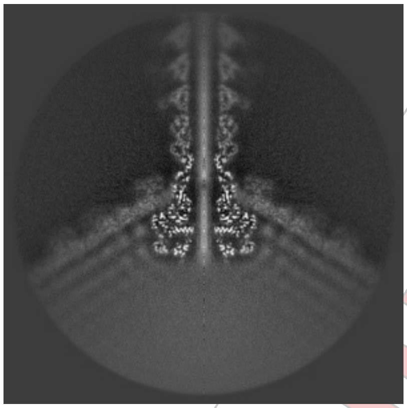
X Index: 200

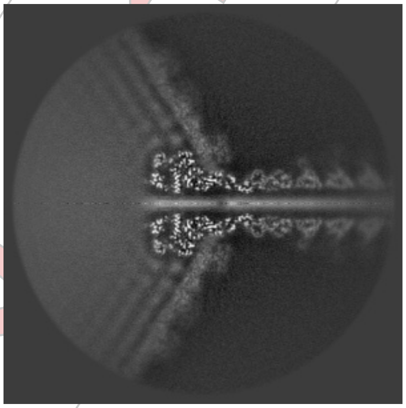
Y Index: 200

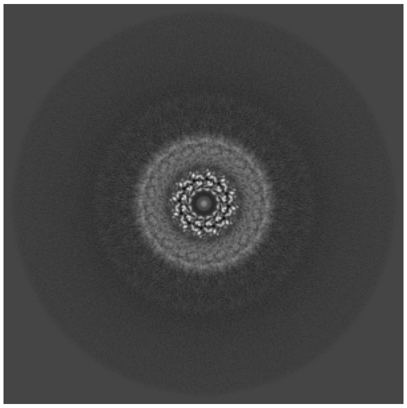
Z Index: 200

The images above show central slices of the map in three orthogonal directions.

For Manuscript Review

6.3 Largest variance slices (i)

6.3.1 Primary map

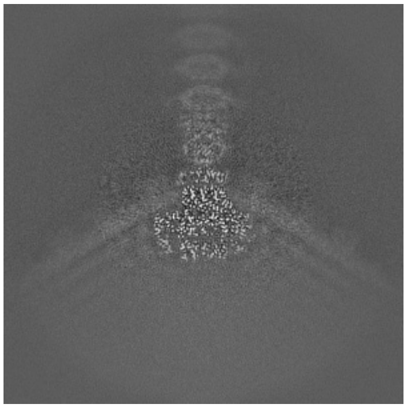
X Index: 220

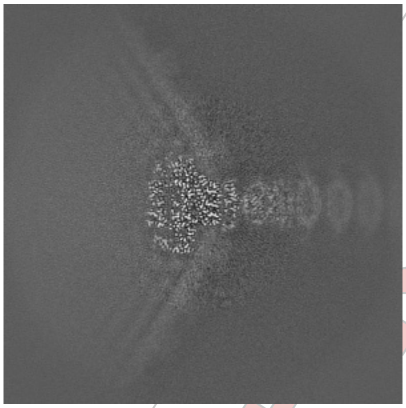
Y Index: 180

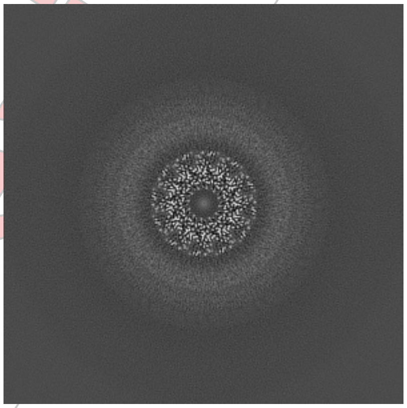
Z Index: 186

6.3.2 Raw map

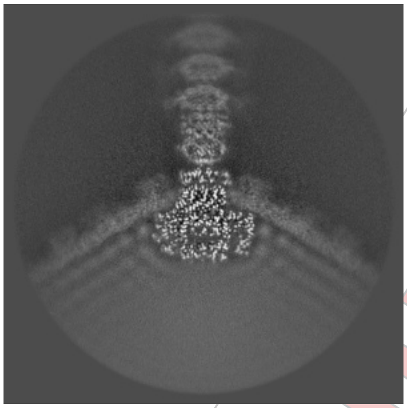
X Index: 180

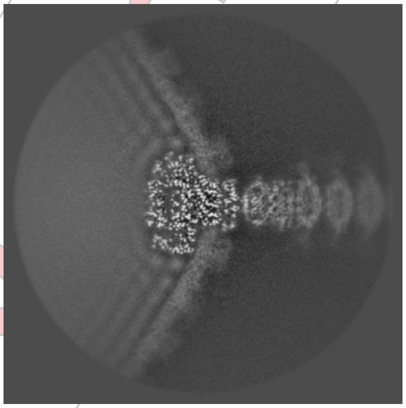
Y Index: 180

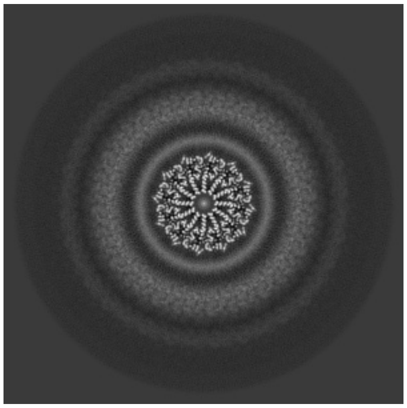
Z Index: 173

The images above show the largest variance slices of the map in three orthogonal directions.

For Manuscript Review

6.4 Orthogonal standard-deviation projections (False-color) (i)

6.4.1 Primary map

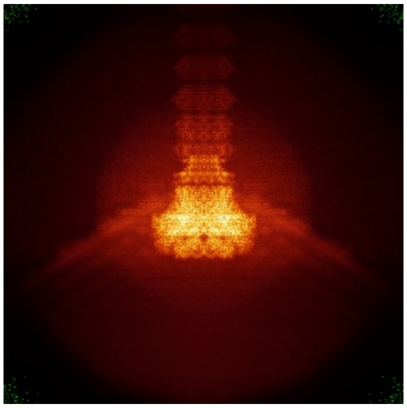
X

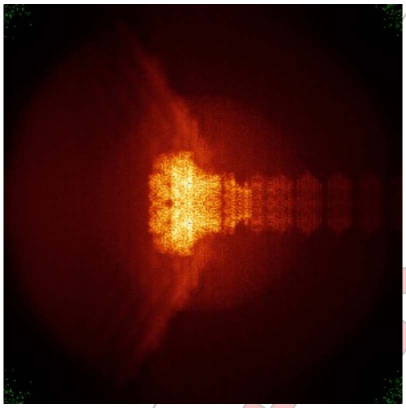
Y

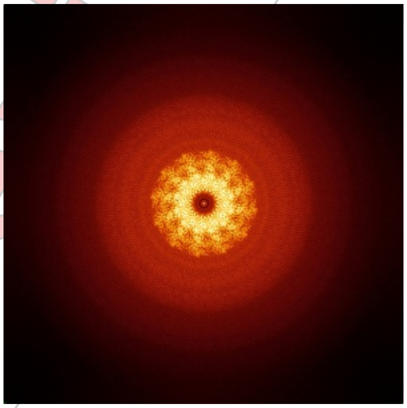
Z

6.4.2 Raw map

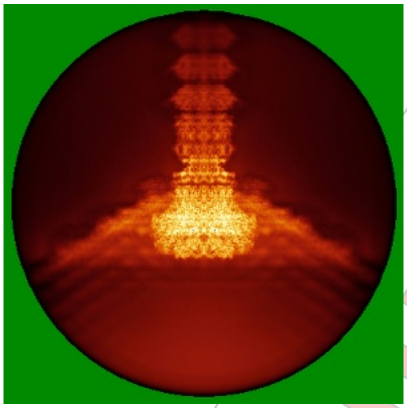
X

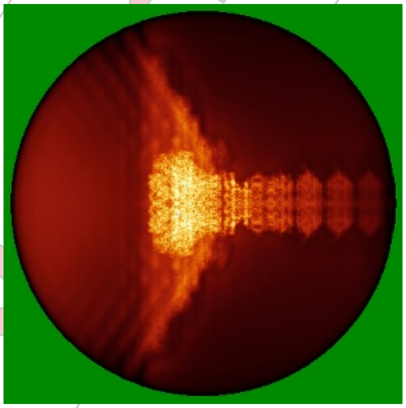
Y

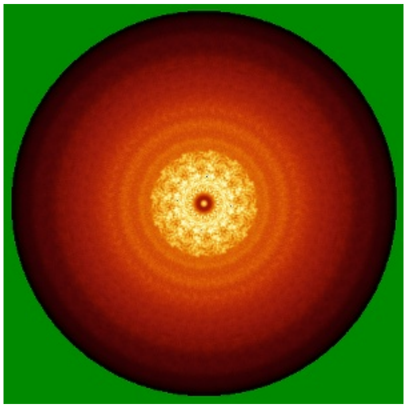
Z

The images above show the map standard deviation projections with false color in three orthogonal directions. Minimum values are shown in green, max in blue, and dark to light orange shades represent small to large values respectively.

For Manuscript Review

6.5 Orthogonal surface views (i)

6.5.1 Primary map

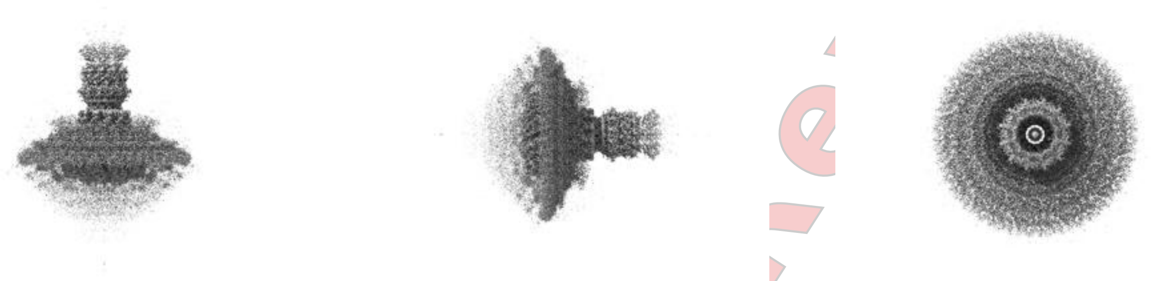

X Y Z

The images above show the 3D surface view of the map at the recommended contour level 5.5. These images, in conjunction with the slice images, may facilitate assessment of whether an appropriate contour level has been provided.

6.5.2 Raw map

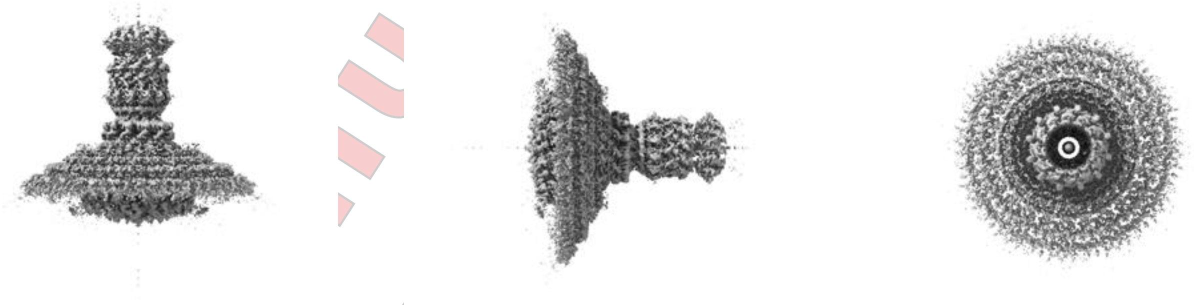

X Y Z

These images show the 3D surface of the raw map. The raw map's contour level was selected so that its surface encloses the same volume as the primary map does at its recommended contour level.

6.6 Mask visualisation (i)

This section was not generated. No masks/segmentation were deposited.

For Manuscript Review

7 Map analysis (i)

This section contains the results of statistical analysis of the map.

7.1 Map-value distribution (i)

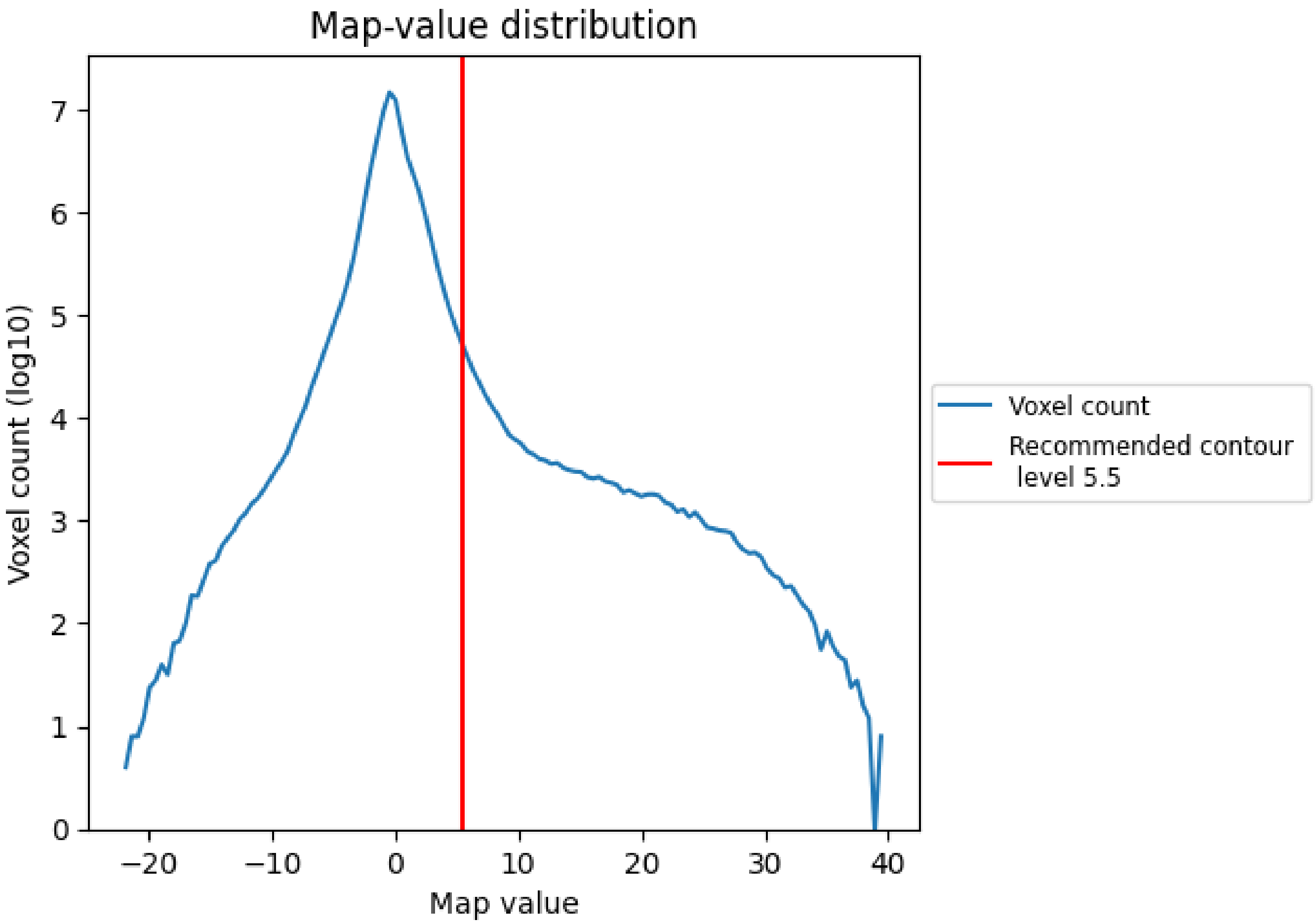

The map-value distribution is plotted in 128 intervals along the x-axis. The y-axis is logarithmic. A spike in this graph at zero usually indicates that the volume has been masked.

For Manuscript Review

7.2 Volume estimate (i)

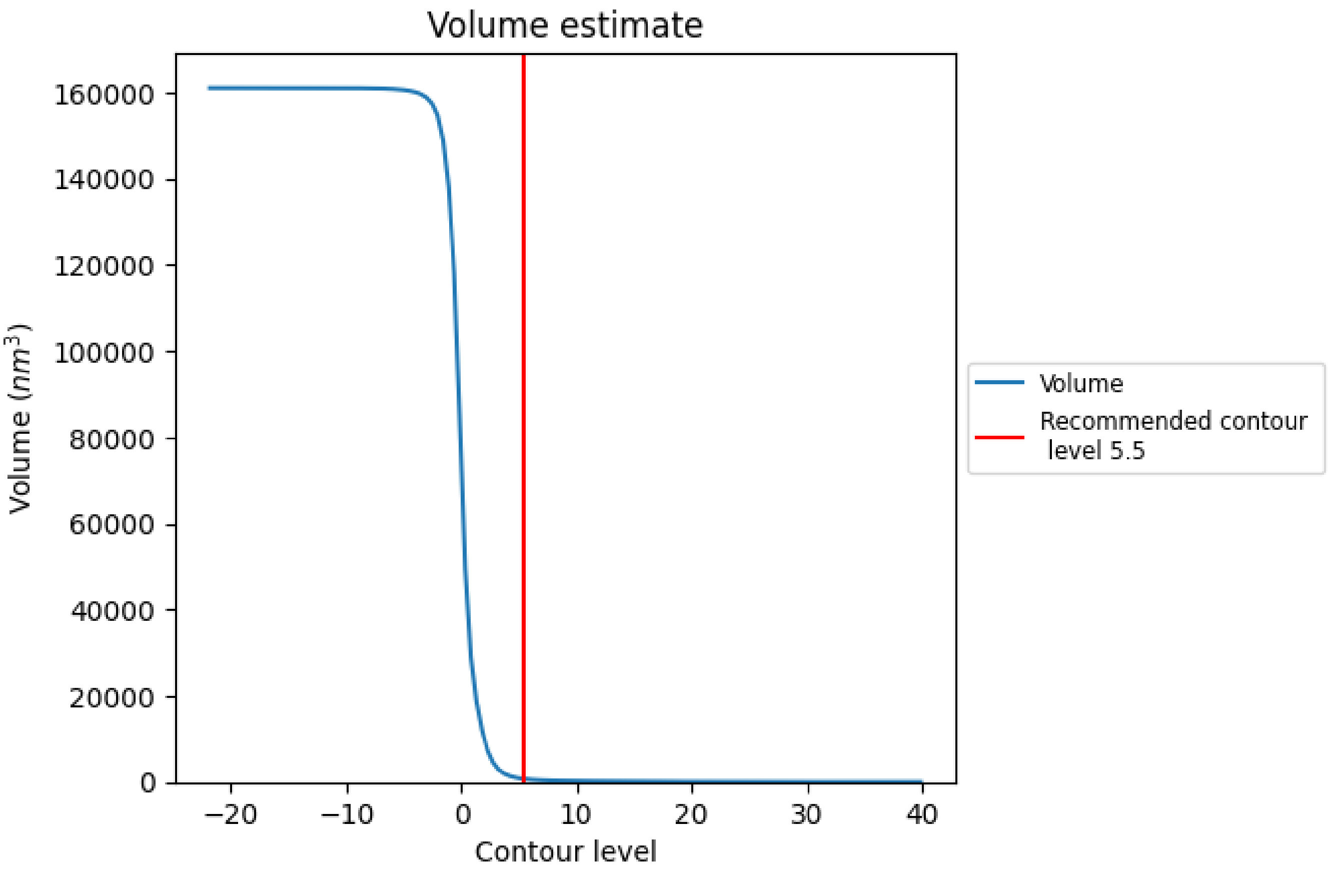

The volume at the recommended contour level is 708 nm^3; this corresponds to an approximate mass of 640 kDa.

The volume estimate graph shows how the enclosed volume varies with the contour level. The recommended contour level is shown as a vertical line and the intersection between the line and the curve gives the volume of the enclosed surface at the given level.

For Manuscript Review

7.3 Rotationally averaged power spectrum (i)

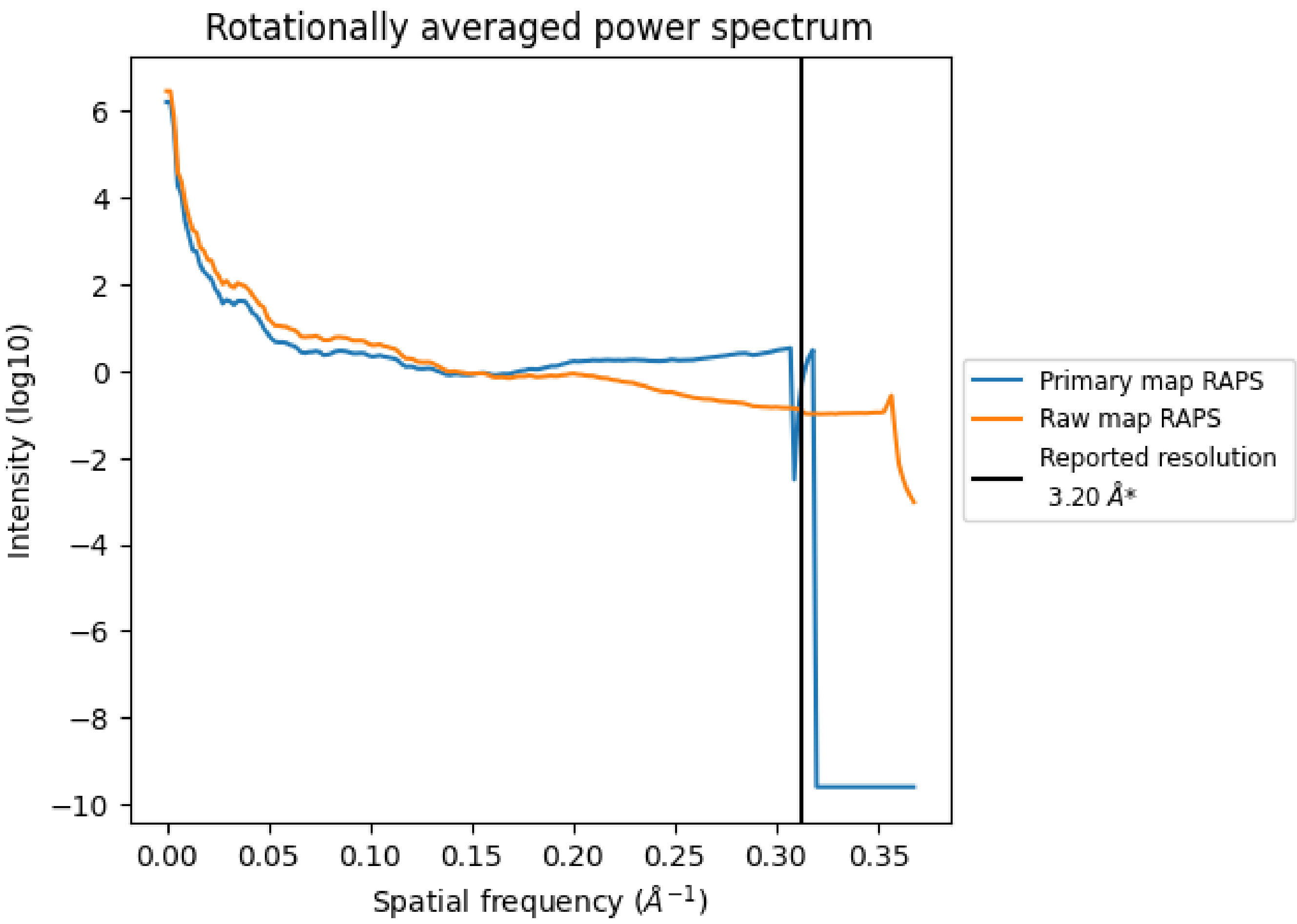

*Reported resolution corresponds to spatial frequency of 0.312 $Å^{-1}$

For Manuscript Review

8 Fourier-Shell correlation (i)

Fourier-Shell Correlation (FSC) is the most commonly used method to estimate the resolution of single-particle and subtomogram-averaged maps. The shape of the curve depends on the imposed symmetry, mask and whether or not the two 3D reconstructions used were processed from a common reference. The reported resolution is shown as a black line. A curve is displayed for the half-bit criterion in addition to lines showing the 0.143 gold standard cut-off and 0.5 cut-off.

8.1 FSC (i)

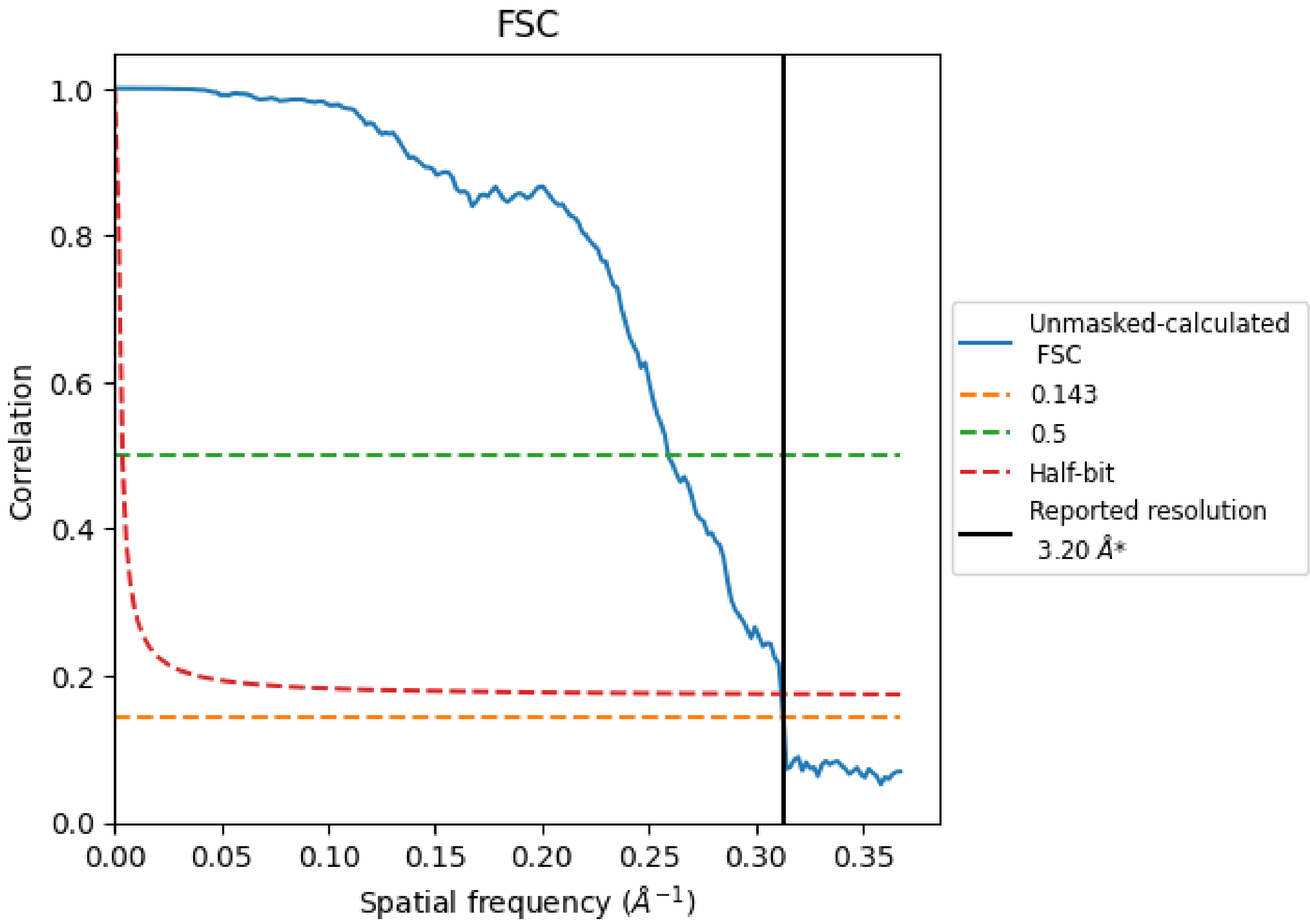

*Reported resolution corresponds to spatial frequency of 0.312 Å^{-1}

For Manuscript Review

8.2 Resolution estimates (i)

Resolution estimate (Å)	Estimation criterion (FSC cut-off)		
	0.143	0.5	Half-bit
Reported by author	3.20	-	-
Author-provided FSC curve	-	-	-
Unmasked-calculated*	3.20	3.86	3.21

*Resolution estimate based on FSC curve calculated by comparison of deposited half-maps.

For Manuscript Review

9 Map-model fit (i)

This section contains information regarding the fit between EMDB map EMD-36847 and PDB model 8K38. Per-residue inclusion information can be found in section 3 on page 6.

9.1 Map-model overlay (i)

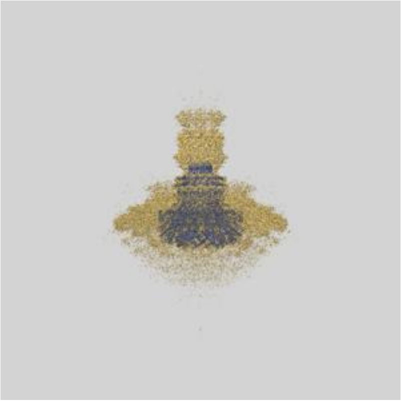

X

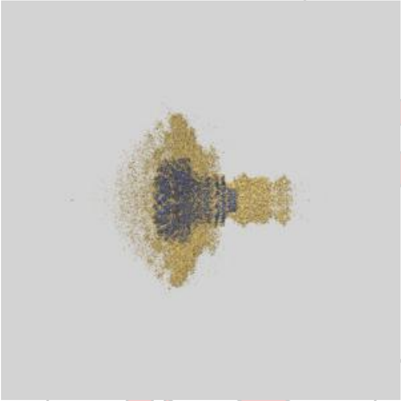

Y

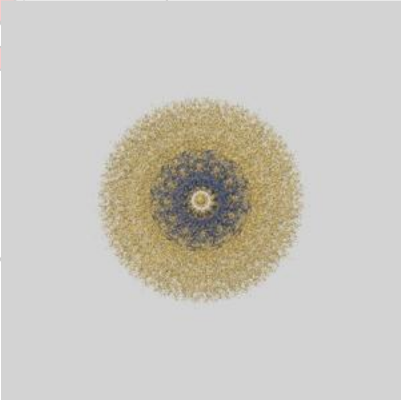

Z

The images above show the 3D surface view of the map at the recommended contour level 5.5 at 50% transparency in yellow overlaid with a ribbon representation of the model coloured in blue. These images allow for the visual assessment of the quality of fit between the atomic model and the map.

For Manuscript Review

9.2 Q-score mapped to coordinate model (i)

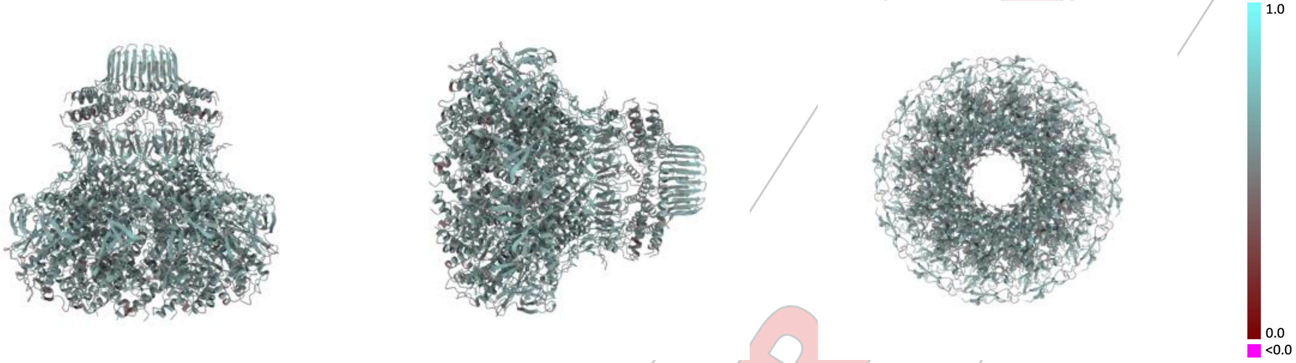

X Y Z

The images above show the model with each residue coloured according its Q-score. This shows their resolvability in the map with higher Q-score values reflecting better resolvability. Please note: Q-score is calculating the resolvability of atoms, and thus high values are only expected at resolutions at which atoms can be resolved. Low Q-score values may therefore be expected for many entries.

9.3 Atom inclusion mapped to coordinate model (i)

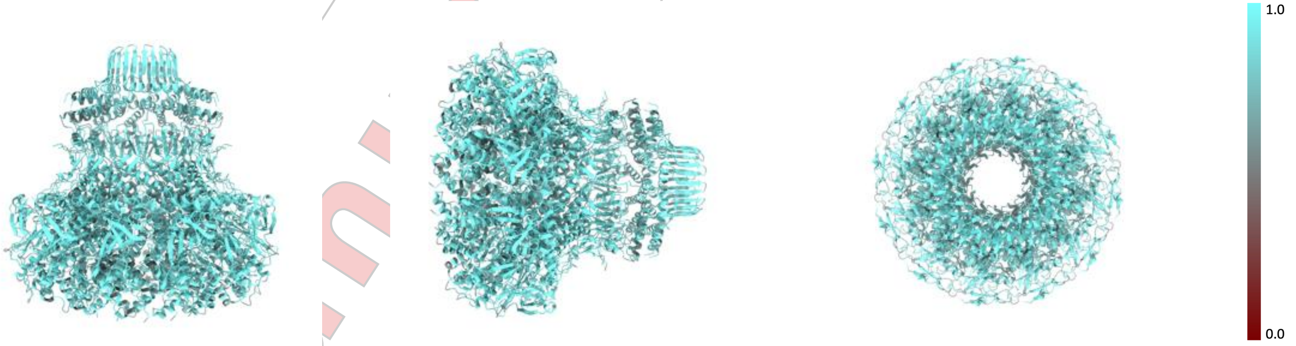

X Y Z

The images above show the model with each residue coloured according to its atom inclusion. This shows to what extent they are inside the map at the recommended contour level (5.5).

For Manuscript Review

9.4 Atom inclusion (i)

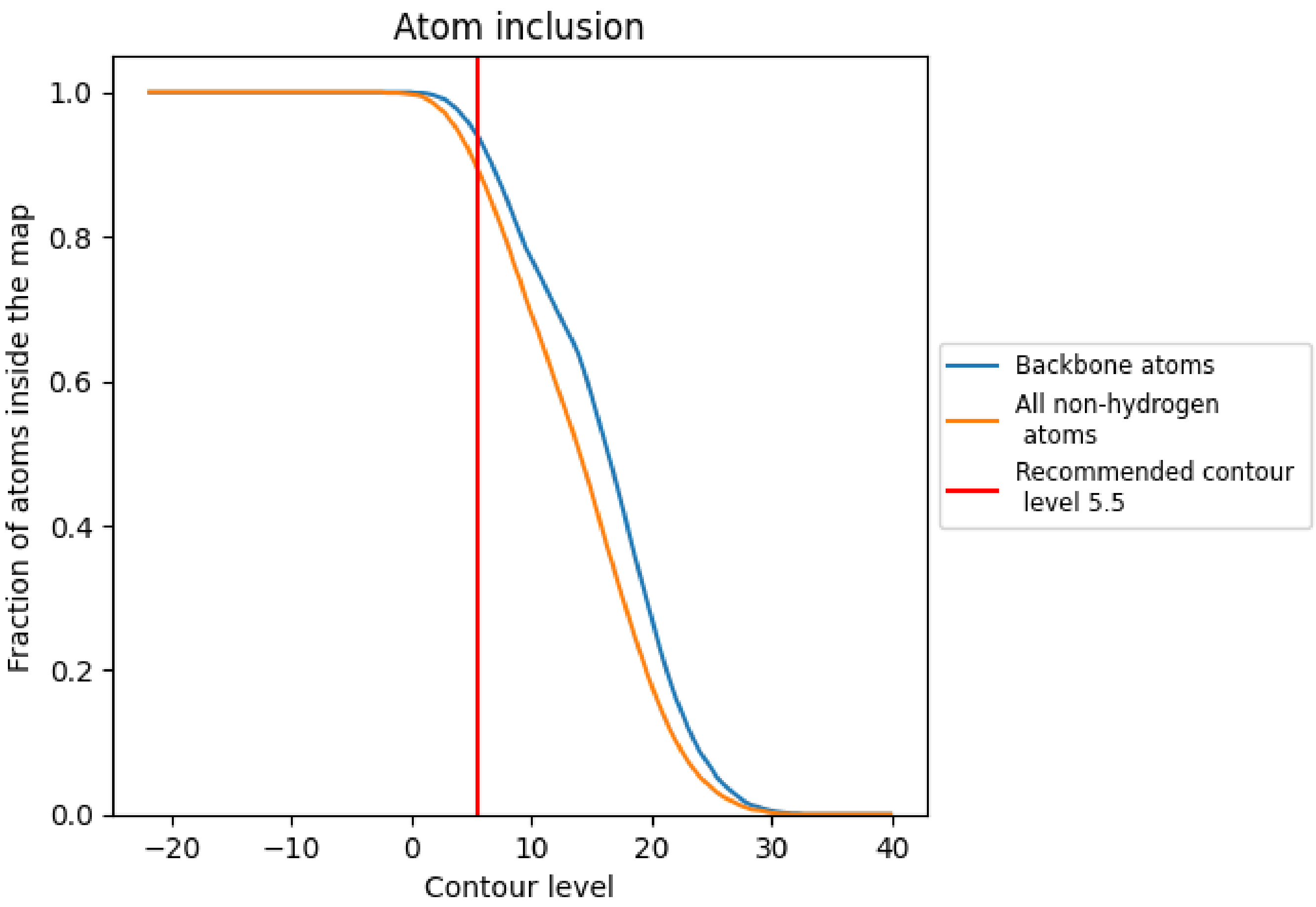

At the recommended contour level, 94% of all backbone atoms, 89% of all non-hydrogen atoms, are inside the map.

For Manuscript Review

9.5 Map-model fit summary (i)

The table lists the average atom inclusion at the recommended contour level (5.5) and Q-score for the entire model and for each chain.

Chain	Atom inclusion	Q-score
All	0.8930	0.5610
A	0.9000	0.5610
B	0.8990	0.5630
C	0.8950	0.5600
D	0.8970	0.5610
E	0.8990	0.5620
F	0.8960	0.5620
G	0.8990	0.5620
H	0.8970	0.5630
I	0.8980	0.5600
J	0.8980	0.5620
K	0.8980	0.5620
L	0.8970	0.5600
M	0.8550	0.5550
N	0.8650	0.5550
O	0.8610	0.5530
P	0.8570	0.5540
Q	0.8670	0.5570
R	0.8630	0.5550
S	0.8530	0.5520
T	0.8780	0.5550
U	0.8610	0.5500
V	0.8530	0.5480
W	0.8740	0.5550
X	0.8650	0.5500

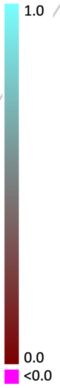

For Manuscript Review
