# Supplementary material for: Structure of the siphophage neck–Tail complex suggests that conserved tail tip proteins facilitate receptor binding and tail assembly
Source: PLoS Biol. 2023 Dec 14;21(12):e3002441. doi: 10.1371/journal.pbio.3002441 (PMC10721106; doi:10.1371/journal.pbio.3002441)
Supplement: S1 Appendix — (ZIP) [file pbio.3002441.s021.zip › PDB Validation Report.zip!/D_1300038919_report-tail-tip.pdf]

Full wwPDB EM Validation Report (i)

Jul 19, 2023 – 05:09 PM JST

PDB ID	:	8K35
EMDB ID	:	EMD-36844
Title	:	Structure of the bacteriophage lambda tail tip complex
Deposited on	:	2023-07-14
Resolution	:	3.44 Å(reported)

This wwPDB validation report is for manuscript review

This is a Full wwPDB EM Validation Report.

This report is produced by the wwPDB biocuration pipeline after annotation of the structure.

We welcome your comments at *validation@mail.wwpdb.org*
A user guide is available at
https://www.wwpdb.org/validation/2017/EMValidationReportHelp
with specific help available everywhere you see the (i) symbol.

The types of validation reports are described at
http://www.wwpdb.org/validation/2017/FAQs#types.

The following versions of software and data (see references (i)) were used in the production of this report:

EMDB validation analysis	:	0.0.1.dev50
Mogul	:	1.8.5 (274361), CSD as541be (2020)
MolProbity	:	4.02b-467
Percentile statistics	:	20191225.v01 (using entries in the PDB archive December 25th 2019)
MapQ	:	1.9.9
Ideal geometry (proteins)	:	Engh & Huber (2001)
Ideal geometry (DNA, RNA)	:	Parkinson et al. (1996)
Validation Pipeline (wwPDB-VP)	:	2.34

For Manuscript Review

1 Overall quality at a glance (i)

The following experimental techniques were used to determine the structure:
ELECTRON MICROSCOPY

The reported resolution of this entry is 3.44 Å.

Percentile scores (ranging between 0-100) for global validation metrics of the entry are shown in the following graphic. The table shows the number of entries on which the scores are based.

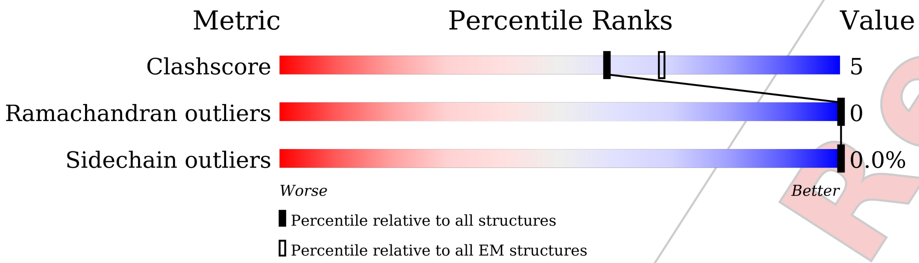

Metric	Whole archive (#Entries)	EM structures (#Entries)
Clashscore	158937	4297
Ramachandran outliers	154571	4023
Sidechain outliers	154315	3826

The table below summarises the geometric issues observed across the polymeric chains and their fit to the map. The red, orange, yellow and green segments of the bar indicate the fraction of residues that contain outliers for >=3, 2, 1 and 0 types of geometric quality criteria respectively. A grey segment represents the fraction of residues that are not modelled. The numeric value for each fraction is indicated below the corresponding segment, with a dot representing fractions <=5% The upper red bar (where present) indicates the fraction of residues that have poor fit to the EM map (all-atom inclusion < 40%). The numeric value is given above the bar.

Mol	Chain	Length	Quality of chain
1	A	1132	• 64% 11% 25%
1	B	1132	• 64% 11% 25%
1	I	1132	• 65% 10% 25%
2	G	232	91% 9%
2	J	232	91% 9%
2	N	232	91% 9%
3	C	109	90% 10%
3	D	109	94% 6%

Continued on next page...

For Manuscript Review

Continued from previous page...

Mol	Chain	Length	Quality of chain
3	E	109	94% 6%
3	F	109	88% 12%
3	H	109	90% 10%
3	O	109	89% 11%
4	K	223	34% 6% 60%
4	Q	223	36% • 60%
4	x	223	40% 60%
5	L	246	50% 12% 38%
5	R	246	55% 7% 38%
5	T	246	54% 9% 38%
5	U	246	53% 9% 38%
5	V	246	52% 10% 38%
5	W	246	52% 10% 38%
6	M	853	• 96%
6	P	853	• 96%
6	S	853	• 96%

For Manuscript Review

2 Entry composition (i)

There are 7 unique types of molecules in this entry. The entry contains 40353 atoms, of which 0 are hydrogens and 0 are deuteriums.

In the tables below, the AltConf column contains the number of residues with at least one atom in alternate conformation and the Trace column contains the number of residues modelled with at most 2 atoms.

- Molecule 1 is a protein called Tip attachment protein J.

Mol	Chain	Residues	Atoms					AltConf	Trace
1	I	851	Total 6633	C 4156	N 1156	O 1301	S 20	0	0
1	A	851	Total 6633	C 4156	N 1156	O 1301	S 20	0	0
1	B	851	Total 6633	C 4156	N 1156	O 1301	S 20	0	0

- Molecule 2 is a protein called Tail tip protein L.

Mol	Chain	Residues	Atoms					AltConf	Trace
2	J	232	Total 1801	C 1117	N 309	O 362	S 13	0	0
2	G	232	Total 1801	C 1117	N 309	O 362	S 13	0	0
2	N	232	Total 1801	C 1117	N 309	O 362	S 13	0	0

- Molecule 3 is a protein called Tail tip protein M.

Mol	Chain	Residues	Atoms					AltConf	Trace
3	H	109	Total 884	C 569	N 154	O 157	S 4	0	0
3	O	109	Total 884	C 569	N 154	O 157	S 4	0	0
3	C	109	Total 884	C 569	N 154	O 157	S 4	0	0
3	E	109	Total 884	C 569	N 154	O 157	S 4	0	0
3	D	109	Total 884	C 569	N 154	O 157	S 4	0	0
3	F	109	Total 884	C 569	N 154	O 157	S 4	0	0

- Molecule 4 is a protein called Tail tip assembly protein I.

For Manuscript Review

Mol	Chain	Residues	Atoms					AltConf	Trace
4	x	89	Total 652	C 403	N 116	O 128	S 5	0	0
4	K	89	Total 652	C 403	N 116	O 128	S 5	0	0
4	Q	89	Total 652	C 403	N 116	O 128	S 5	0	0

- Molecule 5 is a protein called Tail tube protein.

Mol	Chain	Residues	Atoms					AltConf	Trace
5	L	153	Total 1174	C 736	N 199	O 236	S 3	0	0
5	R	153	Total 1174	C 736	N 199	O 236	S 3	0	0
5	T	153	Total 1174	C 736	N 199	O 236	S 3	0	0
5	U	153	Total 1174	C 736	N 199	O 236	S 3	0	0
5	V	153	Total 1174	C 736	N 199	O 236	S 3	0	0
5	W	153	Total 1174	C 736	N 199	O 236	S 3	0	0

- Molecule 6 is a protein called Tape measure protein.

Mol	Chain	Residues	Atoms					AltConf	Trace
6	P	32	Total 241	C 149	N 45	O 45	S 2	0	0
6	M	32	Total 241	C 149	N 45	O 45	S 2	0	0
6	S	32	Total 241	C 149	N 45	O 45	S 2	0	0

- Molecule 7 is IRON/SULFUR CLUSTER (three-letter code: SF4) (formula: Fe_4S_4).

For Manuscript Review

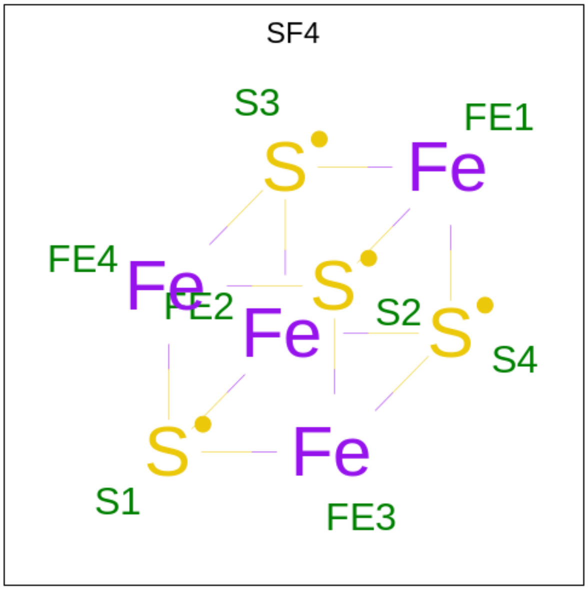

Mol	Chain	Residues	Atoms	AltConf
7	J	1	Total Fe S<br>8 4 4	0
7	G	1	Total Fe S<br>8 4 4	0
7	N	1	Total Fe S<br>8 4 4	0

For Manuscript Review

3 Residue-property plots (i)

These plots are drawn for all protein, RNA, DNA and oligosaccharide chains in the entry. The first graphic for a chain summarises the proportions of the various outlier classes displayed in the second graphic. The second graphic shows the sequence view annotated by issues in geometry and atom inclusion in map density. Residues are color-coded according to the number of geometric quality criteria for which they contain at least one outlier: green = 0, yellow = 1, orange = 2 and red = 3 or more. A red diamond above a residue indicates a poor fit to the EM map for this residue (all-atom inclusion < 40%). Stretches of 2 or more consecutive residues without any outlier are shown as a green connector. Residues present in the sample, but not in the model, are shown in grey.

- Molecule 1: Tip attachment protein J

Chain I:

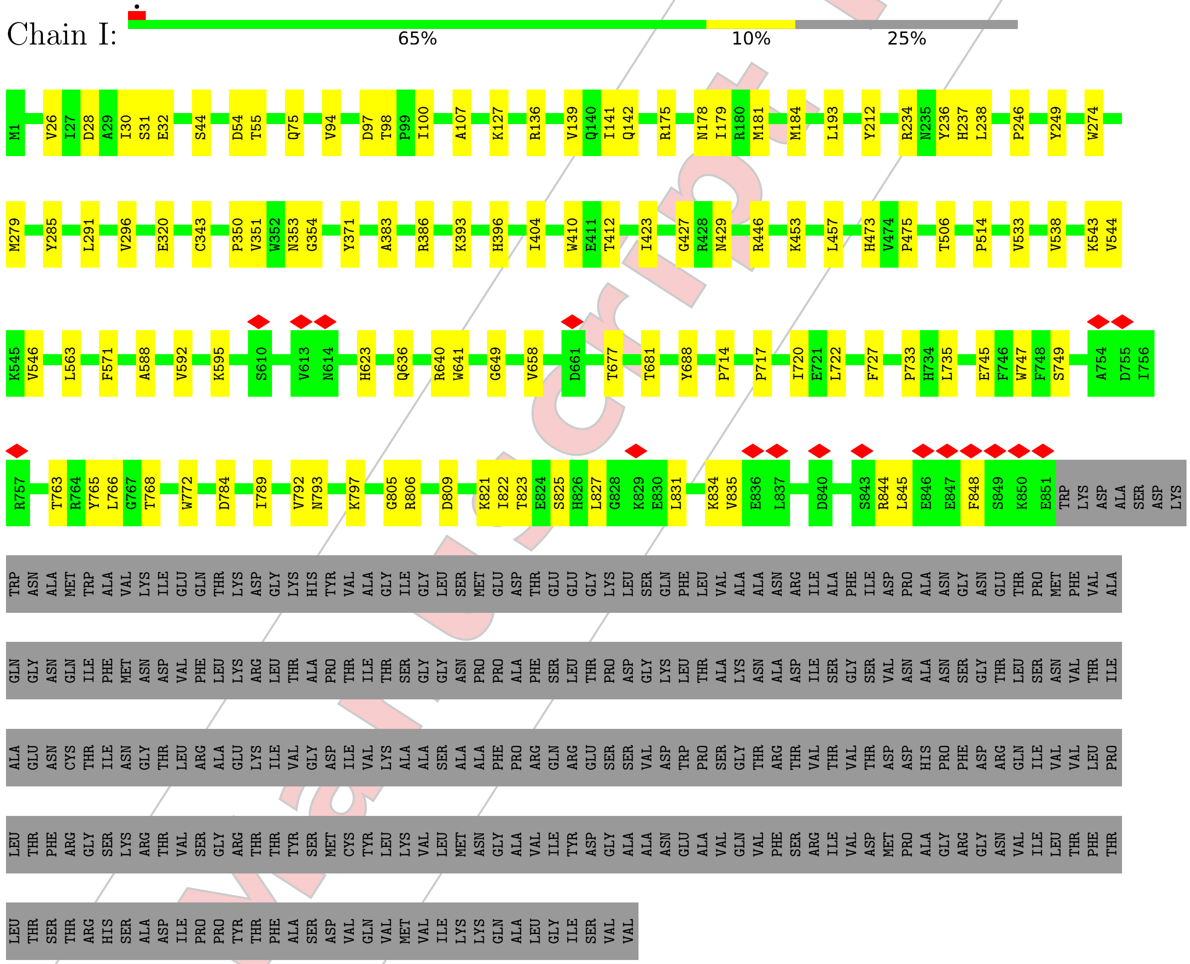

- Molecule 1: Tip attachment protein J

Chain A:

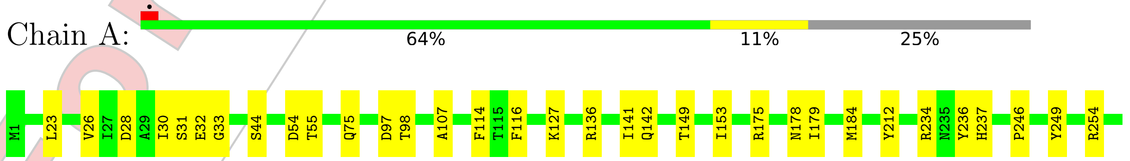

For Manuscript Review

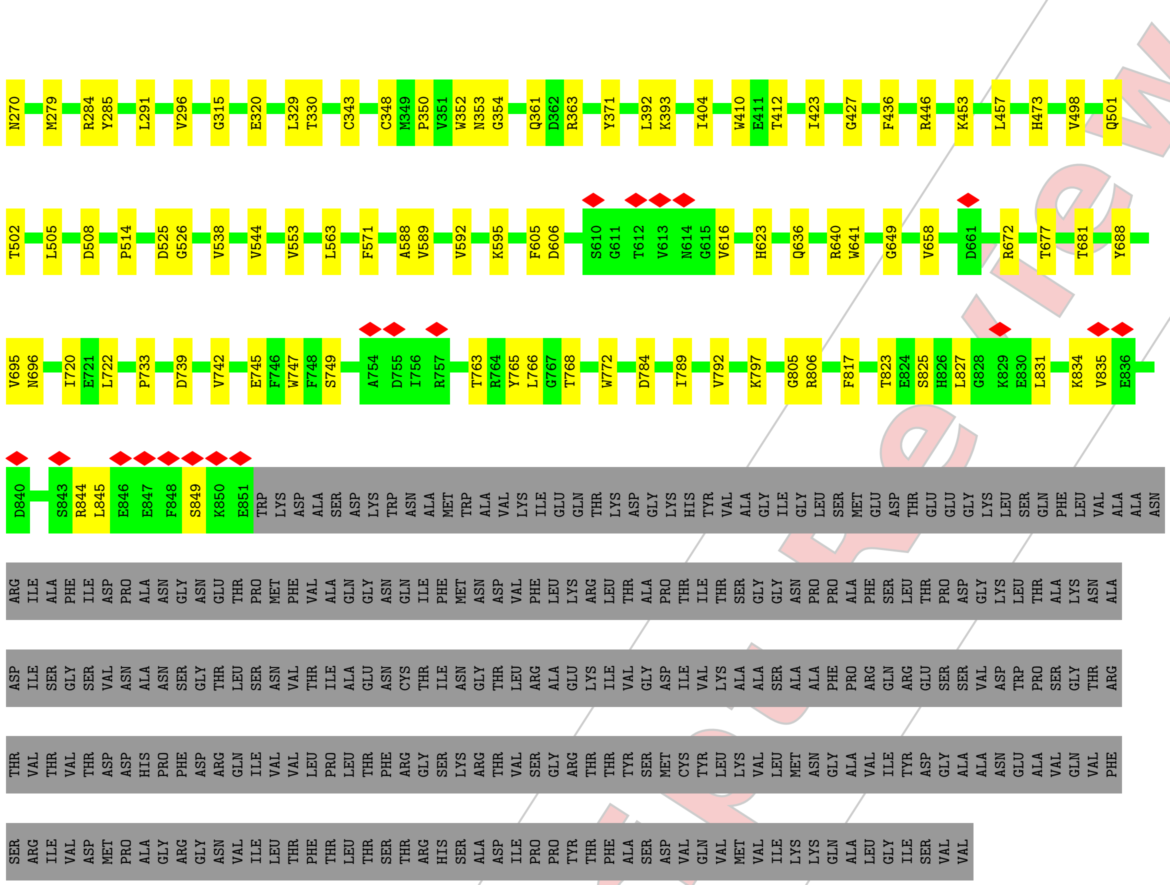

• Molecule 1: Tip attachment protein J

Chain B:

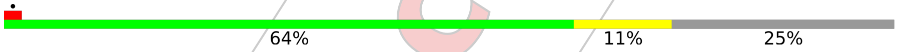

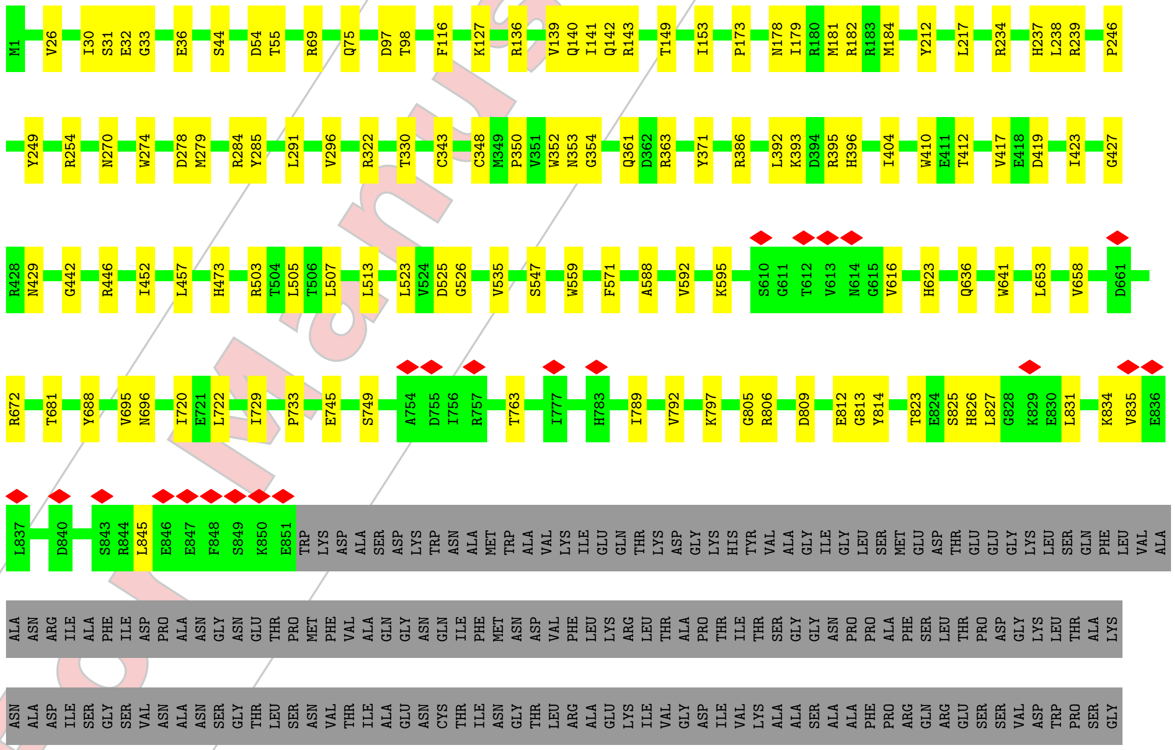

For Manuscript Review

THR ARG THR VAL THR VAL THR ASP ASP HIS PRO PHE ASP ARG GLN ILE VAL VAL LEU PRO LEU THR PHE ARG GLY SER LYS ARG THR VAL SER GLY ARG THR THR TYR SER MET CYS TYR LEU LYS VAL LEU MET ASN GLY ALA VAL ILE TYR ASP GLY ALA ALA ASN GLU ALA VAL GLN

VAL PHE SER ARG ILE VAL ASP MET PRO ALA GLY ARG GLY ASN VAL ILE LEU THR PHE THR LEU THR SER THR ARG HIS SER ALA ASP ILE PRO PRO TYR THR PHE ALA SER ASP VAL GLN VAL MET VAL ILE LYS LYS GLN ALA LEU GLY ILE SER VAL VAL

• Molecule 2: Tail tip protein L

Chain J: 91% 9%

M1 V22 E34 F37 N43 E47 Q52 Y56 R76 S82 M91 L98 V99 K107 D114 E142 D159 F163 P164 G211 C212 R215 Q232

• Molecule 2: Tail tip protein L

Chain G: 91% 9%

M1 E30 E34 F37 E44 W51 Y56 R76 S82 M87 V99 V119 V146 P155 D159 F163 P164 R178 D203 S210 G211 C212 F224 I227 Q232

• Molecule 2: Tail tip protein L

Chain N: 91% 9%

M1 L9 N10 T13 R14 V22 D27 F37 N43 E47 Y56 R76 S82 T102 K107 E142 L143 S144 A145 V146 P155 D159 F163 P164 C212 Q232

• Molecule 3: Tail tip protein M

Chain H: 90% 10%

M1 K2 T3 F4 R5 W6 K7 V8 K9 P10 P18 R52 E63 L72 S94 M95 N109

• Molecule 3: Tail tip protein M

Chain O: 89% 11%

M1 K2 T3 P10 G11 M12 V23 N40 T43 L72 P76 Y77 E78 W79 R80 A87 E105 N109

• Molecule 3: Tail tip protein M

Chain C: 90% 10%

M1 P10 G11 M12 P18 Y29 V46 T47 L48 R52 L58 W68 S94 M95 N109

• Molecule 3: Tail tip protein M

Chain E: 94% 6%

For Manuscript Review

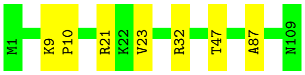

• Molecule 3: Tail tip protein M

Chain D:

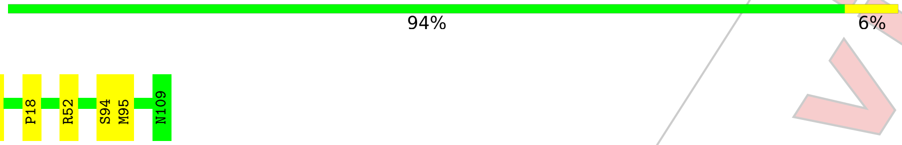

• Molecule 3: Tail tip protein M

Chain F:

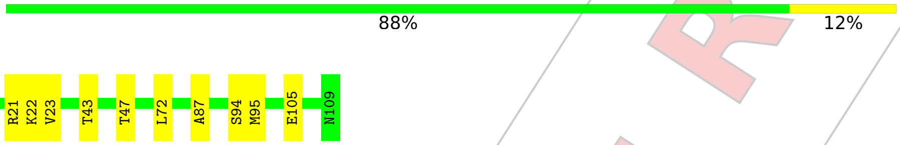

• Molecule 4: Tail tip assembly protein I

Chain x:

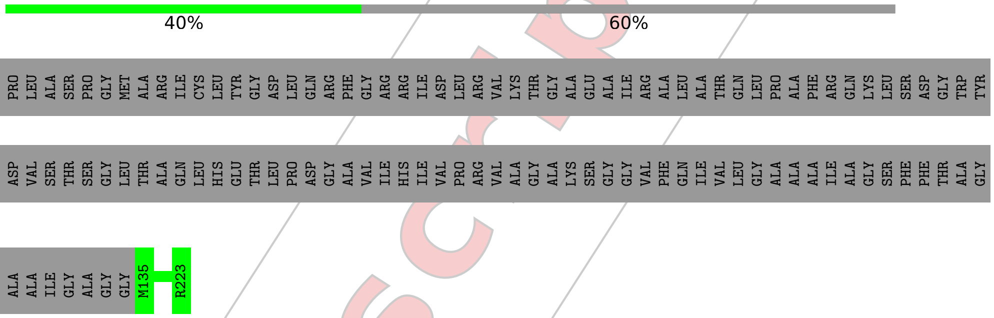

• Molecule 4: Tail tip assembly protein I

Chain K:

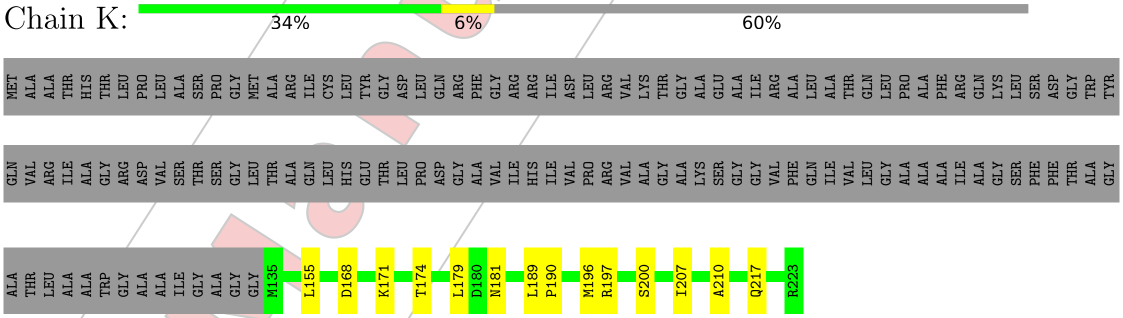

• Molecule 4: Tail tip assembly protein I

Chain Q:

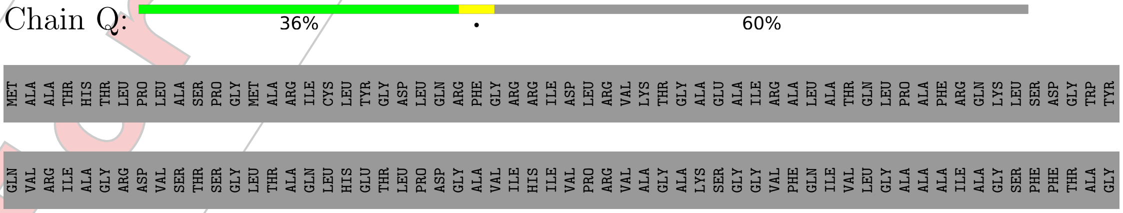

For Manuscript Review

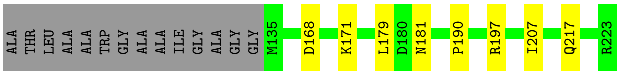

- Molecule 5: Tail tube protein

Chain L: 50% 12% 38%

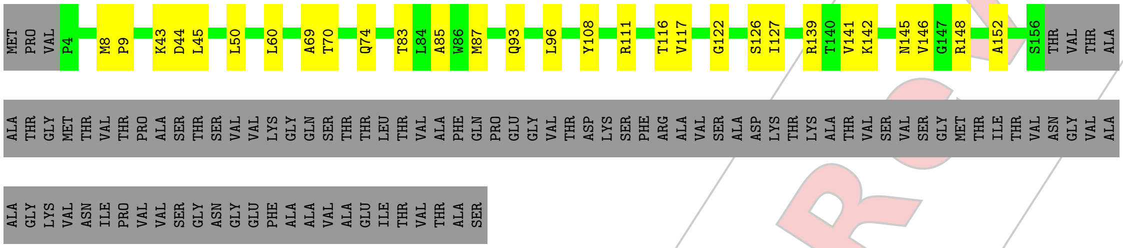

- Molecule 5: Tail tube protein

Chain R: 55% 7% 38%

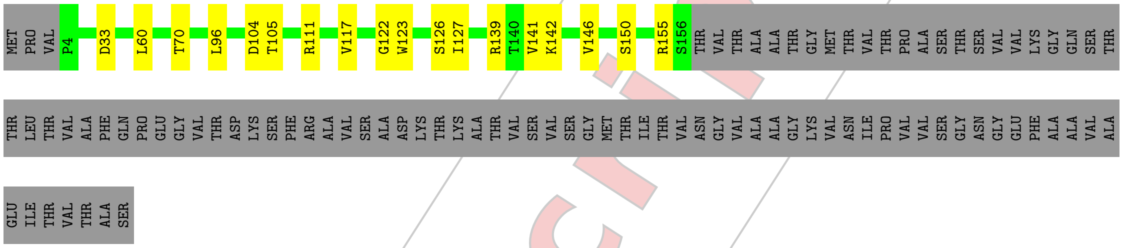

- Molecule 5: Tail tube protein

Chain T: 54% 9% 38%

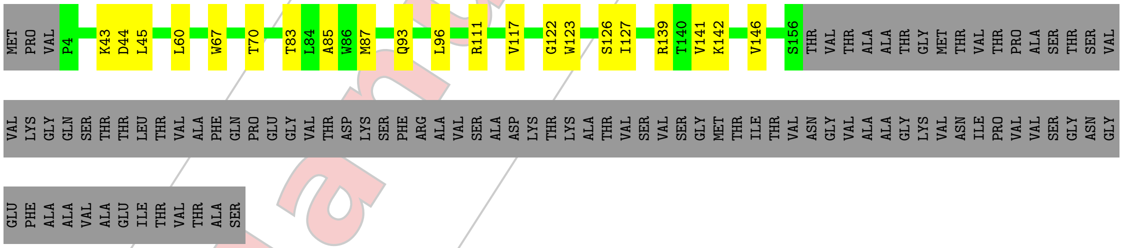

- Molecule 5: Tail tube protein

Chain U: 53% 9% 38%

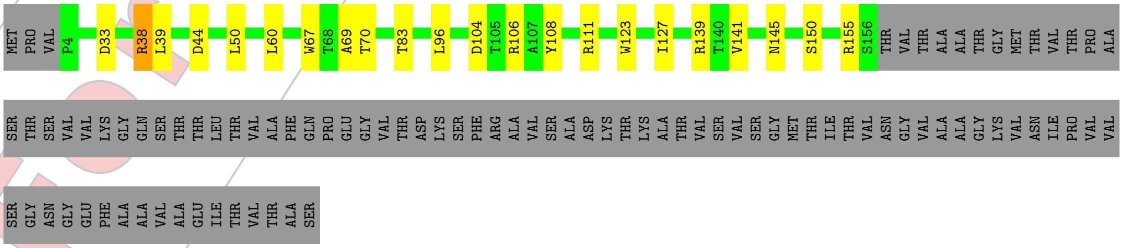

For Manuscript Review

• Molecule 5: Tail tube protein

Chain V: 52% 10% 38%

MET PRO VAL P4 K43 D44 L45 L50 Y55 L60 D61 W67 T70 T83 L84 A85 W86 M87 Q93 L97 N101 Y108 R111 V117 D118 W123 I127 R139 N145 V146 G147 R148 S156 THR VAL THR ALA ALA THR GLY MET THR

VAL THR PRO ALA SER THR SER VAL VAL LYS GLY GLN SER THR THR LEU THR VAL ALA PHE GLN PRO GLU GLY VAL THR ASP LYS SER PHE ARG ALA VAL SER ALA ASP LYS THR LYS ALA THR VAL SER VAL SER GLY MET THR ILE THR VAL ASN GLY VAL ALA ALA GLY LYS VAL ASN

ILE PRO VAL VAL SER GLY ASN GLY GLU PHE ALA ALA VAL ALA GLU ILE THR VAL THR ALA SER

• Molecule 5: Tail tube protein

Chain W: 52% 10% 38%

MET PRO VAL P4 D33 L50 L60 W67 T68 A69 A85 Q93 L96 D104 T105 Y108 R111 V117 G122 W123 S126 I127 G128 K129 R139 T140 V141 K142 N145 V146 S156 THR VAL THR ALA ALA THR GLY MET THR VAL THR PRO ALA

SER THR SER VAL VAL LYS GLY GLN SER THR THR LEU THR VAL ALA PHE GLN PRO GLU GLY VAL THR ASP LYS SER PHE ARG ALA VAL SER ALA ASP LYS THR LYS ALA THR VAL SER VAL SER GLY MET THR ILE THR VAL ASN GLY VAL ALA ALA GLY LYS VAL ASN ILE PRO VAL VAL

SER GLY ASN GLY GLU PHE ALA ALA VAL ALA GLU ILE THR VAL THR ALA SER

• Molecule 6: Tape measure protein

Chain P: • 96%

MET ALA GLU PRO VAL GLY ASP LEU VAL VAL ASP LEU SER LEU ASP ALA ALA ARG PHE ASP GLU GLN MET ALA ARG VAL ARG ARG HIS PHE SER GLY THR GLU SER ASP ALA LYS LYS THR ALA ALA VAL VAL GLU GLN SER LEU SER ARG GLN ALA LEU ALA ALA GLN LYS ALA GLY ILE

SER VAL GLY GLN TYR LYS ALA ALA MET ARG MET LEU PRO ALA GLN PHE THR ASP VAL ALA THR GLN LEU ALA GLY GLY GLN SER PRO TRP LEU ILE LEU LEU GLN GLN GLY GLY GLN VAL LYS ASP SER PHE GLY GLY MET ILE PRO MET PHE ARG GLY LEU ALA GLY ALA ILE THR LEU

PRO MET VAL GLY ALA THR SER LEU ALA VAL ALA THR GLY ALA LEU ALA TYR ALA TRP TYR GLN GLY ASN SER THR LEU SER ASP PHE ASN LYS THR LEU VAL LEU SER GLY ASN GLN ALA GLY LEU THR ALA ASP ARG MET LEU VAL LEU SER ARG ALA GLY GLN ALA ALA GLY LEU THR

PHE ASN GLN THR SER GLU SER LEU SER ALA LEU VAL LYS ALA GLY VAL SER GLY GLU ALA GLN ILE ALA SER ILE SER GLN SER VAL ALA ARG PHE SER SER ALA SER GLY VAL GLU VAL ASP LYS VAL ALA GLU ALA PHE GLY LYS LEU THR THR ASP PRO THR SER GLY LEU THR ALA

MET ALA ARG GLN PHE HIS ASN VAL SER ALA GLU GLN ILE ALA TYR VAL ALA GLN LEU GLN ARG SER GLY ASP GLU ALA GLY ALA LEU GLN ALA ALA ASN GLU ALA ALA THR LYS GLY PHE ASP ASP GLN THR ARG ARG LEU LYS GLU ASN MET GLY THR LEU GLU THR TRP ALA ASP ARG

THR ALA ARG ALA PHE LYS SER MET TRP ASP ALA VAL LEU ASP ILE GLY ARG PRO ASP THR ALA GLN GLU MET LEU ILE LYS ALA GLU ALA ALA TYR LYS LYS ALA ASP ASP ILE TRP ASN LEU ARG LYS ASP ASP TYR PHE VAL ASN ASP GLU ALA ARG ALA ARG TYR TRP ASP ASP ARG

GLU LYS ALA ARG LEU ALA LEU GLU ALA ALA ARG LYS LYS ALA GLU GLN GLN THR GLN GLN ASP LYS ASN ALA GLN GLN GLN SER ASP THR GLU ALA SER ARG LEU LYS TYR THR GLU GLU ALA GLN LYS ALA TYR GLU ARG LEU GLN THR PRO LEU GLU LYS TYR THR ALA ARG GLN GLU

GLU LEU ASN LYS ALA LEU LYS ASP GLY LYS ILE LEU GLN ALA ASP TYR ASN THR LEU MET ALA ALA ALA LYS LYS ASP TYR GLU ALA THR LEU LYS LYS PRO LYS GLN SER SER VAL LYS VAL SER ALA GLY ASP ARG GLN GLU ASP SER ALA HIS ALA ALA LEU LEU THR LEU GLN ALA

GLU LEU ARG THR LEU GLU LYS HIS ALA GLY ALA ASN GLU LYS ILE SER GLN GLN ARG ARG ASP LEU TRP LYS ALA GLU SER GLN PHE ALA VAL LEU GLU GLU ALA ALA GLN ARG ARG GLN LEU SER ALA GLN GLU LYS SER LEU LEU ALA HIS LYS ASP GLU THR LEU GLU TYR LYS ARG

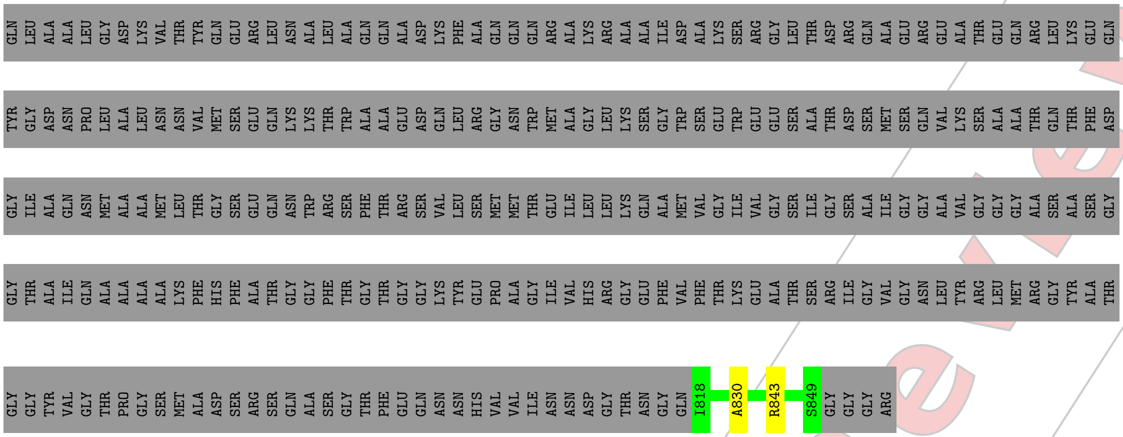

- Molecule 6: Tape measure protein

Chain M:

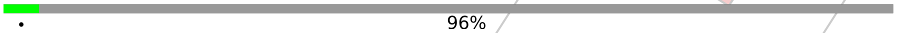

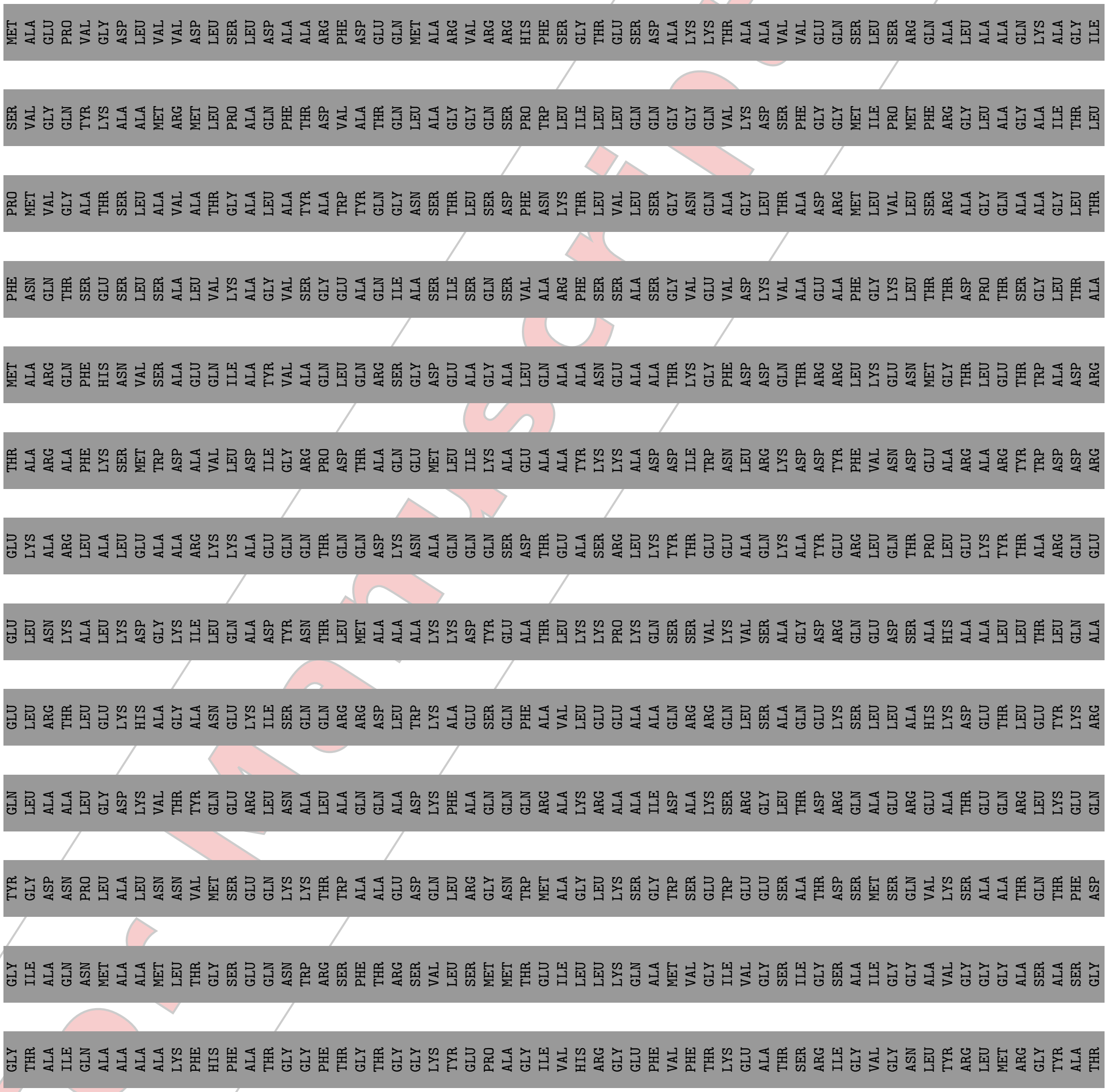

GLY GLY TYR VAL GLY THR PRO GLY SER MET ALA ASP SER ARG SER GLN ALA SER GLY THR PHE GLU GLN ASN ASN HIS VAL VAL ILE ASN ASN ASP GLY THR ASN GLY GLN I818 R843 S849 GLY GLY GLY ARG

• Molecule 6: Tape measure protein

Chain S: 96%

MET ALA GLU PRO VAL GLY ASP LEU VAL VAL ASP LEU SER LEU ASP ALA ALA ARG PHE ASP GLU GLN MET ALA ARG VAL ARG ARG HIS PHE SER GLY THR GLU SER ASP ALA LYS LYS THR ALA ALA VAL VAL GLU GLN SER LEU SER ARG GLN ALA LEU ALA ALA GLN LYS ALA GLY ILE

SER VAL GLY GLN TYR LYS ALA ALA MET ARG MET LEU PRO ALA GLN PHE THR ASP VAL ALA THR GLN LEU ALA GLY GLY GLN SER PRO TRP LEU ILE LEU LEU GLN GLN GLY GLY GLN VAL LYS ASP SER PHE GLY GLY MET ILE PRO MET PHE ARG GLY LEU ALA GLY ALA ILE THR LEU

PRO MET VAL GLY ALA THR SER LEU ALA VAL ALA THR GLY ALA LEU ALA TYR ALA TRP TYR GLN GLY ASN SER THR LEU SER ASP PHE ASN LYS THR LEU VAL LEU SER GLY ASN GLN ALA GLY LEU THR ALA ASP ARG MET LEU VAL LEU SER ARG ALA GLY GLN ALA ALA GLY LEU THR

PHE ASN GLN THR SER GLU SER LEU SER ALA LEU VAL LYS ALA GLY VAL SER GLY GLU ALA GLN ILE ALA SER ILE SER GLN SER VAL ALA ARG PHE SER SER ALA SER GLY VAL GLU VAL ASP LYS VAL ALA GLU ALA PHE GLY LYS LEU THR THR ASP PRO THR SER GLY LEU THR ALA

MET ALA ARG GLN PHE HIS ASN VAL SER ALA GLU GLN ILE ALA TYR VAL ALA GLN LEU GLN ARG SER GLY ASP GLU ALA GLY ALA LEU GLN ALA ALA ASN GLU ALA ALA THR LYS GLY PHE ASP ASP GLN THR ARG ARG LEU LYS GLU ASN MET GLY THR LEU GLU THR TRP ALA ASP ARG

THR ALA ARG ALA PHE LYS SER MET TRP ASP ALA VAL LEU ASP ILE GLY ARG PRO ASP THR ALA GLN GLU MET LEU ILE LYS ALA GLU ALA ALA TYR LYS LYS ALA ASP ASP ILE TRP ASN LEU ARG LYS ASP ASP TYR PHE VAL ASN ASP GLU ALA ARG ALA ARG TYR TRP ASP ASP ARG

GLU LYS ALA ARG LEU ALA LEU GLU ALA ALA ARG LYS LYS ALA GLU GLN GLN THR GLN GLN ASP LYS ASN ALA GLN GLN SER ASP THR GLU ALA SER ARG LEU LYS TYR THR GLU GLU ALA GLN LYS ALA TYR GLU ARG LEU GLN THR PRO LEU GLU LYS TYR THR ALA ARG GLN GLU

GLU LEU ASN LYS ALA LEU LYS ASP GLY LYS ILE LEU GLN ALA ASP TYR ASN THR LEU MET ALA ALA ALA LYS LYS ASP TYR GLU ALA THR LEU LYS LYS PRO LYS GLN SER SER VAL LYS VAL SER ALA GLY ASP ARG GLN GLU ASP SER ALA HIS ALA ALA LEU LEU THR LEU GLN ALA

GLU LEU ARG THR LEU GLU LYS HIS ALA GLY ALA ASN GLU LYS ILE SER GLN GLN ARG ARG ASP LEU TRP LYS ALA GLU SER GLN PHE ALA VAL LEU GLU GLU ALA ALA GLN ARG ARG GLN LEU SER ALA GLN GLU LYS SER LEU LEU ALA HIS LYS ASP GLU THR LEU GLU TYR LYS ARG

GLN LEU ALA ALA LEU GLY ASP LYS VAL THR TYR GLN GLU ARG LEU ASN ALA LEU ALA GLN GLN ALA ASP LYS PHE ALA GLN GLN GLN ARG ALA LYS ARG ALA ALA ILE ASP ALA LYS SER ARG GLY LEU THR ASP ARG GLN ALA GLU ARG GLU ALA THR GLU GLN ARG LEU LYS GLU GLN

TYR GLY ASP ASN PRO LEU ALA LEU ASN ASN VAL MET SER GLU GLN LYS LYS THR TRP ALA ALA GLU ASP GLN LEU ARG GLY ASN TRP MET ALA GLY LEU LYS SER GLY TRP SER GLU TRP GLU GLU SER ALA THR ASP SER MET SER GLN VAL LYS SER ALA ALA THR GLN THR PHE ASP

GLY ILE ALA GLN ASN MET ALA ALA MET LEU THR GLY SER GLU GLN ASN TRP ARG SER PHE THR ARG SER VAL LEU SER MET MET THR GLU ILE LEU LEU LYS GLN ALA MET VAL GLY ILE VAL GLY SER ILE GLY SER ALA ILE GLY GLY ALA VAL GLY GLY GLY ALA SER ALA SER GLY

GLY THR ALA ILE GLN ALA ALA ALA ALA LYS PHE HIS PHE ALA THR GLY GLY PHE THR GLY THR GLY GLY LYS TYR GLU PRO ALA GLY ILE VAL HIS ARG GLY GLU PHE VAL PHE THR LYS GLU ALA THR SER ARG ILE GLY VAL GLY ASN LEU TYR ARG LEU MET ARG GLY TYR ALA THR

GLY GLY TYR VAL GLY THR PRO GLY SER MET ALA ASP SER ARG SER GLN ALA SER GLY THR PHE GLU GLN ASN ASN HIS VAL VAL ILE ASN ASN ASP GLY THR ASN GLY GLN I818 A830 R843 S849 GLY GLY GLY ARG

4 Experimental information (i)

Property	Value	Source
EM reconstruction method	SINGLE PARTICLE	Depositor
Imposed symmetry	POINT, Not provided	
Number of particles used	54385	Depositor
Resolution determination method	FSC 0.143 CUT-OFF	Depositor
CTF correction method	PHASE FLIPPING ONLY	Depositor
Microscope	FEI TITAN KRIOS	Depositor
Voltage (kV)	300	Depositor
Electron dose $(e^-/\text{Å}^2)$	32	Depositor
Minimum defocus (nm)	1600	Depositor
Maximum defocus (nm)	2200	Depositor
Magnification	Not provided	
Image detector	GATAN K3 (6k x 4k)	Depositor
Maximum map value	0.282	Depositor
Minimum map value	-0.126	Depositor
Average map value	0.000	Depositor
Map value standard deviation	0.008	Depositor
Recommended contour level	0.02	Depositor
Map size (Å)	299.2, 299.2, 299.2	wwPDB
Map dimensions	220, 220, 220	wwPDB
Map angles (°)	90.0, 90.0, 90.0	wwPDB
Pixel spacing (Å)	1.36, 1.36, 1.36	Depositor

For Manuscript Review

5 Model quality (i)

5.1 Standard geometry (i)

Bond lengths and bond angles in the following residue types are not validated in this section: SF4

The Z score for a bond length (or angle) is the number of standard deviations the observed value is removed from the expected value. A bond length (or angle) with $|Z| > 5$ is considered an outlier worth inspection. RMSZ is the root-mean-square of all Z scores of the bond lengths (or angles).

Mol	Chain	Bond lengths		Bond angles	
		RMSZ	#|Z| >5	RMSZ	#|Z| >5
1	A	0.28	0/6775	0.51	0/9223
1	B	0.28	0/6775	0.51	0/9223
1	I	0.27	0/6775	0.51	0/9223
2	G	0.32	0/1836	0.52	0/2487
2	J	0.32	0/1836	0.51	0/2487
2	N	0.31	0/1836	0.52	0/2487
3	C	0.28	0/909	0.50	0/1231
3	D	0.28	0/909	0.48	0/1231
3	E	0.28	0/909	0.50	0/1231
3	F	0.28	0/909	0.50	0/1231
3	H	0.28	0/909	0.50	0/1231
3	O	0.28	0/909	0.49	0/1231
4	K	0.29	0/658	0.53	0/888
4	Q	0.28	0/658	0.54	0/888
4	x	0.28	0/658	0.54	0/888
5	L	0.25	0/1203	0.49	0/1639
5	R	0.25	0/1203	0.49	0/1639
5	T	0.32	0/1203	0.51	0/1639
5	U	0.25	0/1203	0.50	0/1639
5	V	0.25	0/1203	0.50	0/1639
5	W	0.25	0/1203	0.49	0/1639
6	M	0.30	0/243	0.48	0/323
6	P	0.30	0/243	0.49	0/323
6	S	0.30	0/243	0.50	0/323
All	All	0.28	0/41208	0.51	0/55983

There are no bond length outliers.

There are no bond angle outliers.

There are no chirality outliers.

There are no planarity outliers.

For Manuscript Review

5.2 Too-close contacts (i)

In the following table, the Non-H and H(model) columns list the number of non-hydrogen atoms and hydrogen atoms in the chain respectively. The H(added) column lists the number of hydrogen atoms added and optimized by MolProbity. The Clashes column lists the number of clashes within the asymmetric unit, whereas Symm-Clashes lists symmetry-related clashes.

Mol	Chain	Non-H	H(model)	H(added)	Clashes	Symm-Clashes
1	A	6633	0	6479	87	0
1	B	6633	0	6479	85	0
1	I	6633	0	6479	72	0
2	G	1801	0	1715	20	0
2	J	1801	0	1715	17	0
2	N	1801	0	1715	16	0
3	C	884	0	881	8	0
3	D	884	0	881	5	0
3	E	884	0	881	6	0
3	F	884	0	881	9	0
3	H	884	0	881	9	0
3	O	884	0	881	8	0
4	K	652	0	661	13	0
4	Q	652	0	661	7	0
4	x	652	0	661	0	0
5	L	1174	0	1122	21	0
5	R	1174	0	1122	12	0
5	T	1174	0	1122	14	0
5	U	1174	0	1122	15	0
5	V	1174	0	1122	22	0
5	W	1174	0	1122	19	0
6	M	241	0	243	1	0
6	P	241	0	243	2	0
6	S	241	0	243	3	0
7	G	8	0	0	1	0
7	J	8	0	0	1	0
7	N	8	0	0	1	0
All	All	40353	0	39312	385	0

The all-atom clashscore is defined as the number of clashes found per 1000 atoms (including hydrogen atoms). The all-atom clashscore for this structure is 5.

All (385) close contacts within the same asymmetric unit are listed below, sorted by their clash magnitude.

For Manuscript Review

Atom-1	Atom-2	Interatomic distance (Å)	Clash overlap (Å)
1:I:54:ASP:OD1	1:I:55:THR:N	2.08	0.86
1:I:745:GLU:HG2	1:I:768:THR:HG22	1.70	0.74
1:A:745:GLU:HG2	1:A:768:THR:HG22	1.70	0.73
2:N:212:CYS:HB2	7:N:301:SF4:S1	2.30	0.71
1:A:834:LYS:HE3	1:B:835:VAL:HA	1.73	0.70
5:R:122:GLY:HA2	5:R:146:VAL:HG12	1.73	0.69
4:Q:168:ASP:HB3	4:Q:171:LYS:HB2	1.75	0.67
1:I:386:ARG:NH1	2:N:142:GLU:OE2	2.28	0.67
5:U:38:ARG:NH1	5:U:39:LEU:O	2.28	0.66
5:U:44:ASP:HB2	5:U:83:THR:HG22	1.76	0.66
1:B:142:GLN:NE2	1:B:178:ASN:OD1	2.28	0.66
1:I:538:VAL:HG12	1:I:544:VAL:HG12	1.76	0.66
1:I:142:GLN:NE2	1:I:178:ASN:OD1	2.28	0.66
5:L:96:LEU:HD21	5:L:141:VAL:HG21	1.78	0.66
3:D:10:PRO:HA	5:T:60:LEU:HD11	1.78	0.65
1:A:44:SER:HB2	1:A:237:HIS:HB3	1.77	0.65
1:A:127:LYS:NZ	1:A:745:GLU:OE2	2.29	0.65
1:I:834:LYS:HE3	1:A:835:VAL:HA	1.77	0.65
1:B:658:VAL:HG22	1:B:688:TYR:HE2	1.62	0.65
1:A:142:GLN:NE2	1:A:178:ASN:OD1	2.29	0.65
1:I:320:GLU:OE2	1:I:446:ARG:NH2	2.30	0.64
5:R:33:ASP:OD1	5:R:111:ARG:NH1	2.29	0.64
1:B:44:SER:HB2	1:B:237:HIS:HB3	1.79	0.64
1:I:127:LYS:NZ	1:I:745:GLU:OE2	2.30	0.64
1:B:136:ARG:HD2	1:B:184:MET:HB2	1.79	0.64
2:J:212:CYS:HB2	7:J:301:SF4:S1	2.38	0.63
5:W:122:GLY:HA2	5:W:146:VAL:HG12	1.80	0.63
3:C:10:PRO:HA	5:L:60:LEU:HD11	1.81	0.62
3:C:68:TRP:O	3:E:32:ARG:NH1	2.32	0.62
1:B:75:GLN:NE2	1:B:212:TYR:O	2.32	0.62
5:L:44:ASP:OD1	5:L:45:LEU:N	2.33	0.62
3:D:3:THR:OG1	3:D:5:ARG:NH1	2.32	0.62
5:W:33:ASP:OD1	5:W:111:ARG:NH1	2.32	0.62
1:I:75:GLN:NE2	1:I:212:TYR:O	2.33	0.61
1:A:733:PRO:HG3	1:A:789:ILE:HD13	1.82	0.61
5:T:44:ASP:OD2	5:T:45:LEU:N	2.33	0.61
1:A:823:THR:HG23	1:A:825:SER:H	1.65	0.61
3:H:7:LYS:HE3	5:V:61:ASP:HB2	1.83	0.61
3:H:10:PRO:HA	5:V:60:LEU:HD11	1.82	0.61
5:L:70:THR:HG23	5:R:123:TRP:HE1	1.66	0.61
1:A:75:GLN:NE2	1:A:212:TYR:O	2.34	0.60
1:A:404:ILE:HG22	1:A:412:THR:HG22	1.82	0.60

Continued on next page...

For Manuscript Review

Continued from previous page...

Atom-1	Atom-2	Interatomic distance (Å)	Clash overlap (Å)
1:A:410:TRP:HB2	4:K:179:LEU:HD11	1.82	0.60
5:T:96:LEU:HD21	5:T:141:VAL:HG21	1.82	0.60
1:I:141:ILE:HG12	1:I:179:ILE:HG22	1.83	0.60
1:I:291:LEU:HD21	1:I:296:VAL:HG23	1.83	0.60
5:U:150:SER:OG	5:U:155:ARG:NH2	2.34	0.60
1:B:141:ILE:HG12	1:B:179:ILE:HG22	1.83	0.60
5:R:127:ILE:HG23	5:R:139:ARG:HD2	1.83	0.60
2:G:37:PHE:HB3	2:G:56:TYR:HD2	1.67	0.60
2:N:22:VAL:HG22	2:N:107:LYS:HG2	1.83	0.60
5:U:127:ILE:HG23	5:U:139:ARG:HD2	1.84	0.60
4:K:168:ASP:HB3	4:K:171:LYS:HB2	1.82	0.60
5:V:70:THR:HG23	5:W:123:TRP:HE1	1.66	0.60
1:A:720:ILE:HG12	1:A:789:ILE:HD12	1.85	0.59
1:B:827:LEU:HD23	1:B:831:LEU:HD23	1.84	0.59
1:I:136:ARG:HD3	1:I:184:MET:HB2	1.82	0.59
1:B:291:LEU:HD21	1:B:296:VAL:HG23	1.85	0.59
5:U:96:LEU:HD21	5:U:141:VAL:HG21	1.85	0.59
1:A:792:VAL:HG23	1:A:797:LYS:HG2	1.86	0.58
1:B:410:TRP:HB2	4:Q:179:LEU:HD11	1.84	0.58
4:K:189:LEU:HD12	4:K:190:PRO:HD2	1.85	0.58
1:B:246:PRO:HG2	1:B:249:TYR:HB2	1.85	0.58
1:A:136:ARG:HD2	1:A:184:MET:HB2	1.85	0.57
1:I:823:THR:HG23	1:I:825:SER:H	1.69	0.57
1:I:827:LEU:HG	1:I:831:LEU:HD23	1.86	0.57
1:B:386:ARG:NH1	2:J:142:GLU:OE2	2.36	0.57
1:I:649:GLY:HA2	4:K:207:ILE:HD12	1.86	0.57
1:B:149:THR:O	4:Q:217:GLN:NE2	2.38	0.57
1:I:792:VAL:HG12	1:I:797:LYS:HG2	1.87	0.57
1:A:361:GLN:OE1	1:A:363:ARG:NH1	2.38	0.57
5:W:127:ILE:HG23	5:W:139:ARG:HD2	1.87	0.57
1:I:371:TYR:OH	1:I:473:HIS:NE2	2.26	0.56
5:W:108:TYR:OH	5:W:145:ASN:OD1	2.23	0.56
3:E:10:PRO:HA	5:W:60:LEU:HD11	1.88	0.56
1:A:107:ALA:O	1:A:175:ARG:NE	2.31	0.56
1:A:636:GLN:HG2	1:A:681:THR:HG22	1.88	0.56
1:B:127:LYS:NZ	1:B:745:GLU:OE2	2.38	0.56
1:A:827:LEU:HG	1:A:831:LEU:HD23	1.87	0.56
1:B:658:VAL:HG22	1:B:688:TYR:CE2	2.41	0.55
1:A:371:TYR:OH	1:A:473:HIS:NE2	2.33	0.55
3:E:9:LYS:HB2	3:E:47:THR:HG23	1.88	0.55
1:A:97:ASP:OD1	1:A:98:THR:N	2.40	0.55

Continued on next page...

For Manuscript Review

Continued from previous page...

Atom-1	Atom-2	Interatomic distance (Å)	Clash overlap (Å)
3:H:52:ARG:NH1	3:H:95:MET:O	2.39	0.55
1:B:330:THR:HA	4:Q:190:PRO:HB3	1.89	0.55
3:O:10:PRO:HA	5:U:60:LEU:HD11	1.88	0.55
1:I:571:PHE:HB3	1:I:588:ALA:HB1	1.89	0.54
1:I:835:VAL:HA	1:B:834:LYS:NZ	2.23	0.54
2:G:212:CYS:HB2	7:G:301:SF4:S1	2.47	0.54
5:V:118:ASP:OD1	5:W:129:LYS:NZ	2.39	0.54
1:B:525:ASP:OD2	1:B:526:GLY:N	2.40	0.54
1:B:792:VAL:HG23	1:B:797:LYS:HG2	1.90	0.54
1:I:727:PHE:HE2	1:B:814:TYR:HD2	1.56	0.54
1:B:54:ASP:OD2	1:B:55:THR:N	2.34	0.54
1:I:30:ILE:HG21	1:I:238:LEU:HD22	1.89	0.54
1:A:291:LEU:HD21	1:A:296:VAL:HG23	1.88	0.54
1:B:722:LEU:HD13	1:B:805:GLY:HA3	1.89	0.54
1:I:640:ARG:HG2	1:I:677:THR:HG22	1.90	0.53
1:B:636:GLN:HG2	1:B:681:THR:HG22	1.91	0.53
5:V:44:ASP:OD2	5:V:45:LEU:N	2.41	0.53
1:I:722:LEU:HD13	1:I:805:GLY:HA3	1.90	0.53
1:B:457:LEU:HD23	1:B:592:VAL:HG11	1.90	0.53
5:R:150:SER:OG	5:R:155:ARG:NH2	2.42	0.53
1:A:141:ILE:HG12	1:A:179:ILE:HG22	1.91	0.53
1:B:279:MET:HG2	2:G:163:PHE:CE1	2.44	0.53
1:B:361:GLN:OE1	1:B:363:ARG:NH1	2.42	0.53
2:G:51:TRP:HB3	2:G:56:TYR:HE1	1.74	0.53
5:U:33:ASP:OD1	5:U:111:ARG:NH1	2.41	0.53
1:I:351:VAL:HG21	1:I:475:PRO:HG2	1.90	0.52
3:D:52:ARG:NH1	3:D:95:MET:O	2.42	0.52
1:I:94:VAL:HG22	1:I:100:ILE:HD12	1.92	0.52
1:I:835:VAL:HA	1:B:834:LYS:HZ2	1.75	0.52
1:A:501:GLN:HG3	1:A:502:THR:HG23	1.92	0.52
1:B:720:ILE:HG12	1:B:789:ILE:HD12	1.92	0.52
5:L:108:TYR:OH	5:L:145:ASN:OD1	2.24	0.52
1:I:44:SER:HB2	1:I:237:HIS:HB3	1.90	0.51
1:B:26:VAL:HG11	1:B:234:ARG:CZ	2.40	0.51
1:B:749:SER:HB3	1:B:763:THR:HG22	1.92	0.51
2:G:37:PHE:HB3	2:G:56:TYR:CD2	2.45	0.51
5:T:85:ALA:O	5:T:93:GLN:NE2	2.43	0.51
1:A:525:ASP:OD1	1:A:526:GLY:N	2.42	0.51
1:B:30:ILE:HG21	1:B:238:LEU:HD22	1.92	0.51
5:T:111:ARG:HG3	5:T:117:VAL:HG12	1.92	0.51
1:I:353:ASN:OD1	1:I:354:GLY:N	2.37	0.51

Continued on next page...

For Manuscript Review

Continued from previous page...

Atom-1	Atom-2	Interatomic distance (Å)	Clash overlap (Å)
1:A:649:GLY:HA2	4:Q:207:ILE:HD12	1.92	0.51
2:J:91:MET:HB3	2:J:98:LEU:HD11	1.91	0.51
1:A:320:GLU:OE2	1:A:446:ARG:NH2	2.43	0.51
2:J:22:VAL:HG22	2:J:107:LYS:HG2	1.93	0.51
1:A:616:VAL:O	1:A:696:ASN:ND2	2.44	0.51
1:I:97:ASP:OD1	1:I:98:THR:N	2.44	0.51
5:V:85:ALA:O	5:V:93:GLN:NE2	2.43	0.51
1:B:823:THR:HG23	1:B:825:SER:H	1.76	0.50
5:T:127:ILE:HG23	5:T:139:ARG:HD2	1.93	0.50
1:B:254:ARG:HH22	1:B:270:ASN:HB2	1.76	0.50
5:U:108:TYR:OH	5:U:145:ASN:OD1	2.24	0.50
1:I:749:SER:HB3	1:I:763:THR:HG22	1.93	0.50
1:I:26:VAL:HG11	1:I:234:ARG:CZ	2.42	0.50
1:A:722:LEU:HD13	1:A:805:GLY:HA3	1.93	0.50
1:B:410:TRP:CD1	4:Q:197:ARG:HD3	2.46	0.50
5:W:96:LEU:HD21	5:W:141:VAL:HG21	1.93	0.50
1:I:720:ILE:HG12	1:I:789:ILE:HD12	1.93	0.50
3:F:10:PRO:HA	5:R:60:LEU:HD11	1.94	0.50
1:I:733:PRO:HG3	1:I:789:ILE:HD13	1.94	0.49
1:I:822:ILE:HD12	1:B:826:HIS:HB3	1.94	0.49
1:B:140:GLN:HE21	1:B:149:THR:HG22	1.77	0.49
5:W:111:ARG:HG3	5:W:117:VAL:HG12	1.94	0.49
5:W:104:ASP:OD1	5:W:105:THR:N	2.44	0.49
5:T:70:THR:HG23	5:U:123:TRP:HE1	1.76	0.49
1:A:623:HIS:O	1:A:641:TRP:HA	2.13	0.49
5:V:148:ARG:HG2	5:V:148:ARG:HH11	1.77	0.49
1:A:26:VAL:HG11	1:A:234:ARG:CZ	2.41	0.49
1:A:330:THR:HA	4:K:190:PRO:HB3	1.93	0.49
1:B:116:PHE:CE2	1:B:153:ILE:HG21	2.47	0.49
2:N:10:ASN:O	2:N:14:ARG:HG3	2.12	0.49
1:A:353:ASN:OD1	1:A:354:GLY:N	2.41	0.49
1:B:97:ASP:OD1	1:B:98:THR:N	2.45	0.49
1:I:506:THR:HG22	1:I:543:LYS:HG2	1.95	0.49
1:I:806:ARG:NH1	1:I:809:ASP:OD2	2.46	0.49
3:O:12:MET:HE1	3:O:76:PRO:HD2	1.95	0.49
5:V:127:ILE:HG23	5:V:139:ARG:HD2	1.93	0.49
2:J:76:ARG:HG3	6:S:843:ARG:HH12	1.78	0.48
5:L:127:ILE:HG23	5:L:139:ARG:HD2	1.93	0.48
5:R:104:ASP:OD1	5:R:105:THR:N	2.45	0.48
1:I:714:PRO:HB3	1:I:793:ASN:ND2	2.28	0.48
5:T:67:TRP:HH2	5:U:50:LEU:HD23	1.78	0.48

Continued on next page...

For Manuscript Review

Continued from previous page...

Atom-1	Atom-2	Interatomic distance (Å)	Clash overlap (Å)
1:A:457:LEU:HD23	1:A:592:VAL:HG11	1.95	0.48
3:O:43:THR:HG22	3:O:105:GLU:HG2	1.95	0.48
1:A:343:CYS:SG	1:A:350:PRO:HG3	2.53	0.48
1:A:410:TRP:CD1	4:K:197:ARG:HD3	2.48	0.48
1:A:508:ASP:OD1	3:C:29:TYR:OH	2.25	0.48
5:U:67:TRP:HH2	5:V:50:LEU:HD23	1.79	0.48
5:V:108:TYR:OH	5:V:145:ASN:OD1	2.22	0.48
1:I:457:LEU:HD23	1:I:592:VAL:HG11	1.96	0.48
5:U:70:THR:HG23	5:V:123:TRP:HE1	1.79	0.48
1:A:739:ASP:O	1:A:742:VAL:HG22	2.14	0.48
1:I:246:PRO:HG2	1:I:249:TYR:HB2	1.95	0.48
3:O:78:GLU:OE2	3:O:80:ARG:NH2	2.43	0.48
3:O:3:THR:HG22	3:O:72:LEU:HB2	1.95	0.48
2:G:82:SER:HB3	3:F:23:VAL:HG11	1.95	0.47
1:A:747:TRP:CH2	1:A:765:TYR:HB2	2.50	0.47
1:B:393:LYS:HD3	2:J:159:ASP:HB2	1.96	0.47
1:A:31:SER:OG	1:A:32:GLU:N	2.46	0.47
5:T:126:SER:HB3	5:T:142:LYS:HB3	1.96	0.47
1:A:28:ASP:OD2	1:A:236:TYR:OH	2.26	0.47
1:A:749:SER:HB3	1:A:763:THR:HG22	1.95	0.47
1:B:371:TYR:OH	1:B:473:HIS:NE2	2.34	0.47
1:B:623:HIS:O	1:B:641:TRP:HA	2.14	0.47
1:A:844:ARG:HH21	1:B:845:LEU:HD22	1.80	0.47
2:N:146:VAL:HG22	3:E:21:ARG:HD2	1.95	0.47
3:H:94:SER:OG	3:H:95:MET:N	2.48	0.47
1:I:107:ALA:O	1:I:175:ARG:NE	2.37	0.47
1:A:33:GLY:HA2	1:A:284:ARG:HH21	1.80	0.47
3:H:3:THR:OG1	3:H:5:ARG:NH1	2.47	0.47
5:V:97:LEU:O	5:V:101:ASN:ND2	2.32	0.47
1:I:279:MET:HG2	2:J:163:PHE:CE1	2.50	0.47
1:I:139:VAL:HG22	1:I:181:MET:HG3	1.96	0.47
1:I:514:PRO:HG3	1:I:563:LEU:HD22	1.97	0.47
1:I:623:HIS:O	1:I:641:TRP:HA	2.13	0.47
1:A:234:ARG:NH2	4:K:181:ASN:HD22	2.13	0.47
1:A:640:ARG:HG2	1:A:677:THR:HG22	1.96	0.47
3:C:12:MET:HG3	3:C:46:VAL:HG12	1.96	0.47
1:B:396:HIS:HA	1:B:429:ASN:HB2	1.97	0.47
1:A:498:VAL:HG12	1:A:505:LEU:HD22	1.96	0.46
1:B:353:ASN:OD1	1:B:354:GLY:N	2.43	0.46
1:B:182:ARG:HE	1:B:184:MET:HE3	1.80	0.46
1:A:116:PHE:CZ	1:A:153:ILE:HD13	2.50	0.46

Continued on next page...

For Manuscript Review

Continued from previous page...

Atom-1	Atom-2	Interatomic distance (Å)	Clash overlap (Å)
2:N:76:ARG:HG3	6:P:843:ARG:HH12	1.80	0.46
5:L:8:MET:HG2	5:L:9:PRO:HD2	1.96	0.46
1:A:498:VAL:HG11	1:A:553:VAL:HG23	1.98	0.46
1:A:571:PHE:HB3	1:A:588:ALA:HB1	1.98	0.46
1:B:722:LEU:HD23	1:B:722:LEU:HA	1.80	0.46
1:I:31:SER:OG	1:I:32:GLU:N	2.49	0.46
1:I:343:CYS:SG	1:I:350:PRO:HG3	2.55	0.46
5:L:146:VAL:HG23	5:W:69:ALA:HA	1.97	0.46
5:L:50:LEU:HD23	5:W:67:TRP:HH2	1.81	0.46
1:A:54:ASP:OD2	1:A:55:THR:N	2.41	0.46
2:N:82:SER:HB3	3:E:23:VAL:HG11	1.97	0.46
3:F:22:LYS:HB2	3:F:22:LYS:HE3	1.64	0.46
5:L:126:SER:HB3	5:L:142:LYS:HB3	1.98	0.46
1:B:31:SER:OG	1:B:32:GLU:N	2.48	0.46
2:J:211:GLY:O	2:J:215:ARG:NH1	2.39	0.46
1:I:844:ARG:HH21	1:A:845:LEU:HD22	1.81	0.45
5:V:87:MET:H	5:V:93:GLN:HE22	1.63	0.45
1:I:821:LYS:N	1:I:821:LYS:HD3	2.32	0.45
1:A:23:LEU:HB2	4:K:210:ALA:HB2	1.99	0.45
1:B:234:ARG:NH2	4:Q:181:ASN:HD22	2.14	0.45
1:A:279:MET:HG2	2:N:163:PHE:CE1	2.52	0.45
2:G:34:GLU:N	2:G:34:GLU:OE2	2.49	0.45
3:C:48:LEU:HD11	3:C:58:LEU:HD11	1.99	0.45
1:B:672:ARG:HH22	1:B:695:VAL:HG21	1.82	0.45
2:J:34:GLU:N	2:J:34:GLU:OE2	2.50	0.45
2:J:52:GLN:N	2:J:114:ASP:OD2	2.50	0.45
5:U:104:ASP:OD2	5:U:106:ARG:NH1	2.49	0.45
1:A:423:ILE:HA	1:A:427:GLY:O	2.17	0.45
1:I:246:PRO:HD3	1:I:274:TRP:CE2	2.53	0.44
1:B:404:ILE:HG22	1:B:412:THR:HG22	1.99	0.44
3:F:3:THR:HG22	3:F:72:LEU:HB2	1.98	0.44
5:T:43:LYS:HB2	5:T:83:THR:HG23	1.99	0.44
1:B:352:TRP:CZ2	2:G:155:PRO:HD2	2.53	0.44
1:B:513:LEU:HD21	1:B:535:VAL:HG11	2.00	0.44
1:A:831:LEU:HD21	1:B:827:LEU:HD21	1.99	0.44
1:B:653:LEU:HD13	1:B:672:ARG:HH11	1.82	0.44
3:H:63:GLU:HG3	3:O:40:ASN:HD22	1.83	0.44
1:A:291:LEU:HD21	1:A:296:VAL:CG2	2.47	0.44
3:O:87:ALA:O	3:D:18:PRO:HD2	2.16	0.44
3:F:9:LYS:HB2	3:F:47:THR:HG23	2.00	0.44
1:A:246:PRO:HG2	1:A:249:TYR:HB2	1.99	0.44

Continued on next page...

For Manuscript Review

Continued from previous page...

Atom-1	Atom-2	Interatomic distance (Å)	Clash overlap (Å)
1:B:417:VAL:HG12	1:B:452:ILE:HD12	2.00	0.44
1:B:507:LEU:HD22	1:B:559:TRP:CE2	2.52	0.44
2:G:44:GLU:OE1	2:G:119:VAL:HG12	2.18	0.44
1:I:658:VAL:HG22	1:I:688:TYR:CE1	2.52	0.44
1:I:848:PHE:HZ	1:A:849:SER:HG	1.66	0.44
3:D:94:SER:OG	3:D:95:MET:N	2.51	0.44
1:I:32:GLU:OE2	1:I:285:TYR:HB3	2.18	0.43
1:B:343:CYS:SG	1:B:350:PRO:HG3	2.57	0.43
1:I:28:ASP:OD2	1:I:236:TYR:OH	2.28	0.43
1:A:393:LYS:HD3	2:G:159:ASP:HB2	2.00	0.43
1:A:784:ASP:OD1	1:A:806:ARG:HD3	2.17	0.43
5:V:55:TYR:O	5:V:70:THR:OG1	2.24	0.43
1:I:291:LEU:HD21	1:I:296:VAL:CG2	2.47	0.43
1:I:396:HIS:HA	1:I:429:ASN:HB2	2.00	0.43
1:B:392:LEU:HD13	2:J:99:VAL:HG12	1.99	0.43
1:B:423:ILE:HA	1:B:427:GLY:O	2.18	0.43
1:B:733:PRO:HG3	1:B:789:ILE:HD13	1.99	0.43
5:L:122:GLY:HA2	5:L:146:VAL:HG12	2.00	0.43
5:R:111:ARG:HG3	5:R:117:VAL:HG12	2.01	0.43
1:B:36:GLU:OE2	1:B:239:ARG:NE	2.46	0.43
1:I:636:GLN:HG2	1:I:681:THR:HG22	2.00	0.43
1:I:845:LEU:HB2	1:A:845:LEU:HD11	1.99	0.43
1:B:33:GLY:HA2	1:B:284:ARG:HH21	1.83	0.43
1:B:291:LEU:HD21	1:B:296:VAL:CG2	2.47	0.43
1:B:806:ARG:NH1	1:B:809:ASP:OD2	2.52	0.43
3:H:9:LYS:HG3	3:H:10:PRO:HD2	2.01	0.43
5:V:148:ARG:HG2	5:V:148:ARG:NH1	2.34	0.43
5:W:85:ALA:O	5:W:93:GLN:NE2	2.46	0.43
1:I:533:VAL:HG11	1:I:546:VAL:HB	2.01	0.43
1:B:616:VAL:O	1:B:696:ASN:ND2	2.52	0.43
2:N:43:ASN:HD21	2:N:47:GLU:HB2	1.84	0.43
3:H:18:PRO:HD2	3:E:87:ALA:O	2.18	0.43
1:A:32:GLU:OE2	1:A:285:TYR:HB3	2.18	0.43
1:B:395:ARG:O	1:B:429:ASN:ND2	2.37	0.43
2:J:76:ARG:HG3	6:S:843:ARG:NH1	2.34	0.43
5:R:126:SER:HB3	5:R:142:LYS:HB3	1.99	0.43
2:J:163:PHE:HB3	2:J:164:PRO:HD3	2.01	0.43
1:A:392:LEU:HD13	2:G:99:VAL:HG12	2.01	0.43
5:L:74:GLN:HG2	5:W:68:THR:HG22	2.01	0.42
1:A:315:GLY:HA3	1:A:605:PHE:CD2	2.55	0.42
1:A:766:LEU:HD23	1:A:772:TRP:NE1	2.34	0.42

Continued on next page...

For Manuscript Review

Continued from previous page...

Atom-1	Atom-2	Interatomic distance (Å)	Clash overlap (Å)
1:B:457:LEU:HD22	1:B:595:LYS:HB3	2.02	0.42
2:J:43:ASN:HD21	2:J:47:GLU:HB2	1.84	0.42
5:L:44:ASP:HB3	5:L:83:THR:HG22	2.00	0.42
5:T:44:ASP:HB3	5:T:83:THR:HG22	2.00	0.42
1:I:193:LEU:HD12	1:I:193:LEU:HA	1.83	0.42
1:I:383:ALA:HB1	2:N:144:SER:HB2	2.02	0.42
1:I:453:LYS:HA	1:I:453:LYS:HD3	1.82	0.42
1:I:717:PRO:HA	1:I:735:LEU:HD23	2.00	0.42
1:A:30:ILE:HA	4:K:200:SER:OG	2.19	0.42
2:G:178:ARG:NH2	2:G:203:ASP:OD2	2.42	0.42
5:V:67:TRP:HH2	5:W:50:LEU:HD23	1.84	0.42
1:B:116:PHE:CZ	1:B:153:ILE:HD13	2.54	0.42
1:B:246:PRO:HD3	1:B:274:TRP:CE2	2.55	0.42
5:L:87:MET:H	5:L:93:GLN:NE2	2.17	0.42
5:R:96:LEU:HD21	5:R:141:VAL:HG21	2.01	0.42
5:T:87:MET:H	5:T:93:GLN:NE2	2.17	0.42
1:I:766:LEU:HD23	1:I:772:TRP:NE1	2.34	0.42
1:A:658:VAL:HG22	1:A:688:TYR:CE1	2.54	0.42
5:L:69:ALA:HA	5:R:146:VAL:HG23	2.02	0.42
1:I:722:LEU:HD23	1:I:722:LEU:HA	1.90	0.42
1:A:116:PHE:CZ	1:A:153:ILE:HG21	2.55	0.42
1:A:606:ASP:OD2	2:G:210:SER:OG	2.27	0.42
1:B:503:ARG:NH2	1:B:547:SER:O	2.49	0.42
3:C:94:SER:OG	3:C:95:MET:N	2.52	0.42
1:B:571:PHE:HB3	1:B:588:ALA:HB1	2.02	0.42
2:N:27:ASP:HB3	2:N:102:THR:HB	2.00	0.42
5:V:87:MET:H	5:V:93:GLN:NE2	2.17	0.42
1:I:404:ILE:HG22	1:I:412:THR:HG22	2.02	0.42
1:B:831:LEU:HD12	1:B:831:LEU:HA	1.85	0.42
2:G:224:PHE:HB2	2:G:227:ILE:HD11	2.01	0.42
5:V:43:LYS:HB2	5:V:83:THR:HG23	2.00	0.42
1:I:747:TRP:CH2	1:I:765:TYR:HB2	2.55	0.42
1:A:392:LEU:HB2	2:G:99:VAL:CG1	2.50	0.42
1:A:453:LYS:HD3	1:A:453:LYS:HA	1.78	0.42
1:A:672:ARG:HH22	1:A:695:VAL:HG21	1.84	0.42
1:B:442:GLY:O	1:B:446:ARG:HG3	2.20	0.42
2:J:82:SER:HB3	3:O:23:VAL:HG11	2.02	0.42
1:A:352:TRP:CZ2	2:N:155:PRO:HD2	2.54	0.42
1:A:404:ILE:HD11	4:K:196:MET:CE	2.50	0.42
1:B:343:CYS:HB3	1:B:348:CYS:O	2.20	0.42
2:N:37:PHE:HB3	2:N:56:TYR:CD2	2.55	0.42

Continued on next page...

For Manuscript Review

Continued from previous page...

Atom-1	Atom-2	Interatomic distance (Å)	Clash overlap (Å)
1:B:278:ASP:OD2	1:B:285:TYR:OH	2.38	0.41
5:L:116:THR:HB	5:L:152:ALA:HB1	2.02	0.41
1:I:423:ILE:HA	1:I:427:GLY:O	2.20	0.41
2:N:163:PHE:HB3	2:N:164:PRO:HD3	2.03	0.41
1:B:69:ARG:HB2	1:B:217:LEU:HD23	2.02	0.41
1:B:419:ASP:O	1:B:423:ILE:HG13	2.21	0.41
2:G:87:MET:HE2	2:G:87:MET:HB3	1.99	0.41
5:L:85:ALA:O	5:L:93:GLN:NE2	2.52	0.41
5:V:111:ARG:HG3	5:V:117:VAL:HG12	2.02	0.41
1:A:514:PRO:HG3	1:A:563:LEU:HD22	2.02	0.41
1:B:139:VAL:HG22	1:B:181:MET:HG3	2.01	0.41
3:H:3:THR:HG22	3:H:72:LEU:HB2	2.03	0.41
5:L:148:ARG:NH1	5:W:67:TRP:HE1	2.19	0.41
1:A:149:THR:O	4:K:217:GLN:NE2	2.54	0.41
1:A:412:THR:HG21	4:K:174:THR:HG23	2.02	0.41
2:N:9:LEU:O	2:N:13:THR:HG23	2.21	0.41
1:I:784:ASP:OD1	1:I:806:ARG:HD3	2.21	0.41
1:A:817:PHE:HE2	1:B:814:TYR:HD1	1.68	0.41
1:B:812:GLU:HG2	1:B:813:GLY:N	2.35	0.41
5:R:70:THR:HG23	5:T:123:TRP:HE1	1.85	0.41
5:U:67:TRP:CH2	5:V:50:LEU:HD23	2.56	0.41
5:U:69:ALA:HA	5:V:146:VAL:HG23	2.02	0.41
1:A:457:LEU:HD22	1:A:595:LYS:HB3	2.02	0.41
3:F:94:SER:OG	3:F:95:MET:N	2.54	0.41
5:L:50:LEU:HD23	5:W:67:TRP:CH2	2.56	0.41
1:I:404:ILE:HD12	1:I:410:TRP:HE3	1.86	0.41
1:I:457:LEU:HD22	1:I:595:LYS:HB3	2.03	0.41
1:A:254:ARG:HH22	1:A:270:ASN:HB2	1.86	0.41
1:A:538:VAL:HG22	1:A:544:VAL:HG12	2.02	0.41
1:A:834:LYS:NZ	1:B:834:LYS:HG2	2.35	0.41
1:B:143:ARG:HG2	1:B:173:PRO:HG3	2.02	0.41
2:G:163:PHE:HB3	2:G:164:PRO:HD3	2.03	0.41
3:C:52:ARG:NH1	3:C:95:MET:O	2.54	0.41
5:W:126:SER:HB3	5:W:142:LYS:HB3	2.02	0.41
1:A:33:GLY:HA2	1:A:284:ARG:NH2	2.34	0.41
1:A:114:PHE:CE2	1:A:179:ILE:HD12	2.56	0.41
2:J:37:PHE:HB3	2:J:56:TYR:CD2	2.56	0.41
2:G:30:GLU:OE1	2:G:30:GLU:HA	2.21	0.41
1:A:329:LEU:HD21	1:A:436:PHE:HZ	1.86	0.40
1:B:322:ARG:HA	1:B:446:ARG:HH21	1.86	0.40
1:B:729:ILE:HD12	1:B:729:ILE:HA	1.92	0.40

Continued on next page...

For Manuscript Review

Continued from previous page...

Atom-1	Atom-2	Interatomic distance (Å)	Clash overlap (Å)
2:J:91:MET:CB	2:J:98:LEU:HD11	2.51	0.40
5:L:43:LYS:HB2	5:L:83:THR:HG23	2.02	0.40
1:I:393:LYS:HD3	2:N:159:ASP:HB2	2.03	0.40
1:B:505:LEU:HD11	1:B:523:LEU:HD11	2.03	0.40
2:G:76:ARG:HG3	6:M:843:ARG:NH1	2.36	0.40
3:C:18:PRO:HD2	3:F:87:ALA:O	2.22	0.40
5:T:122:GLY:HA2	5:T:146:VAL:HG12	2.03	0.40
5:V:70:THR:H	5:W:146:VAL:HG23	1.86	0.40
6:P:830:ALA:HB1	6:S:830:ALA:HA	2.03	0.40
1:I:831:LEU:HD12	1:I:831:LEU:HA	1.87	0.40
1:A:589:VAL:HG11	4:K:155:LEU:HD21	2.03	0.40
2:G:146:VAL:HG22	3:F:21:ARG:HD2	2.03	0.40
1:A:343:CYS:HB3	1:A:348:CYS:O	2.22	0.40
3:F:43:THR:HG22	3:F:105:GLU:HG2	2.03	0.40
5:L:111:ARG:HG3	5:L:117:VAL:HG12	2.02	0.40

There are no symmetry-related clashes.

5.3 Torsion angles (i)

5.3.1 Protein backbone (i)

In the following table, the Percentiles column shows the percent Ramachandran outliers of the chain as a percentile score with respect to all PDB entries followed by that with respect to all EM entries.

The Analysed column shows the number of residues for which the backbone conformation was analysed, and the total number of residues.

Mol	Chain	Analysed	Favoured	Allowed	Outliers	Percentiles	
1	A	849/1132 (75%)	822 (97%)	27 (3%)	0	100	100
1	B	849/1132 (75%)	821 (97%)	28 (3%)	0	100	100
1	I	849/1132 (75%)	822 (97%)	27 (3%)	0	100	100
2	G	230/232 (99%)	223 (97%)	7 (3%)	0	100	100
2	J	230/232 (99%)	223 (97%)	7 (3%)	0	100	100
2	N	230/232 (99%)	222 (96%)	8 (4%)	0	100	100
3	C	107/109 (98%)	105 (98%)	2 (2%)	0	100	100
3	D	107/109 (98%)	104 (97%)	3 (3%)	0	100	100

Continued on next page...

For Manuscript Review

Continued from previous page...

Mol	Chain	Analysed	Favoured	Allowed	Outliers	Percentiles	
3	E	107/109 (98%)	106 (99%)	1 (1%)	0	100	100
3	F	107/109 (98%)	107 (100%)	0	0	100	100
3	H	107/109 (98%)	105 (98%)	2 (2%)	0	100	100
3	O	107/109 (98%)	107 (100%)	0	0	100	100
4	K	87/223 (39%)	86 (99%)	1 (1%)	0	100	100
4	Q	87/223 (39%)	86 (99%)	1 (1%)	0	100	100
4	x	87/223 (39%)	87 (100%)	0	0	100	100
5	L	151/246 (61%)	146 (97%)	5 (3%)	0	100	100
5	R	151/246 (61%)	148 (98%)	3 (2%)	0	100	100
5	T	151/246 (61%)	146 (97%)	5 (3%)	0	100	100
5	U	151/246 (61%)	148 (98%)	3 (2%)	0	100	100
5	V	151/246 (61%)	147 (97%)	4 (3%)	0	100	100
5	W	151/246 (61%)	147 (97%)	4 (3%)	0	100	100
6	M	30/853 (4%)	30 (100%)	0	0	100	100
6	P	30/853 (4%)	30 (100%)	0	0	100	100
6	S	30/853 (4%)	30 (100%)	0	0	100	100
All	All	5136/9450 (54%)	4998 (97%)	138 (3%)	0	100	100

There are no Ramachandran outliers to report.

5.3.2 Protein sidechains (i)

In the following table, the Percentiles column shows the percent sidechain outliers of the chain as a percentile score with respect to all PDB entries followed by that with respect to all EM entries.

The Analysed column shows the number of residues for which the sidechain conformation was analysed, and the total number of residues.

Mol	Chain	Analysed	Rotameric	Outliers	Percentiles	
1	A	726/958 (76%)	726 (100%)	0	100	100
1	B	726/958 (76%)	726 (100%)	0	100	100
1	I	726/958 (76%)	726 (100%)	0	100	100
2	G	198/198 (100%)	198 (100%)	0	100	100
2	J	198/198 (100%)	198 (100%)	0	100	100

Continued on next page...

For Manuscript Review

Continued from previous page...

Mol	Chain	Analysed	Rotameric	Outliers	Percentiles	
2	N	198/198 (100%)	198 (100%)	0	100	100
3	C	96/96 (100%)	96 (100%)	0	100	100
3	D	96/96 (100%)	96 (100%)	0	100	100
3	E	96/96 (100%)	96 (100%)	0	100	100
3	F	96/96 (100%)	96 (100%)	0	100	100
3	H	96/96 (100%)	96 (100%)	0	100	100
3	O	96/96 (100%)	96 (100%)	0	100	100
4	K	71/162 (44%)	71 (100%)	0	100	100
4	Q	71/162 (44%)	71 (100%)	0	100	100
4	x	71/162 (44%)	71 (100%)	0	100	100
5	L	124/196 (63%)	124 (100%)	0	100	100
5	R	124/196 (63%)	124 (100%)	0	100	100
5	T	124/196 (63%)	124 (100%)	0	100	100
5	U	124/196 (63%)	123 (99%)	1 (1%)	81	92
5	V	124/196 (63%)	124 (100%)	0	100	100
5	W	124/196 (63%)	124 (100%)	0	100	100
6	M	23/648 (4%)	23 (100%)	0	100	100
6	P	23/648 (4%)	23 (100%)	0	100	100
6	S	23/648 (4%)	23 (100%)	0	100	100
All	All	4374/7650 (57%)	4373 (100%)	1 (0%)	100	100

All (1) residues with a non-rotameric sidechain are listed below:

Mol	Chain	Res	Type
5	U	38	ARG

Sometimes sidechains can be flipped to improve hydrogen bonding and reduce clashes. There are no such sidechains identified.

5.3.3 RNA ⓘ

There are no RNA molecules in this entry.

For Manuscript Review

5.4 Non-standard residues in protein, DNA, RNA chains (i)

There are no non-standard protein/DNA/RNA residues in this entry.

5.5 Carbohydrates (i)

There are no monosaccharides in this entry.

5.6 Ligand geometry (i)

3 ligands are modelled in this entry.

In the following table, the Counts columns list the number of bonds (or angles) for which Mogul statistics could be retrieved, the number of bonds (or angles) that are observed in the model and the number of bonds (or angles) that are defined in the Chemical Component Dictionary. The Link column lists molecule types, if any, to which the group is linked. The Z score for a bond length (or angle) is the number of standard deviations the observed value is removed from the expected value. A bond length (or angle) with $|Z| > 2$ is considered an outlier worth inspection. RMSZ is the root-mean-square of all Z scores of the bond lengths (or angles).

Mol	Type	Chain	Res	Link	Bond lengths			Bond angles		
					Counts	RMSZ	$\#\|Z\| > 2$	Counts	RMSZ	$\#\|Z\| > 2$
7	SF4	N	301	2	0,12,12	-	-	-		
7	SF4	J	301	2	0,12,12	-	-	-		
7	SF4	G	301	2	0,12,12	-	-	-		

In the following table, the Chirals column lists the number of chiral outliers, the number of chiral centers analysed, the number of these observed in the model and the number defined in the Chemical Component Dictionary. Similar counts are reported in the Torsion and Rings columns. '-' means no outliers of that kind were identified.

Mol	Type	Chain	Res	Link	Chirals	Torsions	Rings
7	SF4	N	301	2	-	-	0/6/5/5
7	SF4	J	301	2	-	-	0/6/5/5
7	SF4	G	301	2	-	-	0/6/5/5

There are no bond length outliers.

There are no bond angle outliers.

There are no chirality outliers.

There are no torsion outliers.

There are no ring outliers.

3 monomers are involved in 3 short contacts:

For Manuscript Review

Mol	Chain	Res	Type	Clashes	Symm-Clashes
7	N	301	SF4	1	0
7	J	301	SF4	1	0
7	G	301	SF4	1	0

5.7 Other polymers ⓘ

There are no such residues in this entry.

5.8 Polymer linkage issues ⓘ

There are no chain breaks in this entry.

For Manuscript Review

6 Map visualisation ⓘ

This section contains visualisations of the EMDB entry EMD-36844. These allow visual inspection of the internal detail of the map and identification of artifacts.

Images derived from a raw map, generated by summing the deposited half-maps, are presented below the corresponding image components of the primary map to allow further visual inspection and comparison with those of the primary map.

6.1 Orthogonal projections ⓘ

6.1.1 Primary map

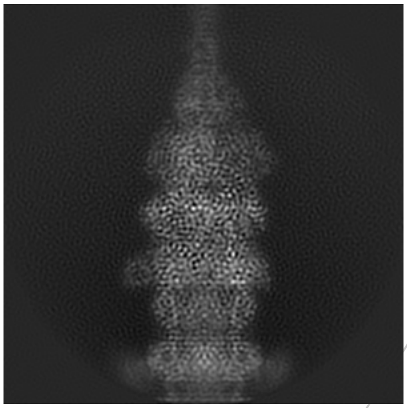
X

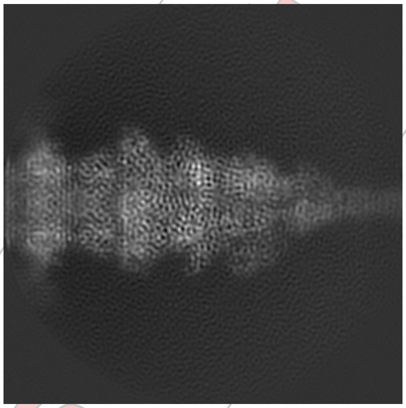
Y

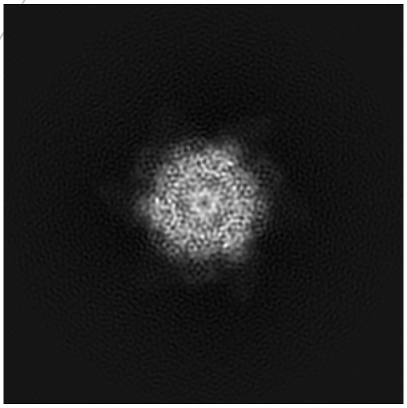
Z

6.1.2 Raw map

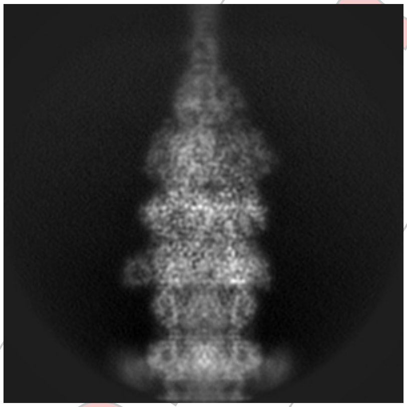
X

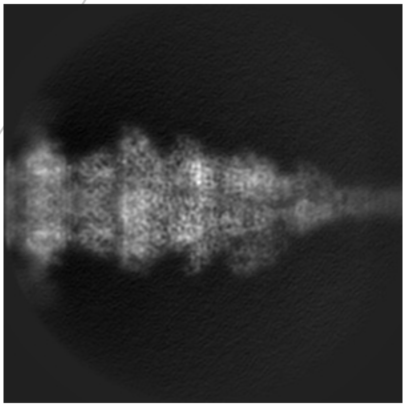
Y

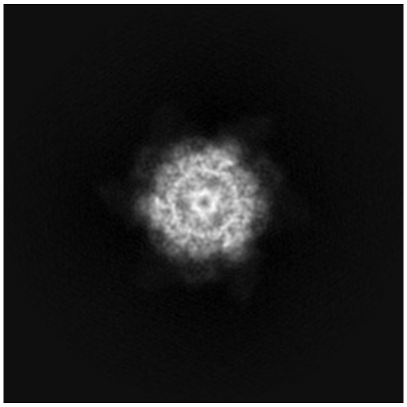
Z

The images above show the map projected in three orthogonal directions.

For Manuscript Review

6.2 Central slices (i)

6.2.1 Primary map

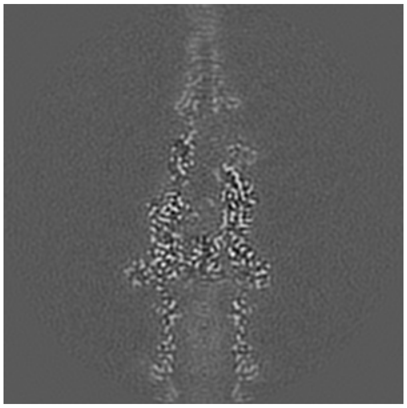
X Index: 110

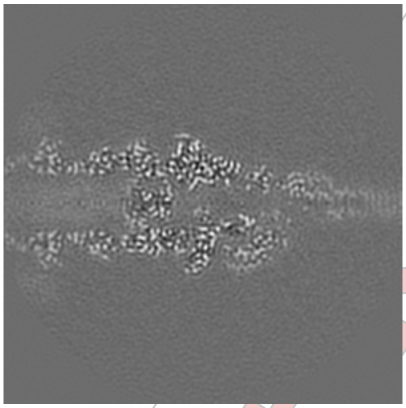
Y Index: 110

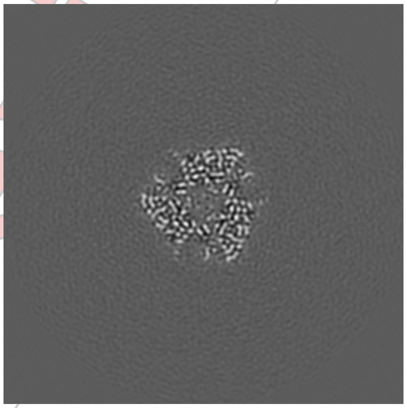
Z Index: 110

6.2.2 Raw map

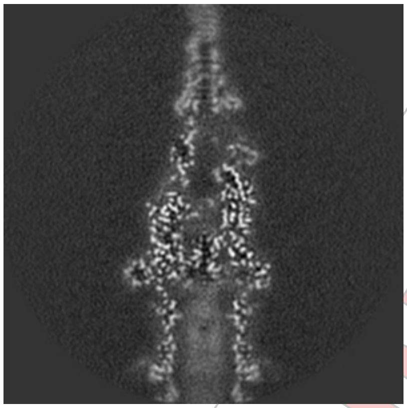
X Index: 110

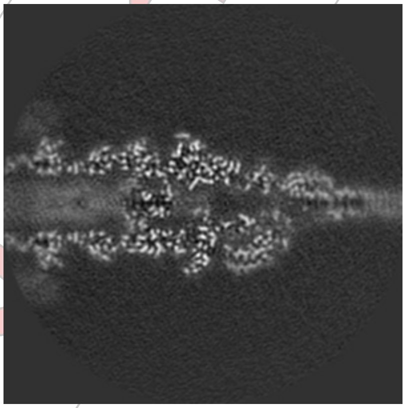
Y Index: 110

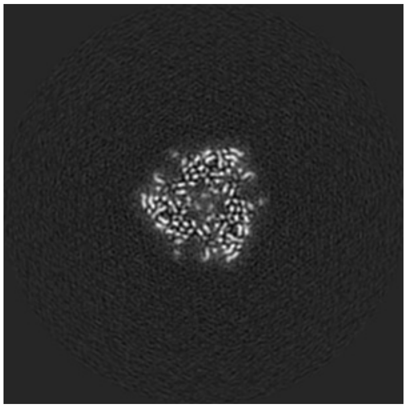
Z Index: 110

The images above show central slices of the map in three orthogonal directions.

For Manuscript Review

6.3 Largest variance slices ⓘ

6.3.1 Primary map

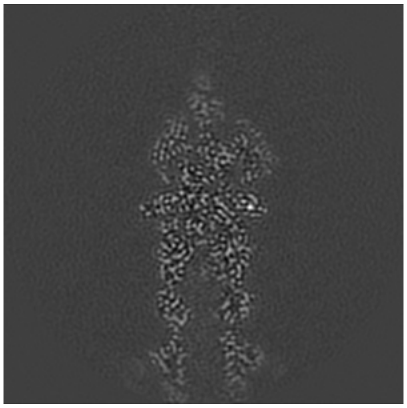
X Index: 122

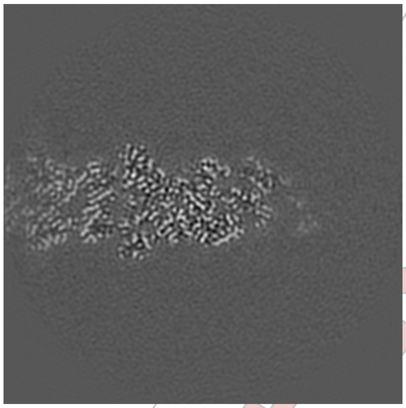
Y Index: 94

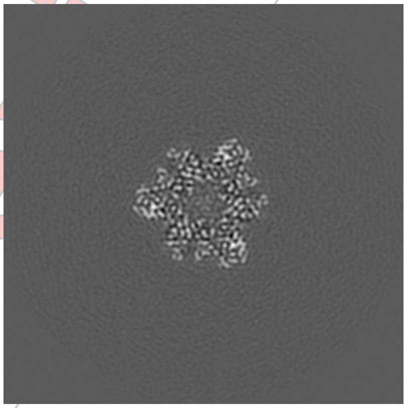
Z Index: 107

6.3.2 Raw map

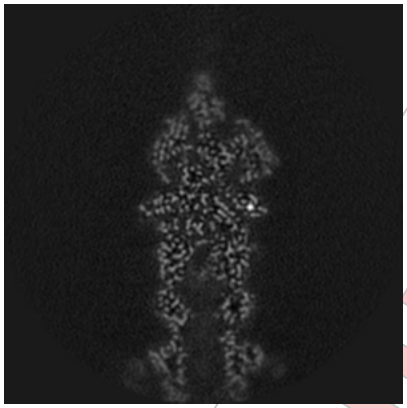
X Index: 122

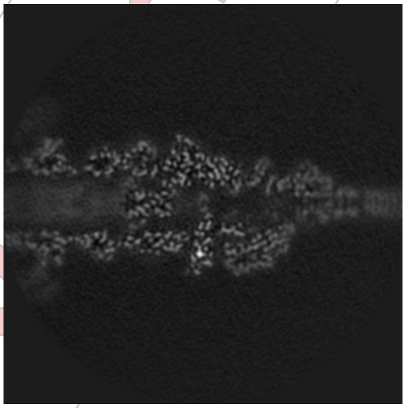
Y Index: 108

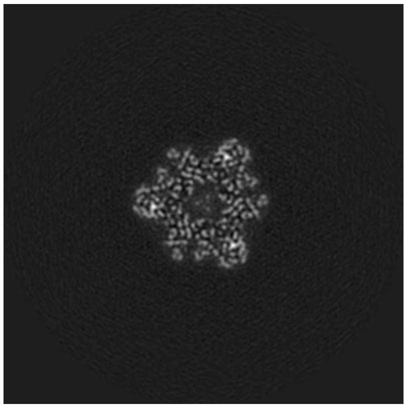
Z Index: 107

The images above show the largest variance slices of the map in three orthogonal directions.

For Manuscript Review

6.4 Orthogonal standard-deviation projections (False-color) (i)

6.4.1 Primary map

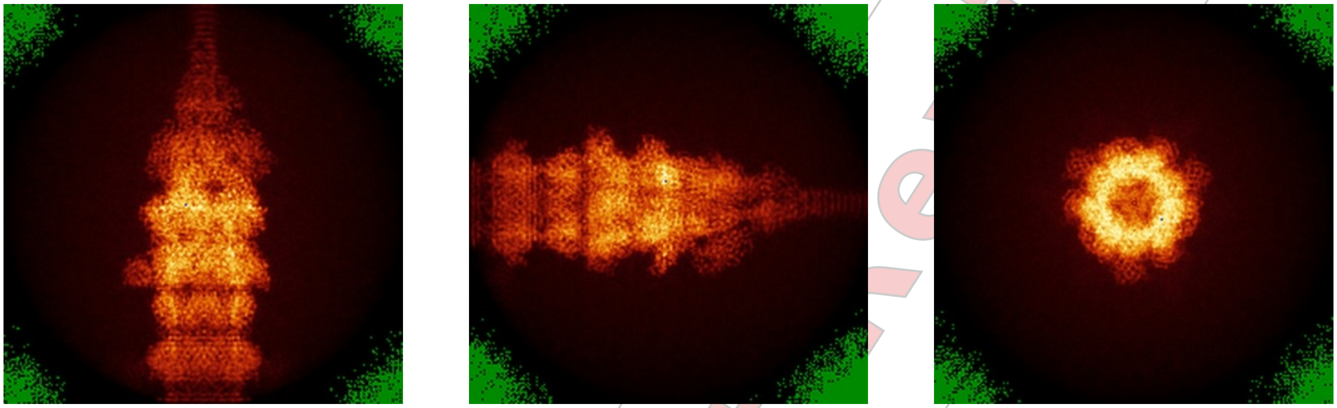

X Y Z

6.4.2 Raw map

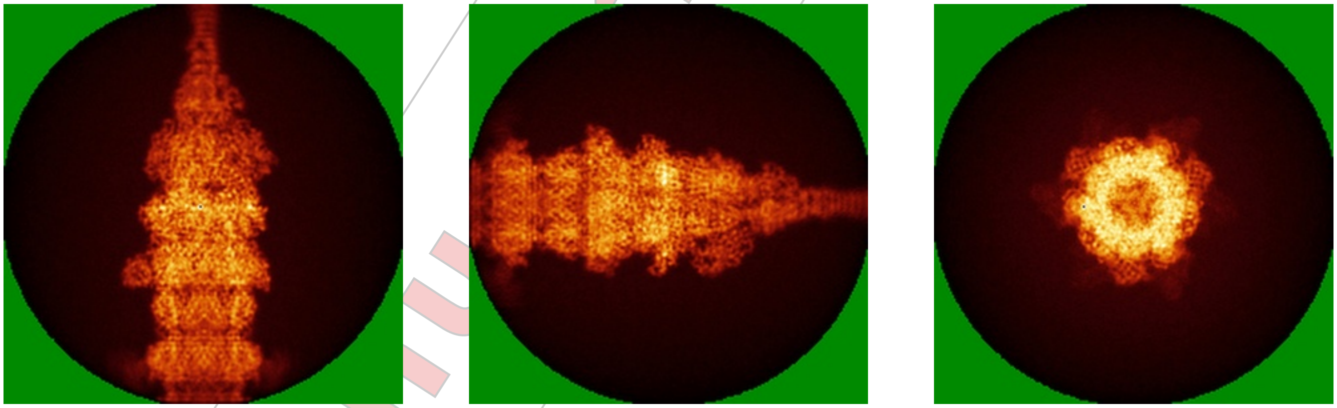

X Y Z

The images above show the map standard deviation projections with false color in three orthogonal directions. Minimum values are shown in green, max in blue, and dark to light orange shades represent small to large values respectively.

For Manuscript Review

6.5 Orthogonal surface views (i)

6.5.1 Primary map

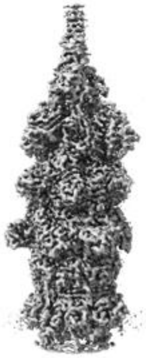

X

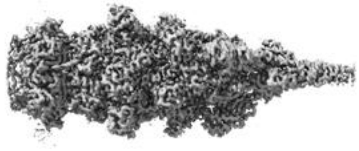

Y

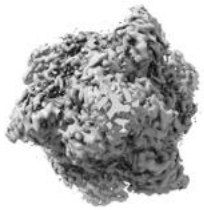

Z

The images above show the 3D surface view of the map at the recommended contour level 0.02. These images, in conjunction with the slice images, may facilitate assessment of whether an appropriate contour level has been provided.

6.5.2 Raw map

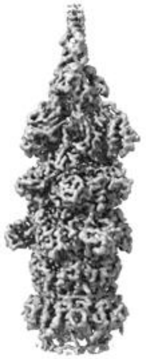

X

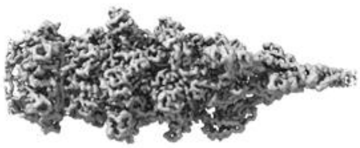

Y

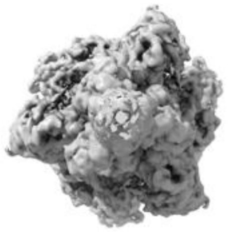

Z

These images show the 3D surface of the raw map. The raw map's contour level was selected so that its surface encloses the same volume as the primary map does at its recommended contour level.

6.6 Mask visualisation (i)

This section was not generated. No masks/segmentation were deposited.

For Manuscript Review

7 Map analysis (i)

This section contains the results of statistical analysis of the map.

7.1 Map-value distribution (i)

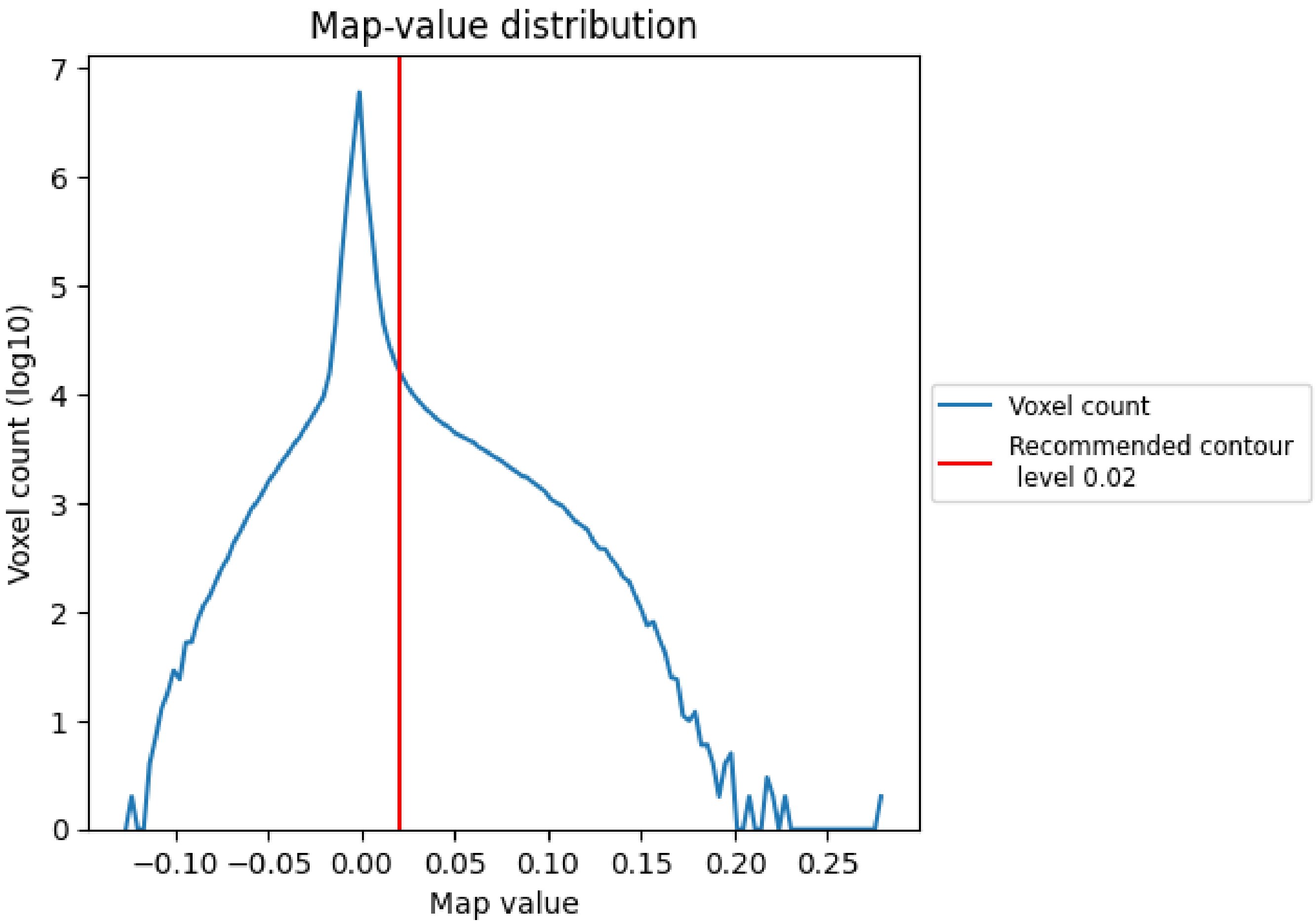

The map-value distribution is plotted in 128 intervals along the x-axis. The y-axis is logarithmic. A spike in this graph at zero usually indicates that the volume has been masked.

For Manuscript Review

7.2 Volume estimate (i)

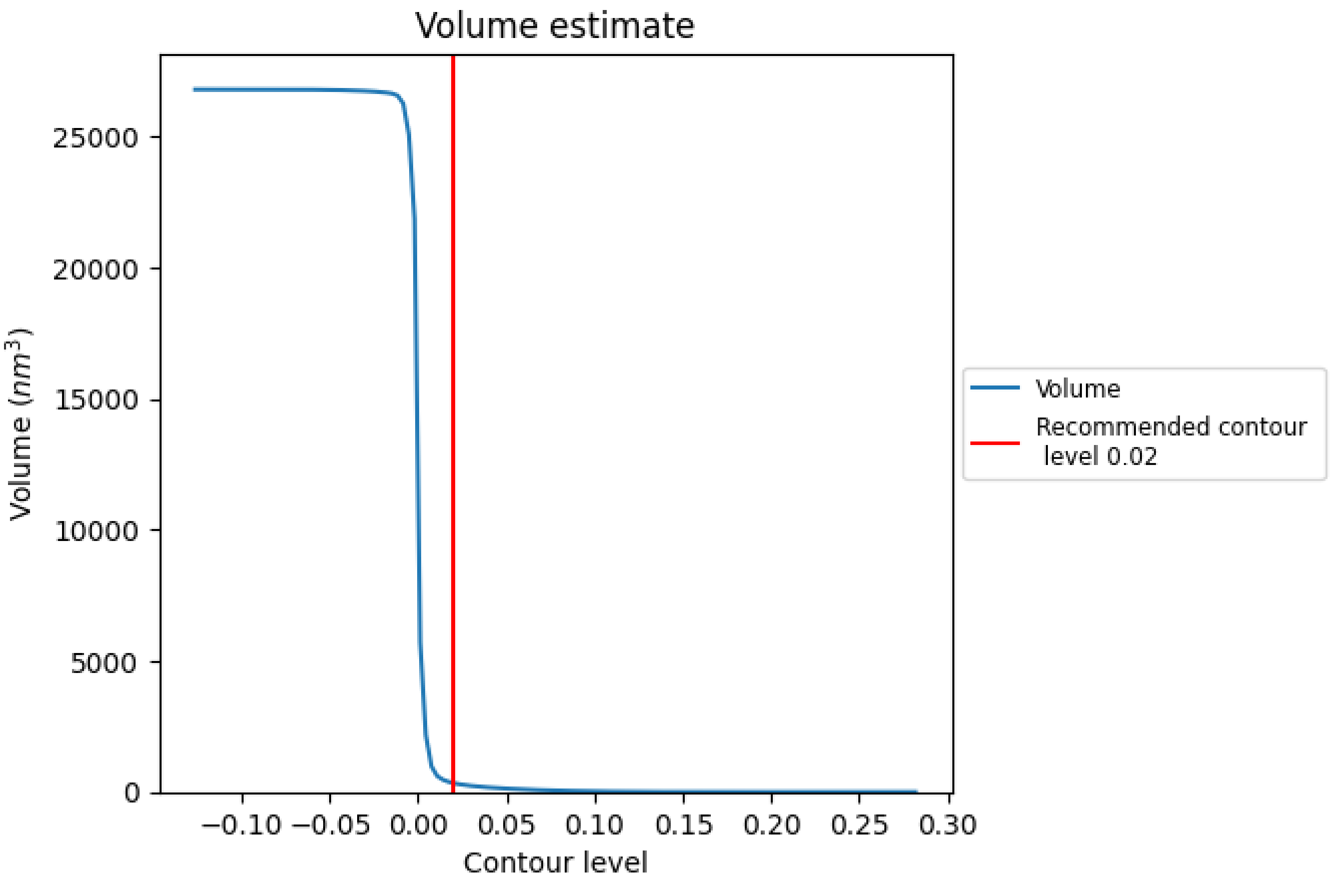

The volume at the recommended contour level is 340 nm^3; this corresponds to an approximate mass of 307 kDa.

The volume estimate graph shows how the enclosed volume varies with the contour level. The recommended contour level is shown as a vertical line and the intersection between the line and the curve gives the volume of the enclosed surface at the given level.

For Manuscript Review

7.3 Rotationally averaged power spectrum (i)

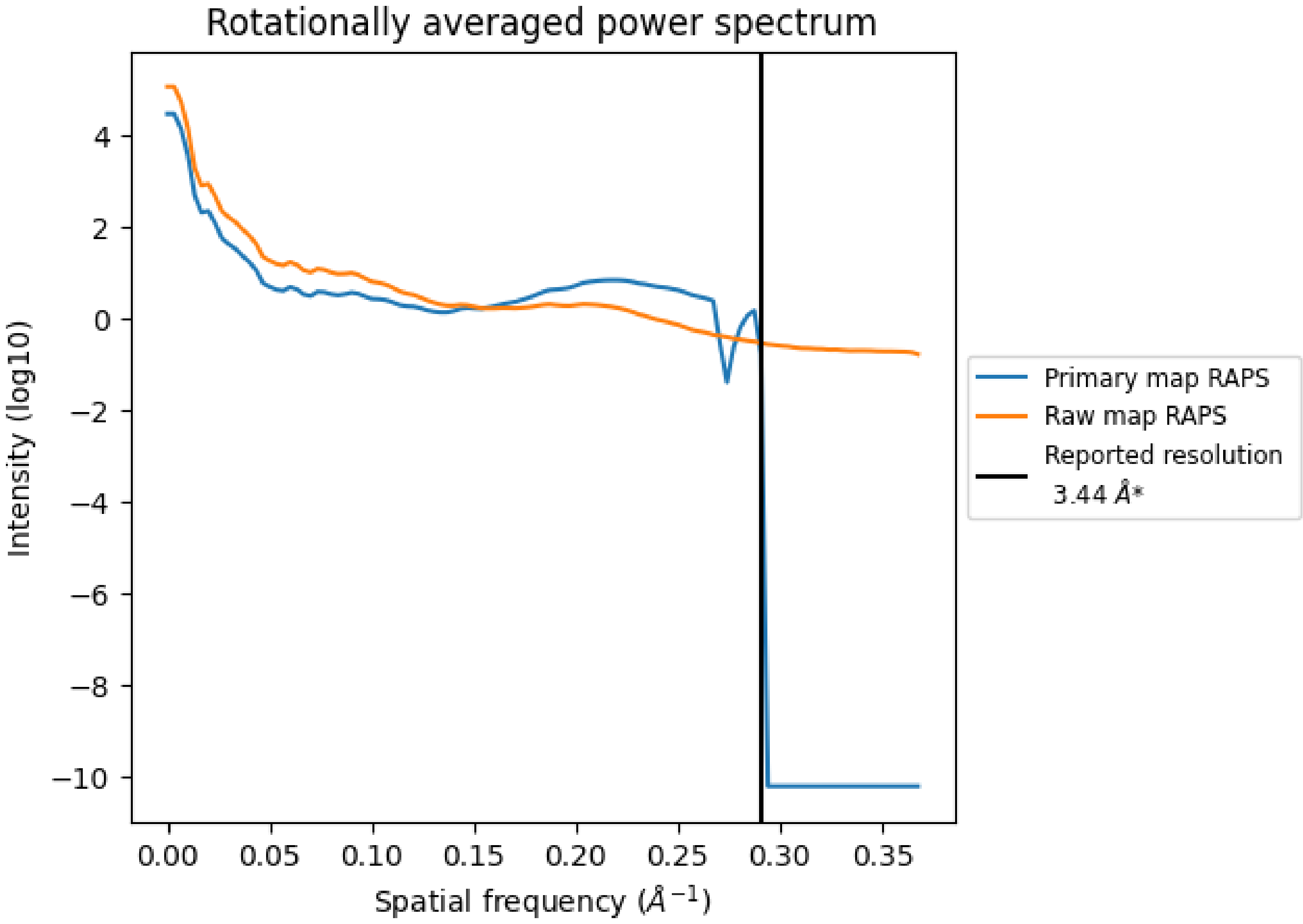

*Reported resolution corresponds to spatial frequency of 0.291 Å^{-1}

For Manuscript Review

8 Fourier-Shell correlation (i)

Fourier-Shell Correlation (FSC) is the most commonly used method to estimate the resolution of single-particle and subtomogram-averaged maps. The shape of the curve depends on the imposed symmetry, mask and whether or not the two 3D reconstructions used were processed from a common reference. The reported resolution is shown as a black line. A curve is displayed for the half-bit criterion in addition to lines showing the 0.143 gold standard cut-off and 0.5 cut-off.

8.1 FSC (i)

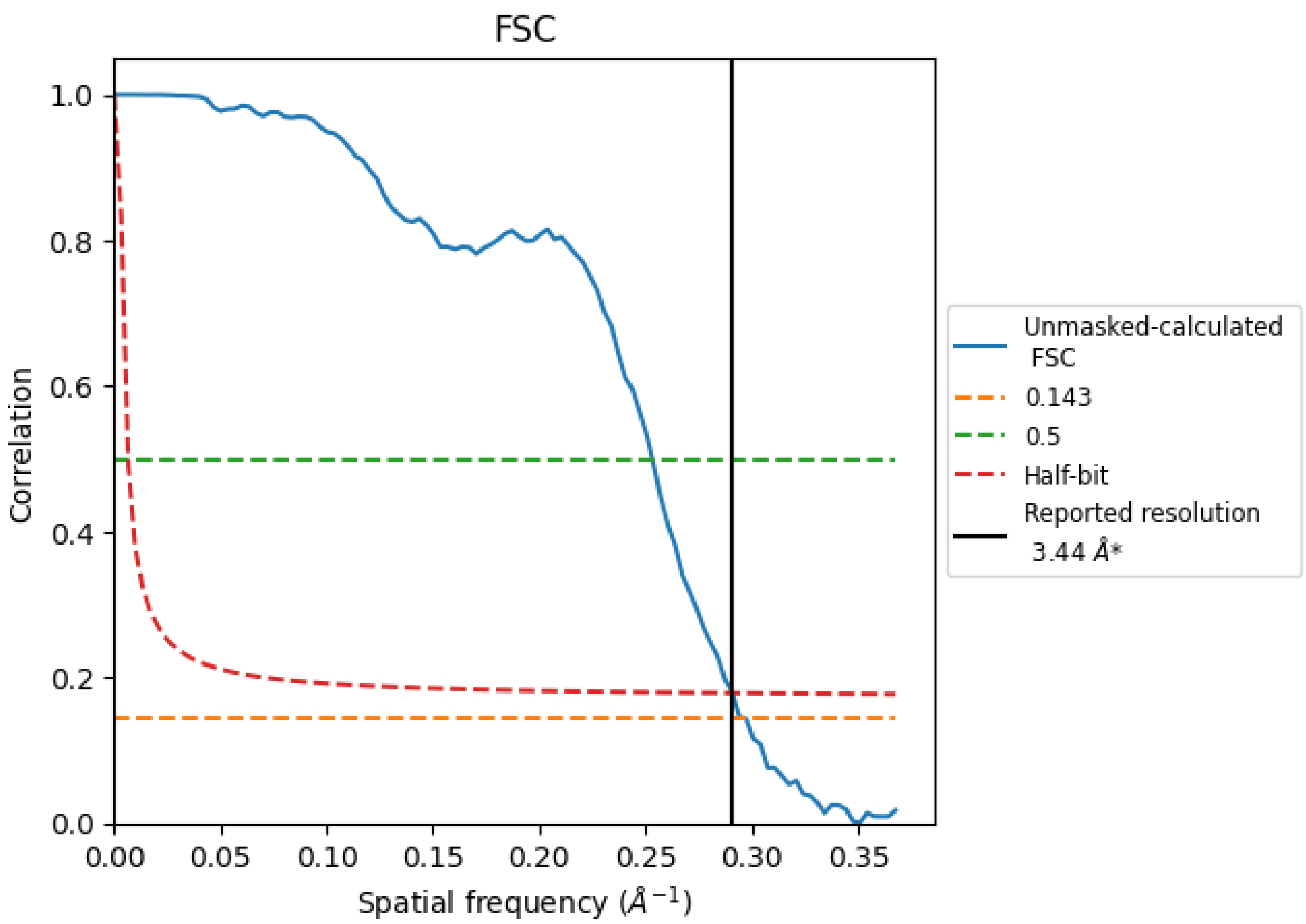

*Reported resolution corresponds to spatial frequency of 0.291 Å^{-1}

For Manuscript Review

8.2 Resolution estimates (i)

Resolution estimate (Å)	Estimation criterion (FSC cut-off)		
	0.143	0.5	Half-bit
Reported by author	3.44	-	-
Author-provided FSC curve	-	-	-
Unmasked-calculated*	3.37	3.95	3.44

*Resolution estimate based on FSC curve calculated by comparison of deposited half-maps.

For Manuscript Review

9 Map-model fit ⓘ

This section contains information regarding the fit between EMDB map EMD-36844 and PDB model 8K35. Per-residue inclusion information can be found in section 3 on page 7.

9.1 Map-model overlay ⓘ

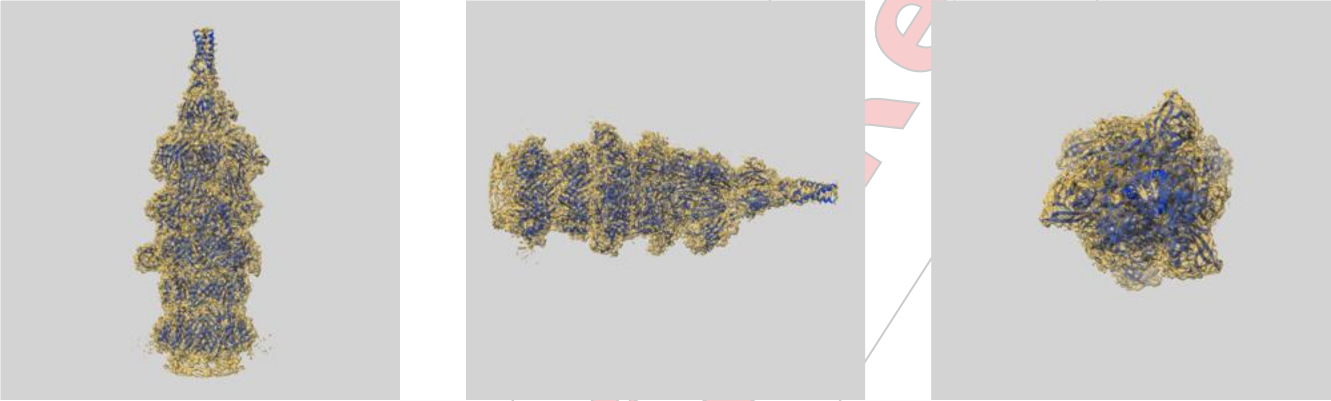

X Y Z

The images above show the 3D surface view of the map at the recommended contour level 0.02 at 50% transparency in yellow overlaid with a ribbon representation of the model coloured in blue. These images allow for the visual assessment of the quality of fit between the atomic model and the map.

For Manuscript Review

9.2 Q-score mapped to coordinate model (i)

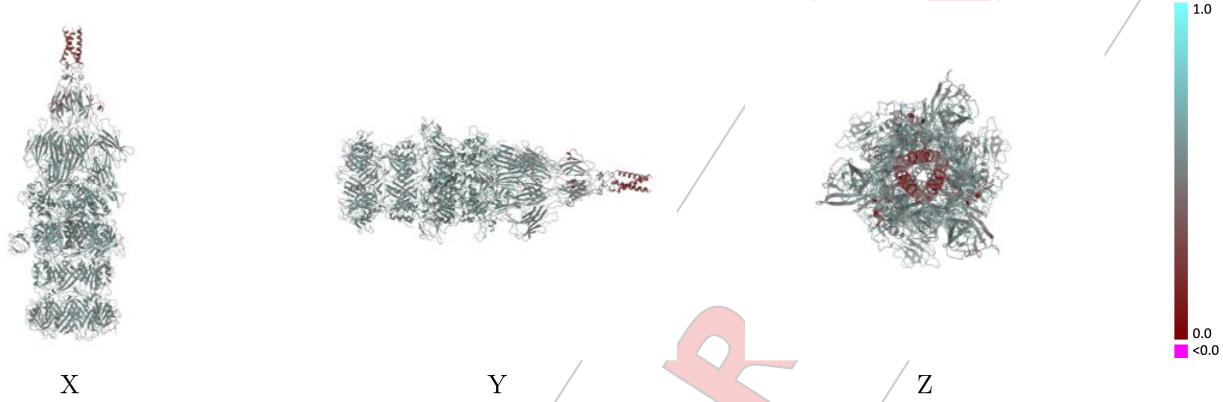

The images above show the model with each residue coloured according its Q-score. This shows their resolvability in the map with higher Q-score values reflecting better resolvability. Please note: Q-score is calculating the resolvability of atoms, and thus high values are only expected at resolutions at which atoms can be resolved. Low Q-score values may therefore be expected for many entries.

9.3 Atom inclusion mapped to coordinate model (i)

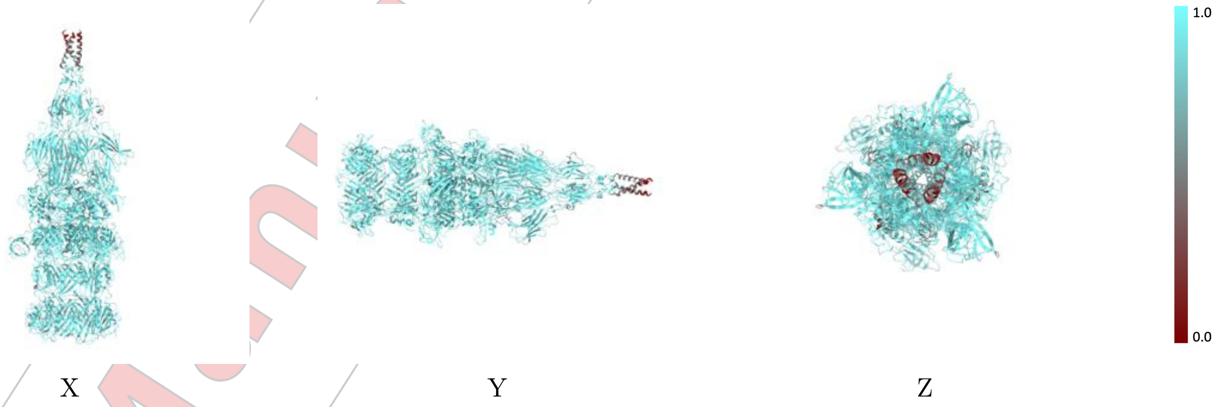

The images above show the model with each residue coloured according to its atom inclusion. This shows to what extent they are inside the map at the recommended contour level (0.02).

For Manuscript Review

9.4 Atom inclusion (i)

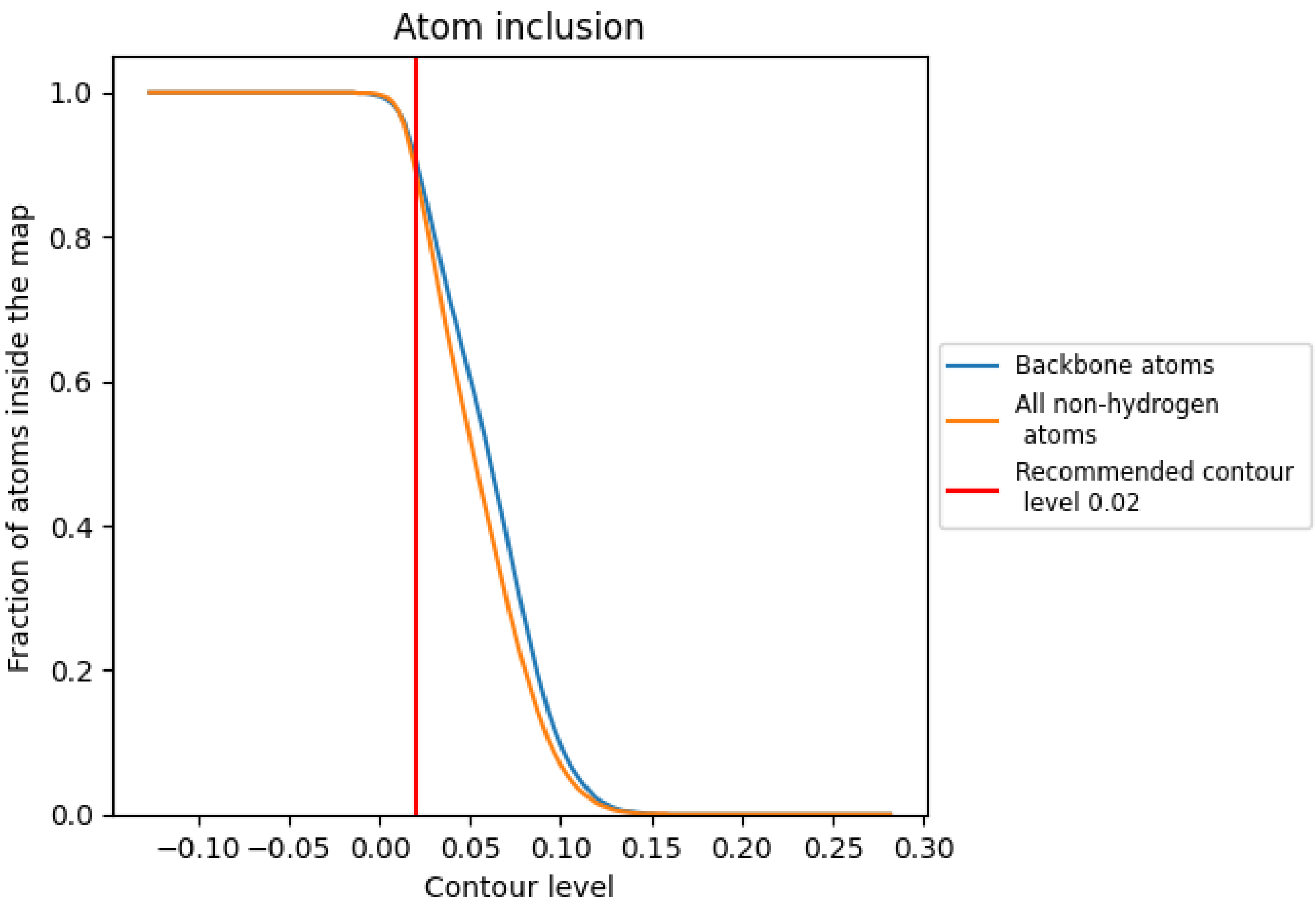

At the recommended contour level, 91% of all backbone atoms, 89% of all non-hydrogen atoms, are inside the map.

For Manuscript Review

9.5 Map-model fit summary (i)

The table lists the average atom inclusion at the recommended contour level (0.02) and Q-score for the entire model and for each chain.

Chain	Atom inclusion	Q-score
All	0.8940	0.5390
A	0.8810	0.5260
B	0.8810	0.5270
C	0.9230	0.5580
D	0.9270	0.5580
E	0.9290	0.5640
F	0.9310	0.5630
G	0.9320	0.5560
H	0.9200	0.5600
I	0.8810	0.5280
J	0.9350	0.5570
K	0.9200	0.5570
L	0.8690	0.5370
M	0.8930	0.5350
N	0.9360	0.5550
O	0.9310	0.5620
P	0.9020	0.5330
Q	0.9060	0.5530
R	0.8700	0.5410
S	0.9060	0.5350
T	0.8690	0.5370
U	0.8700	0.5410
V	0.8700	0.5380
W	0.8630	0.5410
x	0.9190	0.5550

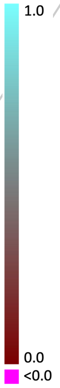

For Manuscript Review
