# Supplementary material for: Structure of the siphophage neck–Tail complex suggests that conserved tail tip proteins facilitate receptor binding and tail assembly
Source: PLoS Biol. 2023 Dec 14;21(12):e3002441. doi: 10.1371/journal.pbio.3002441 (PMC10721106; doi:10.1371/journal.pbio.3002441)
Supplement: S1 Appendix — (ZIP) [file pbio.3002441.s021.zip › PDB Validation Report.zip!/D_1300039349_report-portal-caspsid.pdf]

Full wwPDB EM Validation Report (i)

Jul 19, 2023 – 06:29 PM JST

PDB ID	:	8K39
EMDB ID	:	EMD-36848
Title	:	Structure of the bacteriophage lambda portal vertex
Deposited on	:	2023-07-14
Resolution	:	4.00 Å(reported)

This wwPDB validation report is for manuscript review

This is a Full wwPDB EM Validation Report.

This report is produced by the wwPDB biocuration pipeline after annotation of the structure.

We welcome your comments at *validation@mail.wwpdb.org*
A user guide is available at
https://www.wwpdb.org/validation/2017/EMValidationReportHelp
with specific help available everywhere you see the (i) symbol.

The types of validation reports are described at
http://www.wwpdb.org/validation/2017/FAQs#types.

The following versions of software and data (see references (i)) were used in the production of this report:

EMDB validation analysis	:	0.0.1.dev50
MolProbity	:	4.02b-467
Percentile statistics	:	20191225.v01 (using entries in the PDB archive December 25th 2019)
MapQ	:	1.9.9
Ideal geometry (proteins)	:	Engh & Huber (2001)
Ideal geometry (DNA, RNA)	:	Parkinson et al. (1996)
Validation Pipeline (wwPDB-VP)	:	2.34

For Manuscript Review

1 Overall quality at a glance (i)

The following experimental techniques were used to determine the structure:
ELECTRON MICROSCOPY

The reported resolution of this entry is 4.00 Å.

Percentile scores (ranging between 0-100) for global validation metrics of the entry are shown in the following graphic. The table shows the number of entries on which the scores are based.

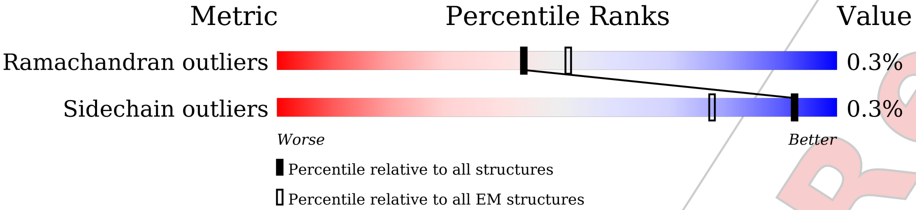

Metric	Whole archive (#Entries)	EM structures (#Entries)
Ramachandran outliers	154571	4023
Sidechain outliers	154315	3826

The table below summarises the geometric issues observed across the polymeric chains and their fit to the map. The red, orange, yellow and green segments of the bar indicate the fraction of residues that contain outliers for >=3, 2, 1 and 0 types of geometric quality criteria respectively. A grey segment represents the fraction of residues that are not modelled. The numeric value for each fraction is indicated below the corresponding segment, with a dot representing fractions <=5% The upper red bar (where present) indicates the fraction of residues that have poor fit to the EM map (all-atom inclusion < 40%). The numeric value is given above the bar.

Mol	Chain	Length	Quality of chain
1	0	341	99% •
1	1	341	• 99% •
1	2	341	• 99% •
1	3	341	• 98% ••
1	4	341	99% •
1	5	341	• 98% ••
1	6	341	7% 99% •
1	7	341	9% 99% •
1	A	341	• 99% •

Continued on next page...

For Manuscript Review

Continued from previous page...

Mol	Chain	Length	Quality of chain
1	B	341	99%
1	C	341	99%
1	D	341	99%
1	E	341	12% 99%
1	F	341	11% 99%
1	G	341	10% 100%
1	H	341	13% 99%
1	I	341	99%
1	J	341	13% 99%
1	K	341	8% 99%
1	L	341	99%
1	M	341	99%
1	N	341	98%
1	O	341	8% 99%
1	P	341	9% 99%
1	Q	341	99%
1	R	341	99%
1	S	341	99%
1	T	341	98%
1	g	341	99%
1	h	341	98%
2	U	533	88% 12%
2	V	533	88% 11%
2	W	533	89% 11%
2	X	533	88% 11%

Continued on next page...

For Manuscript Review

Continued from previous page...

Mol	Chain	Length	Quality of chain
2	Y	533	89% 11%
2	Z	533	88% 11%
2	a	533	89% 11%
2	b	533	88% 11%
2	c	533	88% • 11%
2	d	533	89% 11%
2	e	533	89% 11%
2	f	533	88% 11%

For Manuscript Review

2 Entry composition (i)

There are 2 unique types of molecules in this entry. The entry contains 124618 atoms, of which 0 are hydrogens and 0 are deuteriums.

In the tables below, the AltConf column contains the number of residues with at least one atom in alternate conformation and the Trace column contains the number of residues modelled with at most 2 atoms.

- Molecule 1 is a protein called Major capsid protein.

Mol	Chain	Residues	Atoms					AltConf	Trace
1	0	340	Total 2675	C 1685	N 458	O 519	S 13	0	0
1	1	340	Total 2675	C 1685	N 458	O 519	S 13	0	0
1	2	340	Total 2675	C 1685	N 458	O 519	S 13	0	0
1	3	337	Total 2649	C 1668	N 455	O 514	S 12	0	0
1	4	337	Total 2649	C 1668	N 455	O 514	S 12	0	0
1	5	337	Total 2649	C 1668	N 455	O 514	S 12	0	0
1	6	340	Total 2675	C 1685	N 458	O 519	S 13	0	0
1	7	340	Total 2675	C 1685	N 458	O 519	S 13	0	0
1	A	340	Total 2675	C 1685	N 458	O 519	S 13	0	0
1	B	340	Total 2675	C 1685	N 458	O 519	S 13	0	0
1	C	340	Total 2675	C 1685	N 458	O 519	S 13	0	0
1	D	340	Total 2675	C 1685	N 458	O 519	S 13	0	0
1	E	340	Total 2675	C 1685	N 458	O 519	S 13	0	0
1	F	340	Total 2675	C 1685	N 458	O 519	S 13	0	0
1	G	340	Total 2675	C 1685	N 458	O 519	S 13	0	0
1	H	340	Total 2675	C 1685	N 458	O 519	S 13	0	0
1	I	340	Total 2675	C 1685	N 458	O 519	S 13	0	0

Continued on next page...

For Manuscript Review

Continued from previous page...

Mol	Chain	Residues	Atoms					AltConf	Trace
1	J	340	Total 2675	C 1685	N 458	O 519	S 13	0	0
1	K	340	Total 2675	C 1685	N 458	O 519	S 13	0	0
1	L	340	Total 2675	C 1685	N 458	O 519	S 13	0	0
1	M	340	Total 2675	C 1685	N 458	O 519	S 13	0	0
1	N	337	Total 2649	C 1668	N 455	O 514	S 12	0	0
1	O	340	Total 2675	C 1685	N 458	O 519	S 13	0	0
1	P	340	Total 2675	C 1685	N 458	O 519	S 13	0	0
1	Q	340	Total 2675	C 1685	N 458	O 519	S 13	0	0
1	R	340	Total 2675	C 1685	N 458	O 519	S 13	0	0
1	S	340	Total 2675	C 1685	N 458	O 519	S 13	0	0
1	T	340	Total 2675	C 1685	N 458	O 519	S 13	0	0
1	g	340	Total 2675	C 1685	N 458	O 519	S 13	0	0
1	h	337	Total 2649	C 1668	N 455	O 514	S 12	0	0

- Molecule 2 is a protein called Portal protein B.

Mol	Chain	Residues	Atoms					AltConf	Trace
2	U	468	Total 3675	C 2294	N 663	O 695	S 23	0	0
2	V	472	Total 3708	C 2317	N 668	O 699	S 24	0	0
2	W	472	Total 3708	C 2317	N 668	O 699	S 24	0	0
2	X	472	Total 3708	C 2317	N 668	O 699	S 24	0	0
2	Y	472	Total 3708	C 2317	N 668	O 699	S 24	0	0
2	Z	472	Total 3708	C 2317	N 668	O 699	S 24	0	0

Continued on next page...

For Manuscript Review

Continued from previous page...

Mol	Chain	Residues	Atoms					AltConf	Trace
2	a	476	Total 3743	C 2339	N 673	O 707	S 24	0	0
2	b	472	Total 3708	C 2317	N 668	O 699	S 24	0	0
2	c	472	Total 3708	C 2317	N 668	O 699	S 24	0	0
2	d	472	Total 3708	C 2317	N 668	O 699	S 24	0	0
2	e	472	Total 3708	C 2317	N 668	O 699	S 24	0	0
2	f	472	Total 3708	C 2317	N 668	O 699	S 24	0	0

For Manuscript Review

3 Residue-property plots (i)

These plots are drawn for all protein, RNA, DNA and oligosaccharide chains in the entry. The first graphic for a chain summarises the proportions of the various outlier classes displayed in the second graphic. The second graphic shows the sequence view annotated by issues in geometry and atom inclusion in map density. Residues are color-coded according to the number of geometric quality criteria for which they contain at least one outlier: green = 0, yellow = 1, orange = 2 and red = 3 or more. A red diamond above a residue indicates a poor fit to the EM map for this residue (all-atom inclusion < 40%). Stretches of 2 or more consecutive residues without any outlier are shown as a green connector. Residues present in the sample, but not in the model, are shown in grey.

- Molecule 1: Major capsid protein

Chain 0:

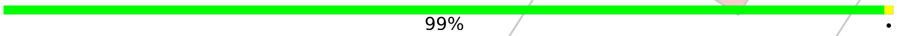

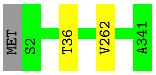

- Molecule 1: Major capsid protein

Chain 1:

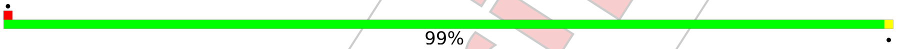

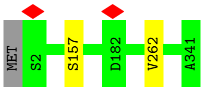

- Molecule 1: Major capsid protein

Chain 2:

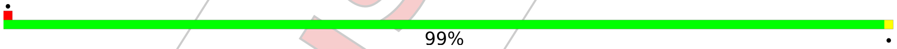

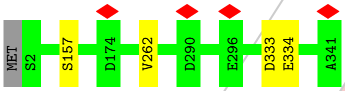

- Molecule 1: Major capsid protein

Chain 3:

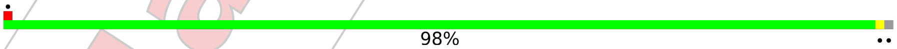

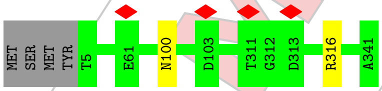

- Molecule 1: Major capsid protein

Chain 4:

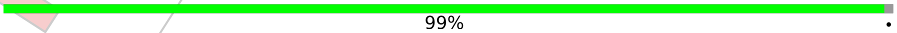

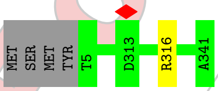

For Manuscript Review

• Molecule 1: Major capsid protein

Chain 5:

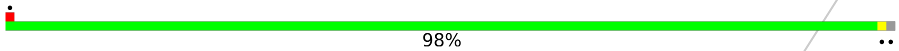

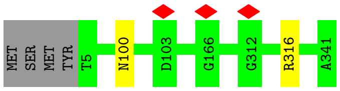

• Molecule 1: Major capsid protein

Chain 6:

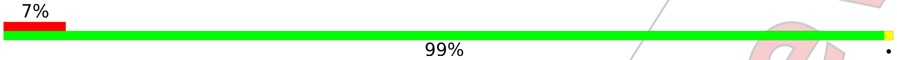

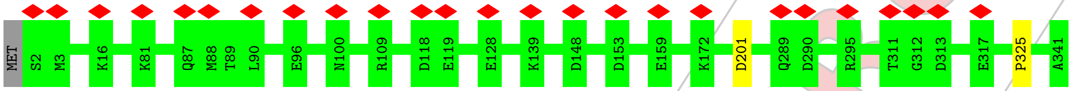

• Molecule 1: Major capsid protein

Chain 7:

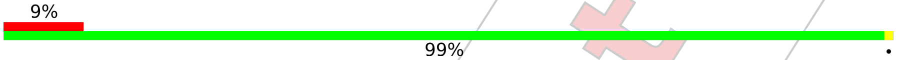

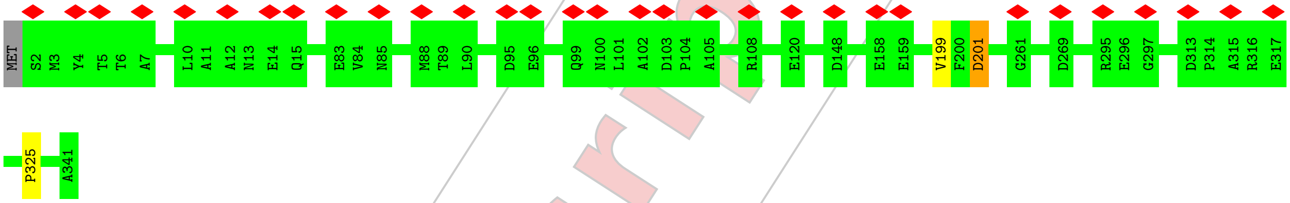

• Molecule 1: Major capsid protein

Chain A:

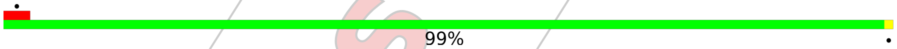

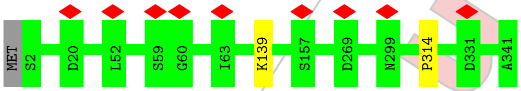

• Molecule 1: Major capsid protein

Chain B:

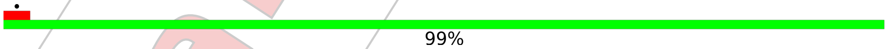

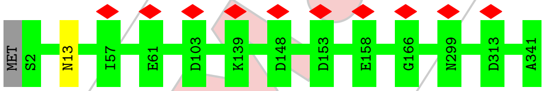

• Molecule 1: Major capsid protein

Chain C:

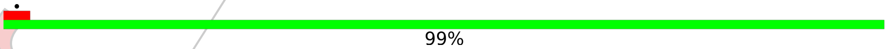

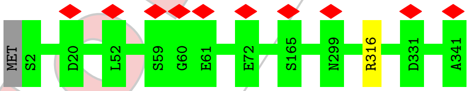

• Molecule 1: Major capsid protein

For Manuscript Review

Chain D:

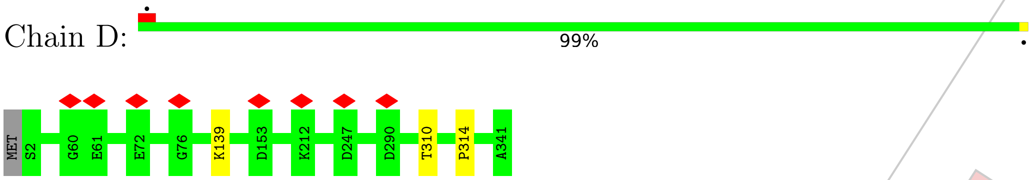

• Molecule 1: Major capsid protein

Chain E:

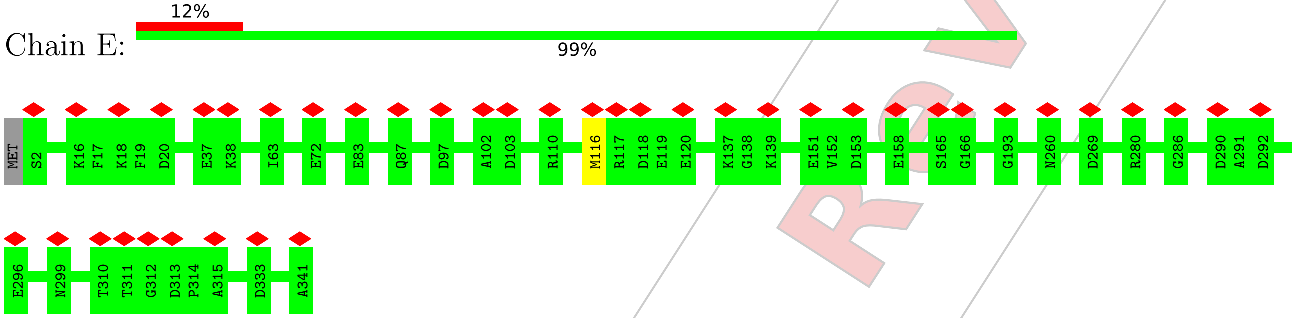

• Molecule 1: Major capsid protein

Chain F:

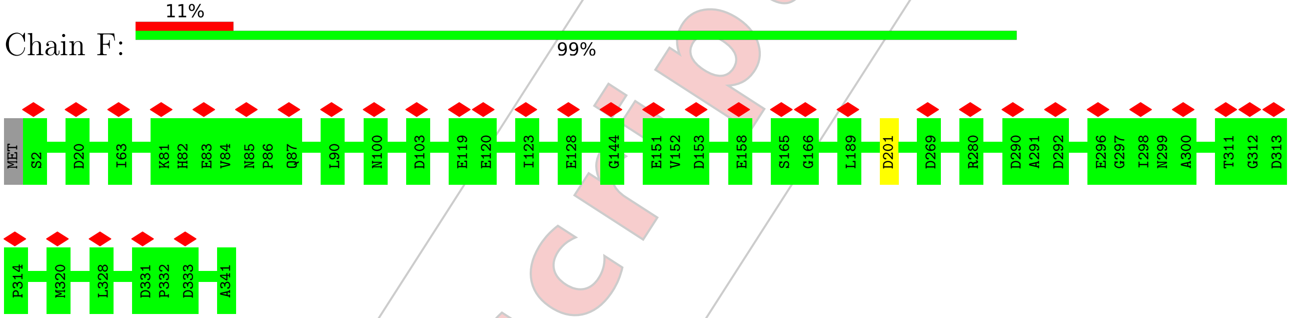

• Molecule 1: Major capsid protein

Chain G:

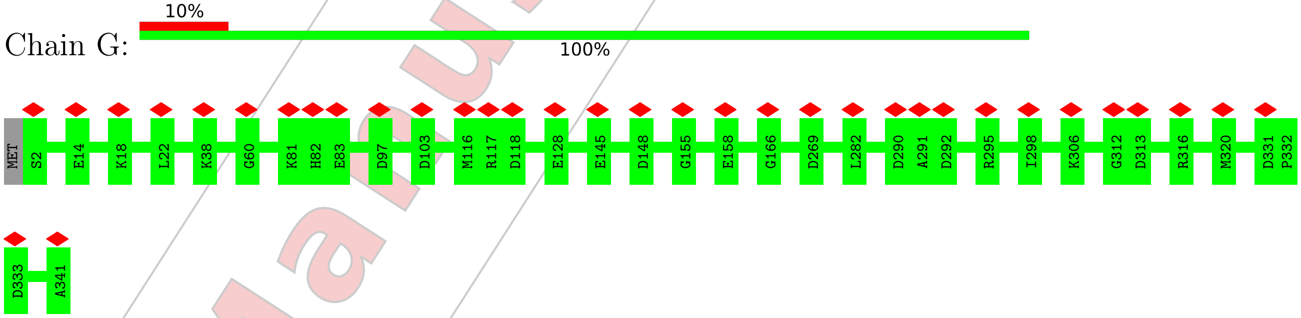

• Molecule 1: Major capsid protein

Chain H:

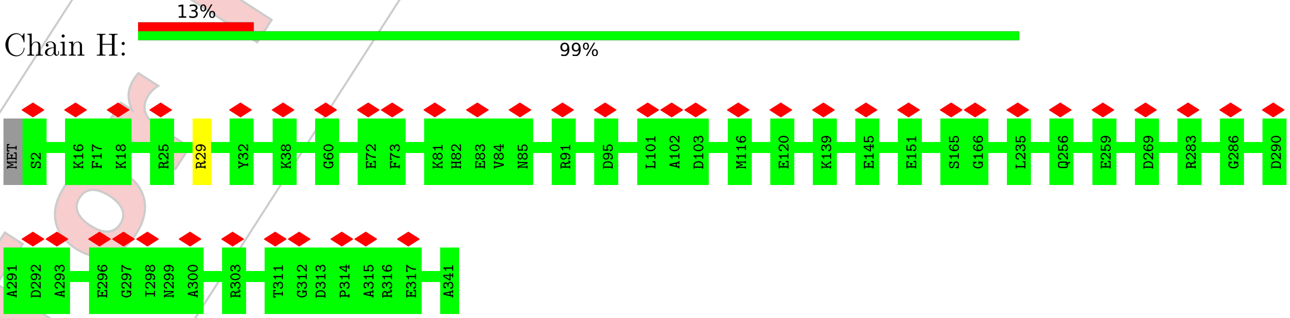

For Manuscript Review

• Molecule 1: Major capsid protein

Chain I:

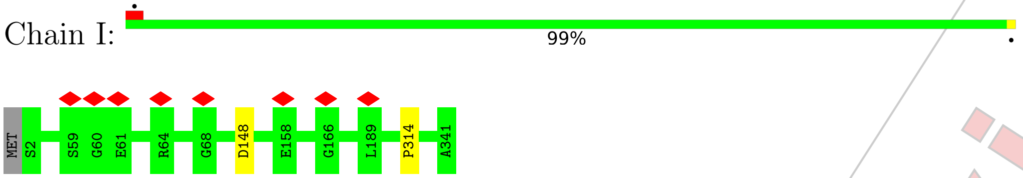

• Molecule 1: Major capsid protein

Chain J:

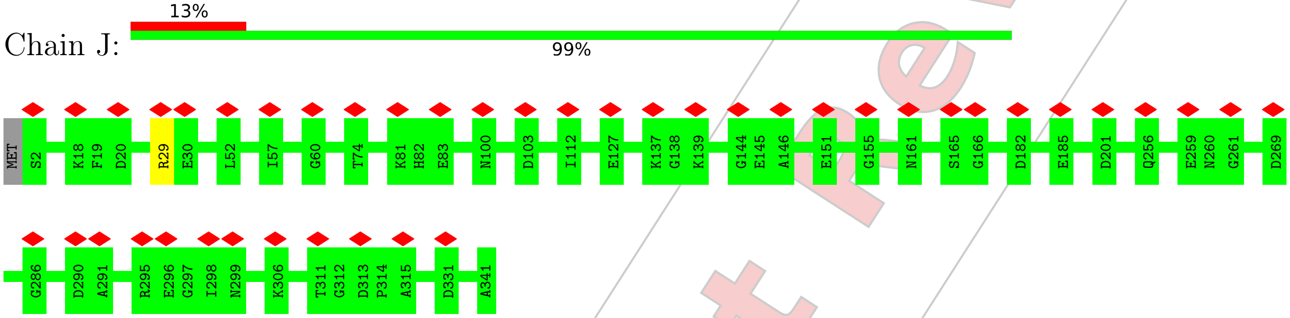

• Molecule 1: Major capsid protein

Chain K:

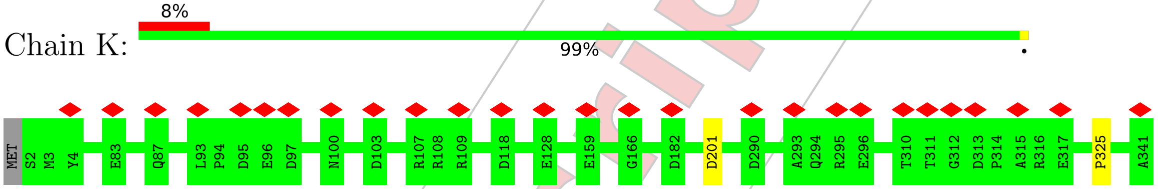

• Molecule 1: Major capsid protein

Chain L:

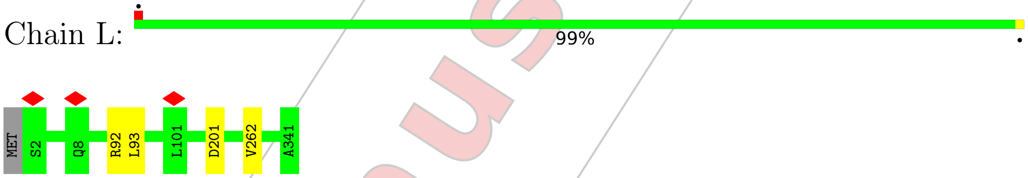

• Molecule 1: Major capsid protein

Chain M:

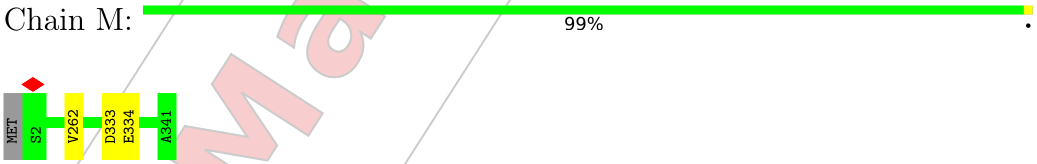

• Molecule 1: Major capsid protein

Chain N:

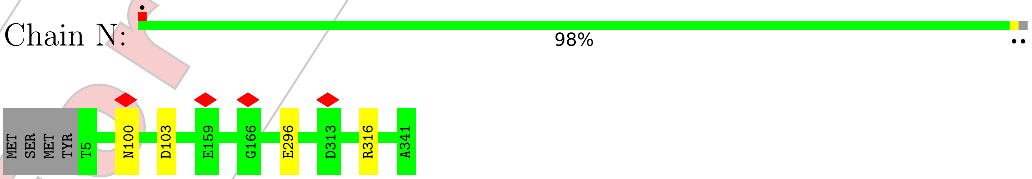

• Molecule 1: Major capsid protein

For Manuscript Review

Chain O:

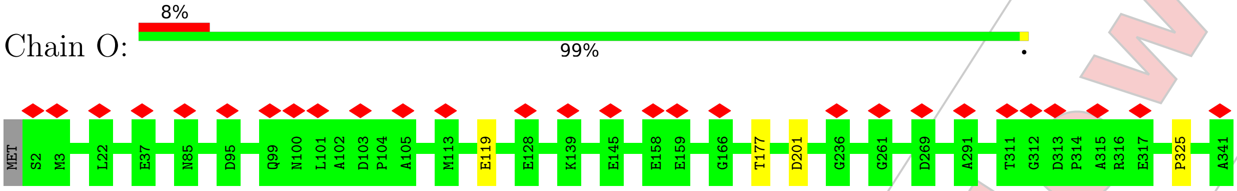

• Molecule 1: Major capsid protein

Chain P:

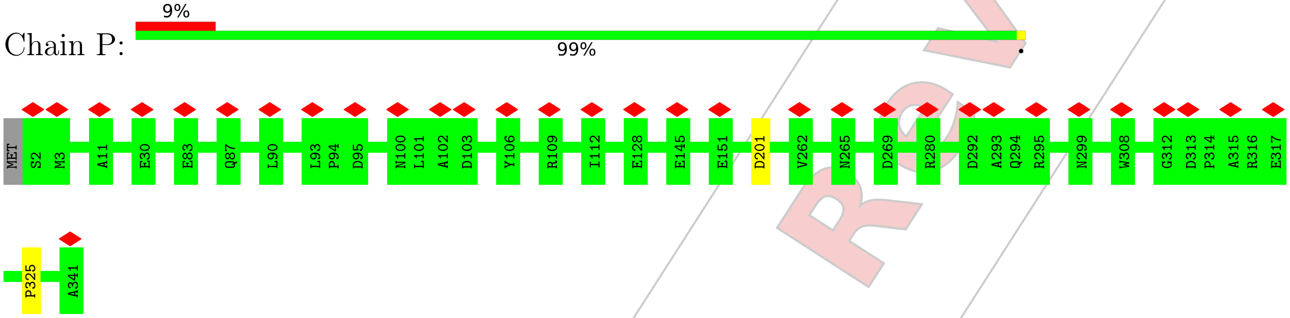

• Molecule 1: Major capsid protein

Chain Q:

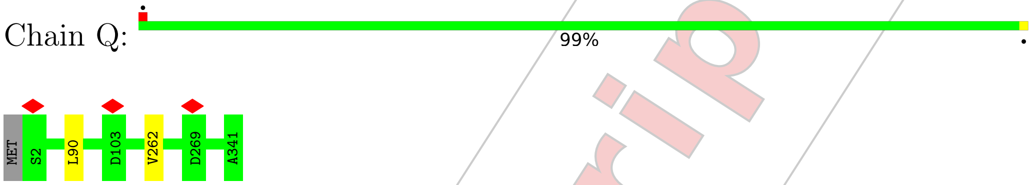

• Molecule 1: Major capsid protein

Chain R:

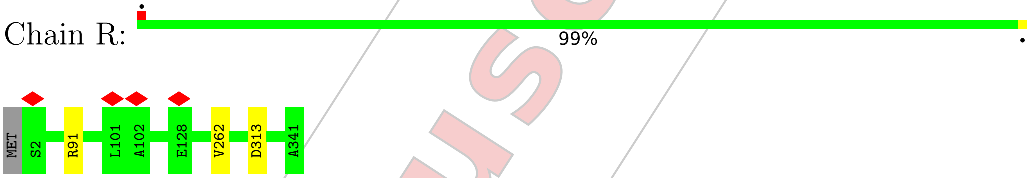

• Molecule 1: Major capsid protein

Chain S:

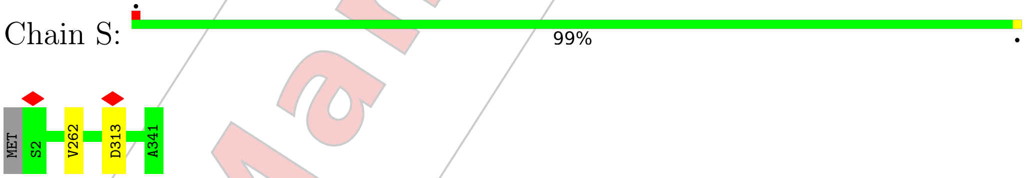

• Molecule 1: Major capsid protein

Chain T:

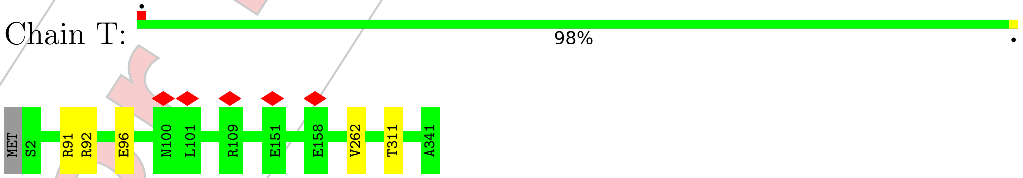

• Molecule 1: Major capsid protein

Chain g:

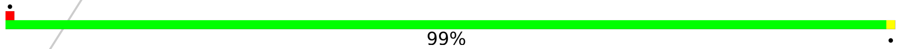

For Manuscript Review

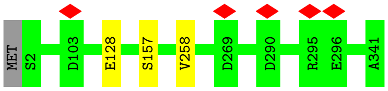

• Molecule 1: Major capsid protein

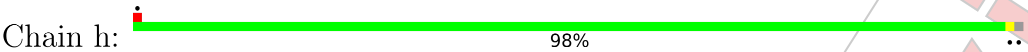

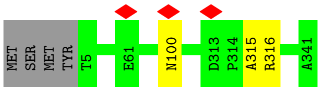

• Molecule 2: Portal protein B

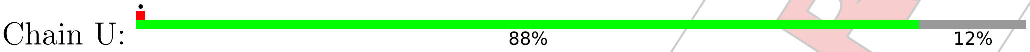

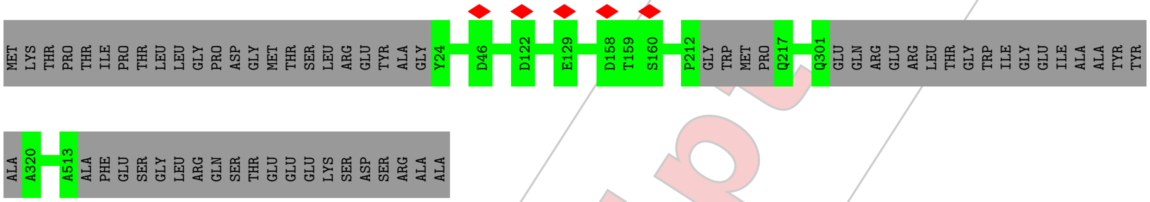

• Molecule 2: Portal protein B

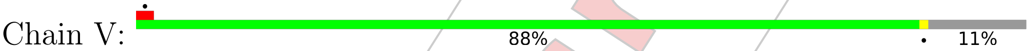

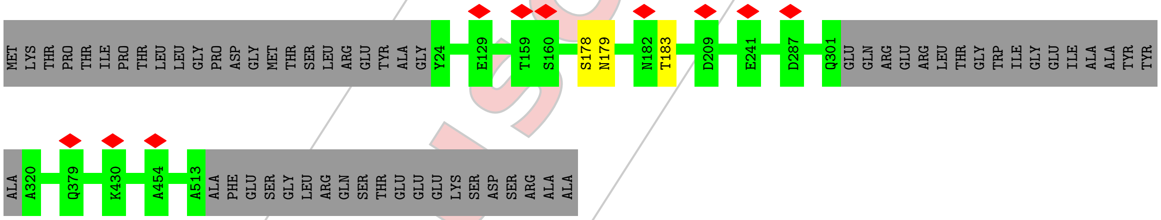

• Molecule 2: Portal protein B

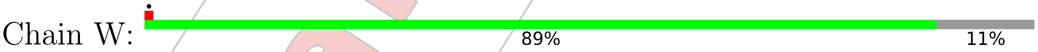

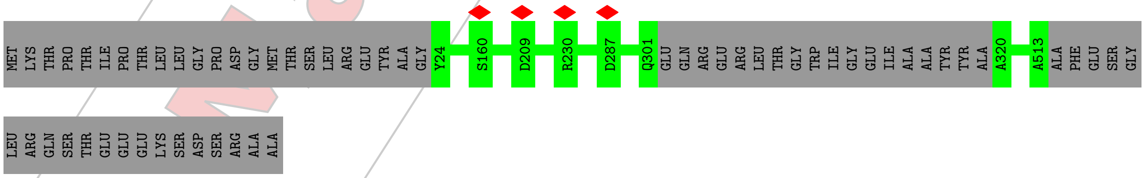

• Molecule 2: Portal protein B

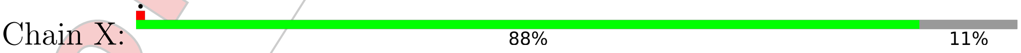

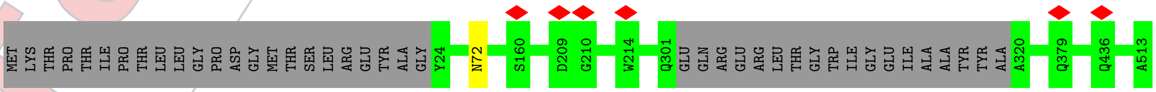

for Manuscript Review

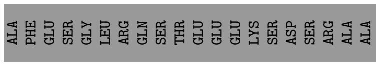

• Molecule 2: Portal protein B

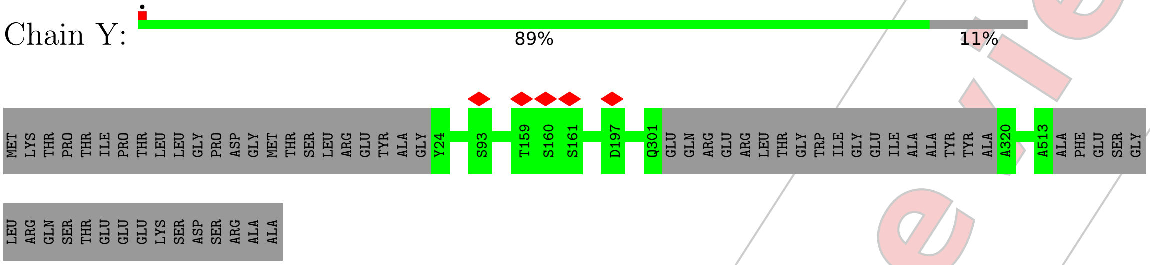

• Molecule 2: Portal protein B

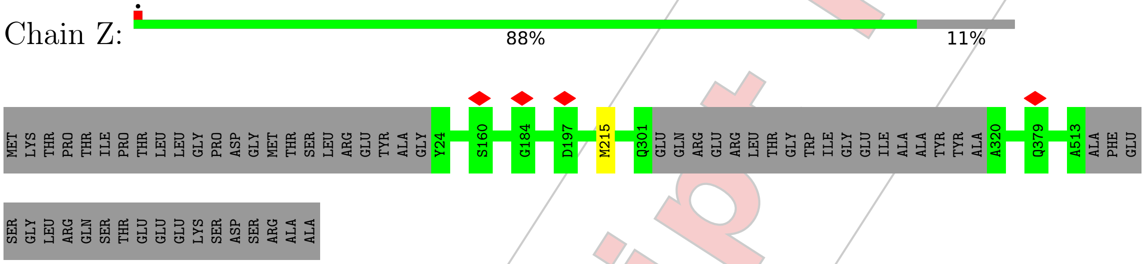

• Molecule 2: Portal protein B

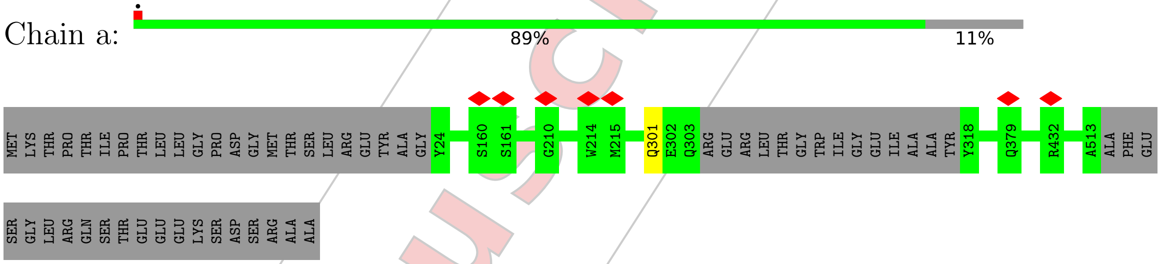

• Molecule 2: Portal protein B

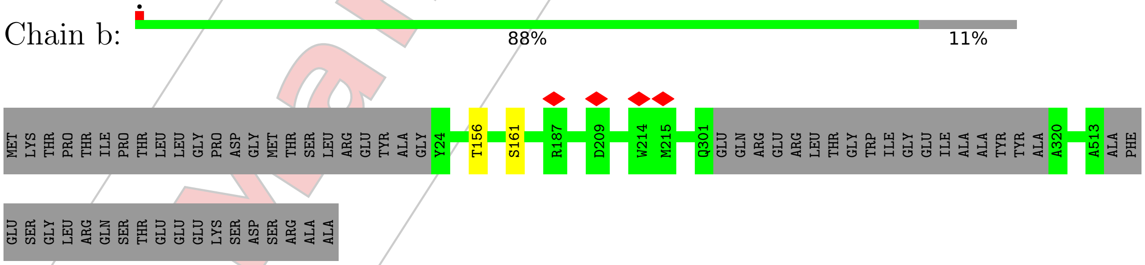

• Molecule 2: Portal protein B

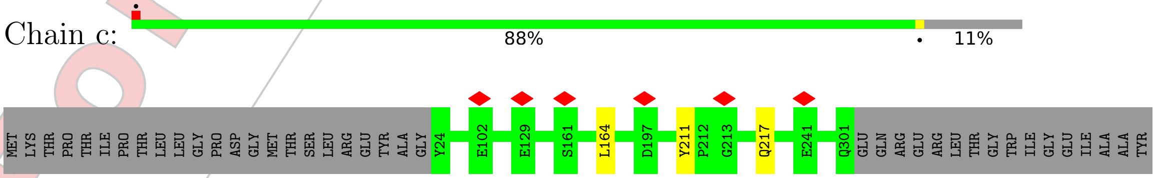

For Manuscript Review

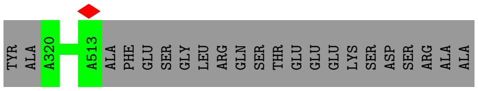

• Molecule 2: Portal protein B

Chain d:

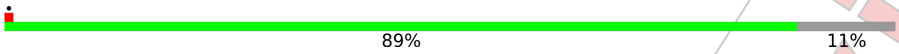

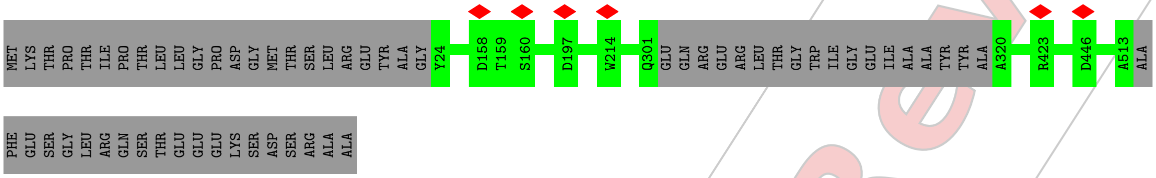

• Molecule 2: Portal protein B

Chain e:

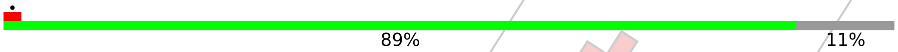

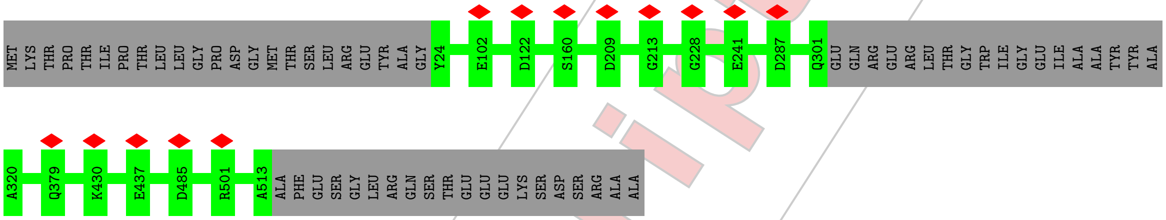

• Molecule 2: Portal protein B

Chain f:

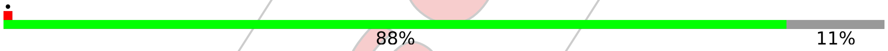

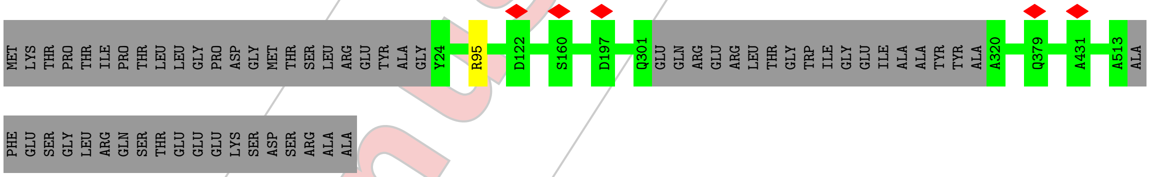

For Manuscript Review

4 Experimental information (i)

Property	Value	Source
EM reconstruction method	SINGLE PARTICLE	Depositor
Imposed symmetry	POINT, Not provided	
Number of particles used	67924	Depositor
Resolution determination method	FSC 0.143 CUT-OFF	Depositor
CTF correction method	PHASE FLIPPING ONLY	Depositor
Microscope	FEI TITAN KRIOS	Depositor
Voltage (kV)	300	Depositor
Electron dose $(e^-/\text{Å}^2)$	32	Depositor
Minimum defocus (nm)	1600	Depositor
Maximum defocus (nm)	2200	Depositor
Magnification	Not provided	
Image detector	GATAN K3 (6k x 4k)	Depositor
Maximum map value	43.077	Depositor
Minimum map value	-28.271	Depositor
Average map value	0.003	Depositor
Map value standard deviation	2.155	Depositor
Recommended contour level	6.0	Depositor
Map size (Å)	544.0, 544.0, 544.0	wwPDB
Map dimensions	400, 400, 400	wwPDB
Map angles (°)	90.0, 90.0, 90.0	wwPDB
Pixel spacing (Å)	1.36, 1.36, 1.36	Depositor

For Manuscript Review

5 Model quality (i)

5.1 Standard geometry (i)

The Z score for a bond length (or angle) is the number of standard deviations the observed value is removed from the expected value. A bond length (or angle) with $|Z| > 5$ is considered an outlier worth inspection. RMSZ is the root-mean-square of all Z scores of the bond lengths (or angles).

Mol	Chain	Bond lengths		Bond angles	
		RMSZ	#$\|Z\|$ >5	RMSZ	#$\|Z\|$ >5
1	0	0.33	0/2728	0.56	0/3693
1	1	0.31	0/2728	0.53	0/3693
1	2	0.32	0/2728	0.54	0/3693
1	3	0.30	0/2701	0.53	0/3657
1	4	0.31	0/2701	0.54	0/3657
1	5	0.30	0/2701	0.53	0/3657
1	6	0.29	0/2728	0.55	0/3693
1	7	0.31	0/2728	0.55	0/3693
1	A	0.30	0/2728	0.54	0/3693
1	B	0.29	0/2728	0.53	0/3693
1	C	0.29	0/2728	0.55	0/3693
1	D	0.29	0/2728	0.53	0/3693
1	E	0.28	0/2728	0.55	0/3693
1	F	0.29	0/2728	0.53	0/3693
1	G	0.27	0/2728	0.51	0/3693
1	H	0.27	0/2728	0.52	0/3693
1	I	0.29	0/2728	0.55	0/3693
1	J	0.27	0/2728	0.51	0/3693
1	K	0.28	0/2728	0.54	0/3693
1	L	0.32	0/2728	0.57	0/3693
1	M	0.34	0/2728	0.56	0/3693
1	N	0.34	0/2701	0.55	0/3657
1	O	0.32	0/2728	0.55	0/3693
1	P	0.28	0/2728	0.54	0/3693
1	Q	0.30	0/2728	0.55	0/3693
1	R	0.28	0/2728	0.54	0/3693
1	S	0.30	0/2728	0.57	0/3693
1	T	0.31	0/2728	0.57	0/3693
1	g	0.37	0/2728	0.57	0/3693
1	h	0.38	0/2701	0.56	0/3657
2	U	0.29	0/3755	0.51	0/5072
2	V	0.31	0/3792	0.53	0/5125
2	W	0.30	0/3792	0.51	0/5125
2	X	0.31	0/3792	0.51	0/5125

For Manuscript Review

Mol	Chain	Bond lengths		Bond angles	
		RMSZ	#|Z| >5	RMSZ	#|Z| >5
2	Y	0.33	0/3792	0.52	0/5125
2	Z	0.30	0/3792	0.51	0/5125
2	a	0.28	0/3828	0.51	0/5174
2	b	0.31	0/3792	0.52	0/5125
2	c	0.35	0/3792	0.55	0/5125
2	d	0.30	0/3792	0.50	0/5125
2	e	0.30	0/3792	0.52	0/5125
2	f	0.29	0/3792	0.51	0/5125
All	All	0.31	0/127208	0.53	0/172106

There are no bond length outliers.

There are no bond angle outliers.

There are no chirality outliers.

There are no planarity outliers.

5.2 Too-close contacts (i)

Due to software issues we are unable to calculate clashes - this section is therefore empty.

5.3 Torsion angles (i)

5.3.1 Protein backbone (i)

In the following table, the Percentiles column shows the percent Ramachandran outliers of the chain as a percentile score with respect to all PDB entries followed by that with respect to all EM entries.

The Analysed column shows the number of residues for which the backbone conformation was analysed, and the total number of residues.

Mol	Chain	Analysed	Favoured	Allowed	Outliers	Percentiles	
1	0	338/341 (99%)	315 (93%)	22 (6%)	1 (0%)	41	75
1	1	338/341 (99%)	318 (94%)	18 (5%)	2 (1%)	25	63
1	2	338/341 (99%)	312 (92%)	23 (7%)	3 (1%)	17	55
1	3	335/341 (98%)	302 (90%)	31 (9%)	2 (1%)	25	63
1	4	335/341 (98%)	303 (90%)	31 (9%)	1 (0%)	41	75
1	5	335/341 (98%)	305 (91%)	28 (8%)	2 (1%)	25	63

Continued on next page...

For Manuscript Review

Continued from previous page...

Mol	Chain	Analysed	Favoured	Allowed	Outliers	Percentiles	
1	6	338/341 (99%)	311 (92%)	25 (7%)	2 (1%)	25	63
1	7	338/341 (99%)	313 (93%)	23 (7%)	2 (1%)	25	63
1	A	338/341 (99%)	320 (95%)	17 (5%)	1 (0%)	41	75
1	B	338/341 (99%)	321 (95%)	17 (5%)	0	100	100
1	C	338/341 (99%)	317 (94%)	20 (6%)	1 (0%)	41	75
1	D	338/341 (99%)	318 (94%)	19 (6%)	1 (0%)	41	75
1	E	338/341 (99%)	320 (95%)	18 (5%)	0	100	100
1	F	338/341 (99%)	318 (94%)	20 (6%)	0	100	100
1	G	338/341 (99%)	318 (94%)	20 (6%)	0	100	100
1	H	338/341 (99%)	318 (94%)	20 (6%)	0	100	100
1	I	338/341 (99%)	317 (94%)	20 (6%)	1 (0%)	41	75
1	J	338/341 (99%)	320 (95%)	18 (5%)	0	100	100
1	K	338/341 (99%)	313 (93%)	23 (7%)	2 (1%)	25	63
1	L	338/341 (99%)	313 (93%)	24 (7%)	1 (0%)	41	75
1	M	338/341 (99%)	311 (92%)	26 (8%)	1 (0%)	41	75
1	N	335/341 (98%)	306 (91%)	27 (8%)	2 (1%)	25	63
1	O	338/341 (99%)	315 (93%)	20 (6%)	3 (1%)	17	55
1	P	338/341 (99%)	308 (91%)	28 (8%)	2 (1%)	25	63
1	Q	338/341 (99%)	316 (94%)	21 (6%)	1 (0%)	41	75
1	R	338/341 (99%)	314 (93%)	22 (6%)	2 (1%)	25	63
1	S	338/341 (99%)	308 (91%)	28 (8%)	2 (1%)	25	63
1	T	338/341 (99%)	313 (93%)	23 (7%)	2 (1%)	25	63
1	g	338/341 (99%)	315 (93%)	22 (6%)	1 (0%)	41	75
1	h	335/341 (98%)	304 (91%)	28 (8%)	3 (1%)	17	55
2	U	462/533 (87%)	447 (97%)	15 (3%)	0	100	100
2	V	468/533 (88%)	449 (96%)	19 (4%)	0	100	100
2	W	468/533 (88%)	449 (96%)	19 (4%)	0	100	100
2	X	468/533 (88%)	444 (95%)	24 (5%)	0	100	100
2	Y	468/533 (88%)	448 (96%)	20 (4%)	0	100	100
2	Z	468/533 (88%)	453 (97%)	14 (3%)	1 (0%)	47	79
2	a	472/533 (89%)	455 (96%)	16 (3%)	1 (0%)	47	79

Continued on next page...

For Manuscript Review

Continued from previous page...

Mol	Chain	Analysed	Favoured	Allowed	Outliers	Percentiles	
2	b	468/533 (88%)	451 (96%)	17 (4%)	0	100	100
2	c	468/533 (88%)	449 (96%)	17 (4%)	2 (0%)	34	71
2	d	468/533 (88%)	452 (97%)	16 (3%)	0	100	100
2	e	468/533 (88%)	450 (96%)	18 (4%)	0	100	100
2	f	468/533 (88%)	450 (96%)	18 (4%)	0	100	100
All	All	15739/16626 (95%)	14799 (94%)	895 (6%)	45 (0%)	44	75

All (45) Ramachandran outliers are listed below:

Mol	Chain	Res	Type
1	2	334	GLU
1	4	316	ARG
1	5	316	ARG
1	C	316	ARG
1	L	262	VAL
1	N	316	ARG
1	O	177	THR
1	Q	262	VAL
1	R	262	VAL
1	R	313	ASP
1	S	262	VAL
1	S	313	ASP
1	T	262	VAL
1	h	316	ARG
1	1	157	SER
1	3	316	ARG
1	7	325	PRO
1	P	325	PRO
1	T	92	ARG
2	c	164	LEU
1	0	262	VAL
1	5	100	ASN
1	6	325	PRO
1	K	325	PRO
1	N	100	ASN
2	c	217	GLN
1	A	314	PRO
1	I	314	PRO
1	g	157	SER
1	h	100	ASN

Continued on next page...

For Manuscript Review

Continued from previous page...

Mol	Chain	Res	Type
1	1	262	VAL
1	2	262	VAL
1	M	262	VAL
1	O	325	PRO
2	a	301	GLN
1	h	315	ALA
1	2	157	SER
1	3	100	ASN
1	7	201	ASP
1	K	201	ASP
1	6	201	ASP
1	O	201	ASP
1	P	201	ASP
2	Z	215	MET
1	D	314	PRO

5.3.2 Protein sidechains (i)

In the following table, the Percentiles column shows the percent sidechain outliers of the chain as a percentile score with respect to all PDB entries followed by that with respect to all EM entries.

The Analysed column shows the number of residues for which the sidechain conformation was analysed, and the total number of residues.

Mol	Chain	Analysed	Rotameric	Outliers	Percentiles	
1	0	289/290 (100%)	288 (100%)	1 (0%)	92	95
1	1	289/290 (100%)	289 (100%)	0	100	100
1	2	289/290 (100%)	288 (100%)	1 (0%)	92	95
1	3	286/290 (99%)	286 (100%)	0	100	100
1	4	286/290 (99%)	286 (100%)	0	100	100
1	5	286/290 (99%)	286 (100%)	0	100	100
1	6	289/290 (100%)	289 (100%)	0	100	100
1	7	289/290 (100%)	287 (99%)	2 (1%)	84	90
1	A	289/290 (100%)	288 (100%)	1 (0%)	92	95
1	B	289/290 (100%)	288 (100%)	1 (0%)	92	95
1	C	289/290 (100%)	289 (100%)	0	100	100
1	D	289/290 (100%)	287 (99%)	2 (1%)	84	90

Continued on next page...

For Manuscript Review

Continued from previous page...

Mol	Chain	Analysed	Rotameric	Outliers	Percentiles	
1	E	289/290 (100%)	288 (100%)	1 (0%)	92	95
1	F	289/290 (100%)	288 (100%)	1 (0%)	92	95
1	G	289/290 (100%)	289 (100%)	0	100	100
1	H	289/290 (100%)	288 (100%)	1 (0%)	92	95
1	I	289/290 (100%)	288 (100%)	1 (0%)	92	95
1	J	289/290 (100%)	288 (100%)	1 (0%)	92	95
1	K	289/290 (100%)	289 (100%)	0	100	100
1	L	289/290 (100%)	286 (99%)	3 (1%)	76	86
1	M	289/290 (100%)	287 (99%)	2 (1%)	84	90
1	N	286/290 (99%)	284 (99%)	2 (1%)	84	90
1	O	289/290 (100%)	288 (100%)	1 (0%)	92	95
1	P	289/290 (100%)	289 (100%)	0	100	100
1	Q	289/290 (100%)	288 (100%)	1 (0%)	92	95
1	R	289/290 (100%)	288 (100%)	1 (0%)	92	95
1	S	289/290 (100%)	289 (100%)	0	100	100
1	T	289/290 (100%)	286 (99%)	3 (1%)	76	86
1	g	289/290 (100%)	287 (99%)	2 (1%)	84	90
1	h	286/290 (99%)	286 (100%)	0	100	100
2	U	377/428 (88%)	377 (100%)	0	100	100
2	V	380/428 (89%)	377 (99%)	3 (1%)	81	89
2	W	380/428 (89%)	380 (100%)	0	100	100
2	X	380/428 (89%)	379 (100%)	1 (0%)	92	95
2	Y	380/428 (89%)	380 (100%)	0	100	100
2	Z	380/428 (89%)	380 (100%)	0	100	100
2	a	383/428 (90%)	383 (100%)	0	100	100
2	b	380/428 (89%)	378 (100%)	2 (0%)	88	93
2	c	380/428 (89%)	379 (100%)	1 (0%)	92	95
2	d	380/428 (89%)	380 (100%)	0	100	100
2	e	380/428 (89%)	380 (100%)	0	100	100
2	f	380/428 (89%)	379 (100%)	1 (0%)	92	95
All	All	13215/13836 (96%)	13179 (100%)	36 (0%)	92	95

For Manuscript Review

All (36) residues with a non-rotameric sidechain are listed below:

Mol	Chain	Res	Type
1	0	36	THR
1	2	333	ASP
1	7	199	VAL
1	7	201	ASP
1	A	139	LYS
1	B	13	ASN
1	D	139	LYS
1	D	310	THR
1	E	116	MET
1	F	201	ASP
1	H	29	ARG
1	I	148	ASP
1	J	29	ARG
1	L	92	ARG
1	L	93	LEU
1	L	201	ASP
1	M	333	ASP
1	M	334	GLU
1	N	103	ASP
1	N	296	GLU
1	O	119	GLU
1	Q	90	LEU
1	R	91	ARG
1	T	91	ARG
1	T	96	GLU
1	T	311	THR
2	V	178	SER
2	V	179	ASN
2	V	183	THR
2	X	72	ASN
2	b	156	THR
2	b	161	SER
2	c	211	TYR
2	f	95	ARG
1	g	128	GLU
1	g	258	VAL

Sometimes sidechains can be flipped to improve hydrogen bonding and reduce clashes. All (25) such sidechains are listed below:

Mol	Chain	Res	Type
1	0	164	GLN

Continued on next page...

For Manuscript Review

Continued from previous page...

Mol	Chain	Res	Type
1	1	289	GLN
1	3	299	ASN
1	5	8	GLN
1	5	299	ASN
1	7	130	GLN
1	7	164	GLN
1	C	115	ASN
1	D	161	ASN
1	K	130	GLN
1	L	115	ASN
1	N	82	HIS
1	N	115	ASN
1	O	130	GLN
1	Q	125	GLN
2	U	376	ASN
2	Y	376	ASN
2	Y	407	GLN
2	c	244	GLN
2	d	407	GLN
2	e	270	GLN
2	f	244	GLN
1	g	130	GLN
1	h	190	ASN
1	h	299	ASN

5.3.3 RNA (i)

There are no RNA molecules in this entry.

5.4 Non-standard residues in protein, DNA, RNA chains (i)

There are no non-standard protein/DNA/RNA residues in this entry.

5.5 Carbohydrates (i)

There are no monosaccharides in this entry.

5.6 Ligand geometry (i)

There are no ligands in this entry.

For Manuscript Review

5.7 Other polymers (i)

There are no such residues in this entry.

5.8 Polymer linkage issues (i)

There are no chain breaks in this entry.

For Manuscript Review

6 Map visualisation (i)

This section contains visualisations of the EMDB entry EMD-36848. These allow visual inspection of the internal detail of the map and identification of artifacts.

Images derived from a raw map, generated by summing the deposited half-maps, are presented below the corresponding image components of the primary map to allow further visual inspection and comparison with those of the primary map.

6.1 Orthogonal projections (i)

6.1.1 Primary map

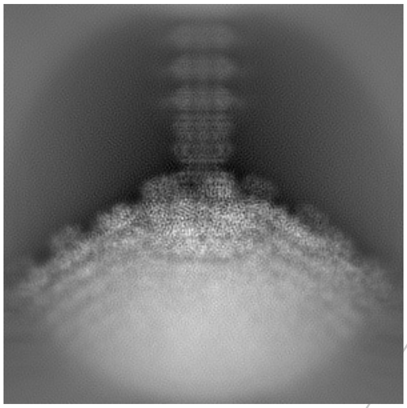
X

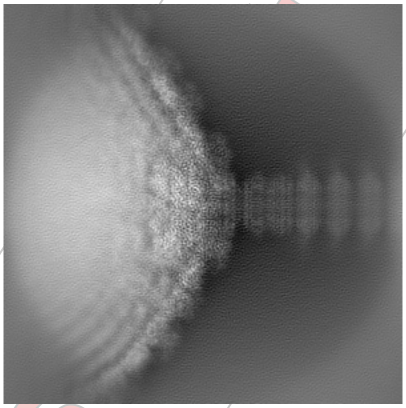
Y

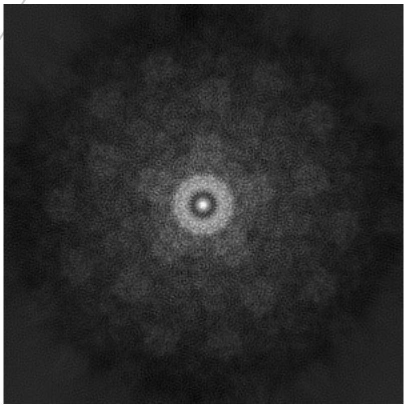
Z

6.1.2 Raw map

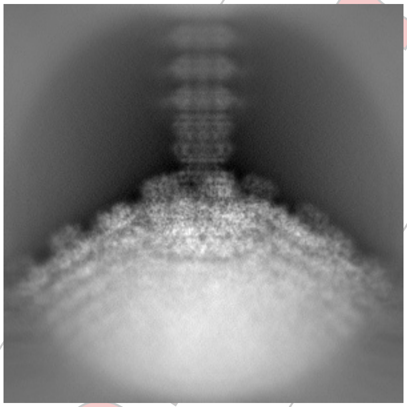
X

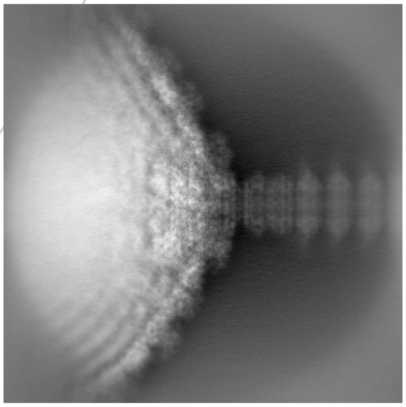
Y

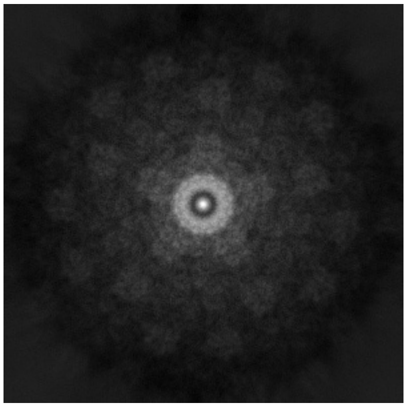
Z

The images above show the map projected in three orthogonal directions.

For Manuscript Review

6.2 Central slices (i)

6.2.1 Primary map

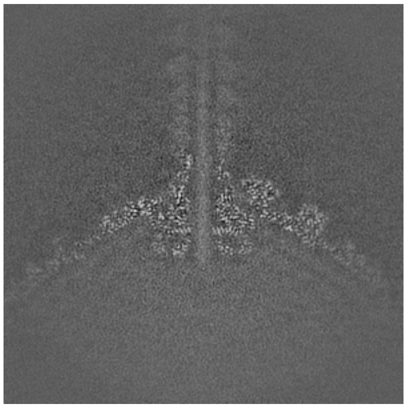
X Index: 200

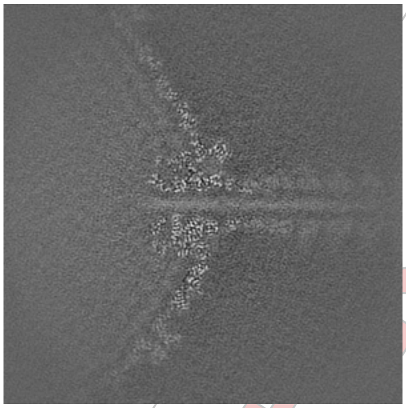
Y Index: 200

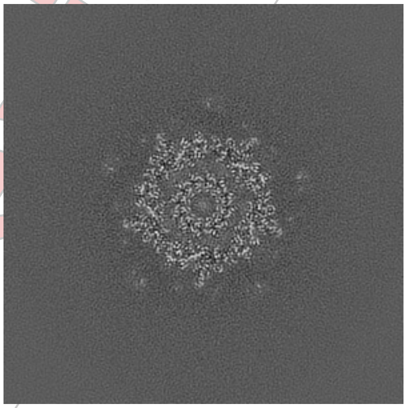
Z Index: 200

6.2.2 Raw map

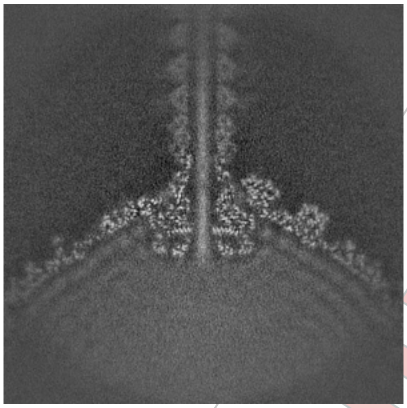
X Index: 200

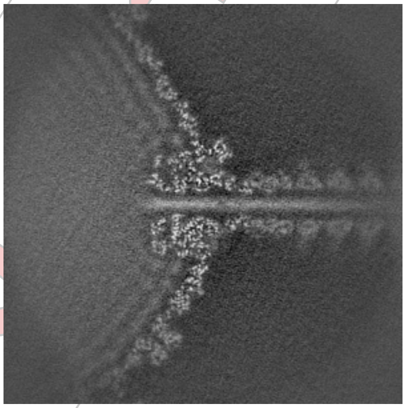
Y Index: 200

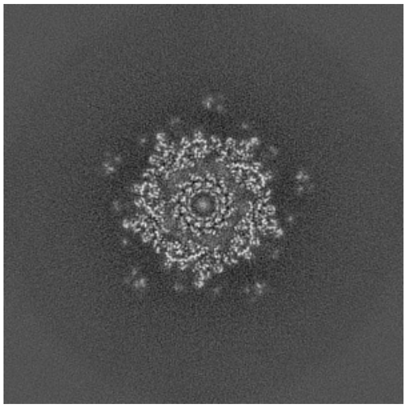
Z Index: 200

The images above show central slices of the map in three orthogonal directions.

For Manuscript Review

6.3 Largest variance slices (i)

6.3.1 Primary map

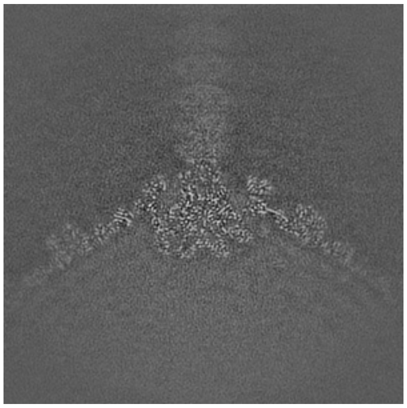
X Index: 216

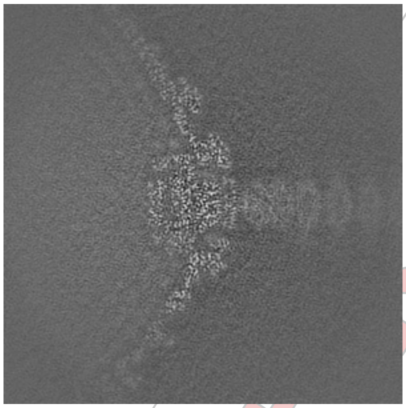
Y Index: 219

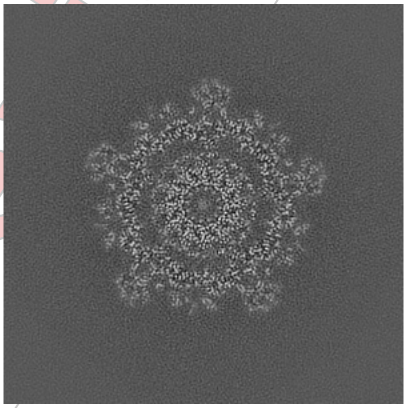
Z Index: 186

6.3.2 Raw map

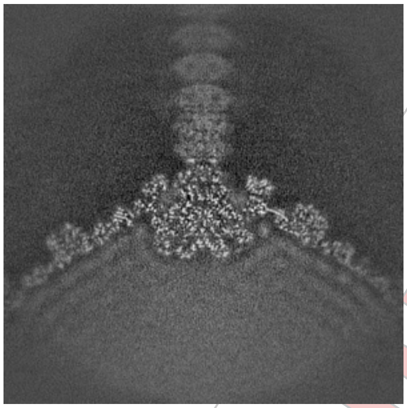
X Index: 216

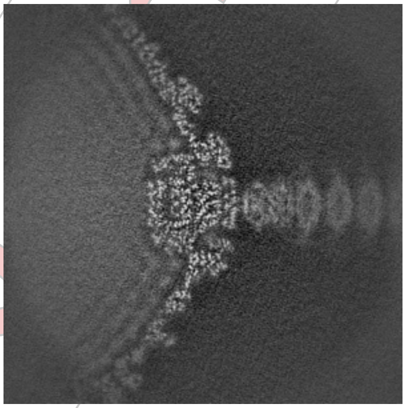
Y Index: 219

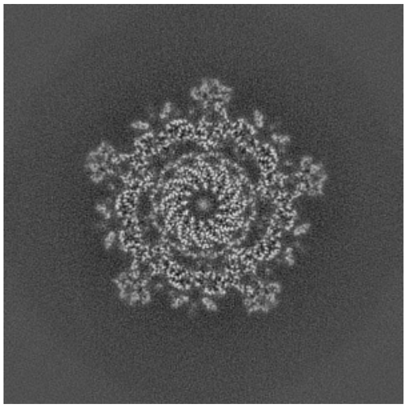
Z Index: 187

The images above show the largest variance slices of the map in three orthogonal directions.

For Manuscript Review

6.4 Orthogonal standard-deviation projections (False-color) (i)

6.4.1 Primary map

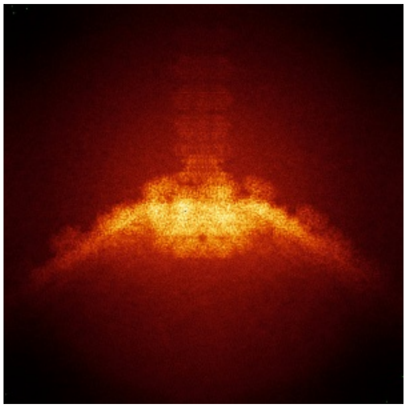
X

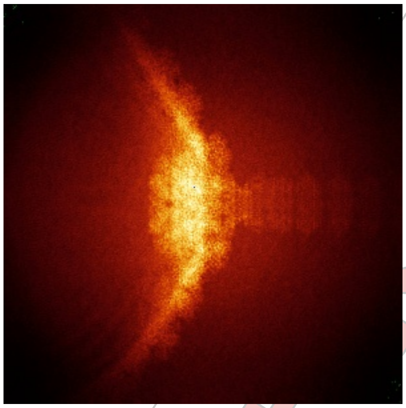
Y

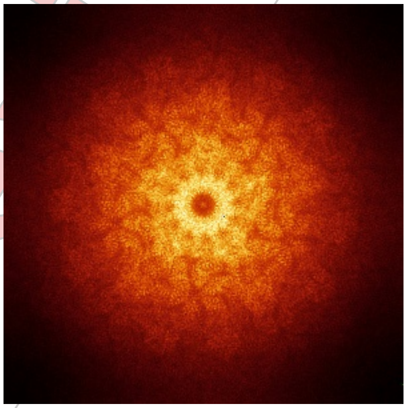
Z

6.4.2 Raw map

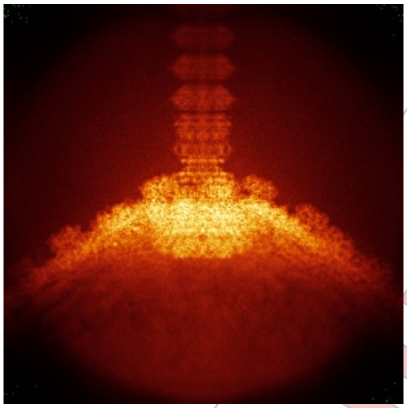
X

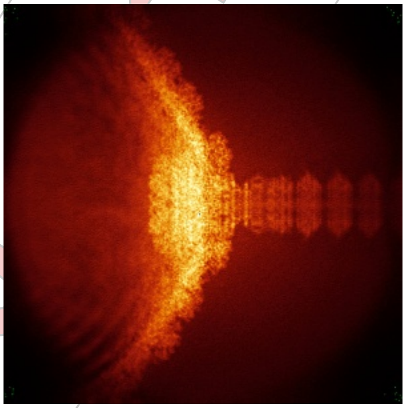
Y

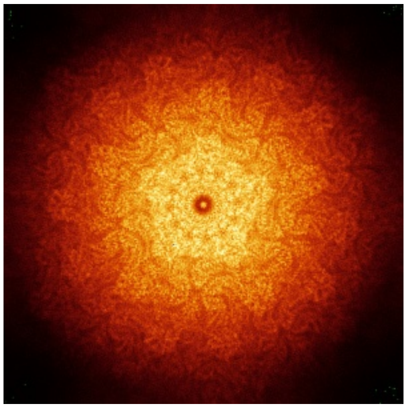
Z

The images above show the map standard deviation projections with false color in three orthogonal directions. Minimum values are shown in green, max in blue, and dark to light orange shades represent small to large values respectively.

For Manuscript Review

6.5 Orthogonal surface views (i)

6.5.1 Primary map

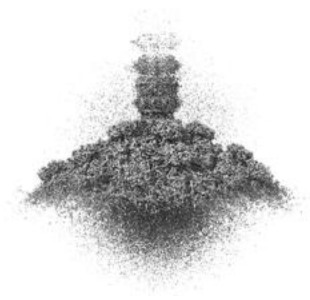

X

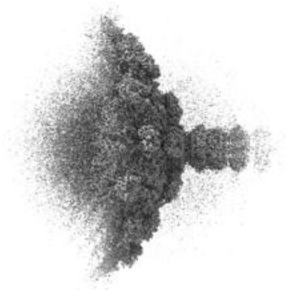

Y

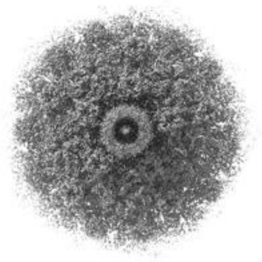

Z

The images above show the 3D surface view of the map at the recommended contour level 6.0. These images, in conjunction with the slice images, may facilitate assessment of whether an appropriate contour level has been provided.

6.5.2 Raw map

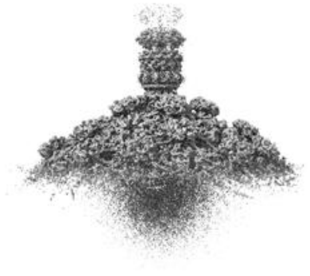

X

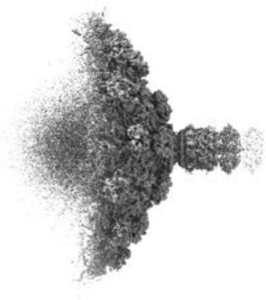

Y

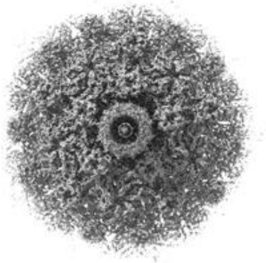

Z

These images show the 3D surface of the raw map. The raw map's contour level was selected so that its surface encloses the same volume as the primary map does at its recommended contour level.

6.6 Mask visualisation (i)

This section was not generated. No masks/segmentation were deposited.

For Manuscript Review

7 Map analysis (i)

This section contains the results of statistical analysis of the map.

7.1 Map-value distribution (i)

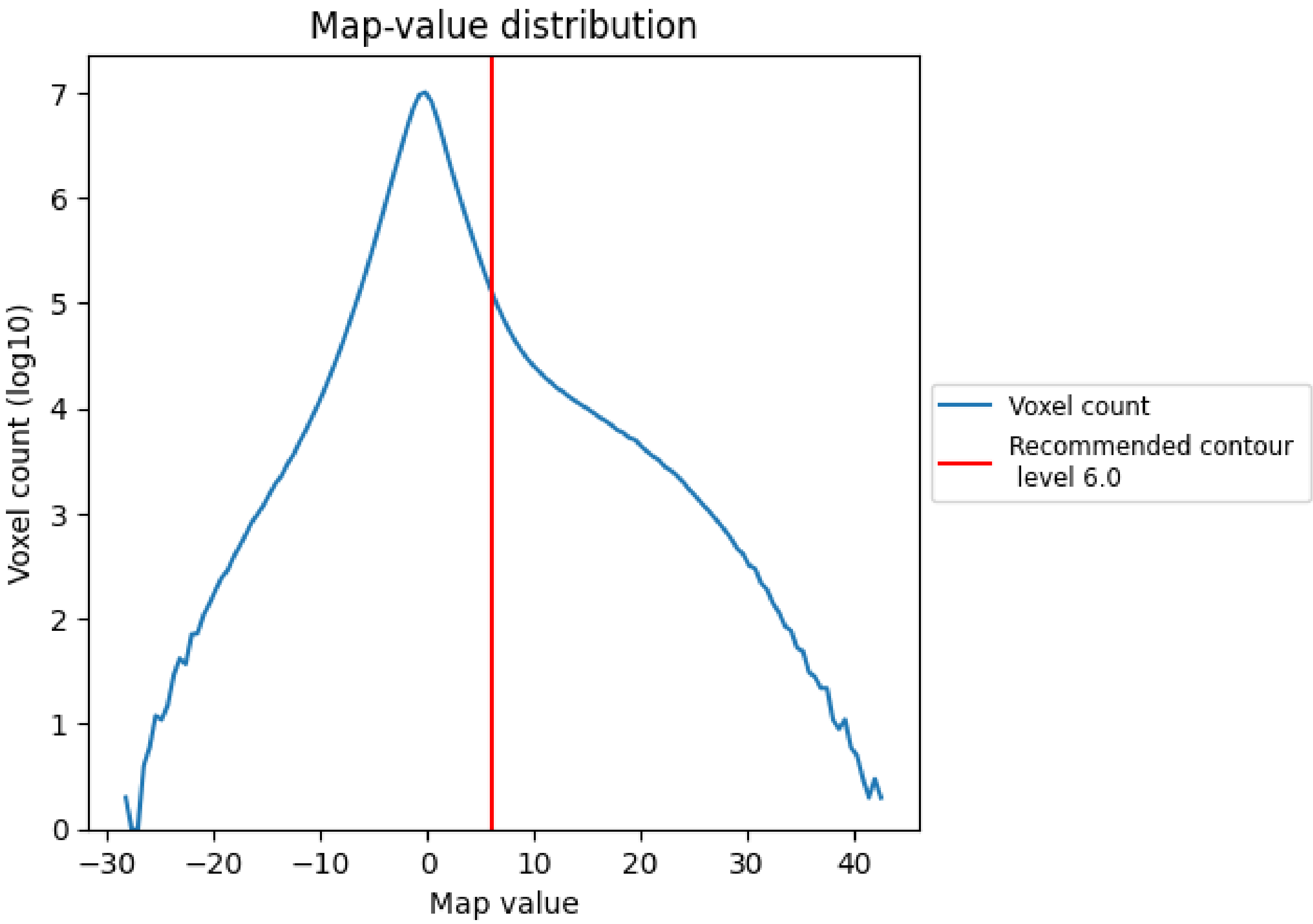

The map-value distribution is plotted in 128 intervals along the x-axis. The y-axis is logarithmic. A spike in this graph at zero usually indicates that the volume has been masked.

For Manuscript Review

7.2 Volume estimate (i)

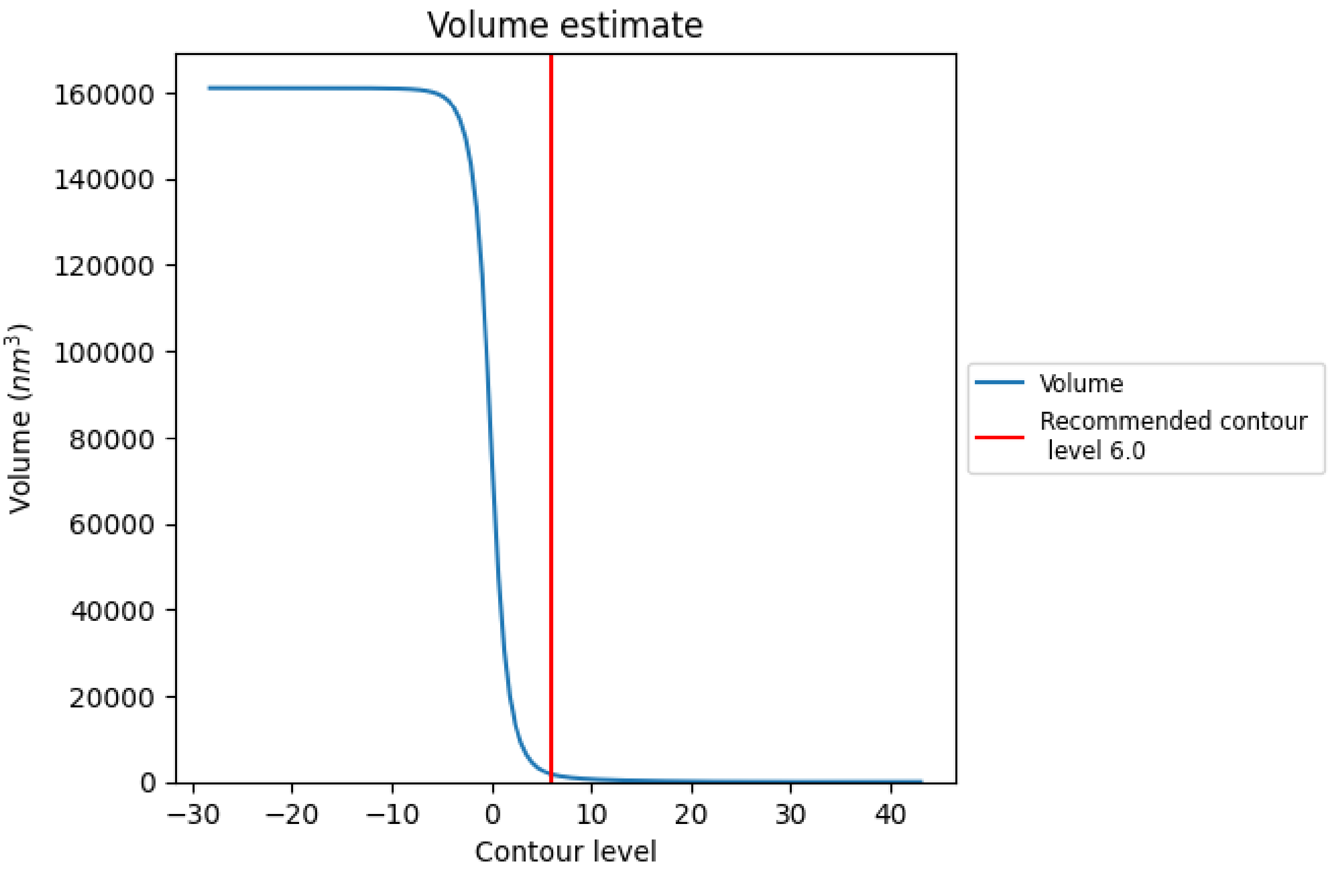

The volume at the recommended contour level is 1805 nm^3; this corresponds to an approximate mass of 1631 kDa.

The volume estimate graph shows how the enclosed volume varies with the contour level. The recommended contour level is shown as a vertical line and the intersection between the line and the curve gives the volume of the enclosed surface at the given level.

For Manuscript Review

7.3 Rotationally averaged power spectrum (i)

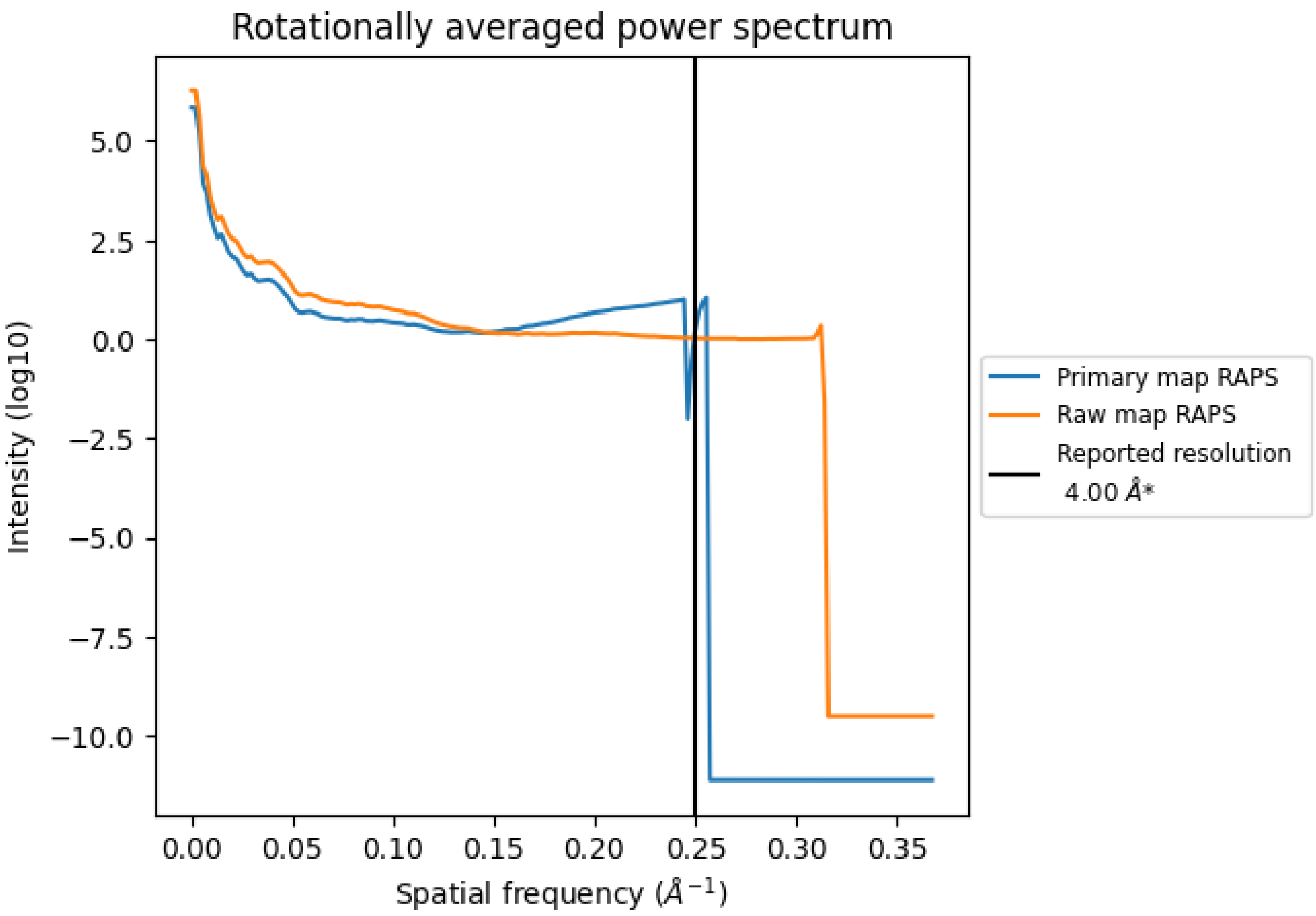

*Reported resolution corresponds to spatial frequency of 0.250 Å^{-1}

For Manuscript Review

8 Fourier-Shell correlation (i)

Fourier-Shell Correlation (FSC) is the most commonly used method to estimate the resolution of single-particle and subtomogram-averaged maps. The shape of the curve depends on the imposed symmetry, mask and whether or not the two 3D reconstructions used were processed from a common reference. The reported resolution is shown as a black line. A curve is displayed for the half-bit criterion in addition to lines showing the 0.143 gold standard cut-off and 0.5 cut-off.

8.1 FSC (i)

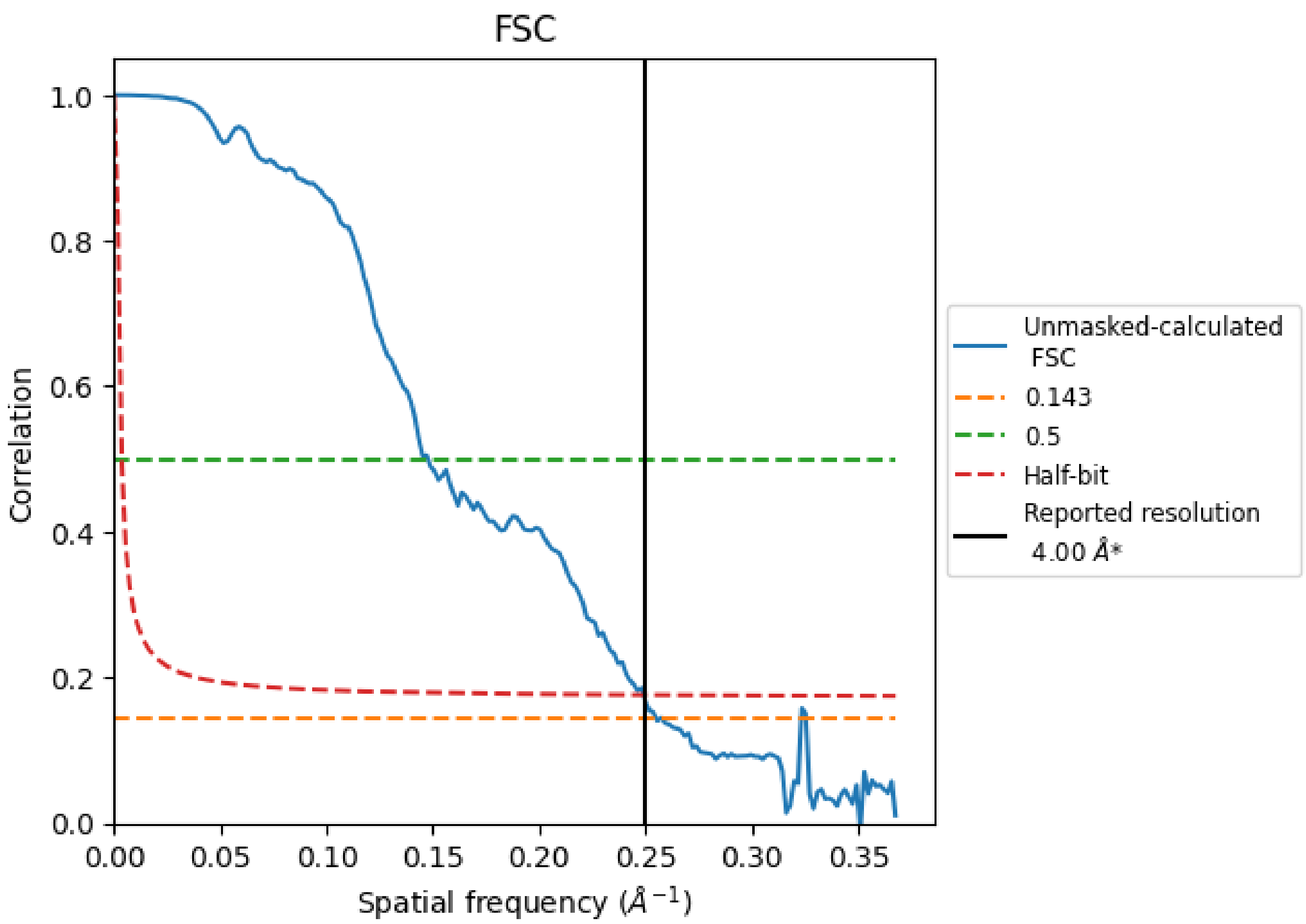

*Reported resolution corresponds to spatial frequency of 0.250 $Å^{-1}$

For Manuscript Review

8.2 Resolution estimates ⓘ

Resolution estimate (Å)	Estimation criterion (FSC cut-off)		
	0.143	0.5	Half-bit
Reported by author	4.00	-	-
Author-provided FSC curve	-	-	-
Unmasked-calculated*	3.92	6.78	4.01

*Resolution estimate based on FSC curve calculated by comparison of deposited half-maps.

For Manuscript Review

9 Map-model fit (i)

This section contains information regarding the fit between EMDB map EMD-36848 and PDB model 8K39. Per-residue inclusion information can be found in section 3 on page 8.

9.1 Map-model overlay (i)

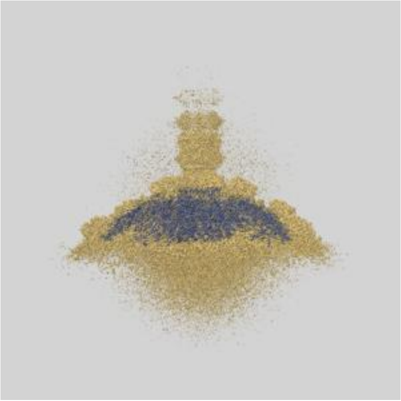

X

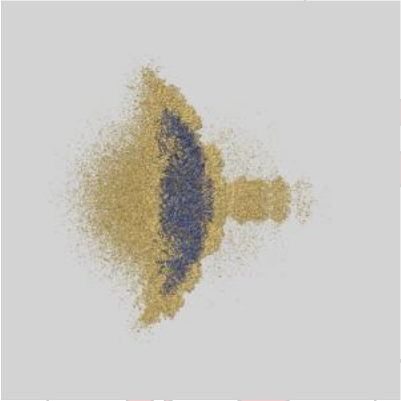

Y

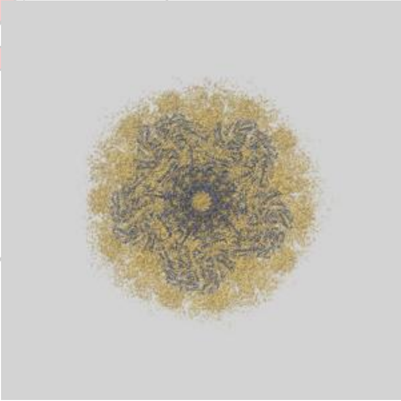

Z

The images above show the 3D surface view of the map at the recommended contour level 6.0 at 50% transparency in yellow overlaid with a ribbon representation of the model coloured in blue. These images allow for the visual assessment of the quality of fit between the atomic model and the map.

For Manuscript Review

9.2 Q-score mapped to coordinate model (i)

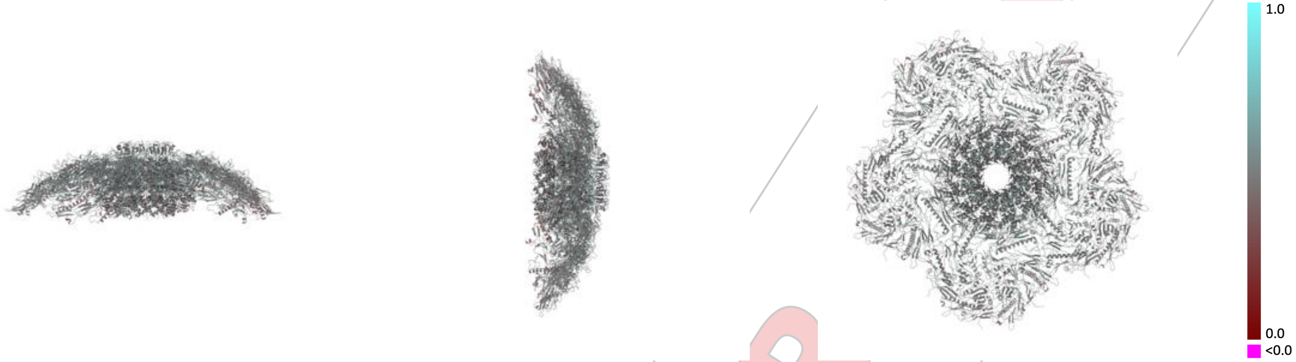

X Y Z

The images above show the model with each residue coloured according its Q-score. This shows their resolvability in the map with higher Q-score values reflecting better resolvability. Please note: Q-score is calculating the resolvability of atoms, and thus high values are only expected at resolutions at which atoms can be resolved. Low Q-score values may therefore be expected for many entries.

9.3 Atom inclusion mapped to coordinate model (i)

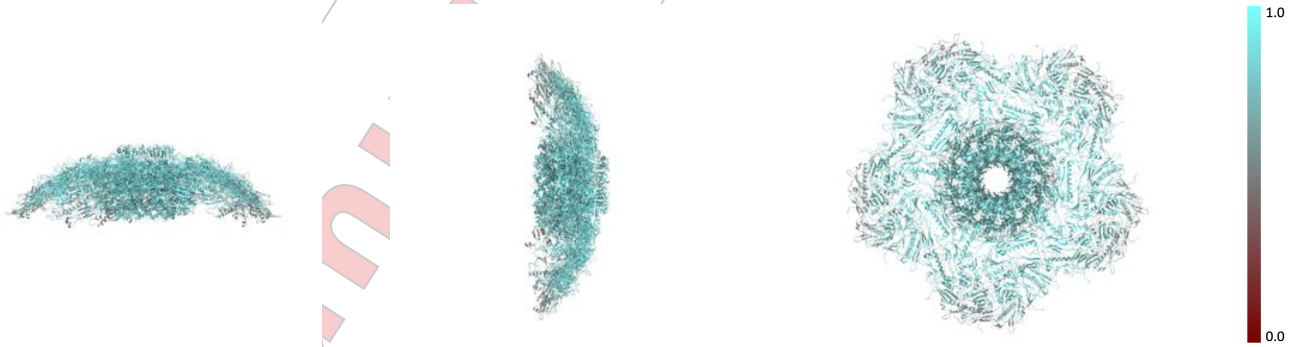

X Y Z

The images above show the model with each residue coloured according to its atom inclusion. This shows to what extent they are inside the map at the recommended contour level (6.0).

For Manuscript Review

9.4 Atom inclusion ⓘ

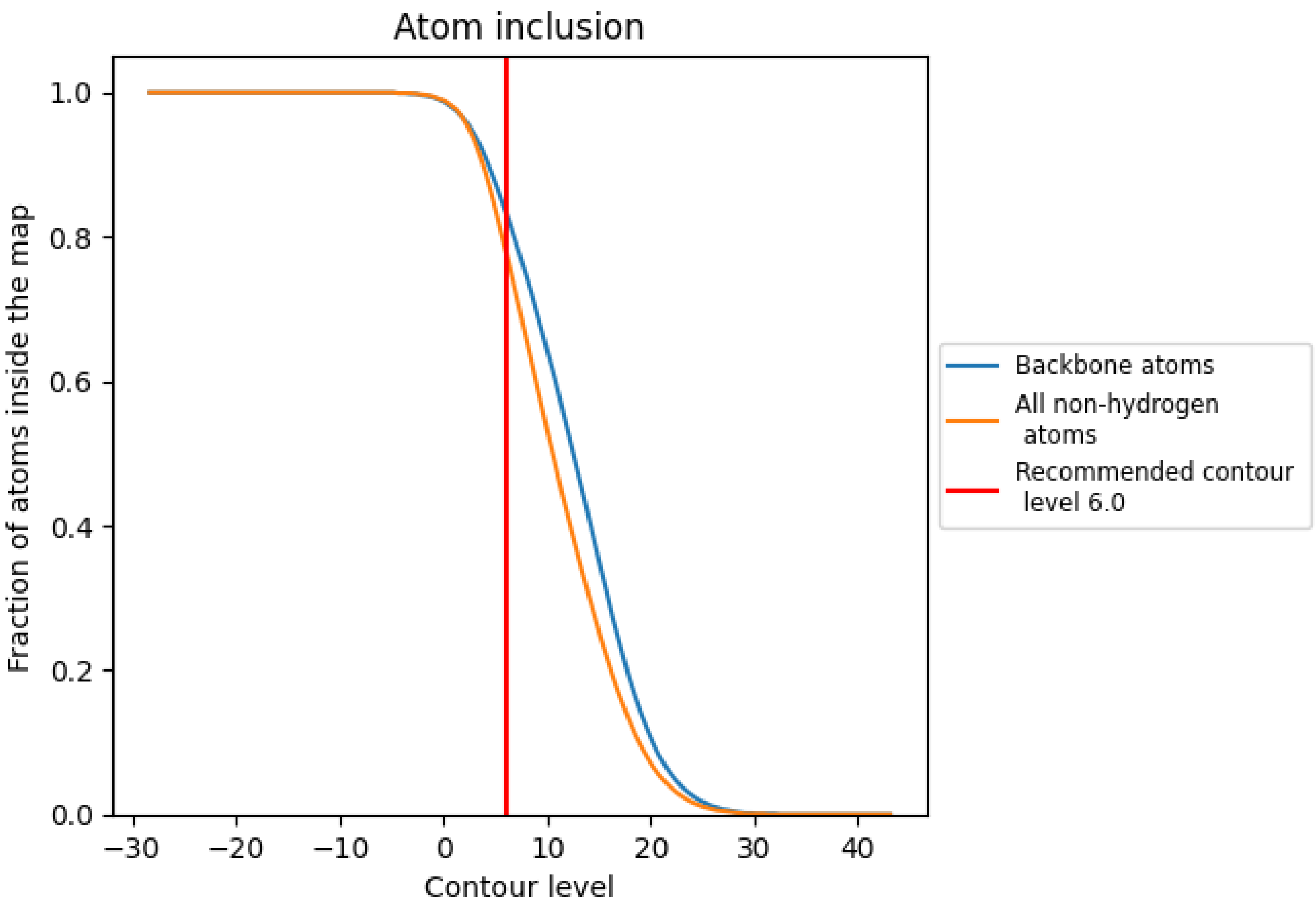

At the recommended contour level, 84% of all backbone atoms, 78% of all non-hydrogen atoms, are inside the map.

For Manuscript Review

9.5 Map-model fit summary (i)

The table lists the average atom inclusion at the recommended contour level (6.0) and Q-score for the entire model and for each chain.

Chain	Atom inclusion	Q-score
All	0.7820	0.4900
0	0.8470	0.5080
1	0.8330	0.5030
2	0.8360	0.5030
3	0.8340	0.5030
4	0.8320	0.5040
5	0.8220	0.4970
6	0.6990	0.4810
7	0.6870	0.4720
A	0.7760	0.4930
B	0.7730	0.4950
C	0.7670	0.4930
D	0.7660	0.4920
E	0.6500	0.4740
F	0.6440	0.4690
G	0.6410	0.4690
H	0.6370	0.4690
I	0.7740	0.4900
J	0.6500	0.4680
K	0.6920	0.4780
L	0.8020	0.4930
M	0.8390	0.5080
N	0.8270	0.5010
O	0.6940	0.4750
P	0.6870	0.4780
Q	0.8060	0.4910
R	0.8050	0.4900
S	0.8100	0.4940
T	0.8020	0.4930
U	0.8140	0.4930
V	0.8100	0.4870
W	0.8120	0.4880
X	0.8180	0.4960
Y	0.8220	0.4940
Z	0.8220	0.4940

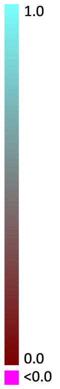

Continued on next page...

For Manuscript Review

Continued from previous page...

Chain	Atom inclusion	Q-score
a	0.8190	0.4870
b	0.8190	0.4890
c	0.8120	0.4860
d	0.8140	0.4880
e	0.8090	0.4860
f	0.8110	0.4880
g	0.8330	0.5030
h	0.8410	0.5050

For Manuscript Review
