# Supplementary material for: Structure of the siphophage neck–Tail complex suggests that conserved tail tip proteins facilitate receptor binding and tail assembly
Source: PLoS Biol. 2023 Dec 14;21(12):e3002441. doi: 10.1371/journal.pbio.3002441 (PMC10721106; doi:10.1371/journal.pbio.3002441)
Supplement: Attachment — Submitted filename: ResponseToReviewers.docx [file pbio.3002441.s022.docx]

We would like to thank the reviewers for their critical review of our work and for their constructive suggestions on how to improve the manuscript. The comments and suggestions have been addressed, and the manuscript has been revised accordingly. To make the responses easy to follow, we have copied the original comments in black, and marked our responses in blue. The page and line numbers correspond to those in the revised article with changes highlighted.

**Reviewer #1:**

The manuscript "In situ structure of the connector and tail of a Siphophage" by Hao Xiao et al describes the structure of the phage lambda connector-tail complex in the mature virion by cryo-electron microscopy. The composite structure was assembled from the capsid with icosahedral symmetry imposed, the portal-connecter region with C12 symmetry, the tail tube using C6 symmetry, and the tail tip with C3 symmetry. This is a sensible strategy as the flexibility of the tail tube precludes solving phage particles in toto, while the head and tail-tip that bracket the tail tube are largely rigid. The structural data presented appear to be quite comprehensive with most of the known protein components partially or fully resolved, except where there is excess flexibility such as the very tail tip and much of the tape-measure protein. The quality of density appears to be sufficient for the atomic models generated although only small regions of fitting can be shown in Figures. As the density maps are not available to the reviewer, grey-scale sections through the structures would be useful for assessing noise, artefacts and overall quality - the only section is in Fig S7a through the tail tube - and (having been burnt before by papers from other groups) I insist on seeing such data before giving a recommendation for publication. In addition, a section through the putative iron atom (lines 247-250) should show significant density due to the high scattering power of iron, and would be an addition to Figure 4F. Nonetheless, this looks overall like a structural tour-de-force, especially for detailing the complex portal-connection organization and especially the tail-tip. What it lacks is a functional payoff from this new structural data, and while something of this sort is attempted in the Supplemental Material, it is inadequate and a better attempt should be added at the end of the main text. If space is a consideration, I suggest shortening the structural details by focusing on the more significant findings in order to allow several paragraphs of conclusions. This is not a recommendation for new experimental data but for significant improvements in the presentation.

We present four gray-scale sections of the structures as follows. Panels A and B are sections of the tail tip. The features in the red circles are iron-sulfur clusters. Panel C is a section of the portal and rings of the adaptor, stopper, and tail terminator. The feature in the yellow circle is an α-helix. Panel D is a section of tail tube. We have added the central section of the iron-binding site to the new Fig. 7F.

A	B	C	D

We have enhanced the manuscript by adding structural comparisons between the tail tips of lambda and other long-tail phages or tail-like machines. Our results suggest that their tail tip contains conserved domains that facilitate tail assembly, receptor binding, cell adsorption, and DNA retaining/releasing. It is intriguing that these domains are distributed in different tail tip proteins in different phages or tail-like machines (see the section “Structure of the tail tip complex” in the revised manuscript for details).

The structural similarity between the tail tips of lambda and T5 (Linares et al. *Structural basis of bacteriophage T5 infection trigger and E. coli cell wall perforation*. Science Advances. 2023;9) suggests that upon the receptor interaction, the tail tip of lambda would undergo structural changes similar to that of T5. The tail tips of lambda and T5 share common domains of HDI–IV, HDII-insertion, plug, and two fibronectins (FNIIIs). For T5, all these domains are located in the baseplate hub protein pb3. By contrast, for lambda, the HDI is located in gpL; the HDII, HDIII, HDIV, two FNIIIs, and the C-terminal region of HDII-insertion are located in gpJ; the N-terminal region of HDII-insertion is located in gpL; and the plug is located in gpI. It is noteworthy that gene I is located between genes L and J, a gene location that corresponds to the position of the T5 pb3 plug domain, except for an inserted gene K (see the new Fig. S9). Therefore, we suggest that gpL, gpI, and the N-terminal region of gpJ (from HDII to FNIII-1 and -2) together correspond to T5 BHP pb3. In addition, the central fiber protein (pb4) and receptor binding protein (pb5) of T5 correspond to the central fiber domain and receptor binding domain of lambda gpJ, respectively.

Specific concerns and considerations:

1. The term "connecter" is used to describe the portal gp8, "adapter" gpW, and "stopper" gpFII, but this is not correct. The portal is separate from the connector, being part of the capsid and involved in DNA packaging, while the connecter is a region that joins the portal to the tail after packaging is complete. Thus, it would be more correct to describe the major components as the capsid, portal, connector, tail tube and tail-tip, and in particular, the parts presented here are the portal, connector and tail (implicitly including the tail tube and tail-tip). This should be reflected in the title and throughout the manuscript. For example, line 77:

The portal combines two rings of adaptor and stopper proteins to form the head-to-tail connector

This does not properly recognize the portal as a separate and critical component that functions not only in packaging and releasing DNA, but likely nucleates capsid assembly.

We thank the reviewer for pointing this out. Reviewer 2 also pointed out the same problem and suggested that we use “neck (connector)” to describe the adaptor and stopper. We have revised the manuscript accordingly.

2. Side-tail fibers are mentioned in passing (line 78, 229), but should be visible in the tail-tip structure, even as truncated knobs that most lambda lab strains include. Four side-tail fibers densities should be observed bound to gpL but none are noted. This is especially interesting in the context of the model that includes 4 trimers of the hub protein, gpL, which accounts for an apparent symmetry mismatch between a hexameric tail/trimeric tip to which the 4 side tail fibers bind.

We thank the reviewer for pointing out the absence of the side tail fiber in our structure. Reviewer 2 also raised the same question. The lambda phage we used is a laboratory strain and is called “lambda wild type” (Hendrix RW, Duda RL. Bacteriophage lambda PaPa: not the mother of all lambda phages. Science. 1992;258:1145-8). It lacks side tail fiber proteins (stf and tfa) as a result of a laboratory-acquired mutation. The lack of the side fiber proteins was further confirmed by our recent mass spectrometry experiment (page 6, lines 139-149; page 17, lines 473-478).

We have revised our manuscript accordingly.

3. Line 96: Our structure of the head is essentially identical to that reported by a recent study on the lambda head [32] except that our structure has higher resolution. This difference - 3.8A vs 3.5A - is really not worthy of comment. I suggest removing "except that our structure has higher resolution" and instead point out any significant improvements with biological meaning, if any (none are described).

We have deleted the line “except that our structure has higher resolution.” Both icosahedral structures of the lambda head are essentially identical (see the figures below).

*Top left: superimposition of our model of gpE on our density map (3.5Å resolution). Right: superimposition of model of gpE (7vik) by Wang et al. on their density map (by using local refinement, 3.7Å resolution, EMD-32011).*

*Bottom Left: superimposition of our gpE model (magenta) with the model by Wang et al. (cyan, PDB ID: 7vik). Bottom right: superimposition of our gpD model (magenta) with the model by Wang et al. (cyan, PDB ID: 7vik).*

4. While this work is an advance on previous capsid and tail tube structures, and cites them, it doesn't provide any context about how the new structures differ from them, and in particular, what new functional insights are provided. More attention is given to comparison with existing NMR structures of monomers, or domains, which are of less relevance being unassembled in solution.

We have added the structural comparison between the tail tip complexes of lambda and other long-tail phages (including T5) or tail-like machines (see the section “Structure of the tail tip complex” (page 10) in the revised manuscript for details.

5. The argument (lines 208-214) that the dsDNA extends down the tail to the 3-4 gpV rings, where it meets the gpH tape measure polymer, is based on the internal tube density transitioning from hollow (gpH) to solid (dsDNA). However, density along a symmetry axis (the tail tube) is notoriously noisy, and one wonders if this is in fact where the portal-connect structure is joined to the separately-calculated tail tube structure. Additional evidence is needed to support this density assignment. Further, the tape-measure protein defines the length of the tail tube - how can it do this if it ends short of the tail tube itself? What then is governing the tail tube length? The other ideas raised, that the gpH-dsDNA interaction favors delivery and allows a release signal to propagate from the tail tip through the tape-measure protein, do not require DNA to spill into the tail tube to make this interaction if the gpH is as long as the tail tube. This paragraph should be re-thought or omitted.

We agree with the reviewer and have deleted this part (page 10, lines 260-274).

6. Use of "Megatron" for the gpJ protein is new to me - I don't think this is in common use. The source seems to be ref [37] from 2020 which appropriated it from a character in the Transformers franchise, but the wording in this 2020 paper is unclear as to whether "Megatron" refers to one large multidomain protein, or several. Thus, I am also confused by line 242 that refers to Megatron in the context of gpL, which is not the "Megatron protein (gpJ)" labeled on Figure 1c. In addition, there is a completely different phage with this name - Mycobacterium phage Megatron - so I suggest "Megatron" should be dropped to avoid contributing further confusion.

We have renamed the gpJ as “central fiber protein” according to the nomenclature of lambda gpJ in a review (Davidson AR et al., 2012. Long Noncontractile Tail Machines of Bacteriophages. Adv Exp Med Biol 726).

We have revised the manuscript and figures accordingly.

7. There is an attempt at functional insights that derive from the new structure in the Supplementary information. In principle, this should be the conclusions of the main text so it's odd to read it here. However, its focus is on assembly when the paper has determined only the end product of assembly and has little to say about the steps to accomplish this. Thus, the assembly itself is inferred from other work, and much is speculative, vague and not novel. The order of accreting subunits isn't demonstrated beyond what is obvious - the head and tail assemble independently, packaging precedes binding of the head completion proteins (connector), and the tail binds to the head.

We have deleted the supplementary information.

8. General comments.

We thank the reviewer for helping us improve our manuscript.

Grammar could be improved in numerous places and some awkward phrasing re-thought - the authors should pay careful attention to what they write. Frequent repetition can also be avoided - for example, this redundancy in line 41:

  During the packaging of the genome, double-stranded DNA (dsDNA) is packaged into the head…

We have deleted it (page 3, line 53).

And an example of mis-wording, on line 75 - the vertex is not replaced, nothing is replaced, instead one vertex is *occupied* by the portal:

  One vertex of the icosahedron is replaced by a dodecameric portal,…

We have revised it (page 4, lines 95-96).

This makes no sense, although I understand the intent (line 240):

  Three copies of gpL and three copies of gpJ, surrounded alternately forming the core of the trimeric conical tail tip.

We have deleted it (page 11, lines 295-296).

**Reviewer #2:**

The MS "In situ structure of the connector and tail of a Siphophage" describes the atomic structure of bacteriophage lambda, namely the "wild type" lambda or the fiberless laboratory strain, as determined by cryo-electron microscopy. The authors report the composition of the virion, the copy number of most component proteins, and the nature of interaction between some components of the virus particle.

Despite containing a wealth of atomic-level information and contrary to the authors' statement (the last sequence of the Abstract), the MS provides little insight into the infection mechanism or assembly of the lambda particle for several reasons.

Infection mechanism.

"Wild type" (fiberless) lambda's infection process is well characterized functionally as lambda is a "model" phage for host receptor-induced DNA release. However, several processes remain poorly understood: a) how the interaction of the tail tip with the host LamB receptor opens the tube channel; b) structural transformation of the neck upon the initiation of DNA release; c) the function of side fibers and their role in the opening of the tube channel. Neither of these is studied in this paper.

We agree with the reviewer that we were unable to determine how the interaction of the tail tip with the host LamB receptor opens the tube channel and structural transformation of the neck upon the initiation of DNA release.

We have enhanced the manuscript by adding structural comparisons between the tail tips of lambda and other long-tail phages or tail-like machines. The structural similarity between the tail tips of lambda and T5 (Linares et al. *Structural basis of bacteriophage T5 infection trigger and E. coli cell wall perforation*. Science Advances. 2023;9) suggests that upon the receptor interaction, the tail tip of lambda would undergo structural changes similar to that of T5. The tail tips of lambda and T5 share the common domains of HDI–IV, HDII-insertion, plug, and two fibronectins (FNIIIs). For T5, all these domains are located in baseplate hub protein pb3. By contrast, for lambda, the HDI is located in gpL; the HDII, HDIII, HDIV, two FNIIIs, and the C-terminal region of HDII-insertion are located in gpJ; the N-terminal region of HDII-insertion is located in gpL; and the plug is located in gpI. In addition, the central fiber protein (pb4) and receptor binding protein (pb5) of T5 correspond to the central fiber domain and receptor binding domain of lambda gpJ, respectively.

Further structural comparisons between the tail tips of lambda and other long-tail phages or tail-like machines suggest that their tail tip contains conserved domains that facilitate tail assembly, receptor binding, cell adsorption, and DNA retaining/releasing. It is intriguing that these domains are distributed in different tail tip proteins for different phages or tail-like machines (see the section “Structure of the tail tip complex” in the revised manuscript for details).

In regard to the side tail fibers, our recent electron microscopy analysis of ultrathin sections of the lambda-infected E. coli cells showed that the lambda wild-type particles were adsorbed perpendicular to the cell surface, although these particles lacked side tail fibers, suggesting that perpendicular adsorption did not rely on the participation of the side tail fibers (page 15, lines 418-426).

2. Assembly (and structure).

Unlike the laboratory-adapted and confusingly called "wild type" lambda, which lacks side fibers as a result of a laboratory-acquired mutation, the tail of the "real wild type" lambda carries four side fibers. These four side fibers are somehow attached to the sixfold (or threefold?) symmetric tail. How? The mystery of four side fibers remains unsolved.

We thank the reviewer for pointing out the absence of the side tail fiber in our structure. Reviewer 1 raised the same question. The lambda phage we used is a laboratory strain and is called “lambda wild type” (Hendrix RW, Duda RL. Bacteriophage lambda PaPa: not the mother of all lambda phages. Science. 1992;258:1145-8). It lacks side tail fiber proteins (stf and tfa) as a result of a laboratory-acquired mutation. The lack of the side fiber proteins was further confirmed by our recent mass spectrometry experiment (See Table S2).

We have revised the manuscript accordingly (page 4, line 102).

The second big question is the interaction of the DNA with the tape measure protein in the tube channel. In a mature lambda particle, one end of DNA molecule is thought to extend into the tube channel from the capsid. The authors assume it is the case here and they distinguish between the DNA and gpH densities in the tube channel by the presence of a lumen in the latter (in gpH). Such interpretation lacks rigor. Has the length of the DNA fragment, which extends into the channel, been determined experimentally earlier? How does that length correlate with the interpretation of the cryoEM map reported here? One way to distinguish DNA from protein is to use "bubblegram" imaging: <https://www.science.org/doi/10.1126/science.1214120> Perhaps, something like that can be attempted here?

We thank the reviewer for pointing out this question. Reviewer 1 and reviewer 3 also raised the same question. We tried the "bubblegram" experiment but did not observe bubbles in the tail tube (see the figures below). Therefore, we have deleted this part (page 10, lines 260-274).

*Cryo-EM images of the lambda particles with a electron dose of 60 e^-^/Å^2^ (left) and 120 e^-^/Å^2^ (right).*

As a final note on assembly, I would like to point out that one structure, no matter the resolution, cannot explain the process of assembly. And yet the Supplementary Information supplied with this paper does just that - is displays a schematic of particle assembly. It even shows four side fibers that weren't in the particle studied here. The process of lambda particle assembly has been delineated in many publications over the years. Various intermediates have been isolated and characterized. This study contains no new experiments that describe such complexes.

We agree with the reviewer and have deleted the Supplementary Information and Fig. S11.

Besides the major concerns about the presentation of data and extrapolation of those results to subjects that have not been studied in this paper (as outlined above), there are many minor concerns that can be fixed by editing and rewriting. Of those, I will highlight only a few significant ones.

First, the title is confusing. The authors describe the structure of the complete "WT" lambda that is free in solution. Nothing is in situ here. N.B. that a phage particle consists of a head (or capsid, not capsid shell, line 39), a tail, a neck, and tail fibers (or, more generally, receptor-binding proteins). The tail is connected to the head not by a tail-to-head connector, but by a neck. Two neck proteins that interact with the portal - gpW and gpFII - are, in fact, head proteins because they do not bind to isolated tails. They are head maturation proteins. So, these proteins cannot be part of the "in situ" tail, they are neck proteins.

We thank the reviewer for pointing this out. Reviewer 1 also pointed out the same problem and suggested we use “connector” to describe the adaptor and stopper. We have used the term “neck (connector)” to describe the adaptor and stopper in the revised manuscript. We have changed the title to “Symmetry-mismatch structure of the bacteriophage lambda.”

Second, the Introduction is a hodgepodge of sentences and concepts taken from different reviews and other papers. There is no flow and everything is just mixed together. Bacteriophage abundance (which is irrelevant for this paper) is immediately followed by a description of a particle structure with the words "pentameric", "icosahedral", "portal", "vertex", which are highly specialized terms only structure-versed people understand (and not all even). Then the DNA packaging is described in one sentence. Then, we learn about some mysterious conformation of the portal. Nothing about tail assembly though. Then we learn about different tail morphologies (shouldn't we have started with this?). And then, we learn that the tail is important for host recognition and attachment. Some general concepts and highly specialized terms are put together into unreadable sentences.

We have revised this paragraph (page 3).

Third, the Results and Discussion section is all about minute details how this residue interacts with that residue. This is irrelevant for our understanding of particle assembly or infection mechanism. These sort of descriptions should be published in a journal that specializes on protein structure. I strongly suggest removing all the minutia into the supplementary information and compare global properties of lambda tail proteins with those of other phages. By global properties I mean surface properties, hydrophobicity, surface charge distribution, etc. Things that are actually important for function.

We have deleted the minute details on how this residue interacts with that residue. We have moved Figs. S6 and S7 to the main text (the new Figs. 4 and 6).

Two points regarding terminology.

1. Beta-strand chain swapping/insertion is called beta-sheet augmentation, not beta-augmentation. See here: <https://pubmed.ncbi.nlm.nih.gov/16828554/>

We have revised them (page 7, line 184; page 8, line 209; page 13, line 355; legends of the new Figures 3, 5, and 7).

2. Near-atomic resolution describes a dataset in which at least some of the atoms are resolved. That is - the resolution limit must be higher that the length of at least some of the bonds in the studied molecule. For proteins, that would be ~1.4A. Resolutions lower than 1.4A are not near-atomic. The maps presented in this paper are great looking and appear to be easily interpretable in terms of amino acids, NOT atoms, so these maps a really far from "near-atomic".  The highest resolution reported here is 3.2 A. This map contains only 8.3% (!) of the information expected in a near-atomic resolution map, which would be at 1.4 A resolution. All other maps are even further away from being "near atomic".

We have deleted all descriptions of “near-atomic resolution” in the manuscript.

One big positive features of the MS is that the figures are done very well. Very good amount of labels, good placement and clarity. This is much appreciated. However, some of the more important figures are relegated to the Supplementary Information section at the expense of showing electron density of side chains and minutia of interactions that are functionally irrelevant. I think Fig. S3 should be in the main text. Fig. S5 is a good candidate for a main text figure as well. But in addition to the folds, the surfaces must be shown and their properties characterized. It's the surface that performs the function, not the spaghetti of the protein fold. Fig. S6 must be a main text figure. This is the only figure that deals with assembly as it shows how these rings are distinguished based on their electrostatic properties. Fig. S7 must be in the main text. I would vote for Fig. S9 to be in the main text as well. Fig. S11 should be published in a separate review paper.

We have moved Figs. S3, S6, S7, and S9 to the main text and have deleted Fig. S11.

**Reviewer #3:**

The manuscript entitled "In situ structure of the connector and tail of a Siphophage" by Xiao and co-authors has been submitted to the PLOS Biology journal. The authors present the structure of the Siphophage Lambda that infects the bacterial species Escherichia coli (E. coli). The structure has been obtained at resolutions varied from ~3.5 to 6 Angstroms between different components of the phage complex; a structure of the nearly complete phage was solved at 20 Angstrom resolution.  The phage Lambda has been used for nearly two decades as a test object for usage verification methods of the cryo-electron microscopy, and software development.  The authors have achieved some progress in the revelation of structural details allowing to trace the polypeptide chains of several phage proteins. This study provides an additional information on structural details of the Lambda phage and suggests some new ideas related to the phage structural organisation, that might be useful at analysis of other bacteriophages.

However, some issues must be addressed and comparison with the published structures should be done before the publication of this work:

The authors submitted the manuscript with rather unclear title: "In situ structure of the connector and tail of a Siphophage". It is easy to assume that the authors want their manuscript to be cited; in this case they must specify in the title which phage they have analysed. There are so many Siphoviridae phages, so readers will be interested in what was specifically new in this study.

We have changed the title to “Symmetry-mismatch structure of the bacteriophage lambda.”

The authors did not discuss or indicated why this phage was important and should be subjected for this research, since as a test sample this phage was used for quite long time. There are some problems with logistic of the manuscript which is rather inconsistent. In the abstract they move from the tail to the connector-tail complex and its symmetry, then again to the tail. The structure of the head was not mentioned. The mechanism of infection was mentioned, but it was not explained and properly discussed in the manuscript. The main part of the introduction is dedicated to the classification of phages: at the beginning the authors started from the phage head and moved their discussion towards the tails tip, but still it was not explained why it was important to study Siphoviridae phages and, specifically, the Lambda phage.

We have added the importance of studying the lambda phage: “The lambda phage is used as a model system and is important in the attainment of the current understanding of the sophisticated and complex mechanisms by which the expression of genesis is controlled, the understanding of DNA virus assembly, and the understanding of the molecular nature of lysogeny” (page 4, lines 86-90).

We have revised the abstract and introduction.

Results and discussion start from the head and overall organisation of the phage, but then the authors jumped to the connector, tail, tip, and details of structural organisation of the head were not provided. Apparently, a resolution of it was rather low and not comparable with the results published by G. Lander and J. Johnson in 2008 and by Wang C, Zeng J, Wang J. in 2022 (Structural basis of bacteriophage lambda capsid maturation. Structure, 2022 Apr;30(4):637-645.e3. DOI: 10.1016/j.str.2021.12.009. Resolution: 5.03).

We did not provide the details of the structural organization of the head because the high-resolution structure of the lambda head was previously published (Wang et al., *Structural basis of bacteriophage lambda capsid maturation*. Structure. 2022;30). Our models of gpD and gpE, which were built based on our head density map at a resolution of 3.5 Å (See Fig. S2B), are essentially identical to those reported by Wang et al (see the figures below). Our models of gpD and gpE are essentially identical to the subnanometer resolution structure reported by G. Lander et al. (see the figures below).

*Top left: superimposition of our model of gpE on our density map (3.5Å resolution). Right: superimposition of model of gpE (7vik) by Wang et al. on their density map (by using local refinement, 3.7Å resolution, EMD-32011).*

*Bottom Left: superimposition of our gpE model (magenta) with the model by Wang et al. (cyan, PDB ID: 7vik). Middle: superimposition of our gpD model (magenta) with the model by Wang et al. (cyan, PDB ID: 7vik). Right: superimposition of our models of gpD (orange) and gpE (purple) on the density map by G. Lander et al.*

The structure of the Lambda tail at 2.7 A resolution has been reported and deposited to the EMBD database in 2022 (EMD-25611, Prokhorov NS, Yang Q, Catalano CE, Morais MC). No comparison was done with previously reported results.

The model of gpV based on our tail tube density map at a resolution of 3.6 Å is very similar to the model based on the density map at a resolution of 2.7 Å (EMD-25611, see the Figures below).

*Left: superimposition of our model of gpV on our density map. Middle: superimposition of the model of gpV By Prokhorov et al. (PDB ID: 7t2e) on their density map (EMD-25611). Right: superimposition of our model (magenta) with their model (cyan, PDB ID: 7t2e).*

Unfortunately, the methods are not complete: the authors wrote that they have used their own software packages. No information was provided if these packages are available for other users, where are the links to these packages, which parameters were used during structural analysis, how high was percentage of used data in the last reconstructions, where are the atomic models used in the fitting come from. The quality of the structures reported suggests that the authors were not able to trace de novo polypeptide chains, or the accession codes must be provided if the authors used some homologues structures.

The software package used for symmetry-mismatch reconstruction (including the tutorial and test images) can be downloaded from <https://cryoem.hunnu.edu.cn>. We have revised the manuscript accordingly (page 5, lines 125-126).

The percentages of the particle images used in the final reconstructions ranged from 66% to 80% (see the new Table S4).

We did not use homologous structures. Based on our density maps, we built de novo atomic models for the head proteins (gpE and gpD), the portal and neck (including two 12-fold rings of the portal protein gpB and the adaptor protein gpW, and a 6-fold ring of the stopper protein gpFII), the tail tube (including a 6-fold ring of the tail terminal protein gpU and thirty-two 6-fold rings of the tail tube protein gpV), and the tail tip (including a 6-fold ring of the distal tail protein gpM, 3-fold trimers of the hub protein gpL, insertion protein gpI, central fiber protein gpJ, and a C-terminal α-helix of the tape measure protein gpH) (see the new Table S4).

What were the RMSDs between different portal proteins when the authors have done alignments: Figure S5, second raw.  Where was the alignment done and according to which features? The authors have used for the SPP1 the atomic model of the 13-mer, why the authors did not use the 12-mer? The latest structure is of better quality, see  7Z4W (gp612 - gp1512 - gp166 complex).

We aligned the portal proteins using the MatchMaker of UCSF Chimera. The RMSDs between the portal proteins of lambda and SPP1, T7, and T4 are 2.52, 2.48, and 2.03 Å, respectively. We have replaced the SPP1 atomic model of the 13-mer with 12-mer (7Z4W) in Fig. S5 (now is the new S4 Fig), and revised the figure legend accordingly.

It is interesting that the authors suggested that the tape measure protein and DNA are interwinding within the central channel of the tail. One can wonder on which evidences this suggestion was made. Do the authors have any biochemical evidence that the first pieces of the ejected DNA are intertwined with the tape measure protein? Does the tape measure protein is injected into the host cells?  Did the authors measured the diameter of the inner tube of the tails? it seems that it is significantly smaller if one will compare the diameter of the dsDNA complexed with the tape measure proteins … Would be essential to provide assessments of these values.

We have deleted the suggestion for the type measure protein gpH (page 10, lines 260-274).

The authors have to provide the standard table where the overall information on the data collection will be given, the quality of the fittings, how the models were obtained, values of the correspondence between maps and polypeptide chains, RMSD, Molprobity scores, and results from the Ramachandran plots (for all fitted proteins).

We have provided the information in the new Table S4.

The part of "the Cryo-EM and data collection" is very confusing, it seems that the authors are not familiar neither with the equipment they have used nor with the requirements for the publications. Typically, Quantifoil R2/1 copper grids (line 315) have the 2-nm thick continues carbon film, not 5-nm. If the authors have used 5 nm thick carbon layer, that have to be explained. and the proper number for this type of grids should be provided (see this and the other websites <https://www.jenabioscience.com/about-us/news-blog/3342-cryo-em-grids-available)>.

We thank the reviewer for their critical review of our Materials and Methods.

The thickness of the Quantifoil Holey Carbon Film we used was about 20 nm (<https://www.tedpella.com/calibration_html/QUANTIFOIL_TEM_Substrates.aspx>). Our initial cryo-EM results showed that a small number of lambda particles entered the holes in the grid. To let the phage particles enter the holes, we covered the Quantifoil grids with a 5-nm-thick carbon film using a turbomolecular pumped coater (Q150TE, Quorum, UK).

We have revised the description and provided the information of the mesh number of the quantifoil grids (page 17, lines 480-484).

They have used unusual terminology "double-blotting". That must be explained what the authors have meant.

We have changed the term “double-blotting” to “After blotting the excess sample solution on the grid two times” (page 17, line 485).

The information on GIF is not complete. The microscope was equipped with a Gatan Quantum energy filter. What was the slit used at the data collection? What was the voltage in the microscope? What was a defocus range used at the data collection? The authors have to check how the magnification was consistent with the pixel size information. What was written in the MS is not consistent and does not correspond to the information given on websites of electron microscopes.  Was the pixel size calibrated? If it was done, that has to be explained.

The Gatan Quantum energy filter with a slit width of 20 eV was used to remove inelastically scattered electrons, the electron microscope was operated at a voltage of 300 kV, and the defocus range was from 1.6 to 2.2 μm. The K3 detector was operated in the super-resolution mode, and automatic data collection was performed using the FEI EPU software with a magnification of 53,000 (physical pixel size: 1.36 Å) in the "super-resolution" mode, which resulted in a pixel size of 0.68 Å. The pixel size was calibrated using 500-nm diffraction grating replica and latex calibration standard.

We have revised the manuscript accordingly (page 17, lines 488-495).

Image processing: what was a size of boxes for the processing of the complete phage images? Sizes pf the boxes should be given in pixels (not in the angstroms) with indication of the pixel sizes. It seems that at analysis of the different elements of the phage the authors have used different pixel sizes and box sizes.

The box size for the images of the complete phage is 900×900 pixels (resampled to a pixel size of 5.44 Å). The box sizes for the tail tube images and tail tip images are 220×220 pixels. The box size for the neck images are 400×400 pixels.

We have revised the manuscript accordingly (page 18, lines 503-505; page 18, line 525; page 19, line 532; page 19, line 535).

What was special in analysis of the symmetry mismatch: the authors were "using our symmetry-mismatch reconstruction method". No details were provided on specific features of this approach.

The software package for symmetry-mismatch reconstruction (including the tutorial and test images) can be downloaded from <https://cryoem.hunnu.edu.cn>. We have revised the manuscript accordingly (page 5, lines 125-126).

The authors did not provide any details on what has been found. What kind interactions were revealed between proteins at pseudo equivalent positions? The authors did not explain how the portal proteins interact with the capsid.

We added the description of the asymmetric interactions between the portal and capsid (pages 6 and 7, lines 162-168), which is similar to those observed in structures of phages T4 and sf6 (Fang Q. et al. *Structural morphing in a symmetry-mismatched viral vertex*. Nat Commun. 2020,11:1713; Li F. et al. *High-resolution cryo-EM structure of the Shigella virus Sf6 genome delivery tail machine*. Sci Adv. 2022,8:eadc9641).

We also added the description of the pseudo 6-fold symmetrical interaction between the gpM ring and gpJ-gpL complex in the tail tip (page 11, lines 290-294). The hexameric gpM ring exihibits a pseudo 6-fold symmetry in order to adapt the interaction with the 3-fold symmetrical gpJ-gpL complex.

In general, the authors did not provide any conclusion related to their research. What have they found?

We have enhance the manuscript by adding structural comparisons between the tail tips of lambda and other long-tail phages or tail-like machines. Our results suggest that their tail tip contains conserved domains that facilitate tail assembly, receptor binding, cell adsorption, and DNA retaining/releasing. It is intriguing that these domains are distributed in different tail tip proteins in different phages or tail-like machines (see the section “Structure of the tail tip complex” in the revised manuscript for details).

The structural similarity between the tail tips of lambda and T5 (Linares et al. *Structural basis of bacteriophage T5 infection trigger and E. coli cell wall perforation*. Science Advances. 2023;9) suggests that the upon the receptor interaction, the tail tip of lambda would undergo similar structural changes upon the receptor interaction similar to that of T5. The tail tips of lambda and T5 share common domains of HDI–IV, HDII-insertion, plug, and two fibronectins (FNIIIs). For T5, all these domains are located in the baseplate hub protein pb3. By contrast, for lambda, the HDI is located in gpL; the HDII, HDIII, HDIV, two FNIIIs, and the C-terminal region of HDII-insertion are located in gpJ; the N-terminal region of HDII-insertion is located in gpL; and the plug is located in gpI. It is noteworthy that gene I is located between genes L and J, a gene location that corresponds to the position of the T5 pb3 plug domain, except for an inserted gene K (see the new Fig. S9). Therefore, we suggest that gpL, gpI, and the N-terminal region of gpJ (from HDII to FNIII-1 and -2) together correspond to T5 BHP pb3. In addition, the central fiber protein (pb4) and receptor binding protein (pb5) of T5 correspond to the central fiber domain and receptor binding domain of lambda gpJ, respectively.

In addition, our recent electron microscopy analysis of ultrathin sections of the lambda infected E. coli cells showed that the lambda wild type particles were adsorbed perpendicular to the cell surface, although these particles lacked side tail fibers, suggesting that perpendicular adsorption did not rely on the participation of the side tail fibers (S11 Fig, page 15 lines 418--426).

The authors did not provide reports from the EMDB database, where the quality of the structures and fitting of the atomic models would be given. This information is essential for the result quality assessments.

We have uploaded the structures to PDB and EMDB. The reports from the EMDB database are included in the supporting information with the manuscript submitted.

Minor comments:

The authors should show FSC curves for all reconstructions and indicate what was a resolution at 0.5 and at 0.143 levels.

We have indicated the resolutions at 0.5 and at 0.143 levels in the FSC curves (see the new Fig. S1B).

Please rephrase: "The portal is surrounded by 12 copies of gpB, which exhibits  the canonical portal fold of phages …". The portal is not "surrounded"… it is composed by 12 copies of the protein …

We have revised it (page 6, lines 154-155).

The authors did not provide the info on how symmetries of the different parts of the tail were verified.

The symmetries of the different parts of the tail were verified by the high resolution of the structures we obtained. The high resolution structure could not have been obtained if the symmetry was incorrectly applied during the reconstruction.

Portal proteins do not have a "funnel" shape.

We have deleted it (page 6, line 151).

Lines 209-215. "…the rod-like structure below the third gpV ring was hollow..." The authors have to indicate how big were the changes in the inner diameter of the tale tube, how big were changes in the densities of the tail rings and how much their diameters were changes?  The sentence on lines 211-213 is not justified, since not information was provided.

We agree with the reviewer. Reviewer 1 and reviewer 2 also raised the same question. We have deleted this part (page 10, lines 260-274).

Lines 219-222, The statement is not supported by any biochemical information.

We agree with the reviewer. Reviewer 1 and reviewer 2 also raised the same question. We have deleted this statement (page 10, lines 260-274).

The authors write that "all data are fully available without restriction ". The data are not, available, no information on deposition was provided, no reports on the structure qualities and fittings.

We have uploaded the structures to PDB and EMDB. The reports from the EMDB database are included in the supporting information with the manuscript submitted (see Table S4).

Line 293. A part of the sentence is confusing, should be rephrased: "…formed  by a cascade of gpH trimer…" . Here there is a linguistical problem: of which "cascade" are the authors talking? Is it a process, or a set of structural elements?

We have deleted “a cascade of”.

The English has to be checked.

We have checked the English of the manuscript again.
