# Supplementary material for: Structure of the siphophage neck–Tail complex suggests that conserved tail tip proteins facilitate receptor binding and tail assembly
Source: PLoS Biol. 2023 Dec 14;21(12):e3002441. doi: 10.1371/journal.pbio.3002441 (PMC10721106; doi:10.1371/journal.pbio.3002441)
Supplement: Attachment — Submitted filename: ResponseToReviewers.docx [file pbio.3002441.s024.docx]

We would like to thank the reviewers for their critical review of our work and for their constructive suggestions on how to improve our manuscript. All comments and suggestions have been addressed, and the manuscript has been revised accordingly.

In accordance with the editor’s suggestion, we have modified the main title and reduced the number of main figures in the manuscript to 4. In accordance with the reviewer 3’s suggestion, we have reconstructed the tail tube by imposing 3-fold symmetry. We found that six copies of the C-terminal domain of gpV formed 3-fold symmetrical protrusions, which occurred because two neighboring C-terminal domains of gpV intertwined, forming a single protrusion on the ring. The previous structure of the tail tube, in which six protrusions were observed on each ring, was an artifact of the C6 symmetry imposed during reconstruction. We have revised Figs. 2 and 6 (new Figs 1 and S8) and the main text (page 10, lines 259-262) accordingly (please refer to our response to the last question).

The page and line numbers outlined in this document correspond to those in the revised manuscript with tracked changes (manuscript_changes_tracked.pdf).

**Reviewer #2:**

The new version of the MS, now titled "Symmetry-mismatch structure of the bacteriophage lambda", has been significantly improved, but the quality of the delivery of information is still not great. The Abstract and Intro are still a little awkward, and the main text consists of long, information-dense paragraphs without subsections. The existing sections can be divided into subsections with subsection titles, and long paragraphs (almost all of them!) should be divided into smaller paragraphs. This is beyond the duty of a reviewer to fix things like this in a paper this long and this information rich.

We have divided the subsection titled “Structure of the tail tip complex” into three subsections and have added two subsection titles of “Structural comparison of gpI, gpL, and gpJ with their counterparts in other phages and phage tail-like machines” and “Ultrathin sections of lambda-infected cells”. We have also divided long paragraphs into smaller ones.

For this reason, only a few easy fixes and suggested edits are listed below.

I suggest changing the existing, somewhat odd-sounding title to one of the following three:

Symmetry mismatches in the bacteriophage lambda tail

Atomic structure of the bacteriophage lambda tail

Atomic structure of bacteriophage lambda

According to the editor’s suggestion, we have changed the title to “Structure of the siphophage neck-tail complex suggests that conserved tail tip proteins facilitate receptor binding and tail assembly”.

Abstract

25  present the in situ structure of the neck-tail complex of the siphophage lambda wild type, which lacks side tail fiber.

Consider: …of the siphophage lambda "wild type", the most widely used, laboratory-adapted fiberless mutant.

We have revised it in accordance with the reviewer’s suggestion.

28  of 246 tail protein molecules responsible for neck-tail assembly and stabilization have been

…of 246 tail protein molecules forming the tail and neck have been

We have revised it in accordance with the reviewer’s suggestion.

29  characterized. The DNA in the neck is clamped by the adaptor protein rather than the stopper

30  protein.

We do not know what an adaptor and stopper are at this point, so this sentence is confusing.

We have deleted this sentence.

34  The side tail fibers are not required for the perpendicular

35  adsorption of lambda.

Consider: The side tail fibers are not required for the phage particle to orient itself perpendicular to the host cell surface during attachment.

We have revised it in accordance with the reviewer’s suggestion.

Intro

39  The majority of known phages belong to the order Caudovirales and contain a tail attached to

40  the pentameric vertex of the icosahedral capsid (head) through a portal or through a portal in

41  complex with neck (connector) proteins [2].

Consider: The majority of known phages belong to the order Caudovirales and contain a tail attached to the portal protein located in a unique vertex of the capsid [2].

We have revised it to “The majority of known phages belong to the order *Caudovirales* and contain a tail attached either to a portal alone or to a portal in complex with a neck (connector) located in a unique vertex of the capsid” (page 3, lines 44-46).

79  Here, we present the cryo-EM structure of the phage lambda, which is a laboratory-

80  adapted strain and is called "lambda wild type" [35].

Consider: Here, we present the cryo-EM structure of the laboratory-adapted mutant of phage lambda, commonly known as lambda "wild type". This mutant lost its host cell-binding side tail fibers as a result of laboratory adaptation [35].

Also consider replacing "strain" with "mutant" throughout when you describe various flavors of lambda. But this is your call - maybe "strain" is more appropriate, I am not sure.

 We have revised it (page 4, lines 85-88). We have replaced "strain" with "mutant" in the manuscript when describing lambda.

Fig 4 needs work.

The legend is too brief.

The two columns must be labeled "toward head" and "toward tail tip" or "head-facing side" and "tail tip-facing side".

The color bar showing the scale of e-potential must be given in kT/e.

It must be explicitly stated in the figure legend whether the coloring scheme - the color scale of the e-potential range - is the same for all surfaces.

We have revised the Fig. 4 (new Fig. S7) and the figure legend in accordance with the reviewer’s suggestion.

Fig. 6E. The color bar showing the scale of e-potential must be given in kT/e.

We have revised the Fig. 6E (new Fig. S8) in accordance with the reviewer’s suggestion.

Fig. 8. All proteins must be labeled with their trivial names, if available, or gene product names/numbers. PDB IDs can and do change. Proteins get renamed also, but this is less often.

For example, the label T4 must be changed to T4 gp27. T6SS is T6SS VgrG N-terminal domain (or module). Also, in the figure legend, state which species VgrG belongs to.

The same is applicable to Fig. S6. All proteins in the figure must be labeled with their names or gene product numbers (e.g. T4 is actually T4 gp48, I assume?).

We have revised the Fig. 8 (new Fig. S11) and Fig. S6 (new Fig. S9) in accordance with the reviewer’s suggestion.

**Reviewer #3:**

The authors have done (before the first revision) significant amount of analysis and spend a lot of energy on interpretation of the results. However, it is still rather difficult to read the revised MS. What was new and different from the previously published structures? The authors did not do the proper comparison of their own results with them. While the paper was improved in sense of terminology, but, unfortunately, the MS has to go to the next round of modifications and revised mainly in the part related to methods.

Comparison of the tails structures and interpretation should be completed: what was RSMD between this new structure of the tail protein (gpV) and deposited to the EMBD database in 2022 (EMD-25611)? RMSD of 3A for the entire structure indicates noticeable  differences (since this is an average value), where are the differences located? The authors were not the first who have obtained this structure. Please make the proper citations.

Our structure is essentially identical to the cryo-EM structure of the lambda tail tube deposited by another group in the EM Data Bank (EMD-25611, to be published) and Protein Data Bank (PDB ID: 7T2E, to be published) with a RMSD of 0.45 Å. We have provided a comparison in the main text (page 10, lines 256-259).

Sectioning of cells. The procedure of sample preparation for the sectioning of the cell infected by phages is not fully described. Which cells were used in this experiment? What were the concentrations of cells and phages used at sample preparations? What was the magnification? Why the authors insist that the tails (being so flexible) must be perpendicular to the surface of the host cell? It is not an essential requirement for the infectivity according to the figure S11 and in general for phages, that are very effective.

We have provided the detailed procedure of sample preparation for sectioning cells infected with phages (page 17, lines 481-485).

We agree that perpendicular adsorption on the cell surface may not be necessary for the phage infectivity. Therefore, we have revised the main text accordingly (pages 16 and 17, lines 456-464).

The procedure of the ab-initio fitting is still not fully described as well. Did the authors fit proteins as monomers? If yes, how they have defined densities for one monomer? It seems that the authors did some homology modelling, if they have done matching with other phage proteins.  The authors have to describe honestly what was done.

The resolutions of our structures of the portal, neck, tail tube, and tail tip range from 3.2-3.5 angstrom. We built ab initio atomic models for these lambda proteins and did not reference any homology protein.

Reference on 7Z4W coordinates was not given. See for the reference  https://doi.org/10.1038/s41467-022-34999-8.

We have cited the paper (page 3, line 66; page 11, line 285; reference 27).

The interpretation of the  symmetry in the portal/neck complex is rather confusing. The authors write in the answers to reviewers that they have done reconstructions with different symmetries  and the resolution obtained indicated what sort of symmetry had a part of the complex.  However it is not very efficient approach, since there are links between C12 , C6 and C3 symmetries. Applying high rotational symmetries one will be able to see details of the components with high level of symmetry but the info related to the links between components  of low-rank symmetry and components  of high rank symmetry will be not interpretable.

We agree that implementing rotational symmetry may result in uninterpretable structural information regarding the links between components of low-rank symmetry and components of high-rank symmetry.

We noticed this problem and carefully resolved this type of structures in two regions. The first region was the link between the hexameric gpM ring and the 3-fold symmetric tail tip. As shown in Fig. 4D, the E-loop of gpM, which interacted with the tail tip, exhibited two conformations, thus enabling adaption to C6-C3 symmetry mismatch. The second region was the hexameric ring of gpV. Six copies of the N-terminal domain of gpV formed the 6-fold symmetrical tail tube ring, whereas six copies of the C-terminal domain of gpV formed 3-fold symmetrical protrusions, which occurred because two neighboring C-terminal domains of gpV intertwined, forming a single protrusion on the ring (Fig 1A and Fig. S8B; page 10, lines 259-262; please refer to our response to the last question).

The order of figures is rather messy: the order of their numbering does not correspond to the order of referencing them in the text. Please check it.

We have reorganized both the main figures and the supplementary figures. In accordance with the editor’s suggestion, we have also reduced the number of the main figures in the manuscript to 4, and have moved the other figures to the Supporting Information.

The following comments will be labelled according to the version PBIOLOGY-D-23-00002_R2.pdb, the part with the corrections.

Thank you for helping us improve our manuscript.

Lines 110-113. The phrase is confusing: "…the major domains in the tail tips are structurally conserved, although they are distributed in different tail tip proteins in different phages or tail-like machines". Please rephrase and explain it in the conclusions. The word "although" does not fit to the idea which the authors want to deliver.

The phrase has been modified, as follows: “… the major domains in the tail tips are structurally conserved; however, these domains are distributed in different tail tip proteins in various phages or tail-like machines” (page 4, lines 95 and 96).

The stopper does not clamp DNA of the phage, it may block the exit (abstract, line 33).

We have deleted this sentence.

Lines 160-162. "The tunnel loop, which was previously indicated to clamp dsDNA to avoid leakage during the phage maturation". The idea is not consistent with the other publications, where it was suggested and proved by biochemical experiments by mutations, that the portal complex is a driving motor, that pumps DNA into the procapsid. The tunnel loops help to move the DNA inside, they do not clamp DNA.

We agree that tunnel loops aid in moving DNA during DNA packaging. Tunnel loops also stabilize dsDNA to avoid leakage during phage maturation (Rao et al., The remarkable viral portal vertex: structure and a plausible model for mechanism. *Curr Opin Virol* 2021, 51:65–73; Dedeo et al., Portal Protein: The Orchestrator of Capsid Assembly for the dsDNA Tailed Bacteriophages and Herpesviruses. *Annu Rev Virol* 2019. 6:141–60).

We have revised our manuscript and have added three references accordingly (References 34, 41, and 42; page 7, lines 175-177).

Lines 248-254. The information written in these lines is not quite correct.  Tunnel loops do not clamp the DNA. The stopper in SPP1 is not closed by the loops of gp16 but helices. The refence should be updated.

As mentioned earlier, tunnel loops stabilize dsDNA to avoid leakage during phage maturation. We have changed “clamp” to “stabilize” in the main text.

The new cryo-EM structure indicated that the stopper in SPP1 is closed by the helices of gp16 (reference 27). We have revised the main text and updated the reference (page 11, line 291).

What is it "solid-liquid ethane" (line 486)?  Something is wrong: was it slush ethane? Nowadays it really unusual to use such ethane. However, if they authors had bits of solid ethane during the grid freezing, the grids could be crumpled which would be not surprising and that will create problems at data collection.

We have deleted the “solid-“.

Where is the gpD protein in the table S1? As it has been mentioned before the terminology should be corrected: not  a "cement protein", but  a "cementing protein", see doi: 10.1016/j.str.2008.05.016. The length of the protein is 110 aa and mol w. 29.67 kDa, it would be recommended to mention that in the tables.

We have changed the “cement protein” to “cementing protein” throughout the manuscript. We have cited the paper (doi: 10.1016/j.str.2008.05.016; reference 33).

In addition, we have added gpD and gpE to Table S1.

Line 484. FEI Vitrobot ->  should be Thermo Fisher Scientific (TFS) Vitrobot

Revisions have been made in accordance with the reviewer’s suggestion.

Line 487. 300 kV Titan Krios G3i -> should be Thermo Scientific Krios G3i Cryo-TEM

Revisions have been made in accordance with the reviewer’s suggestion.

Line 488 The K3 Gatan camera is not Summit, such name has only K2 camera.

Revisions have been made in accordance with the reviewer’s suggestion.

Line 492. It is wrong expression:"Automatic data were …" -should be : "data collection was done automatically using the TFS EPU software …."

Revisions have been made in accordance with the reviewer’s suggestion.

Line 493. For the pixel size 1.36A magnification given by the authors is wrong, it should be higher, more as 60 000,possibly much higher. Please check, what was it.

We have confirmed that the magnification of 53,000 corresponds to the pixel size of 1.36 Å.

Line 501. What was a reason to have such huge box size as 900x900  or (if the pixel size was 5.44 A ) = 4,900x4.900 A, if the overall size of the phage was ~2,400A (according to Fig 1)?

We used a large box because we selected the icosahedral head center as the reconstruction center. This box size was approximately double that of the entire lambda particle.

Fig 6. "… (B) Density map of the tail tube filtered to a resolution of 6 Å. Superposition of an atomic model of gpVC (PDB DI: 2L04) on the protrusion (right)" . It is confusing. It seems that the fit of the extra domain of gpV was not done in a best way (it seems to be tilted in a wrong direction)since it was not resolved. Different domains should be labelled in the figure.

Thank you for drawing our attention to this problem. After we refitted the gpV**_C_** domain, we discovered that each protrusion fitted well with the two copies of gpV**_C_** atomic models (see new Fig. S8B), indicating that each protrusion contained two gpV**_C_** domains from two neighboring gpV subunits. The previous structure of the tail tube, in which six protrusions were observed on each ring, was an artifact of the C6 symmetry imposed during reconstruction. We have revised Figs. 2 and 6 (new Figs 1 and S8) and the main text (page 10, lines 259-262) accordingly and labeled the domains in the new Fig. S8B.
